# Prognostic Value of a Novel Signature With Nine Hepatitis C Virus-Induced Genes in Hepatic Cancer by Mining GEO and TCGA Databases

**DOI:** 10.3389/fcell.2021.648279

**Published:** 2021-07-16

**Authors:** Jianming Wei, Bo Wang, Xibo Gao, Daqing Sun

**Affiliations:** ^1^Department of General Surgery, Tianjin Medical University General Hospital, Tianjin, China; ^2^Department of Paediatric Surgery, Tianjin Medical University General Hospital, Tianjin, China; ^3^Department of Dermatology, Tianjin Children’s Hospital, Tianjin, China

**Keywords:** hepatic cancer, hepatitis C virus-induced genes, signature, prognosis, bioinformatics

## Abstract

**Background:**

Hepatitis C virus-induced genes (HCVIGs) play a critical role in regulating tumor development in hepatic cancer. The role of HCVIGs in hepatic cancer remains unknown. This study aimed to construct a prognostic signature and assess the value of the risk model for predicting the prognosis of hepatic cancer.

**Methods:**

Differentially expressed HCVIGs were identified in hepatic cancer data from the Gene Expression Omnibus (GEO) and The Cancer Genome Atlas (TCGA) databases using the library (“limma”) package of R software. The protein–protein interaction (PPI) network was constructed using the Cytoscape software. Functional enrichment analysis was performed using the Gene Ontology (GO) and Kyoto Encyclopedia of Genes and Genomes (KEGG) pathways. Univariate and multivariate Cox proportional hazard regression analyses were applied to screen for prognostic HCVIGs. The signature of HCVIGs was constructed. Gene Set Enrichment Analysis (GSEA) compared the low-risk and high-risk groups. Finally, the International Cancer Genome Consortium (ICGC) database was used to validate this prognostic signature. Polymerase chain reaction (PCR) was performed to validate the expression of nine HCVIGs in the hepatic cancer cell lines.

**Results:**

A total of 143 differentially expressed HCVIGs were identified in TCGA hepatic cancer dataset. Functional enrichment analysis showed that DNA replication was associated with the development of hepatic cancer. The risk score signature was constructed based on the expression of *ZIC2*, *SLC7A11*, *PSRC1*, *TMEM106C*, *TRAIP*, *DTYMK*, *FAM72D*, *TRIP13*, and *CENPM*. In this study, the risk score was an independent prognostic factor in the multivariate Cox regression analysis [hazard ratio (HR) = 1.433, 95% CI = 1.280–1.605, *P* < 0.001]. The overall survival curve revealed that the high-risk group had a poor prognosis. The Kaplan–Meier Plotter online database showed that the survival time of hepatic cancer patients with overexpression of HCVIGs in this signature was significantly shorter. The prognostic signature-associated GO and KEGG pathways were significantly enriched in the risk group. This prognostic signature was validated using external data from the ICGC databases. The expression of nine prognostic genes was validated in HepG2 and LO-2.

**Conclusion:**

This study evaluates a potential prognostic signature and provides a way to explore the mechanism of HCVIGs in hepatic cancer.

## Introduction

Hepatocellular carcinoma (HCC) is the sixth most common cancer and was the third leading cause of cancer-related deaths worldwide in 2020 (Sung et al., 2021). The incidence of HCC has increased sharply over the recent decades (Zhang et al., 2012; McGlynn et al., 2021). The 5−year overall survival (OS) rate of HCC is still unsatisfactory, and its pathogenesis remains controversial.

The development of liver cancer is a complex process, including chronic inflammation and epigenetic modifications (Plissonnier et al., 2018). Hepatitis C virus (HCV) infection is a prominent risk factor for the development of HCC (Ali et al., 2011). Each year, 4–5% of patients with chronic hepatitis C develop HCC (Bartosch et al., 2009). The combination of host, environmental, and viral factors results in HCV-related carcinogenesis (Vescovo et al., 2016). HCV enhances the invasiveness of HCC *via* epidermal growth factor receptor (EGFR)-mediated invadopodia formation and activation (Ninio et al., 2019). Many non-coding RNAs (ncRNAs) play important roles in biological processes, such as differentiation, proliferation, and cell death, in HCC (Yang et al., 2014; Refai et al., 2019). However, the molecular mechanism of HCV-induced genes (HCVIGs) in hepatocarcinogenesis is not well-understood.

To explore HCVIGs in hepatic cancer, we analyzed HCV-induced expression profiling of hepatic and non-hepatic cancer cell lines from GSE70781 and identified a total of 1,582 differentially expressed genes (DEGs). Then, a total of 1,286 DEGs were common genes in the Gene Expression Omnibus (GEO) and The Cancer Genome Atlas (TCGA) databases. We extracted 1,284 DEGs from TCGA hepatic cancer gene expression, including 407 hepatic cancer tissues and 58 adjacent normal tissues. Gene Ontology (GO) enrichment and Kyoto Encyclopedia of Genes and Genomes (KEGG) pathway analyses were performed to analyze the molecular processes. We used univariate Cox proportional risk regression analysis to identify 21 prognostic HCVIGs from TCGA hepatic cancer dataset. To predict the prognosis of hepatic cancer, we constructed nine prognostic HCVIGs signatures using *ZIC2*, *SLC7A11*, *PSRC1*, *TMEM106C*, *TRAIP*, *DTYMK*, *FAM72D*, *TRIP13*, and *CENPM*. The risk score was identified as an independent factor. OS analysis showed that patients in the low-risk group had a better prognosis than those in the high-risk group according to the median risk score. Finally, the International Cancer Genome Consortium (ICGC) databases were analyzed to validate the prognostic signature. Polymerase chain reaction (PCR) revealed that *ZIC2*, *SLC7A11*, *PSRC1*, *TMEM106C*, *TRAIP*, *DTYMK*, *FAM72D*, *TRIP13*, and *CENPM* were upregulated in the hepatic cancer cell line HepG2.

## Materials and Methods

### The Workflow of the Model Construction Process

**FIGURE 1:**
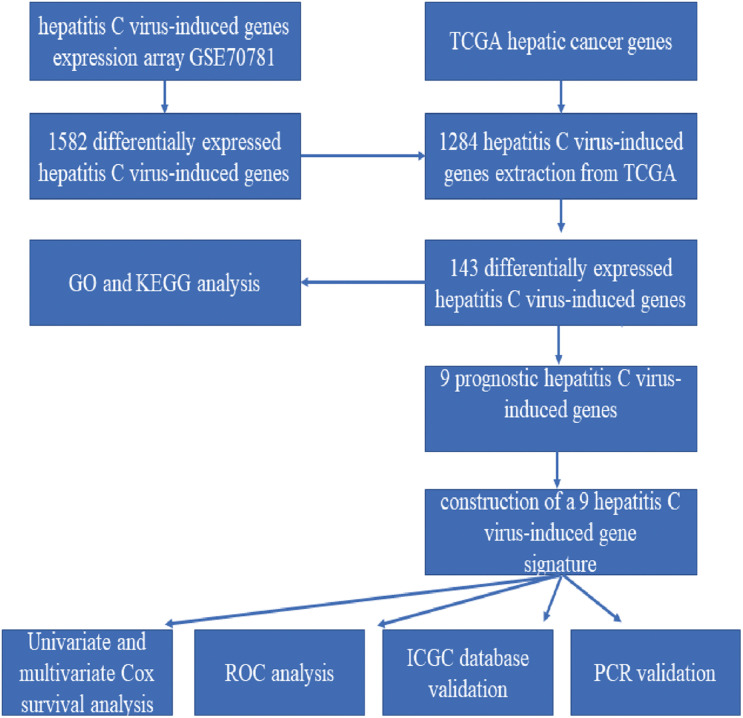
The workflow of the model construction process.

The detailed workflow is shown in Figure 1.

### Data Acquisition and Processing

Hepatitis C virus-induced genes (HCVIGs) were downloaded from the GEO^1^ including hepatic and non-hepatic cancer cell lines in GSE70781. The original gene expression profiles and clinical data of hepatic cancer were obtained from TCGA data portal^2^. Differentially expressed HCVIGs from the GEO database were extracted using TCGA data. The JP Project from the International Cancer Genome Consortium (ICGC-LIRI-JP) was downloaded from the Database of Hepatocellular Carcinoma Expression Atlas (HCCDB) for external validation.

### Function Annotation and Gene Set Enrichment Analysis

Differentially expressed HCVIGs were identified using the limma package. Fold change > 2.0 and adjusted <0.05 were selected as the significantly differentially expressed HCVIGs. GO is a community-based bioinformatics resource that includes biological processes (BP), cell components (CC), and molecular functions (MF) (Peng et al., 2017). The KEGG is a knowledge base for systematic analysis of gene functions, linking genomic information with higher-order functional information (Kanehisa and Goto, 2000). GO and KEGG enrichment results were generated by R package “ggplot2,” “enrichplot,” “clusterProfiler,” and “GOplot” for the purpose of analysis. Gene Set Enrichment Analysis (GSEA) comparing the low-risk and high-risk groups was performed by GSEA software (version 4.0.3). Functional annotation with a *P*-value < 0.05 was considered statistically significant.

### PPI Network and Hub Genes Analysis

STRING database (Szklarczyk et al., 2017)^3^ was used to analyze the functional interactions between expressed proteins. The STRING network was then input to Cytoscape software (version 3.6.0) (Yang et al., 2019) to construct a protein–protein interaction (PPI) network of 143 differentially expressed HCVIGs. The top 10 genes were selected using the CytoHubba application (Zhou et al., 2019).

### Construction of the Nine Prognostic HCVIGs Signature

Differentially expressed HCVIGs were selected to construct a multivariate Cox regression model. OS curves were plotted using the Kaplan–Meier (K–M) method. The relationships were tested using the log-rank test. The nine prognostic HCVIGs-associated signature was constructed using coefficients in multivariate Cox regression analysis (Qiu et al., 2020). Nine prognostic values of HCVIGs were validated using the Kaplan–Meier Plotter (Menyhárt et al., 2018).

### Real-Time RT-qPCR Assay

Hepatic cancer cell line HepG2 and normal hepatic cell line LO-2 were used to test the expression of nine prognostic values of HCVIGs. Real-time RT-qPCR experiments were performed as previously described. The primer sequences used are shown in Supplementary Table 1. Relative mRNA levels after correction for GAPDH control mRNA were expressed.

### Statistical Analysis

R software (version 3.5.2) was used to perform all statistical analyses in this study. Statistical significance was set at *P* < 0.05. Univariate Cox regression analyses were performed to assess the relationship between expression profiles and prognosis. Multivariate Cox regression was used to construct a risk model according to the clinical factors correlated with survival. Receiver operating characteristic (ROC) curve and the corresponding area under the ROC curve (AUC) were analyzed by the package of “survivalROC” in R.

## Results

### Identification of Differentially Expressed HCVIGs

**FIGURE 2:**
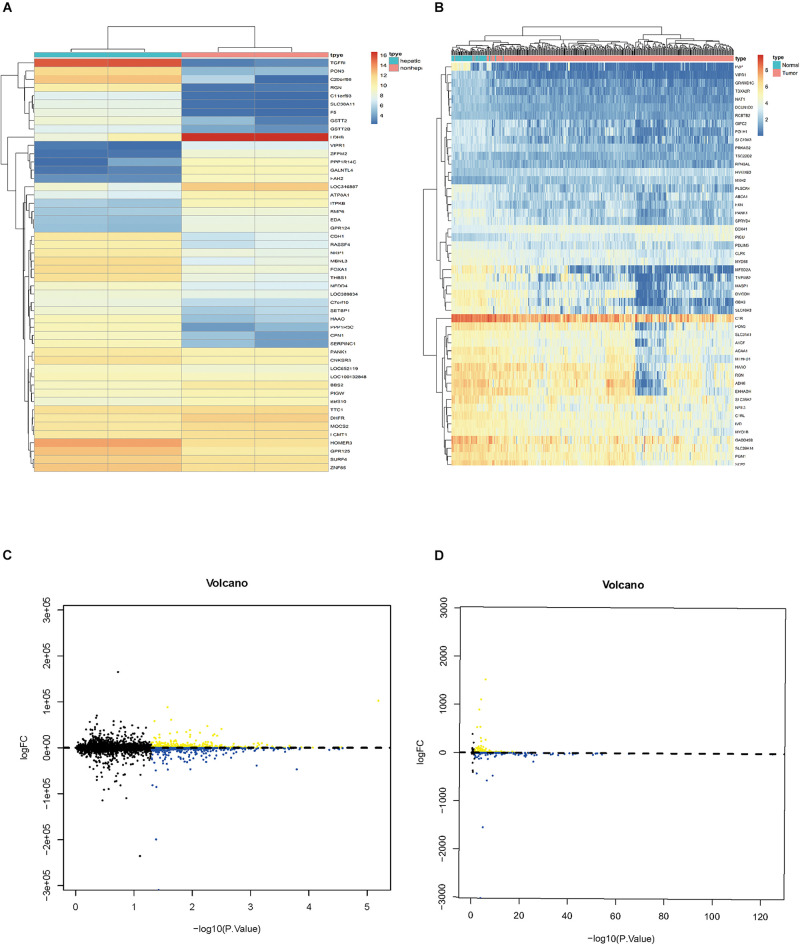
Identification of differentially expressed hepatitis C virus-induced genes. **(A)** Heat map of differentially expressed HCVIGs expression levels in GSE70781. **(B)** Volcano plot for differentially expressed HCVIGs expression levels in GSE70781. **(C)** Heat map of differentially expressed HCVIGs expression levels in TCGA dataset. **(D)** Volcano plot for differentially expressed HCVIGs expression levels in TCGA dataset.

A total of 1,582 differentially expressed HCVIGs were identified using the R language software in GSE70781. Among the differentially expressed HCVIGs, 744 were downregulated, whereas a total of 838 were upregulated. These results are presented as a heat map (Figure 2A) and a volcano plot (Figure 2C). We obtained the gene expression and clinical data for all 465 patients, including 407 hepatic cancer tissues and 58 adjacent normal tissues from TCGA database. A total of 1,286 differentially expressed HCVIGs were common genes between GSE70781 and TCGA. A total of 1,284 differentially expressed HCVIGs were extracted from TCGA database. Finally, a total of 143 differentially expressed HCVIGs were selected from TCGA, including 2 low-expression genes and 141 over-expression genes, as shown in the heat map (Figure 2B) and the volcano plot (Figure 2D).

### GO Enrichment and KEGG Pathway Analysis

**FIGURE 3:**
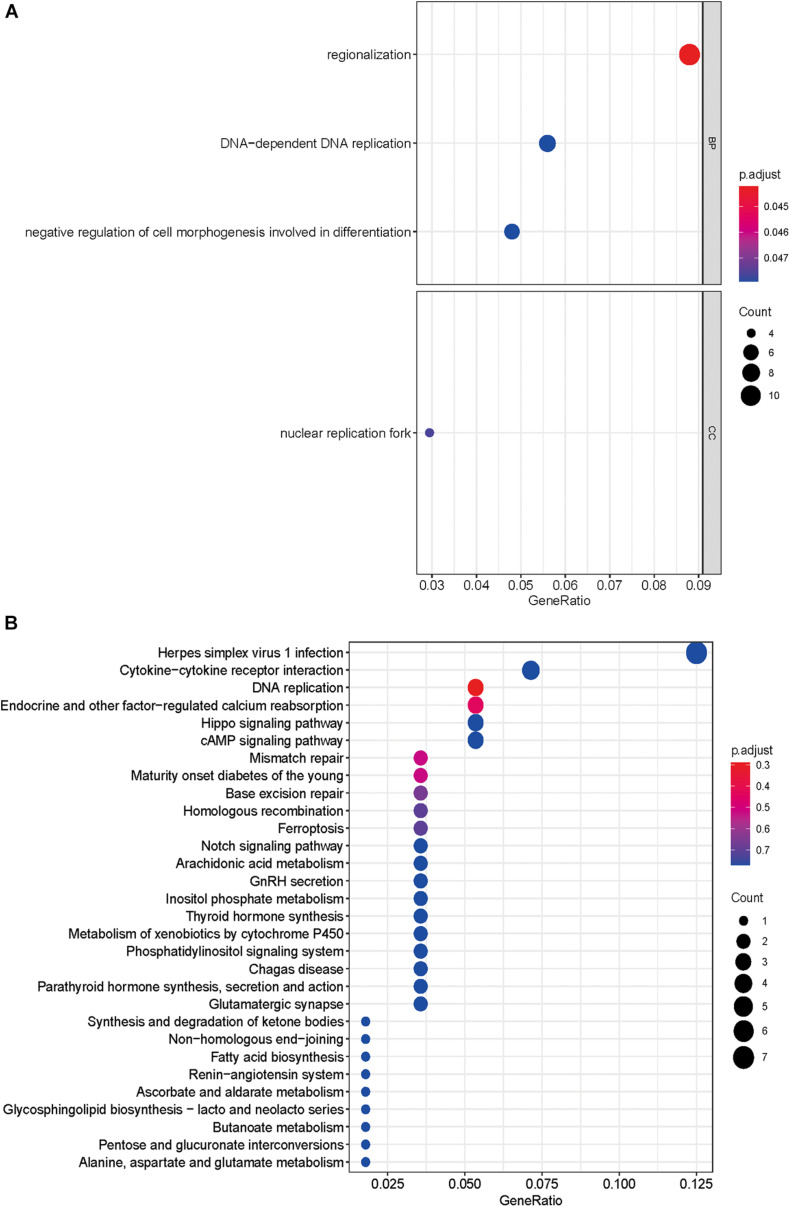
Gene Ontology and KEGG pathway enrichment analyses. **(A)** GO analysis of 143 differentially expressed hepatitis C virus-induced genes. “BP” represents “biological process,” “CC” represents “cellular component,” and “MF” represents “molecular function.” **(B)** Kyoto Encyclopedia of Genes and Genomes analysis of differentially expressed hepatitis C virus-induced genes.

A total of 143 differentially expressed HCVIGs were subjected to GO and KEGG functional annotation. A total of three GO terms of BP and one GO term of CC were statistically significant (*P* < 0.05). The four GO terms included “regionalization,” “negative regulation of cell morphogenesis involved in differentiation,” “DNA-dependent DNA replication,” and “nuclear replication fork.” The results are shown in Figure 3A. In addition, in Figure 3B, a total of 148 KEGG pathways were analyzed; the top three KEGG pathways were DNA replication, endocrine and other factor-regulated calcium reabsorption, and mismatch repair. These results are closely associated with HCV infection.

### PPI and Hub Genes Analysis

**FIGURE 4:**
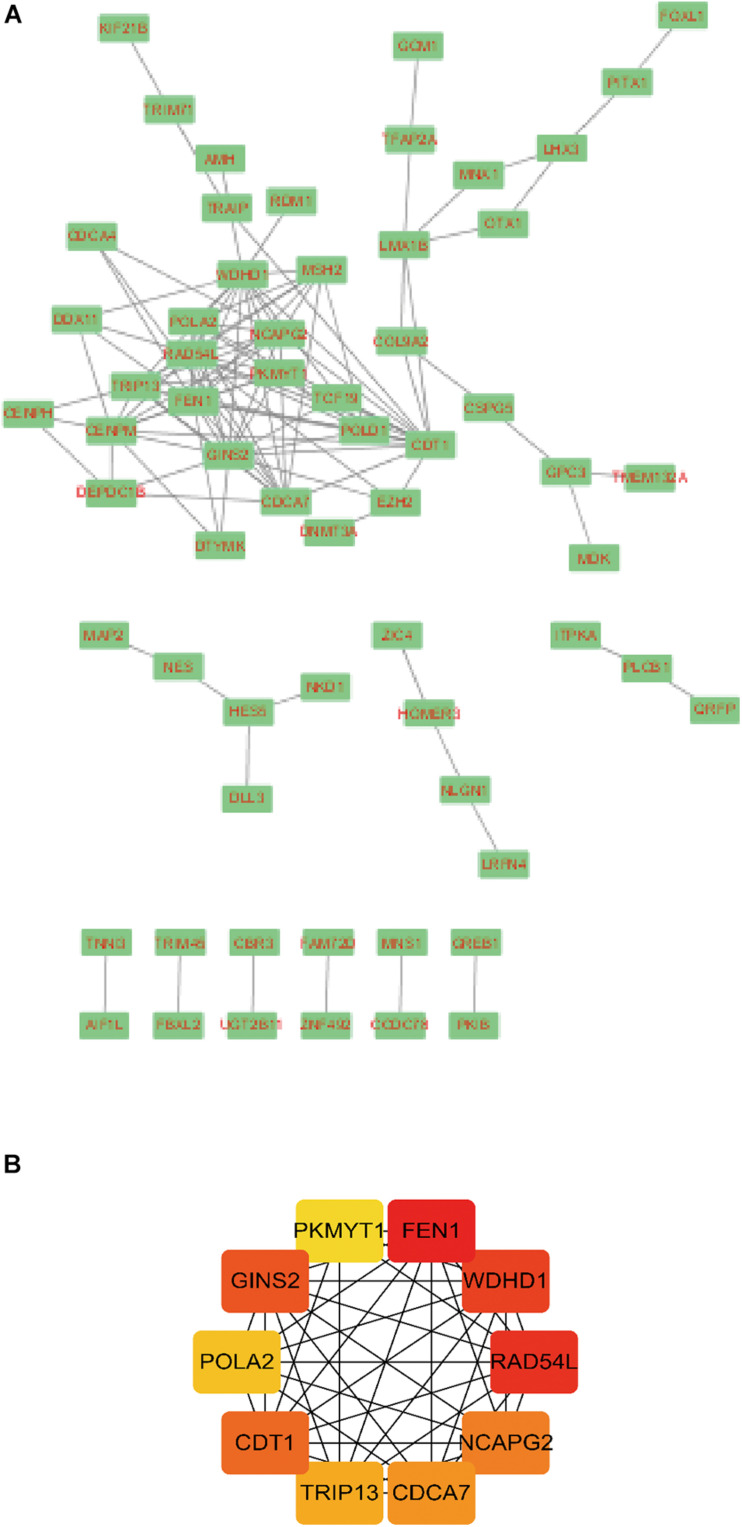
Module analysis of the PPI network. A total of 143 differentially expressed hepatitis C virus-induced genes PPI network in **(A)** Cytoscape and **(B)** CytoHubba application. Circles represent genes, lines represent the interaction of proteins between genes, and the results within the circle represent the structure of proteins. Line color represents the evidence of interaction between proteins.

A total of 141 nodes and 120 edges were downloaded from the STRING database, and then Cytoscape software was used to visualize the regulatory network of the genes (Figure 4A). The CytoHubba application identified the top 10 hub genes (Figure 4B). These genes included *FEN1*, *WDHD1*, *RAD54L*, *GINS2*, *CDT1*, *NCAPG2*, *CDCA7*, *TRIP13*, *POLA2*, and *PKMYT1*.

### Nine Prognostic HCVIGs Risk Score Model Construction

**FIGURE 5:**
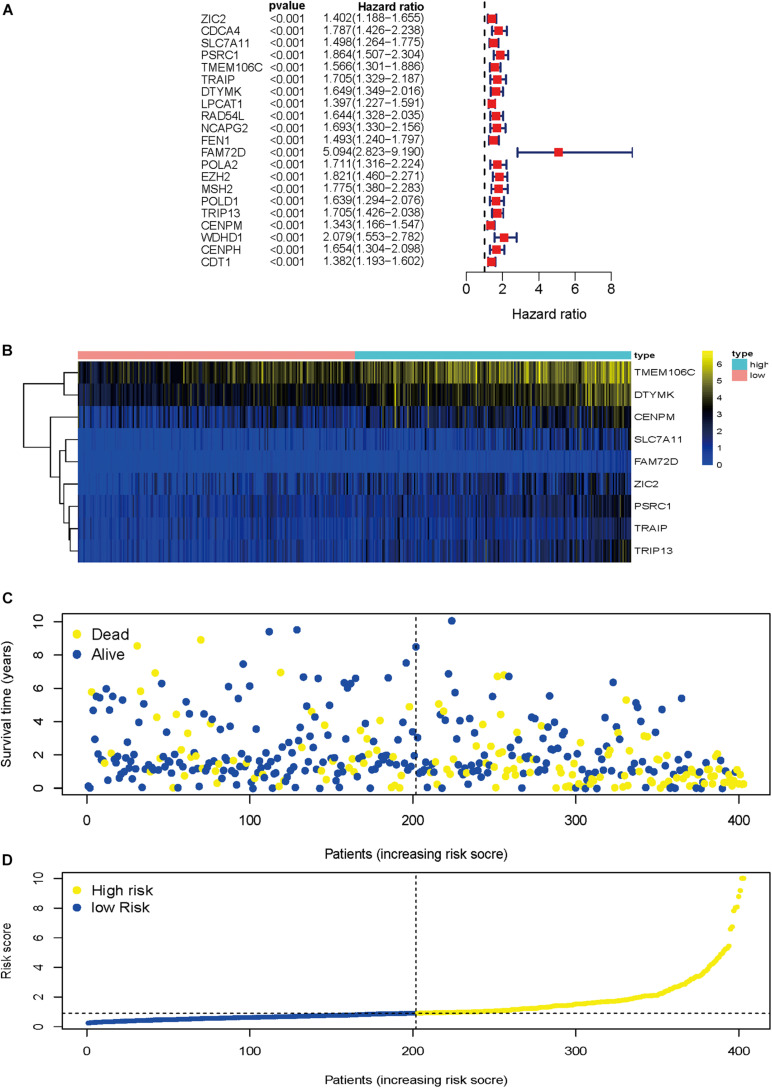
Regression analysis and characteristics of prognostic gene signatures. **(A)** Forest map of 21 prognostic hepatitis C virus-induced genes by univariate Cox regression. **(B)** Heat map of hepatitis C virus-induced gene expression in the prognostic signature of hepatic cancer. **(C)** Distribution of follow-up times in the training sample. **(D)** Distributions of risk scores in all samples.

To identify HCVIGs associated with hepatic cancer, we screened out 21 prognostic HCVIGs using univariate analysis (Figure 5A). Finally, nine prognostic genes (*ZIC2*, *SLC7A11*, *PSRC1*, *TMEM106C*, *TRAIP*, *DTYMK*, *FAM72D*, *TRIP13*, and *CENPM*) of hepatic cancer were identified. The coefficients of the nine prognostic genes are shown in Table 1. Nine prognostic genes were determined to construct the risk model. The study formula for the risk score was as follows (Wang et al., 2019): Risk score = (0.169963967 × expression value of *ZIC2*) + (0.335235013 × expression value of *SLC7A11*) + (0.35212068 × expression value of *PSRC1*) + (0.3061227 × expression value of *TMEM106C*) + (−0.537475693 × expression value of *TRAIP*) + (0.461239921 × expression value of *DTYMK*) + (1.079744869 × expression value of *FAM72D*) + (0.3435407 × expression value of *TRIP13*) + (−0.489318594 × expression value of *CENPM*).

**TABLE 1:** Genes included in prognostic hepatitis C virus-induced genes signature.

Id	Coef	HR	HR.95L	HR.95H	*p*-value
ZIC2	0.169964	1.185262	0.982667	1.429627	0.075546
SLC7A11	0.335235	1.398269	1.148343	1.702589	0.000848
PSRC1	0.352121	1.42208	0.986723	2.049524	0.058988
TMEM106C	0.306123	1.358149	1.064037	1.733556	0.013954
TRAIP	−0.53748	0.584221	0.335822	1.016356	0.057101
DTYMK	0.46124	1.586039	1.053333	2.388154	0.02719
FAM72D	1.079745	2.943928	1.194113	7.257866	0.019012
TRIP13	0.343541	1.409931	1.038792	1.913671	0.027514
CENPM	−0.48932	0.613044	0.429589	0.874844	0.006998

Heat map was drawn to investigate genes expression profiles in the high-risk and low-risk HCC groups. Patients were divided into the high-risk and low-risk groups based on the median risk score. The risk score distribution, follow-up time, and gene heat map are shown in Figures 5B–D.

### Nine Prognostic HCVIGs Predict a Prognosis in Hepatic Cancer

**FIGURE 6:**
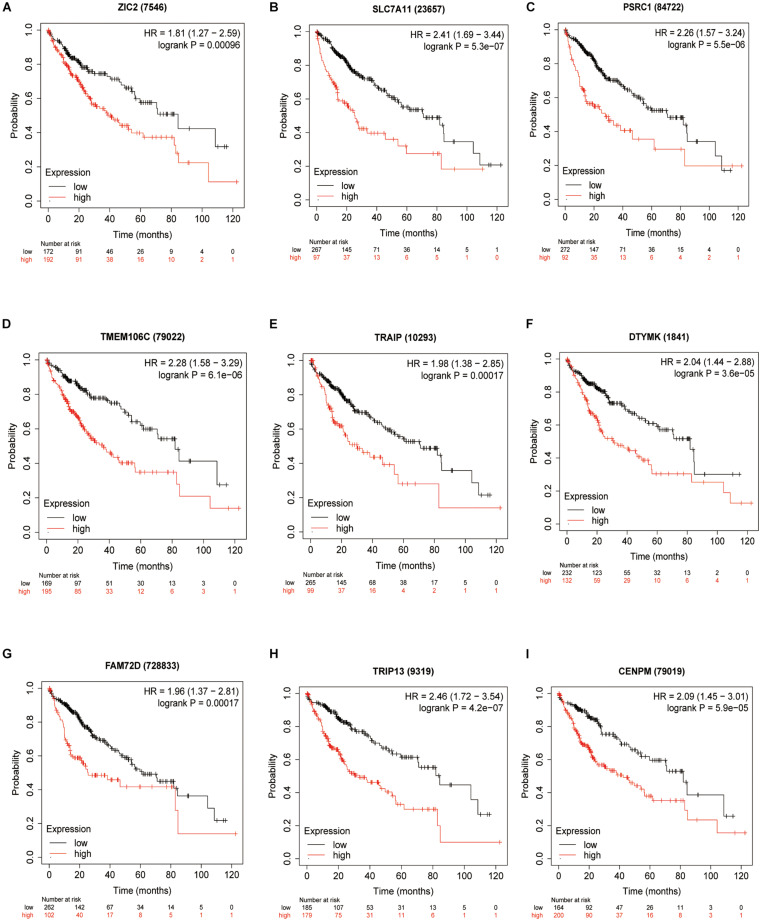
KM survival analysis of hepatitis C virus-induced gene signatures. Overall survival analysis for nine hepatitis C virus-induced genes in the signatures **(A)** ZIC2, **(B)** SLC7A11, **(C)** PSRC1, **(D)** TMEM106C, **(E)** TRAIP, **(F)** DTYMK, **(G)** FAM72D, **(H)** TRIP13, and **(I)** CENPM.

To validate the potential values of *ZIC2*, *SLC7A11*, *PSRC1*, *TMEM106C*, *TRAIP*, *DTYMK*, *FAM72D*, *TRIP13*, and *CENPM* predicting prognosis, we generated Kaplan–Meier survival curves from the Kaplan–Meier Plotter online database (Nagy et al., 2018) using data for 364 liver cancer patients. Among the nine HCVIGs, high expression of *ZIC2*, *SLC7A11*, *PSRC1*, *TMEM106C*, *TRAIP*, *DTYMK*, *FAM72D*, *TRIP13*, and *CENPM* correlated with a poor prognosis. This result is shown in Figures 6A–I.

### Cox Proportional Hazard Regression Analysis

**FIGURE 7:**
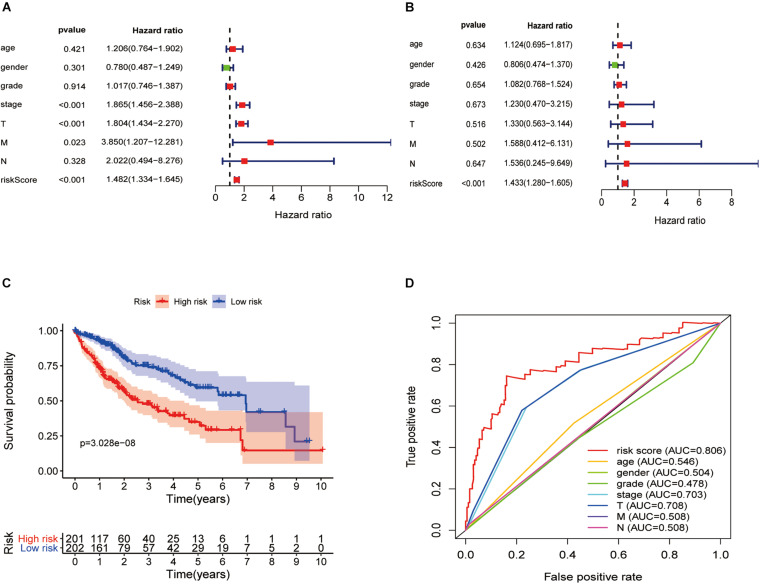
Hepatitis C virus-induced gene signatures are significantly associated with prognosis. Forest plot of associations between risk factors and the survival of hepatic cancer in **(A)** univariate and **(B)** multivariate Cox regression analyses. **(C)** Kaplan–Meier analysis of TCGA hepatic cancer patients grouped according to median risk. High-risk scores are associated with poor survival. **(D)** Multi-index ROC curve of risk score and other indicators.

In the univariate Cox regression analysis, we found that stage [hazard ratio (HR) = 1.865, 95% CI = 1.456–2.388, *P* < 0.001], tumor (T; HR = 1.804, 95% CI = 1.434–2.270, *P* < 0.001), metastasis (M; HR = 3.850, 95% CI = 1.207–12.281, *P* = 0.023), and risk score (HR = 1.482, 95% CI = 1.334–1.645, *P* < 0.001) were significantly correlated with OS. Furthermore, in the multivariate Cox regression analysis, the risk score (HR = 1.433, 95% CI = 1.280–1.605, *P* < 0.001) was found to be an independent risk factor for prognosis in patients with hepatic cancer (Figures 7A,B). Using packages “survival” and “survminer” of the R language, the survival curves revealed that the low-risk group had a better survival outcome (Figure 7C). In this study, the AUC of the ROC curve included the following: risk score (AUC = 0.806), age (AUC = 0.546), sex (AUC = 0.504), grade (AUC = 0.478), stage (AUC = 0.703), T (AUC = 0.708), M (AUC = 0.508), and node (N) (AUC = 0.508). The AUC value of the risk score was higher than that of other clinical parameters (Figure 7D), revealing that this Cox regression model predicted prognosis well in hepatic cancer patients.

### GSEA of the Prognostic Signature in the Low-Risk and High-Risk Groups

**FIGURE 8:**
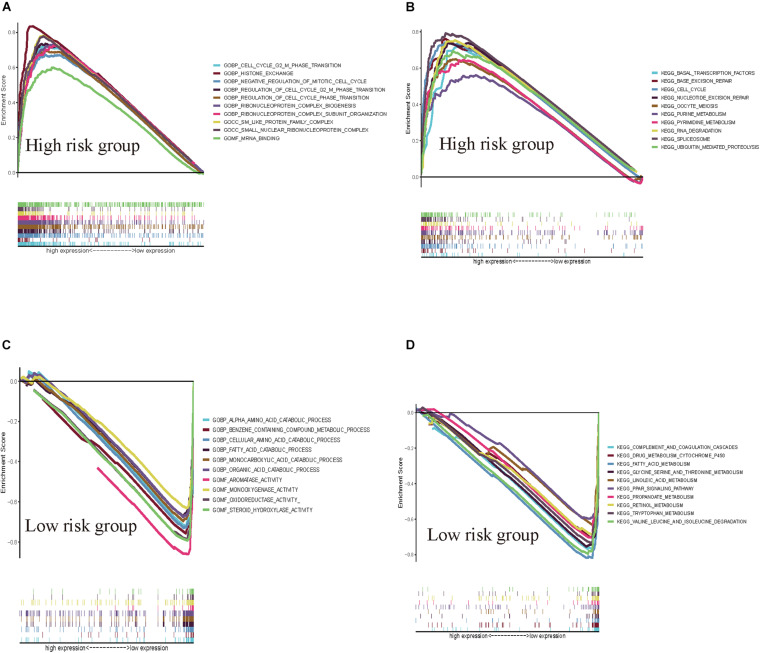
GSEAs for hepatitis C virus-induced gene signatures in hepatic cancer. GSEA shows the GO **(A)** and KEGG pathways **(B)** enriched in the high-risk group of the hepatitis C virus-induced gene signatures in hepatic cancer. GSEA reveals the GO **(C)** and KEGG pathways **(D)** enriched in the low-risk group of the hepatitis C virus-induced gene signatures in hepatic cancer.

GSEAs were performed to compare the low-risk group with the high-risk group of the HCVIG signature. The results showed that these prognostic signature-associated GO and KEGG pathways are significantly enriched in the high-risk (Figures 8A,B) and low-risk groups (Figures 8C,D). The top five KEGG pathways in the high-risk group were RNA degradation, pyrimidine metabolism, ubiquitin mediated proteolysis, cell cycle, and oocyte meiosis. However, the top five KEGG pathways in the low-risk group were complement and coagulation cascades, drug metabolism cytochrome P450, tryptophan metabolism, linoleic acid metabolism, and retinol metabolism.

### External Verification of the Prognostic HCVIG Signature

The HCVIG signature was tested in a hepatic cancer cohort from the ICGC database for validation using the same risk score formula and the same cut-off value.

**FIGURE 9:**
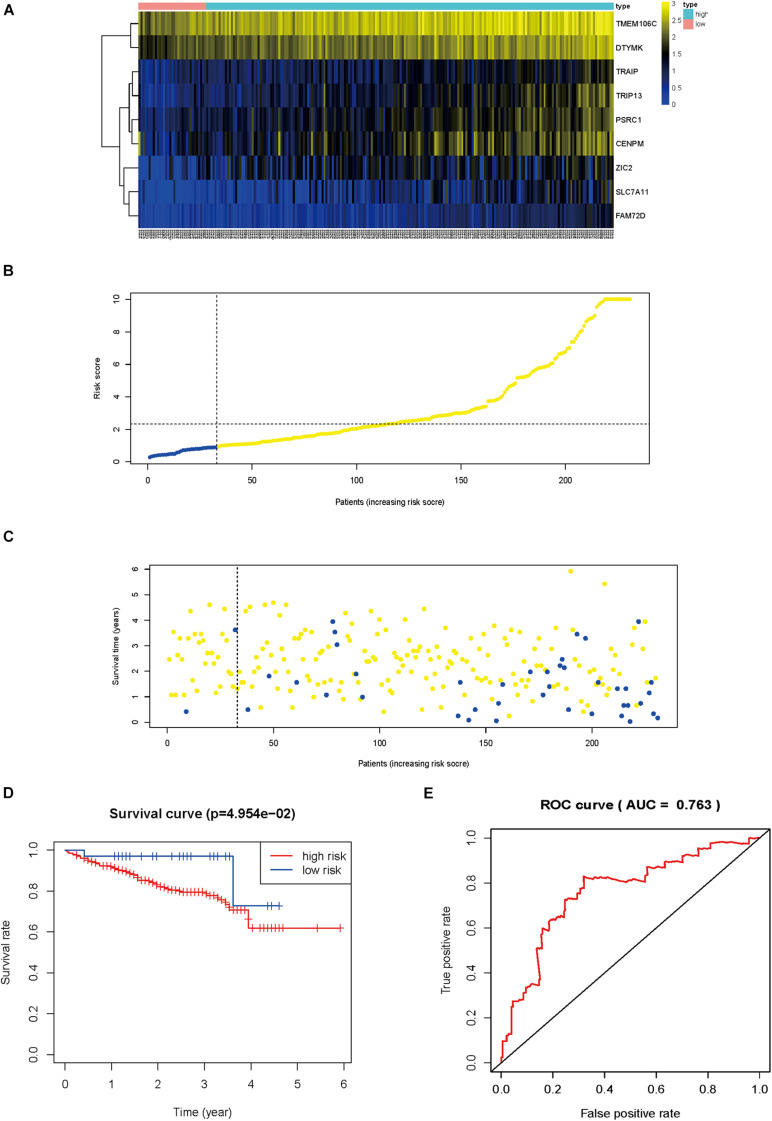
External verification of the prognostic hepatitis C virus-induced gene signature. Heat map of gene expression pattern **(A)**, risk scores distribution **(B)**, survival status of patients **(C)**, Kaplan–Meier plot for OS of patients in different risk groups **(D)**, time-dependent ROC curves for the prognostic value of hepatitis C virus-induced gene signature **(E)**.

**FIGURE 10:**
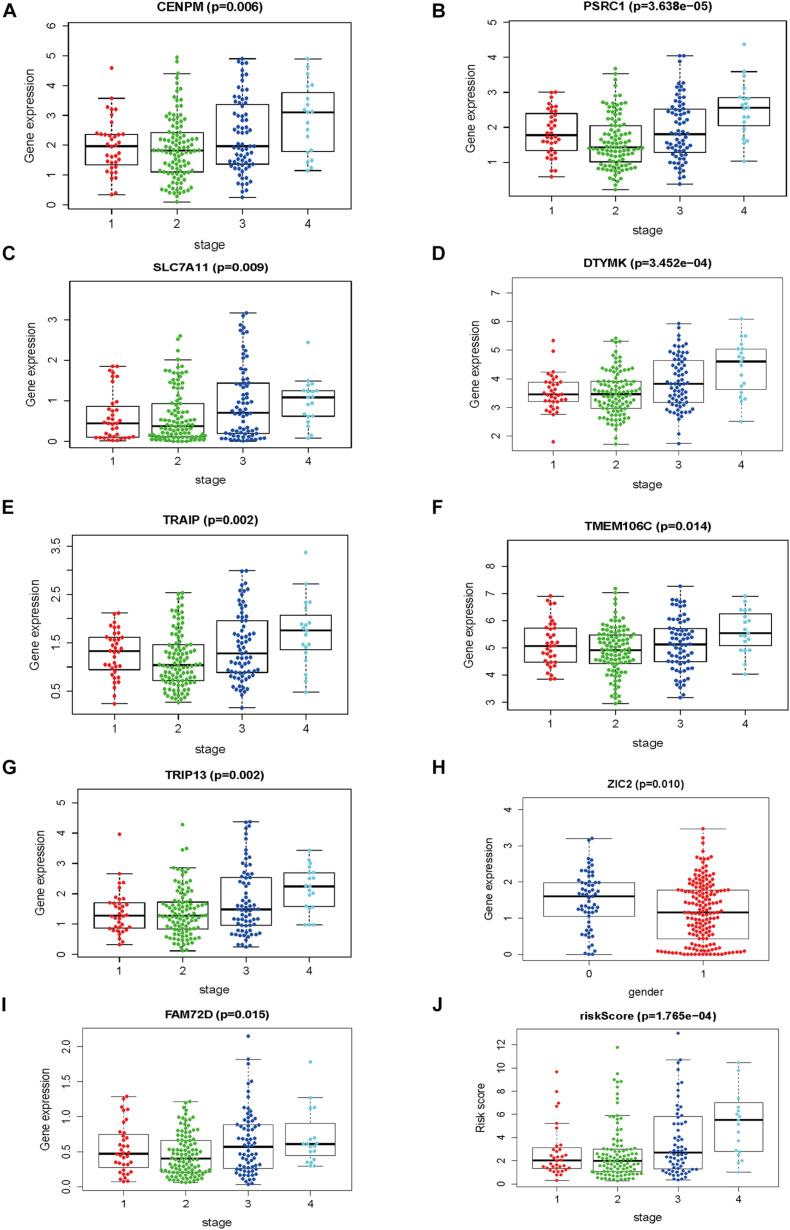
The correlations between risk score and clinicopathological factors. **(A)** CENPM and stage, **(B)** *PSRC1* and stage, **(C)** *SLC7A11* and stage, **(D)** *DTYMK* and stage, **(E)** *TRAIP* and stage, **(F)** *TMEM106C* and stage, **(G)** *TRIP13* and stage, **(H)** *ZIC2* and gender **(I)** *FAM72D* and stage, and **(J)** risk score and stage.

The gene expression pattern, risk distribution, and survival status are shown in Figures 9A–C. The Kaplan–Meier plot showed that patients in the high-risk group had a significantly poorer OS than those in the low-risk group in the validation cohort (Figure 9D). The HCVIG signature had a good accuracy to predict OS in the validation cohort, with an AUC of 0.763 (Figure 9E). Multivariate Cox regression analyses showed that gender, stage, prior malignancy, and risk score were found to be independent risk factors for prognosis in patients with hepatic cancer (Table 1). The relations between risk score and clinicopathological factors are shown in Figure 10.

### Nine Prognostic HCVIGs Are Overexpressed in Hepatic Cancer

**FIGURE 11:**
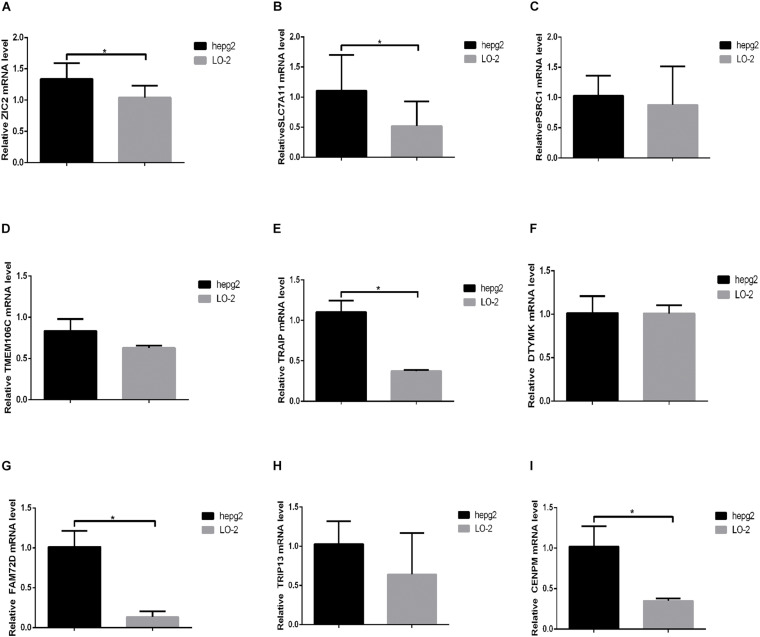
The expression of nine prognostic HCVIGs in hepatic cancer. **(A)** *ZIC2*, **(B)** *SLC7A11*, **(C)** *PSRC1*, **(D)** *TMEM106C*, **(E)** *TRAIP*, **(F)** *DTYMK*, **(G)** *FAM72D*, **(H)** *TRIP13*, and **(I)** *CENPM*.

To explore the expressions of *ZIC2*, *SLC7A11*, *PSRC1*, *TMEM106C*, *TRAIP*, *DTYMK*, *FAM72D*, *TRIP13*, and *CENPM*, these genes were tested in hepatic cancer cell line (HepG2) and normal hepatic cell line (LO-2). The expressions of *ZIC2*, *SLC7A11*, *PSRC1*, *TMEM106C*, *TRAIP*, *DTYMK*, *FAM72D*, *TRIP13*, and *CENPM* are shown in Figure 11.

## Discussion

HCV is an RNA virus that integrates its genetic material into the host genome (Hoshida et al., 2014). Persistent HCV infection is a critical factor in fibrosis/cirrhosis and HCC. HCV can infect and replicate in hepatocytes, impair normal functions of other liver cells, and promote fibrosis/cirrhosis (Sasaki et al., 2017). However, the mechanism of HCV infection in the development of HCC remains unclear.

Many reports have shown that ncRNAs play important roles in many cancers (Hansen et al., 2013; Riquelme et al., 2016; Chen et al., 2020). Aberrant expression of ncRNAs is a key player in HCV-related HCC metastasis, invasion, dissemination, and recurrence (Bandiera et al., 2016; Li et al., 2019; Shehab-Eldeen et al., 2019). However, the functional involvement of HCV-induced genes in liver pathogenesis remains to be explored.

In the present study, we analyzed a total of 143 differentially expressed HCVIGs in TCGA hepatic cancer dataset using the mRNA expression of 1,582 differentially expressed HCVIGs in GSE70781. With univariate and multivariate Cox regressions, a total of 21 prognostic genes were identified. Finally, the signature of nine prognostic HCVIGs was constructed. The Kaplan–Meier Plotter was used in this study to validate the prognostic value of the nine HCVIGs. To the best of our knowledge, this study was the first to establish a nine prognostic HCVIG signature predicting prognosis in hepatic cancer.

The zinc finger of the cerebellum (ZIC) gene family is composed of five members, Zic1–Zic5 (Zhao et al., 2019). The transcription factor zinc family member 2 (*ZIC2*) has been reported to promote proliferation, invasion, and progression in HCC (Zhu et al., 2015; Lu et al., 2017); we found that the overexpression of *ZIC2* had a poor prognosis. The cystine/glutamate antiporter solute carrier family 7 member 11 (*SLC7A11*) is a novel prognostic biomarker for hepatic carcinoma (Zhang et al., 2018). Proline and serine-rich coiled-coil 1 (*PSRC1*), which is encoded by *PSRC1* and is also known as DDA3, has been shown to be involved in HCC (Yang et al., 2011). Transmembrane protein 106C (*TMEM106C*) is differentially expressed and promotes the development of HCC (Luo et al., 2020). TRAF-interacting protein (*TRAIP*) is a master regulator of DNA interstrand crosslink repair (Wu et al., 2019). Guo et al. (2020) showed that *TRAIP* promotes malignant behaviors and is correlated with poor prognosis in liver cancer. Deoxythymidylate kinase (*DTYMK*) is a novel gene associated with mitochondrial DNA depletion syndrome (MDDS) (Lam et al., 2019). Wang et al. (2020) reported that *DTYMK* is a crucial gene associated with immune cell infiltration in HCC. Family with sequence similarity 72-member D (*FAM72D*) is a prognostic gene signature for kidney renal cell carcinoma (Hu et al., 2019); however, the mechanism of *FAM72D* in HCC remains unclear. Thyroid hormone receptor interactor 13 (*TRIP13*) is an AAA+ ATPase that plays an important role in mitotic checkpoint (Zhang et al., 2019). Zhu et al. (2019) showed that elevated TRIP13 drives the AKT/mTOR pathway to induce the progression of HCC by interacting with ACTN4. Zheng et al. (2020) reported that the upregulation of Centromere protein M (*CENPM*) facilitates tumor metastasis *via* the mTOR/p70S6K signaling pathway in pancreatic cancer. *CENPM* plays an important role in HCC (Xiao et al., 2019; Wu and Yang, 2020).

Bioinformatics enrichment analysis showed that 143 differentially expressed HCVIGs were mainly related to DNA replication. DNA replication is a crucial biological process that is tightly regulated to ensure accurate and complete transmission of genetic information to daughter cells (Boyer et al., 2016). DNA replication is fundamental for cellular proliferation and genome stability. Interestingly, many studies have found that DNA replication plays a vital role in liver cancer development (Cervello et al., 2012; Kitao et al., 2018). According to this result, HCVIGs may be associated with host cell DNA replication.

In the present study, we developed and validated the prognostic signature of nine HCVIGs using TCGA, GEO, and ICGC databases. Then, *ZIC2*, *SLC7A11*, *PSRC1*, *TMEM106C*, *TRAIP*, *DTYMK*, *FAM72D*, *TRIP13*, and *CENPM* were identified. Finally, *ZIC2*, *SLC7A11*, *PSRC1*, *TMEM106C*, *TRAIP*, *DTYMK*, *FAM72D*, *TRIP13*, and *CENPM* were upregulated in the hepatic cancer cell line HepG2. In conclusion, this prognostic nine HCVIGs signature could predict prognosis in HCC patients.

This study has two limitations. First, we were unable to collect sufficient hepatic cancer cases to validate the predictive power of this signature. Second, the mechanism of HCVIGs contained in gene signatures requires further study.

## Data Availability Statement

Hepatic cancer data was downloaded from the TCGA database (https://tcga-data.nci.nih.gov/tcga/) under the accession number(s) gdc_download_20201008_115541.090406. Hepatitis C virus-induced genes chip data sets was downloaded from Gene Expression Omnibus database GSE78092 (https://www.ncbi.nlm.nih.gov/gds/?term=GSE70781) under the accession number(s) GSE70781_series_matrix.txt.gz.

## Author Contributions

JW analyzed hepatitis C virus-induced gene expression data of hepatic cancer from TCGA database. BW and XG contributed to the writing and editing of the manuscript. All authors read and approved the final manuscript.

## Conflict of Interest

The authors declare that the research was conducted in the absence of any commercial or financial relationships that could be construed as a potential conflict of interest.

## Footnotes

1 https://www.ncbi.nlm.nih.gov/geo/

2 https://tcga-data.nci.nih.gov/tcga/

3 https://string-db.org/
