# Supplementary material for: Prognostic Value of a Novel Signature With Nine Hepatitis C Virus-Induced Genes in Hepatic Cancer by Mining GEO and TCGA Databases
Source: Front Cell Dev Biol. 2021 Jul 16;9:648279. doi: 10.3389/fcell.2021.648279 (PMC8322788; doi:10.3389/fcell.2021.648279)
Supplement: Supplementary file 1 [file Data_Sheet_1.docx]

The primer sequences

ZIC2

| Forward Primer | GCGCAACTCCACAACCAGTA |  |  |  |
| --- | --- | --- | --- | --- |
| Reverse Primer | TGCCGCATATAGCGGAAAAAG |  |  |  |

SLC7A11

| Forward Primer | TCTCCAAAGGAGGTTACCTGC |  |  |  |
| --- | --- | --- | --- | --- |
| Reverse Primer | AGACTCCCCTCAGTAAAGTGAC |  |  |  |

PSRC1

| Forward Primer | AGGAAGACATAACAGTGTTGGTG |  |  |  |
| --- | --- | --- | --- | --- |
| Reverse Primer | GCATTTGGGTCACTTCGGTG |  |  |  |

TMEM106C

| Forward Primer | TTCACCGGGAGAGATAGCATC |  |  |  |
| --- | --- | --- | --- | --- |
| Reverse Primer | AAGGACTGAATGCGGAAACAG |  |  |  |

TRAIP

| Forward Primer | TCCGACTTCTTCGATCACTCC |  |  |  |
| --- | --- | --- | --- | --- |
| Reverse Primer | AACCACTGAATTAGGCACTGC |  |  |  |

DTYMK

| Forward Primer | CCGGTTCCCGGAAAGATCAAC |  |  |  |
| --- | --- | --- | --- | --- |
| Reverse Primer | TCCCAGCGATTTGCAGAAAAA |  |  |  |

FAM72D

| Forward primer | GGAGTGGGCGAAGAGAAGTC | Plus | 20 | 911 | 930 | 60.11 | 60.00 | 2.00 | 2.00 |
| --- | --- | --- | --- | --- | --- | --- | --- | --- | --- |
| Reverse primer | CCTCCACCCTCTCCAAATCTC |  |  |  |  |  |  |  |  |

TRIP13

| Forward Primer | ACTGTTGCACTTCACATTTTCCA |  |  |  |
| --- | --- | --- | --- | --- |
| Reverse Primer | TCGAGGAGATGGGATTTGACT |  |  |  |

CENPM

| Forward primer | CAGGGCGGTTTGAAAGATCG |  |  |  |  |  |  |  |  |
| --- | --- | --- | --- | --- | --- | --- | --- | --- | --- |
| Reverse primer | GAGGGACTTTGCCAAGTGGA |  |  |  |  |  |  |  |  |
