# Supplementary material for: Prognostic Value of a Novel Signature With Nine Hepatitis C Virus-Induced Genes in Hepatic Cancer by Mining GEO and TCGA Databases
Source: Front Cell Dev Biol. 2021 Jul 16;9:648279. doi: 10.3389/fcell.2021.648279 (PMC8322788; doi:10.3389/fcell.2021.648279)
Supplement: Supplementary file 2 [file Data_Sheet_2.docx]

geneNames GSM813828 GSM813829 GSM813830 GSM813831

A1BG 133.04 29.29 194.21 549.16

A1CF 5899 5794 15.11 10.93

A2BP1 15.4 11.8 106 14.7

A2LD1 456 103 2920 457

A2M 27300 23450 4.573 4.724

A2ML1 4.36 5.84 5.42 6.28

A4GALT 4760 4960 3790 4590

A4GNT 9.4 7.8 11.5 7.12

AAA1 3.015 4.875 4.435 3.645

AAAS 780 1020 1150 2120

AACS 577.5 672 649.5 598

AACSL 2.81 4.9 687 3.42

AADAC 237 716 5.96 4.91

AADACL2 2.86 4.6 4.21 3.48

AADACL3 96.35 122.3 98.245 128.7

AADACL4 3.31 4.18 3.79 3.19

AADAT 390 445 33.1 823

AAGAB 5040 7940 7560 9370

AAK1 1463.1 1831.4 657.3 700

AAMP 3000 4620 3570 5510

AANAT 10.1 16.6 25.2 118

AARS 1340 1770 953 1060

AARS2 2350 1950 2220 4790

AARSD1 2246 2316 1568 2338

AASDH 214 139 107 264

AASDHPPT 2040 2450 3350 4510

AASS 84.7 363 664 578

AATF 4660 6300 13200 8010

AATK 86.9827272727273 8.70181818181818 174.15 265.320909090909

ABAT 2379.305 416.805 61.385 173.815

ABCA1 600.5 807 31.55 14.345

ABCA10 196 187 32.7 148

ABCA11P 385 382 72.2 220

ABCA12 3.75 5.93 36.9 6.97

ABCA13 4.12 4.67 4.28 3.62

ABCA17P 2.885 4.635 28.375 3.53

ABCA2 42.8 51.3 101.1 52.05

ABCA3 200 5.75 1420 385

ABCA4 11 15.4 27.3 9.22

ABCA5 713 454 95.1 383

ABCA6 8.86 56.7 5.14 4.2

ABCA7 488 301 817 249

ABCA8 3.11727272727273 11.5245454545455 4.48090909090909 3.68909090909091

ABCA9 17600 23300 19400 23000

ABCB1 2027.45454545455 585.090909090909 38.7272727272727 284.363636363636

ABCB10 1691 818 667.5 1755.5

ABCB11 3.37 5.14 4.66 3.95

ABCB4 420 871 4.28 63

ABCB5 4.94333333333333 14.555 13.9575 6.77416666666667

ABCB6 8460 11500 5720 2210

ABCB7 1080 1110 840 2460

ABCB8 212.6 627 208 690.25

ABCB9 24.4 47.9 79.3 121

ABCC1 600 352 1100 662

ABCC10 273 160 156 218

ABCC11 37.1 26.9 5.47 10.6

ABCC12 3.61 5.85 5.39 4.42

ABCC13 10.085 5.7 8 4.305

ABCC2 11100 7950 26.8 38.4

ABCC3 1905 773.5 1580 4.915

ABCC4 123 110 228 197

ABCC5 1289.66666666667 1496 1311.33333333333 1462.66666666667

ABCC6 2206.53846153846 624.769230769231 87.7076923076923 249.253846153846

ABCC6P1 888 775 4.09 50.6

ABCC8 24.3 29 16.8 184

ABCC9 3.805 7.395 5.665 4.72

ABCD1 76.6 87.05 80.2 63.25

ABCD2 3.48 12.3 5.22 4.22

ABCD3 327 712.5 311.5 499.5

ABCD4 471.55 632.15 626.1 1480.6

ABCE1 2425 3620 1970 3245

ABCF1 10500 5180 1880 8200

ABCF2 1390 2030 2580 4190

ABCF3 3880 3840 4880 2530

ABCG1 16 14.5 7.37 25.7

ABCG2 64.3 174 24.4 54.8

ABCG4 3.67 5.83 67.3 151

ABCG5 21.06 80.73 15.913 169

ABCG8 23.4 363 5.64 19.6

ABHD1 24.9 37.6 27.4 154

ABHD10 16.2 15.3 4.32 6.81

ABHD11 678.333333333333 338.533333333333 2277 1240

ABHD12 573 1480 1370 616

ABHD12B 3.845 5.02 5.65 5.32

ABHD13 64 32.7 35.9 30.2

ABHD14A 3570 5215 5835 4885

ABHD14B 458 433 901 566

ABHD15 198 213 275 183

ABHD2 1280 396 278 268

ABHD3 2210 1140 1000 800

ABHD4 733 1690 642 421

ABHD5 1000 3680 4560 2530

ABHD6 791 938 2600 1930

ABHD8 5060 4020 3600 1250

ABI1 306 406 466 453

ABI2 205 956 996 1620

ABI3 55.6 43 30.5 40.7

ABI3BP 7.085 7.39 9.93 10.195

ABL1 747.35 741.8 539.1 702

ABL2 352.7475 337.9925 326.475 492.5025

ABLIM1 2523 4738.66666666667 5019 3036.06666666667

ABLIM2 443.233333333333 7.71333333333333 28.8333333333333 35.6666666666667

ABLIM3 504 334.4 9.26 34.84

ABO 26.6 15.9 26.1 22.8

ABP1 12.2 19.4 22.4 16.4

ABR 979.35 570.2 3082 1911

ABRA 3.73 5.72 5.62 4.85

ABT1 449 456 105 758

ABTB1 217 1111 1787 468.5

ABTB2 540 826 1020 881

ACAA1 12500 16800 5460 5400

ACAA2 9520 7740 22500 3780

ACACA 4288.5 4287 5900 5975.5

ACACB 153.2 163.41 78.1 447.95

ACAD10 262.85 314.4 236.35 2163.75

ACAD11 186 352 241 683

ACAD8 110 167 556 203

ACAD9 4653.5 6520 9855 5040

ACADL 26.8 5.53 37.5 295

ACADM 1330 1260 1990 2700

ACADS 64.3 85 69.6 98.1

ACADSB 1114.5 2129 256.4 1932.5

ACADVL 18800 14000 21200 4210

ACAN 41.95 19.13 22.4 33.4

ACAP1 6.67 7.28 10.9 8.6

ACAP2 324 522 629 326

ACAP3 7240 4795 4785 7149

ACAT1 3713.33333333333 3050 5806.66666666667 5313.33333333333

ACAT2 31400 34900 7990 12900

ACBD3 617 478 666 981

ACBD4 301.95 382.3 312 304.75

ACBD5 979 1726.5 796.35 581.6

ACBD6 8800 5680 8500 11400

ACBD7 700 745 632 581

ACCN1 62.8 72.9 76.1 69.8

ACCN2 26.5 332 825 2040

ACCN3 16.6 22.6 16.5 48.3

ACCN4 3120 1560 1390 540

ACCN5 4.02 6.33 5.94 4.86

ACCS 149 93.6 186 236

ACCSL 52 8.92 5.2 4.11

ACD 4210 5640 8750 5910

ACE 27.4054545454545 25.3090909090909 27.4545454545454 27.3018181818182

ACE2 3.24 11.9 16.2 65.6

ACER1 38.5 44.2 38.9 57.7

ACER2 48.25 45 41.45 39.6

ACER3 78.5 55.7 129 89.1

ACHE 111 120 112 151

ACIN1 1330 1410 1170 1740

ACLY 4060 3270 3360 4660

ACMSD 3.54 148 124 4.31

ACN9 2720 4090 7030 4920

ACO1 1710 1610 1470 1850

ACO2 8656 6147 6368 7577

ACOT1 8980 7180 1500 7390

ACOT11 120.75 207.05 86.7 245.35

ACOT12 65.7 108 18 18

ACOT13 21600 16000 4760 8460

ACOT4 932 370 14.4 3.44

ACOT6 3.4 6.76 5.03 4.08

ACOT7 959 1620 5340 2200

ACOT8 887 811 1190 1080

ACOT9 2923.5 4455 5245 3200

ACOX1 8790 3230 2370 4280

ACOX2 644.8 2167 277.6 17.76

ACOX3 1380 596 129 250

ACOXL 3.915 4.81 8.435 99.18

ACP1 2998.5 3573.19666666667 2700.96666666667 4670.6

ACP2 302.5 250.9 149.25 217.4

ACP5 3080 978 9.64 15.6

ACP6 133 341 697 465

ACPL2 292.5 247 213.45 803.5

ACPP 8.305 45.95 9.165 45.7

ACPT 39 44.2 59.4 38.2

ACR 73.65 92.8 75.2 104.15

ACRBP 35.3 31.2 47.6 28.5

ACRC 413 135 455 887

ACRV1 3.4 5.48 5.03 4.05

ACSBG1 29.88 10.295 18.8 18.375

ACSBG2 22 16.1 18.1 11.3

ACSF2 232 625 623 320

ACSF3 629.5 56.85 539 811.5

ACSL1 468.9 502.1 205.9 571.6

ACSL3 2780 3470 810 1380

ACSL4 3870 4950 159 610

ACSL5 200 884 218 184

ACSL6 199 14.43 8.69 102.7

ACSM1 8.513 7.725 6.038 5.087

ACSM2B 55.6 213 37.4 33.1

ACSM3 2.9 65 568 117

ACSM4 3.51 5.61 5.18 4.19

ACSM5 37.435 51.91 27.11 37.95

ACSS1 8.06181818181818 7.8 14.0863636363636 736.636363636364

ACSS2 568 835 334 438

ACSS3 2580 1470 24.4 367

ACTA1 226.4 113 27.93 375.4

ACTA2 21.4 22.8 359 143

ACTB 41854.5454545455 38918.1818181818 42172.7272727273 30472.7272727273

ACTBL2 2690 2030 1910 1320

ACTC1 3.5 5.65 5.22 4.13

ACTG1 70600 66400 91500 76000

ACTG2 24.9 561 17.5 24.9

ACTL6A 7710 6530 10800 4730

ACTL6B 61.1 70.1 83.2 88.5

ACTL7A 22.8 26.6 22.6 24.3

ACTL7B 17.9 13.8 5.79 5.58

ACTL8 41 21.515 218 58.2

ACTL9 26.7 17.9 26.2 19.8

ACTN1 4750 3750 5270 2010

ACTN2 28.1 25.8 17.8 57.5

ACTN3 263 253 249 121

ACTN4 2806 4910 5771 1689

ACTR10 129 182 47.9 334

ACTR1A 1770 2950 2040 2850

ACTR1B 594 1210 1170 1340

ACTR2 6020 3560 2720 3900

ACTR3 9570 8919 17930 7412

ACTR3B 467.333333333333 912 1029 1088.33333333333

ACTR3C 110 180 113 152

ACTR5 129 156 194 307

ACTR6 1230 1810 1830 2600

ACTR8 236 250 218 436

ACTRT1 80.9 5.68 5.24 4.29

ACTRT2 17.6 17.4 17 18.6

ACVR1 1218.5 646 332.5 257.6

ACVR1B 21.5 104 103 87.4

ACVR1C 37.4666666666667 120.1 10.8233333333333 81.3333333333333

ACVR2A 257 228 170 212

ACVR2B 373 748 383 2030

ACVRL1 4.11 6.35 444 62.3

ACY1 2740 6490 4060 1340

ACY3 16.3 279 26.5 14.7

ACYP1 1750 2060 6210 4710

ACYP2 833 270 972 249

ADA 3320 1720 5250 8350

ADAD1 10.9 10.5 7.06 8.17

ADAD2 63.1 124 99.4 123

ADAL 68.7 38.3 5.45 350

ADAM10 912.5 671.5 3750 252

ADAM11 28.85 24.55 77.55 275

ADAM12 25.62 22.43 32.5233333333333 27.96

ADAM15 4225 7530 12970 7076

ADAM17 498 902 592 713

ADAM18 7.32 4.29 3.88 3.25

ADAM19 763.55 7.205 5.275 384.4

ADAM2 3.74 37.3 5.62 4.79

ADAM20 4.38 5.75 5.5 4.56

ADAM21 8.68 11.185 19.82 18.55

ADAM22 197 28.8 238 114

ADAM23 31.5 447.5 4.5 263.5

ADAM28 10.5 40.4 15.9 6.7

ADAM29 8.14 18.5 4.6 4.39

ADAM30 3.08 5.01 4.54 3.71

ADAM32 23.05 21.85 37 12.05

ADAM33 116.833333333333 121.763333333333 107.186666666667 94.2566666666667

ADAM3A 3.38 5.38 5.05 4.3

ADAM5P 3.09 4.98 4.58 3.78

ADAM6 3.43 4.74 4.32 3.53

ADAM7 23.8 60.7 14.7 22.1

ADAM8 511 469 325 629

ADAM9 120 330 83.4 63.5

ADAMDEC1 3.58 5.79 5.34 4.36

ADAMTS1 129.65 561 100.6 1720

ADAMTS10 23.6 16.4 13.5 108

ADAMTS12 2.9 4.64 5.23 3.52

ADAMTS13 122.7 79.1 141.3 509.5

ADAMTS14 2.93 4.63 4.28 15.6

ADAMTS15 5.83 7.98 20.6 13.2

ADAMTS16 3.56 10.4 22 6.36

ADAMTS17 2.55 4.13 11 249

ADAMTS18 2.81 4.53 5.18 10.2

ADAMTS19 452 107 50 285

ADAMTS2 15.38 15.9 14.655 37.05

ADAMTS20 3 4.84 4.45 7.26

ADAMTS3 16 167 22.9 278

ADAMTS4 18.6 6.14 5.82 67.3

ADAMTS5 5.66 6 6.73 6.32

ADAMTS6 7.99666666666667 7.96333333333333 5.68333333333333 13.58

ADAMTS7 21233.8625 22908.4825 13341.1 24639.815

ADAMTS8 3.45 5.63 5.16 12.5

ADAMTS9 376 564 7130 317

ADAMTSL1 14.045 14.3925 13.1775 17.025

ADAMTSL2 139.433333333333 25.3333333333333 24.0666666666667 25.2666666666667

ADAMTSL3 4.245 7.075 53.25 7.5

ADAMTSL4 21.175 76.0833333333333 16.165 22.7566666666667

ADAMTSL5 15281 13944.5 10866.5 12625.5

ADAP1 508 245 756 374

ADAP2 532 36.7 109 1620

ADAR 13900 16900 12200 14500

ADARB1 103.7 268 419 669

ADARB2 7.3 9.515 11.185 14.285

ADAT1 498 665 873 636

ADAT2 13691.5 12250.5 8427 8301.5

ADAT3 2890 2150 1390 6200

ADC 25.935 17.405 43.45 292

ADCK1 90.2 76.1 120 193

ADCK2 86.2 20.6 198 403

ADCK4 72 61.1 154 148

ADCK5 531 365 562 265

ADCY1 99.2 137 27.8 404

ADCY10 29.3 4.6 4.16 7.62

ADCY2 3.675 6.555 7.065 6.605

ADCY3 167 976 781 6270

ADCY4 139.25 82.535 164.55 122.485

ADCY5 4.2 4.63 102 7.8

ADCY6 491 844 2280 411

ADCY7 219 276 1200 85.9

ADCY8 3.84 6.05 5.6 4.53

ADCY9 3850 2820 3180 2210

ADCYAP1 2.77 4.48 181 3.42

ADCYAP1R1 33 47.1 40.3 68.8

ADD1 576 504 171 385

ADD2 5.04 5.31 4.89333333333333 642.666666666667

ADD3 2130 6320 3700 2970

ADH1A 80.8 8.29 131 4.67

ADH1B 2.95 4.69 4.3 3.58

ADH1C 24.8 32.2 1070 11

ADH4 160 15.4 8.38 12

ADH5 2260 3330 1380 2130

ADH6 107.8 77.455 19.58 4.345

ADH7 3.06 4.955 5.115 3.705

ADHFE1 4.885 11.855 81.75 13.35

ADI1 3245 3695 5000 4470

ADIG 17.7 7.65 7.15 3.44

ADIPOQ 6.624 8.585 8.578 9.973

ADIPOR1 1747.5 2322.5 1799.5 1731.5

ADIPOR2 4200 5690 4480 4720

ADK 1368 1554 1649 2353

ADM 3769 7621 341.1 3129

ADM2 396 219.5 126.95 154.5

ADNP 2820 3435 2895 3965

ADNP2 567 639 901 724

ADO 273 461 604 497

ADORA1 3.05 4.85 292 9.85

ADORA2A 63.1 221 10.155 59.85

ADORA2B 548 555 113 2710

ADORA3 5.9 5.95 5.56 4.54

ADPGK 1440 1803.5 1223.5 1503.5

ADPRH 80.1 26.4 119 77.9

ADPRHL1 118 75.3 196 75.3

ADPRHL2 541 899 1080 1300

ADRA1A 335.736666666667 363.096666666667 251.343333333333 396.51

ADRA1B 3.98 4.84 89.6 63.1

ADRA1D 11.2 13.5 14 12

ADRA2A 3.44 5.54 5.14 6.79

ADRA2B 3.11 6.59 4.58 3.73

ADRA2C 3670 1680 65.5 2970

ADRB1 41.4 199 6.17 92.4

ADRB2 5.555 5.54 236.9 339

ADRB3 37.9 47.6 45.8 52.7

ADRBK1 11400 14900 6330 6100

ADRBK2 861 5640 156 3620

ADRM1 2900 5750 3850 3190

ADSL 6220 8020 4400 8560

ADSS 2700 1540 4100 3270

ADSSL1 65.1 58.3 733 198

AEBP1 6.85 550 4.21 4.15

AEBP2 1160 1350 2100 1590

AEN 585.5 703 466.5 1035

AES 13600 5420 15100 2620

AFAP1 168 15.5 175 177

AFAP1L1 9.27 565 2450 541

AFAP1L2 4.96 31.8 153 91.8

AFARP1 275 329 839 532

AFF1 249.5 283.5 266.5 190

AFF2 8.675 20.45 5.06 5.28

AFF3 59.2 16.7 68.7 16.7

AFF4 535.5 543.5 991.5 735

AFG3L1 1399.73333333333 1801.46666666667 2470 3080.7

AFG3L2 590 1460 1810 1040

AFM 958 95.7 4.47 3.7

AFMID 532.1 273.633333333333 503 338.033333333333

AFP 7300 228000 45.1 3.61

AFTPH 2103.63636363636 1412.72727272727 985.181818181818 940.454545454545

AGA 795 1170 168 954

AGAP1 534.409090909091 251.527272727273 387 508.545454545455

AGAP11 239 283 215 420

AGAP2 16.6 14.9 5.57 38.1

AGAP3 5645 5205 5645 14250

AGAP7 707.25 762 529 997.75

AGBL1 44.55 50.75 48.9 47

AGBL2 44 39.4 4.41 7.74

AGBL3 139.6 167.645 184.415 150.055

AGBL4 48.21 43.4 42.69 55.05

AGBL5 129 128 318 609

AGER 58.305 154.745 144 61.225

AGFG1 972 968 717.5 533.5

AGFG2 837.45 2200.4 317.95 186

AGGF1 630 823 1060 1220

AGK 359 651 660 1650

AGL 1800 2200 2460 3830

AGMAT 15050 3302 8.502 310.7

AGPAT1 4720 3440 3560 4160

AGPAT2 17800 27600 25900 15000

AGPAT3 4030 920 298 1690

AGPAT4 15.3 7.23666666666667 5.3 746.666666666667

AGPAT5 534 1050 545 1310

AGPAT6 1150 1490 727 983

AGPAT9 2280 3180 2030 1230

AGPHD1 171.5 303 333.5 1078

AGPS 1837.33333333333 3896.66666666667 893.333333333333 2473.33333333333

AGR2 27.7 31700 13100 30.1

AGR3 3.75 26 19 4.65

AGRN 1910 3170 4970 411

AGRP 47.4 46.3 101 58.8

AGT 80620 12510 5.157 8.653

AGTPBP1 304.1 554 874 1247

AGTR1 651 101 4.51 39.1

AGTR2 2.8 4.55 10.62 20.835

AGTRAP 135 272 925 351

AGXT 575 37.5 21.5 28.3

AGXT2 3.09 90.5 4.54 3.69

AGXT2L1 831 15.6 3.96 333

AGXT2L2 279 238 782 596

AHCTF1 1176 624 693.5 393.15

AHCY 2300 3010 4570 3090

AHCYL1 4852.5 8420 6530 7700

AHCYL2 325 647 1310 1030

AHDC1 336 486.5 283.5 253.5

AHI1 110.5 222 53.65 286

AHNAK 927 4201 10646.6666666667 906

AHNAK2 6.105 5.365 561 19

AHR 9325 4460 1488.5 338.5

AHRR 246 5.73 75.1 326

AHSA1 5693 5825 5672 8844

AHSA2 3230 1350 3190 2480

AHSG 165000 11000 41.1 34

AHSP 10.1 12.7 15.6 8.28

AICDA 3.93 6.2 5.67 4.61

AIDA 328.033333333333 283.766666666667 554.4 289.266666666667

AIF1 46.6 22.875 23.22 22.8

AIF1L 250.4 125.866666666667 1424.66666666667 1483

AIFM1 25100 19300 9720 28300

AIFM2 1497.25 1480.8 249.75 279.935

AIFM3 72.5 11.5 4.24 3.51

AIG1 2343.33333333333 4170 1503.33333333333 2450

AIM1 119 203 5.78 29.7

AIM1L 1861.5 1233.5 264.9 96.8

AIM2 3.28 54.6 4.88 3.94

AIMP1 7332 8613 6564 7813

AIMP2 19200 9180 15400 13800

AIP 7390 4340 1890 4510

AIPL1 3.5 5.7 5.24 12.1

AIRE 43.4 37.2 38.4 44.1

AJAP1 3.47 5.015 717 3.755

AK1 745 976 1456 1427.5

AK2 5655.5 5987.5 13136.25 18075

AK3 9490 1579.5 1531.5 2556

AK3L1 7281.5 3848.5 2523.5 5159

AK5 15.16 13.01 106.225 47.555

AK7 4.897 23.19 45.33 13.535

AKAP1 9879 6478.5 5736.5 9708

AKAP10 183 491 341 259

AKAP11 526 981 1035 1646.5

AKAP12 334.833333333333 323.5 452.583333333333 691.5

AKAP13 2500 3170 1050 2130

AKAP14 6.525 7.49 5.18 6.895

AKAP2 77.9 172 12.3 58.8

AKAP3 4.26 6.4 6.09 5.15

AKAP4 7.86545454545455 7.76909090909091 7.47636363636364 7.70909090909091

AKAP5 17.0427272727273 16.4654545454545 9.47181818181818 45.5363636363636

AKAP6 2.95 4.79 4.35 3.6

AKAP7 354 498 85.7 1000

AKAP8 4360 2580 2660 4530

AKAP8L 1670 1010 1450 1870

AKAP9 1457.5 2275 1420 2115

AKD1 24.35 18.95 14.93 23.305

AKIRIN1 3630 4526.66666666667 3233.33333333333 4653.33333333333

AKIRIN2 861 571 696 1150

AKNA 4.15 33.66 12.31 5.34

AKNAD1 20.775 19.38 16.615 16.05

AKR1A1 980 2120 1490 1440

AKR1B1 1110 3990 18500 11400

AKR1B10 3030 10335 49.7 10.36

AKR1B15 1470 5180 52.1 10.3

AKR1C1 460 50950 5005 8.435

AKR1C3 502 37200 13200 16.7

AKR1C4 75.65 2934.5 338.25 7.85

AKR1CL1 3.6 4.69 4.28 3.55

AKR1D1 4.1 4.66 4.3 3.56

AKR1E2 3.18 5.17 7.7 5.62

AKR7A2 4343 5174 11190 7015

AKR7A3 1050 1300 2940 1970

AKR7L 965.8 1130.65 2534.2 1704.95

AKT1 2170 2080 2080 1890

AKT1S1 1840 1300 2120 1840

AKT2 793 1173.33333333333 1142.33333333333 857.666666666667

AKT3 9.725 11.725 30.085 996.5

AKTIP 130 203 521.5 341.5

ALAD 370 326 452 377

ALAS1 2210 2990 2200 2140

ALAS2 67.9 44.6 130 51.8

ALB 442000 241000 5.58 4.63

ALCAM 5710 2050 4680 1430

ALDH16A1 1080 713 1730 723

ALDH18A1 2000 3040 3030 4050

ALDH1A1 55705 18865 2303.5 34.8

ALDH1A2 3.64 5.81 5.47 255

ALDH1A3 5.8 10.6 4.24 258

ALDH1B1 1500 681 1900 2200

ALDH1L1 8.05 6.9 10.1 11.1

ALDH1L2 27.2 24.6 27.1 233

ALDH2 3550 4570 6120 2560

ALDH3A1 63.3 59.4 10090 74.35

ALDH3A2 14800 8175 10550 3060

ALDH3B1 86.1333333333333 135 132.133333333333 77.8666666666667

ALDH3B2 27.5 19.7 33.4 165

ALDH4A1 1935 1101.5 1936 516

ALDH5A1 233 492 6.02 788

ALDH6A1 641 938 1430 1070

ALDH7A1 1621.96666666667 1479.33333333333 2525 1741

ALDH8A1 8.05 4.81 4.36 42.8

ALDH9A1 1190 1520 2850 2960

ALDOA 27400 26600 46300 30600

ALDOAP2 13.7 28.6 14.1 23.7

ALDOB 25.8 13.6 11.785 14.775

ALDOC 61.1 478 624 213

ALG1 1178 1250.75 1487.25 1890

ALG10 306 457 428 617

ALG10B 285 358 544 799

ALG11 1240 4120 2550 2810

ALG12 268 370 306 339

ALG13 1081.7 2354 885.6 1869

ALG14 2800 1980 876 3360

ALG1L 604 904 1020 1800

ALG1L2 479.5 573 778.5 1162

ALG2 947 1445 855.5 1079

ALG3 8870 9430 8850 6210

ALG5 9330 8670 11900 15700

ALG6 1030 2420 1730 6510

ALG8 16500 20800 15700 22900

ALG9 229 424 189 275

ALK 3.4 5.46 5.05 4.11

ALKBH1 285 296 297 637

ALKBH2 2130 2050 8990 8940

ALKBH3 603.5 260 426.5 869

ALKBH4 427 1260 945 1720

ALKBH5 1909.5 2120 2045 2540

ALKBH6 300 722 654 415

ALKBH7 3520 1710 4410 2960

ALKBH8 199.2 236.55 209.1 610.95

ALLC 3.536 5.537 4.971 4.078

ALMS1 337.45 855.8 414.75 891.05

ALMS1P 71.7 212 88.2 224

ALOX12 3.56 11.8 5.46 4.19

ALOX12B 17.7 18 18.6 16.3

ALOX12P2 3.54 14.9 7.16 3.84

ALOX15 25.505 20.38 25.195 28.915

ALOX15B 57.1 60.6 43.5 66.4

ALOX5 4.15272727272727 5.19818181818182 4.79363636363636 46.7509090909091

ALOX5AP 2.56 4.14 3.76 3.16

ALOXE3 41.4 39.6 52 53

ALPI 28.4 50.6 33.5 34.5

ALPK1 122 116 170 312

ALPK2 10.5 490 8.61 4.48

ALPK3 4570 78.6 57.4 154

ALPL 14 9.09 29.6 29.7

ALPP 179 203 178 189

ALPPL2 1250 1130 1130 1030

ALS2 164.333333333333 338 221 184.2

ALS2CL 85.2 113 179 80.6

ALS2CR10 72.1 20.6 14 12.5

ALS2CR11 3.96 4.65 119 3.74

ALS2CR12 5.62 5.17 4.97 13.3

ALS2CR4 1054 1308.5 1168 2390

ALS2CR8 24 29.3 86.6 21

ALX1 104 128 5.08 393

ALX3 17.1 21.5 136 34.9

ALX4 6.655 17.15 13.68 195.6

AMAC1 3.27 5.33 4.86 3.88

AMAC1L2 26.4 20.7 27.9 34.8

AMACR 471.85 284.6 485.75 217.7

AMBN 17.7 27.7 18.3 856

AMBP 97700 58900 271 4.19

AMBRA1 1202.3 1273.7 766.15 1945

AMD1 15310 11355 9695 18425

AMDHD1 1030 436 45 19

AMDHD2 967 563 1180 672

AMELX 16.1 5.66 10 12.7

AMELY 5.04 5.95 7.78 6.33

AMFR 722 1088.5 675 1056

AMH 62.6 84.7 138 209

AMHR2 2.59 14.8 7.13 115

AMICA1 1786.81 637.935 9.22 5.145

AMIGO1 267 251 151 484

AMIGO2 146 3470 14.4 95.2

AMIGO3 202.95 214.95 190.8 169.95

AMMECR1 1197 2080 1278 2025

AMMECR1L 3540 3530 4680 4620

AMN 1320 1180 651 986

AMN1 55.5 107 4.95 108

AMOT 480.5 13.56 9.62 5644.5

AMOTL1 333.5 351.45 194.245 495

AMOTL2 6380 15200 9610 2090

AMPD1 3.81 5.965 5.61 7.205

AMPD2 564 1120 775 1190

AMPD3 83.8 139 5.53 29.7

AMPH 19.6 23.3 21.5 248

AMT 1216 2194.5 31.6 30.85

AMTN 3.69 4.16 3.78 3.17

AMY1C 84.1 24.3 46.35 326

AMZ1 8954 7902.575 6552.355 6951.92

AMZ2 2510 5335 5400 12800

ANAPC1 1116.86 1258.7 1712.48 1809.26

ANAPC10 1230 2290 1210 2110

ANAPC11 95500 98100 113000 101000

ANAPC13 1910 2990 2720 2780

ANAPC2 117 94.1 113 172

ANAPC4 6170 7130 3350 4520

ANAPC5 15800 17500 28200 34400

ANAPC7 852 1680 1020 1890

ANG 959 1420 106 306

ANGEL1 243 266 271 544

ANGEL2 523 768.5 370.5 1475

ANGPT1 5.64 1150 5.31 114

ANGPT2 3.2 6.6 4.77 3.79

ANGPT4 24.5 22.2 31.2 31.9

ANGPTL1 6.17 5.545 6.245 6.935

ANGPTL2 18.5 563 8.58 6.49

ANGPTL3 1730 2160 4.15 3.45

ANGPTL4 66.6 416 794 9.82

ANGPTL5 3.43 5.51 5.1 4.27

ANGPTL6 35.6 47.5 39.1 58.8

ANGPTL7 3.215 5.14 4.732 6.615

ANK1 7.915 27.215 2942 41.45

ANK2 76.45 100.05 37.665 56.1

ANK3 723.566666666667 343.4 258.906666666667 519.666666666667

ANKAR 52.7 80.7 23.2 57.9

ANKDD1A 3.705 7.16 12.41 20.09

ANKDD1B 2.75 4.47 4.73 5.89

ANKFN1 11.4 5.29 5.12 7.69

ANKFY1 202.9 140.05 230.85 249.1

ANKH 75.4 77.65 16.08 133.5

ANKHD1 1200 2200 6310 2700

ANKHD1-EIF4EBP3 786 499 877 801

ANKIB1 129 236 334 200

ANKK1 3.85 4.93 4.91 10.8

ANKLE1 7.95 40.8 5.54 10.3

ANKLE2 368.181818181818 595.090909090909 719.272727272727 1109.72727272727

ANKMY1 488.5 538 428.5 319.5

ANKMY2 472 458 493 324

ANKRA2 191 328 212 292

ANKRD1 2610 10600 496 179

ANKRD10 826.5 269.5 355.5 433.5

ANKRD11 2679.8 3566.575 4812.125 4495.05

ANKRD12 413.2 1160.16666666667 420.233333333333 283.233333333333

ANKRD13A 872 763.5 727 1181.5

ANKRD13B 145.15 151.95 426 228

ANKRD13C 50.4 161 167.35 211.5

ANKRD13D 5670 7750 2780 1930

ANKRD16 1900 940 4380 3090

ANKRD17 1980.5 831.65 612.55 1286

ANKRD18A 45 26.1 82.3 300

ANKRD19 4.38 7.87 8.345 1170

ANKRD2 11.6 11.2 88.2 26

ANKRD20A1 71.2 38.5 200 213

ANKRD20A2 98.7725 129.4425 142.0925 380.475

ANKRD20A5 624 479 552 915

ANKRD20B 48.7 60.6 109 357

ANKRD22 3.64 5.61 5.49 4.67

ANKRD23 68.895 113.25 150.645 122.28

ANKRD24 46.9 46.3 51.4 41.1

ANKRD26 177.945454545455 247.136363636364 180.809090909091 260.154545454545

ANKRD26P1 12.65 12.6 23.5 24.45

ANKRD27 450.8 544.5 1384 579.5

ANKRD28 684 692 480 1510

ANKRD29 472 208 4.14 86.7

ANKRD30A 60.295 5.94 4.16 37.94

ANKRD30B 4.83333333333333 5.26 4.63 3.90666666666667

ANKRD31 8.035 6.8 37.11 23.73

ANKRD32 430.5 1209.5 894.5 1390

ANKRD33 307 258 381 295

ANKRD33B 13 28.3 192 25.1

ANKRD34A 3.69 5.73 25.6 111

ANKRD34B 3.37 5.48 352 103

ANKRD34C 4.11 6.29 5.93 4.9

ANKRD35 10.8 33.9 23.6 6.2

ANKRD36 314.533333333333 756 683.333333333333 995.666666666667

ANKRD36B 270 396.5 844.5 723.5

ANKRD36BL1 356 292 259 279

ANKRD37 148 155 41.4 155

ANKRD39 6460 7840 5840 5760

ANKRD40 2290 3300 3360 4340

ANKRD42 25857.75 21456.75 23347 25934.95

ANKRD43 578.4 300.3 113.45 531.5

ANKRD44 15.72 14.945 33.055 44.53

ANKRD45 9960 10800 11700 13800

ANKRD46 1610 1850 987 2420

ANKRD49 925 924 1140 2450

ANKRD5 313 235 263.5 176.5

ANKRD50 143 158 101 233

ANKRD52 4110 15500 9490 11100

ANKRD53 27.8 26.4 27.9 59.8

ANKRD54 236.090909090909 332.727272727273 352.454545454545 502.636363636364

ANKRD55 3.82 6.03 5.76 4.82

ANKRD56 72.9 14.6 15 4.91

ANKRD57 5050 2470 2740 1000

ANKRD58 48.1 72.4 74.9 191

ANKRD6 47.5 32.1 34.5 150

ANKRD60 8.86 5.9 5.52 5.3

ANKRD62 2.67 4.315 3.915 48.255

ANKRD7 5.88 65.7 3.82 85.9

ANKRD9 3330 3000 4580 5020

ANKS1A 5650 1880 3370 4700

ANKS1B 10.7933333333333 12.99 10.9733333333333 9.06

ANKS3 3090 3700 5550 5120

ANKS4B 370 113 4.65 3.77

ANKS6 1470 2580 7640 5250

ANKZF1 269.8 247.8 234.2 340.1

ANLN 3120 2690 2970 1590

ANO1 3.19 5.18 61.8 3.97

ANO10 827.5 4910 5695 3920

ANO2 3410 21.9 22.8 51.7

ANO3 3.72 201 5.6 4.6

ANO4 3.49 203 5.21 46.8

ANO5 196 409 4.55 809

ANO6 1066.28 3305.4 1795.15 722.55

ANO7 224.8 147.85 102.15 165.5

ANO8 337 151 144 217

ANO9 65.4 34.1 44.5 31.5

ANP32A 859.166666666667 1339.67333333333 903.6 1982.16666666667

ANP32B 8620 4.52 10500 16400

ANP32C 734 1130 684 1340

ANP32D 593 946 679 1380

ANP32E 2393.33333333333 3160 2126.66666666667 3226.66666666667

ANPEP 249 423 20 39.7

ANTXR1 2.865 29.3 264.5 553.5

ANTXR2 660.166666666667 1225.2 277.566666666667 290.833333333333

ANTXRL 8.145 6.855 9.96 10.81

ANUBL1 21.0618181818182 89.7636363636364 59.0272727272727 153

ANXA1 152 7880 47900 559

ANXA10 174 10.9 5.42 3.67

ANXA11 3821.8 5168.9 7631.15 3603.65

ANXA13 20.4 26.7 12.8 12.6

ANXA2 20575 35525 34640 5547

ANXA2P1 6620 11900 13900 1790

ANXA2P3 895 1720 1660 198

ANXA3 4174 5334 3669 901.9

ANXA4 15000 7000 34200 1050

ANXA5 11800 38500 14100 13600

ANXA6 20.3 1120 1180 2720

ANXA7 4220 6250 4835 3670

ANXA8L2 27.7 284 31.7 103

ANXA9 1934 1295 29.21 12.54

AOAH 3.22 5.24 4.79 3.83

AOC2 21.3 13.8 20 28.5

AOC3 82.2 54.2 68.5 189

AOX1 37 848.5 15.715 16.65

AOX2P 3.52 5.54 5.3 4.46

AP1AR 466 601 435 584

AP1B1 21459 23966.5 17929.5 23652

AP1G1 2180 3340 2450 3800

AP1G2 424 374 394 404

AP1M1 3060 1590 2190 3210

AP1M2 932 2250 2060 119

AP1S1 599 1260 1450 716

AP1S2 239 581 271.5 2040

AP1S3 600.333333333333 484 901.666666666667 525.666666666667

AP2A1 299 347 588 338

AP2A2 1003.85 1577 746.25 1327

AP2B1 703 1700 2460 1600

AP2M1 28300 35700 45400 34200

AP2S1 62500 66900 124000 85900

AP3B1 1740 1770 2600 2460

AP3B2 3.44 5.61 8.21 39.3

AP3D1 4950 2430 3050 4120

AP3M1 518 957 435 916

AP3M2 471 1440 567 1300

AP3S1 3897 3815.66666666667 6423.33333333333 5136.66666666667

AP3S2 3290 3950 2940 3530

AP4B1 241 254.6 428.5 561.5

AP4E1 2190 2480 2880 2470

AP4M1 228.5 252 278 151.1

AP4S1 106.5 1065 153.5 177

APAF1 119 119 161 125

APBA1 70.3 45.9 50.925 69.65

APBA2 103 19.5 24.2 119

APBA3 425 208 211 155

APBB1 137 314 178 579

APBB1IP 11.07 48.6 4.02 15.02

APBB2 314 295.5 100.1 211.4

APBB3 84.3 204.7 270.3 359.6

APC 60.6272727272727 54.5636363636364 70.8818181818182 99.2727272727273

APC2 38 22.7 34.4 73.5

APCDD1 2.63 4.27 3.86 189

APCDD1L 2.95 4.7 4.35 3.57

APCS 23.4 24.7 28.9 33.4

APEH 1570 1210 4220 1690

APEX1 1470 1590 902 1700

APEX2 1660 3910 1670 4130

APH1A 1536.18181818182 1238.81818181818 916.454545454545 1735.45454545455

APH1B 100 1960 283 348

API5 720 923 718 1146

APIP 559 1390 950 1560

APITD1 1300 3310 5570 2890

APLF 2.75 4.44 4.03 3.36

APLN 17.455 29.24 22.262 47.3

APLNR 13.8 13.3 20.5 14.7

APLP1 24 44.1 403 59.9

APLP2 12000 26700 31100 21800

APOA1 24027.2727272727 3594.54545454545 16.3736363636364 17.3581818181818

APOA1BP 5905 11005 7750 15300

APOA2 300000 148000 9.79 4.88

APOA4 18.3 55.4 10.3 3.36

APOA5 104 12.9 21.7 14.9

APOB 18280 10740 6.076 5.464

APOB48R 18.8 56.7 14.7 17.9

APOBEC1 3.67 5.95 5.43 4.3

APOBEC2 2.94 4.67 4.31 3.57

APOBEC3A 4.02 6.22 5.75 4.71

APOBEC3B 54.7 113 1820 305

APOBEC3C 4.395 7.339 256.9 365

APOBEC3D 12.6 22.3 11.5 28.8

APOBEC3F 50.385 74.49 204.5 321.5

APOBEC3G 3.37 5.48 5.03 27.1

APOBEC3H 5.73 5.51 13.3 16.6

APOBEC4 2.71 4.4 3.98 3.33

APOC1 35700 21700 3420 4390

APOC2 10500 6100 10.4 8.19

APOC3 3180 1940 5.56 4.53

APOC4 46.4 59.3 38.5 27.1

APOD 14.8 5.15 4.74 3.91

APOE 242000 146000 636 2130

APOF 3.23 5.27 4.78 3.88

APOH 58800 7490 5.5 4.49

APOL1 900 1320 931 1100

APOL2 2270 4020 2020 2350

APOL3 13 5.73 4.45 7.83

APOL4 61.215 105.65 45.525 56.49

APOL5 11.1 7.11 9.46 15.8

APOL6 27.1666666666667 9.54666666666667 5.52333333333333 3.87333333333333

APOLD1 54.95 72.3 312.5 139.5

APOM 4480 3000 699.5 181

APOO 3680 9690 8660 10900

APOOL 1430 6.37 925 1490

APP 14790 6906 17755 4098

APPBP2 505 729 822 1850

APPL1 2550 2080 6360 6670

APPL2 115.6 379.2 291.5 201.6

APRT 29400 21700 39700 58000

APTX 1128.66666666667 1082.33333333333 1461 2331.33333333333

AQP1 40.55 33.55 65.65 133.75

AQP10 52.1 39.3 20.4 31.4

AQP11 340 270 133 93.3

AQP12A 3.11 5.07 4.61 4.29

AQP2 12 14.7 13 15.6

AQP3 469.754545454545 144.672727272727 11.5045454545455 70.8090909090909

AQP4 14.725 15.61 14.8 31.28

AQP5 244 215 237 232

AQP6 4.085 4.625 6.325 11.29

AQP7 58.6 5.42 256 10.5

AQP7P1 115.05 17.25 285.8 37.65

AQP7P2 7.7 5.76 62.8 6.16

AQP7P3 150 5.67 2310 86.5

AQP8 32.2 44.2 54.2 47.2

AQP9 8.881 6.314 9.528 7.692

AQR 833 1047.5 555.5 870

AR 5.989 5.378 4.977 70.15

ARAF 473 544 343 848

ARAF2P 27000 28800 23000 27000

ARAP1 227.6 277.65 463.2 370.75

ARAP2 499 7.02 430 210

ARAP3 2.78 281 4.09 256

ARC 13.4 22.7 44.6 104

ARCN1 1660 2075 3770 2640

ARD1A 1610 2450 4660 7030

ARD1B 556 910 1360 2450

AREG 12230 3285 5.14 17.435

ARF1 2310 1540 2580 3760

ARF3 4350 9640 8750 8090

ARF4 13900 14100 12600 12500

ARF5 16300 24400 32400 31200

ARF6 956 1210 1100 1190

ARFGAP1 2290 3930 1730 3030

ARFGAP2 10200 8930 5530 8560

ARFGAP3 3370 4820 3520 3600

ARFGEF1 547.7 777.25 1775.5 506.85

ARFGEF2 1060 1490 841 737

ARFIP1 1464.5 1994.5 1168 1852.5

ARFIP2 1560 1470 1340 2730

ARFRP1 1196.93333333333 1724.33333333333 2022.33333333333 1339

ARG1 187.5 53.3 7.38 3.87

ARG2 658 1010 261 992

ARGFX 38.3 66.6 56.2 84.9

ARGFXP2 14.2 11 15.4 16.8

ARGLU1 3900 1800 3090 2660

ARHGAP1 18100 12400 14500 5550

ARHGAP10 261 57.8 336 405

ARHGAP11A 165 181 485 178

ARHGAP11B 621 685 734 469

ARHGAP12 4330 3470 1440 1410

ARHGAP15 2.77 4.47 4.06 3.38

ARHGAP17 797.7 529.75 438.25 1387.9

ARHGAP18 2090 1790 848 302

ARHGAP19 145.6 462.333333333333 284 354

ARHGAP20 3.23 5.29 51.2 90.6

ARHGAP21 530 755 601 643

ARHGAP22 494 18.6 1160 2520

ARHGAP23 28.2333333333333 15.1466666666667 609.333333333333 34.1

ARHGAP24 10 32.6 10.8 3.2

ARHGAP25 5.86 270 5.32 53.4

ARHGAP26 276 59.1 294 316

ARHGAP27 1920 1130 18100 2280

ARHGAP28 3.675 5.07 4.74 77.35

ARHGAP29 27.2 50.8 281 32.7

ARHGAP30 103.965 51.935 79.65 33.625

ARHGAP4 9730 6663.3 16450 5751

ARHGAP5 301.25 6267.5 288.15 544.75

ARHGAP6 4.175 5.3 88.4 12.18

ARHGAP9 24.7 29.7 6.72 10.9

ARHGDIA 2090 2730 1930 4090

ARHGDIB 66.7 144 79.8 48.4

ARHGDIG 17.7 15.1 29.5 448

ARHGEF1 91.67 85.585 138.895 59.585

ARHGEF10 1960 6.62 5090 3100

ARHGEF10L 5880 4725 4065 479

ARHGEF11 256 414 200 254

ARHGEF12 53.3 37.2 86.5 86.8

ARHGEF15 1260 1140 907 874

ARHGEF16 2710 477 4710 442

ARHGEF17 245 1630 2020 385

ARHGEF18 4859.5 2361.75 2542.4 3231

ARHGEF19 160.115 199.75 221.5 67.425

ARHGEF2 291.6 446.5 291.15 222.35

ARHGEF3 197 185 386 320

ARHGEF4 8.54 6.73 20.86 415.79

ARHGEF5 1150 713 2090 3.42

ARHGEF5L 347 61.1 587 17.7

ARHGEF6 3.39 5.46 5.02 56.1

ARHGEF7 560.166666666667 365.366666666667 512.7 476

ARHGEF9 58.75 6.19 197 185

ARID1A 623 506 1000 465

ARID1B 556.5 620 476.5 806.5

ARID2 279 717 439 994

ARID3A 9000 8980 627 1240

ARID3B 5100 8700 1740 9260

ARID3C 25 17.1 23.2 24.4

ARID4A 137.9 77.02 107.15 73.44

ARID4B 793 571 851 1050

ARID5A 1371 768.5 315 416.5

ARID5B 1086.65 1083 265.4 275.2

ARIH1 1020.5 1510.5 422 1155

ARIH2 5795 16600 7315 12750

ARL1 2060 4170 3190 3690

ARL10 21.2 14.6 4.28 187

ARL11 3.34 5.44 4.95 3.95

ARL13A 3.095 4.98 4.535 3.72

ARL13B 77.55 183 339 232.6

ARL14 12 148 269 8.82

ARL15 70.9 42.8 148 193

ARL16 1800 3150 6740 5820

ARL17 641.5 417 823 1770

ARL17P1 167 202 466 952

ARL2 1350 768 2280 1140

ARL2BP 872 1660 1220 3420

ARL3 37.8 179 148 264

ARL4A 1470 1925 687 497.5

ARL4C 5.1 2060 2140 2020

ARL4D 319 482 375 367

ARL5A 853 1290 659 1010

ARL5B 825 430 257 242

ARL5C 67.5 76.7 64.9 86.2

ARL6 73.9 153 287 181

ARL6IP1 4397 2853 5355 1667

ARL6IP4 7990 10055 13860 18675

ARL6IP5 1560 633 2040 3920

ARL6IP6 1600 1810 2460 1420

ARL8A 4760 4220 4910 3530

ARL8B 4112 2926 2204 1957

ARL9 53.3 37.1 26.1 71.7

ARMC1 1510 2510 2580 3480

ARMC10 1623.33333333333 4420 11170 2796.66666666667

ARMC2 5.45 15.2 13.7 29

ARMC3 19.555 4.67 35.29 20.55

ARMC4 2.9 4.65 4.26 344

ARMC5 1845.5 1193 1470 2417

ARMC6 3900 3580 2600 3540

ARMC7 1190 1110 4820 1360

ARMC8 1357 1807.5 1461 1233.5

ARMC9 65.5 373.5 473.5 1041.5

ARMCX1 2.93 1670 4.33 3.61

ARMCX2 3.38 108 4.98 462

ARMCX3 671 1470 192 601

ARMCX4 4.64 68.6 5.33 31

ARMCX5 1090 4120 827 3520

ARMCX6 1348.5 5480 850 2146.5

ARMS2 6.11 6.35 24.2 9.18

ARNT 336 489 265 470

ARNT2 9.45 15.715 80.65 359.2

ARNTL 442 138 433 300

ARNTL2 232 291 551 444

ARPC1A 9140 17000 11900 12500

ARPC1B 2870 3110 7670 1400

ARPC2 22670 22430 31930 19860

ARPC3 28600 34600 44700 34700

ARPC4 897 924 1300 453

ARPC5 19500 18350 27150 14050

ARPC5L 4320 5810 6290 4160

ARPM1 173.8 119.1 181.4 132.1

ARPP-21 3.32 13.95 4.945 4.05

ARPP19 3380 1840 2560 1960

ARR3 8.26 11.1 9.16 12.8

ARRB1 144 228 497 180

ARRB2 2990 2420 1740 3430

ARRDC1 654 446 2270 566

ARRDC2 251 74.7 706 270

ARRDC3 106 337 74.6 34.9

ARRDC4 72.4 4.55 57.6 616

ARRDC5 5.86 5.86 5.44 4.44

ARSA 3130 5270 3230 4620

ARSB 195 83.1 70.4 62.3

ARSD 27.8033333333333 244.366666666667 572 28.8833333333333

ARSE 3.76 804 286 25.1

ARSF 20.7 18.6 17.1 14.2

ARSG 24.2 41.85 29.3 41.2

ARSH 26.3 32.9 29.2 34.1

ARSI 3.07 13.1 4.81 3.71

ARSJ 66.5 134 24.7 12

ARSK 83.95 108.7 119.9 104.3

ART1 2.74 4.46 4.03 6.96

ART3 2.9 5.28 4.27 3.51

ART4 31.19 9.197 4.927 4.392

ART5 14.8 16.05 28.1 600

ARTN 115 87.3 104 102

ARV1 3160 938 3660 7410

ARVCF 656.5 264.75 150.05 118.75

ARX 42.8 38.6 42.5 44.3

AS3MT 121635 129130 141002.175 130049.1

ASAH1 6620 9330 8450 3440

ASAH2 150 202 156 174

ASAM 4.61 5.6 21.9 112

ASAP1 540 564 379 407

ASAP2 203 1350 2230 627

ASAP3 56.9909090909091 417.272727272727 42.5272727272727 203.172727272727

ASB1 1388.5 930 663.5 674.5

ASB10 352 622 2310 361

ASB11 3.662 15.483 4.882 4.004

ASB12 5.9 4.96 13.9 19.3

ASB13 1237.5 382.5 2736.5 2010.5

ASB14 3.78 5.92 7.01 4.88

ASB15 12.5 14.6 11.6 11.3

ASB16 266 203 155 174

ASB17 3.52 5.71 5.26 4.3

ASB18 60.5 59.7 34.9 62.2

ASB2 60.3 34.1 53.2 25.8

ASB3 1190 1200 1800 2440

ASB4 3.39 42.2 5.05 4.14

ASB5 6.01 4.91 4.49 3.71

ASB6 248 209 297 361

ASB7 319 391.5 332 350.5

ASB8 137 180 119 259

ASB9 625 747 1830 36.7

ASCC1 914 1000 1020 1100

ASCC2 409 345 422 615

ASCC3 1180 1110 741 1400

ASCL1 17.1 35.3 27.4 6.66

ASCL2 3.94 5.42 8.43 9.8

ASCL3 15.6 19 32.3 22.5

ASCL4 3.87 6.06 5.83 4.95

ASCL5 512 296 473 428

ASF1A 211 237 99.8 244

ASF1B 920.8 993.6 3499 1443

ASFMR1 14.4 13.2 9.4 12

ASGR1 23270 4338 109.3 102.85

ASGR2 45800 20700 24.7 20.7

ASH1L 360 369 326 483

ASH2L 2210 2730 3130 2400

ASIP 11.6 10.3 15.3 17.9

ASL 3840 3470 2580 766

ASMT 3.09 5 4.54 3.71

ASMTL 80.5 559 2450 680

ASNA1 1600 956 2420 1620

ASNS 25700 10200 4040 51300

ASNSD1 9370 7950 3490 14700

ASPA 3.16 29.2 4.65 3.8

ASPDH 56.9 34.5 27.3 22.7

ASPG 12.3 18.6 18.5 33.1

ASPH 7106.66666666667 7716.66666666667 5940 1051.66666666667

ASPHD1 54.7 560 11100 447

ASPHD2 12.92 15.43 60.1 297

ASPM 12715 8050 6710 6190

ASPN 6.2 6.475 7.85 9.615

ASPRV1 127 244 68.4 406

ASPSCR1 1087.64 422.44 953.88 1083.96

ASRGL1 2630 1930 2210 4930

ASS1 4220 377.5 749.5 1459

ASTE1 195.7 219.3 325.9 323.5

ASTL 24.8 34.2 27.6 27.5

ASTN1 3.085 4.95 4.56 25.95

ASTN2 2199.35 1570.65 1599 782.65

ASXL1 1800 2190 944 3090

ASXL2 987.7 1201.35 1233.35 1631.5

ASXL3 8.5 5.59 5.15 465

ASZ1 3.67 5.93 5.49 4.49

ATAD1 5030 6000 5040 7100

ATAD2 12500 7200 14200 5090

ATAD2B 425 548 386 536

ATAD3A 20700 20500 26700 20900

ATAD3B 11318 17016 13976.6666666667 12817.3333333333

ATAD3C 240.15 278.65 330.1 248.25

ATAD4 195.9 24.3 24 5.776

ATAD5 293 422 395 502

ATCAY 3.51818181818182 5.32181818181818 7.23181818181818 4.61545454545455

ATE1 229 474 380 247

ATF1 2460 2250 2940 2970

ATF2 441 439 358 286

ATF3 1126.25 474.875 65.4975 1661.75

ATF4 88200 105000 42400 135000

ATF5 521 548 1546 279.3

ATF6 1860 1390 2240 2870

ATF6B 1020 498 224 734

ATF7 3.71 5.99 5.46 4.35

ATF7IP 1136.63636363636 1172.90909090909 1375.09090909091 1132.54545454545

ATF7IP2 1010 1150 83.4 610

ATG10 126.95 146.75 218.4 95.85

ATG12 1764.45 1415.3 2616.85 1450.25

ATG16L1 1239 1295 1029 1308

ATG16L2 75.7 567 760 1190

ATG2A 2259 1843 1427.5 2545.5

ATG2B 320 376 470 1250

ATG3 3950 4280 9850 5230

ATG4A 1040 1890 531 1270

ATG4B 1870 2135 997 2060

ATG4C 148.566666666667 330.666666666667 207 743.666666666667

ATG4D 221.7 86.21 296.7 385.5

ATG5 1750.45454545455 1669.54545454545 2187.90909090909 1617.72727272727

ATG7 503 417 617 603

ATG9A 644 578.5 544 703

ATG9B 8.605 12.55 15.05 15.13

ATHL1 144.05 101 171.85 181.6

ATIC 11100 8200 6410 9560

ATL1 3.64 6.37 104 245

ATL2 1697.4 2111.1 1131.06666666667 2263.13333333333

ATL3 961.8 3107 904.8 700.1

ATM 103.509090909091 121.090909090909 169.818181818182 154.454545454545

ATMIN 385.775 517 624.95 450.925

ATN1 424 703 1700 671

ATOH1 2.68 4.34 3.94 3.28

ATOH7 12.3 4.76 12.7 31.4

ATOH8 1020 5.15 40.5 16

ATOX1 7020 7770 10200 8380

ATP10A 2.71 4.4 28.5 3.31

ATP10B 12.1 4.51 4.09 3.4

ATP10D 6.3 72 33.3 22.8

ATP11A 5810 1340 1720 1120

ATP11B 391 232 268 224

ATP11C 305 234 222.5 1035.5

ATP12A 4.41 4.63 4.2 40.6

ATP13A1 992 1340 752 944

ATP13A2 201 206 764 326

ATP13A3 10125 3720 3059 1589.5

ATP13A4 3.23 5.235 4.765 3.86

ATP13A5 3.24 5.27 4.81 3.94

ATP1A1 12600 30000 10900 26400

ATP1A2 3.28 5.285 4.855 3.99

ATP1A3 312 360 263 1060

ATP1A4 750.5 1505 621 1540

ATP1B1 133000 75200 59600 4390

ATP1B2 15.4 42.4 28.1 211

ATP1B3 48100 50800 90400 74400

ATP1B4 3.42 5.55 5.12 4.14

ATP2A1 17.0666666666667 8.34333333333333 18.7033333333333 20.5

ATP2A2 875 1680 988 1204

ATP2A3 10.01 19 32.25 40.85

ATP2B1 428.9 913.6 158.4 285

ATP2B2 57.15 93.95 8.445 12.955

ATP2B3 70.1 65.15 63 67.8

ATP2B4 8.62166666666667 198.883333333333 64.9625 249.816666666667

ATP2C1 1150 1210 2210 1180

ATP2C2 3.84 10.8 5.96 3.8

ATP4A 3.53 5.61 5.27 22.7

ATP4B 3.57 5.72 9.38 4.28

ATP5A1 30200 37100 51300 35600

ATP5B 18100 25700 41100 32500

ATP5C1 5850 5210 9140 8310

ATP5D 11800 4410 9500 7840

ATP5E 83557 88610 80577.5 66616.5

ATP5F1 19800 24700 22900 35900

ATP5G1 8340 16000 21400 22100

ATP5G2 32600 46000 55300 68900

ATP5G3 102000 123000 91100 140000

ATP5H 38900 44300 82300 41600

ATP5I 52000 34700 28500 47300

ATP5J 32700 31100 38700 37500

ATP5J2 18350 36650 30150 31100

ATP5L 76200 83200 154000 115000

ATP5L2 9859.5 11002.915 18002.705 12961.8

ATP5O 56300 63700 101000 105000

ATP5S 526.5 488 847 1295

ATP5SL 11060 14680 10493 10587

ATP6AP1 7530 11900 6420 16900

ATP6AP1L 297 119 444 206

ATP6AP2 2275 3965 2775 5151

ATP6V0A1 11900 9830 13200 17900

ATP6V0A4 17.2 15.1 5.33 12.7

ATP6V0B 10300 11600 8860 15400

ATP6V0C 68900 67100 109000 81400

ATP6V0D1 3540 3700 4810 5410

ATP6V0D2 3.8 5.99 5.52 4.52

ATP6V0E1 8030 10700 14100 9310

ATP6V0E2 229 82.3 398 1110

ATP6V1A 16000 11700 13000 15000

ATP6V1B1 17.6 11.8 35.3 11.9

ATP6V1B2 1130 2740 2260 1490

ATP6V1C1 6885 4500 4470 4595

ATP6V1C2 154 202 205 211

ATP6V1D 2030 1320 2120 2420

ATP6V1E1 1780 3360 1330 1680

ATP6V1E2 134 422 119 526

ATP6V1F 15200 17600 34800 30100

ATP6V1G1 12700 19200 13800 17300

ATP6V1G2 47.5 29.2 44.7 111

ATP6V1G3 3.87 5.9 16.1 4.56

ATP6V1H 634 395 858 868

ATP7A 118.14 98.148 43.808 213.36

ATP7B 1550 1960 1220 2450

ATP8A1 163 135 1010 1160

ATP8A2 9.05333333333333 4.82 45.2433333333333 25.3366666666667

ATP8B1 2690 380 879 21.8

ATP8B2 113 177 11.9 180

ATP8B3 14.64 31.385 551.35 22.7

ATP8B4 3.6 5.74 5.43 4.44

ATP9A 2695.5 2748.5 1061 2002.5

ATP9B 1094.90909090909 850.272727272727 1698.90909090909 225.909090909091

ATPAF1 36.4 129 28.6 282

ATPAF2 901 1160 1020 833

ATPBD4 59.8 35.8 25.25 32.875

ATPGD1 13.8 11.4 32 23.6

ATPIF1 17670 24900 42500 67400

ATR 1169.5 1358.5 1199 1884.5

ATRIP 129.7 315.5 287.5 313

ATRN 1450 1010 292 524

ATRNL1 33.67 34.925 38.7 39.65

ATRX 1152.225 1178.125 741.575 1216.425

ATXN1 122.333333333333 180.533333333333 9.90333333333333 66

ATXN10 4025 2162 2926 2656.5

ATXN1L 583.35 1564.705 1146.4 1068.6

ATXN2 2740 3820 5310 4790

ATXN2L 11555 10866.3333333333 8288.83333333333 10791.3333333333

ATXN3 105 261.85 241.55 767.5

ATXN3L 3.57 5.46 5.125 7.845

ATXN7 65.7 56.4 90.4 79.9

ATXN7L1 34.7 181.5 125.1 163.5

ATXN7L2 607 438 348 443

ATXN7L3 560 586 442 608

ATXN8 275 244 254 170

ATXN8OS 9.54 5.56 30.1 4.1

AUH 1140 1760 1860 1870

AUP1 6950 5420 5560 6610

AURKA 4980 11000 6550 5630

AURKAIP1 15500 12700 18000 11300

AURKAPS1 1720 3020 1780 1620

AURKB 1770 1450 1310 2030

AURKC 17.8 9.16 52.9 18

AUTS2 709.414166666667 1141.30833333333 34.8516666666667 3203.5

AVEN 3944 2353 2076 2123

AVIL 7.27 7.04 15.2 10.9

AVL9 545.636363636364 324.636363636364 302.545454545455 232.181818181818

AVP 213 258 232 262

AVPI1 1125 1315 1985 999

AVPR1A 3.75 128 8.88 4.74

AVPR1B 17.3 4.22 3.84 5.72

AVPR2 8.96 6.9 168 9.31

AWAT1 9.38 92.9 8.87 123

AWAT2 3.72 5.99 5.59 4.56

AXIN1 322 207 636 352

AXIN2 115.8 242.5 30.25 229.5

AXL 152 965 6870 208

AZGP1 325 20.4 17 28

AZI1 5740 3620 9090 7700

AZI2 668.5 1280.5 1223.5 979.5

AZIN1 6690 3340 3250 3870

AZU1 12.7 11.3 13.9 9.85

B2M 23070 11910 6436 8298

B3GALNT1 859 1340 122 419

B3GALNT2 1225 789.5 1084 1463.5

B3GALT1 52.77 16.73 142.5 5.325

B3GALT2 62.1 26.6 4.05 3.38

B3GALT4 295 270 29.1 66.3

B3GALT5 3.384 5.412 5.385 4.093

B3GALT6 3340 3020 2960 2170

B3GALTL 331 638 649 310

B3GAT1 3.83 6.04 7.2 4.61

B3GAT2 73.5 75 10.9 198

B3GAT3 2040 1730 1760 2590

B3GNT1 6160 7930 1320 4580

B3GNT2 342 322 297 193

B3GNT3 190 23.7 9010 157

B3GNT4 10.4 5.8 57.3 111

B3GNT5 1020 341 4.68 945

B3GNT6 2.85 4.63 4.2 3.47

B3GNT7 288.35 339.85 299.6 346.45

B3GNT8 169.5 257.9 99.86 126.7

B3GNT9 86.5 25.9 45 27.5

B3GNTL1 205 266 278 361

B4GALNT1 1300 2440 76.6 672

B4GALNT2 4.93 14.7 3.75 5.02

B4GALNT3 10 7.17 25.875 23.86

B4GALNT4 108 6.42 5.39 20.5

B4GALT1 327 243 513 183

B4GALT2 1550 2060 2640 2850

B4GALT3 6861.81818181818 5340.90909090909 4411.81818181818 7250.90909090909

B4GALT4 731 1170 1040 738

B4GALT5 2410 2080 3390 2450

B4GALT6 7.48 5.37 16.7 112

B4GALT7 302 613 678 561

B9D1 391.5 1535 1865 2075

B9D2 818 1030 3040 1070

BAALC 5.105 12.425 7.25 12.42

BAAT 154 160 4.17 6.29

BACE1 944.333333333333 883.666666666667 2244.33333333333 863.333333333333

BACE2 19.3 12.5 32 2190

BACH1 1015.5 895.45 784 697.35

BACH2 40.8 26.9 8.17 67.25

BAD 1390 829 1980 2050

BAG1 5273.33333333333 3536.66666666667 6426.66666666667 7963.33333333333

BAG2 6720 6180 68 4310

BAG3 882 1120 1140 1180

BAG4 329 718 474 467

BAG5 2500 1950 3550 4140

BAGE 30.885 87.22 42.39 90.15

BAGE4 44.8 130 50.8 110

BAHCC1 150 187 106 307

BAHD1 3618 3155 3274 3505

BAI1 33 44.9 4450 229

BAI2 66.81 295.55 86.25 432.5

BAI3 3.62 5.81 50.3 227

BAIAP2 213.666666666667 393.633333333333 1788.66666666667 455.666666666667

BAIAP2L1 2730.05 2378 2566.7 1280.85

BAIAP2L2 5381.15 1262.35 4414.55 57.95

BAIAP3 42.5 5.85 73.6 11.1

BAK1 1780 1550 2890 2640

BAMBI 26600 45500 9820 10800

BANF1 4380 3420 5330 5830

BANF2 3.2 5.09 4.71 3.86

BANK1 128.4 65.99 10.661 4.18

BANP 7820 5323 4852.5 7340

BAP1 863 527 1060 1760

BARD1 1270 966 1150 1310

BARHL1 10.7 10.2 9.22 15.8

BARHL2 11 6.03 14.7 20.1

BARX1 2.67 19 3.94 489

BARX2 3.74 6.3 5.63 99

BASP1 25.7 28.2 3700 2820

BAT1 542 867 917 1230

BAT2 886 807 777 1200

BAT2D1 4770 3650 2620 4980

BAT2L 1247.66666666667 1901 1151.33333333333 2783

BAT3 47500 19625 25030 34180

BAT4 13000 4930 5390 6350

BAT5 277 276 730 193

BATF 15.1 181 7.92 5.07

BATF2 86 100 360 105

BATF3 204 493 27.6 1480

BAX 4837 8167.09090909091 5061.45454545455 8336.90909090909

BAZ1A 2584 1534 927.7 2906

BAZ1B 2935 3105 4195 4050

BAZ2A 500 1050 1080 958

BAZ2B 180.1 125.5 143.7 171.1

BBC3 2710 1010 312 2120

BBOX1 62.6 63.6 4.03 64.4

BBS1 134 221 66.9 266

BBS10 351 393 824 904

BBS12 79.5 151 199 520

BBS2 550.5 582.3 1602 1740

BBS4 793 2120 1180 2330

BBS5 8714.6 9260.85 9403 11986.5

BBS7 23 83.1 75.4 144

BBS9 230.6 142.95 200.1 524.6

BBX 1773.63636363636 2063.63636363636 5138.18181818182 4298.18181818182

BC036928 349 871 33 471

BCAM 62.8 93.9 94.7 11.7

BCAN 3.67 5.68 9.17 3.52

BCAP29 371 1172 908.5 1019

BCAP31 17100 23800 19700 45500

BCAR1 9902 12869.5 11071.5 3915.85

BCAR3 434 1800 1710 206

BCAR4 23.5 18.1 25.6 35.5

BCAS1 72.8 28.35 6.01 34.5

BCAS2 4860 5360 4970 11800

BCAS3 63.4333333333333 84.9 69.5 267.333333333333

BCAS4 244 322 198 278

BCAT1 2935 3150 8.58 2468.5

BCAT2 1400 2080 5080 1770

BCCIP 877 1799 1011.5 2475

BCDIN3D 370 1050 1000 1690

BCHE 22.6 57.1 228 683

BCKDHA 2280 2620 2810 2070

BCKDHB 460 382.333333333333 927 694

BCKDK 2221 1232.5 3200 4210

BCL10 44.8 74.3 38.2 51.5

BCL11A 203.575 38.765 17.5775 580.95

BCL11B 7.22 9.03 34.975 46.99

BCL2 6.75454545454545 7.04727272727273 121.090909090909 107.445454545455

BCL2A1 1726.65 2707.655 2297.45 1757.01

BCL2L1 1235 2138 1299 159.6

BCL2L10 15.8 5.32 6.09 3.91

BCL2L11 674.333333333333 884.333333333333 769 1177.66666666667

BCL2L12 20510 20550 24755 23175

BCL2L13 886.816666666667 2642.91666666667 975.483333333333 1111.70833333333

BCL2L14 21.81 9.991 11.435 4.466

BCL2L15 68.65 55.8 62.35 47.7

BCL2L2 1840 1330 1250 1410

BCL3 6380 7690 3100 436

BCL6 2770 752 454 185

BCL6B 5.4 5.72 7.85 539

BCL7A 1050 414 986 2730

BCL7B 994 2240 1830 1490

BCL7C 575.2 657.1 2074 1579

BCL8 12.6 21.7 36.8 53.8

BCL9 388 800 626 727

BCL9L 388.5 772.5 974.5 99

BCLAF1 8605 7160 6505 15100

BCMO1 15.2 16.8 46.2 12.4

BCO2 84.3 29.9 53.8 73.3

BCOR 302 422 414 1800

BCORL1 455 2220 554 2020

BCORL2 170.79 72.81 57.195 64.75

BCR 926 1983 1010 1477.66666666667

BCS1L 4580 5620 3310 9650

BDH1 423 6.31 773 98.3

BDH2 59.3 42.7 21.8 126

BDKRB1 4.1 28.5 37.5 11.9

BDKRB2 44.4 13.5 11.2 41.7

BDNF 10.72 12.18 152.15 5.445

BDNFOS 8.67 5 21.1 11.6

BDP1 627 643 761 922

BEAN 36.6 37.9 32.7 34.5

BECN1 3420 4260 4140 4810

BECN1L1 6.31 75.7 8.14 12.5

BEGAIN 25.2 17.6 30.5 131

BEND2 2.79 4.53 4.1 3.39

BEND3 1388 992 749.5 1358.5

BEND4 3.825 8.275 7.795 1302.5

BEND5 3.74 5.77 5.63 74.6

BEND6 95.7 25.27 13.855 54.55

BEND7 348 257.5 32.95 439

BEST1 10.408 15.38 13.676 10.872

BEST2 77.3 64.8 89.1 86.9

BEST3 5.785 21.25 11.37 131.65

BEST4 129 80.8 27.2 34.6

BET1 293.85 411.5 458.05 573.5

BET1L 192.2 305.6 201.266666666667 316.333333333333

BET3L 3.27 5.46 8.08 3.27

BEX1 24600 15900 4.96 10000

BEX2 7900 7000 932 19100

BEX4 68.4 511 4.64 2070

BEX5 11.1 196 595 739

BEYLA 2.745 8.165 4.915 4.56

BFAR 4060 2960 3320 2760

BFSP1 1064 307.6 1152 698.7

BFSP2 3.04 4.81 4.42 4.45

BGLAP 165.25 141.35 278 181.05

BGN 30.8 6.75 80.8 13.4

BHLHA15 90.1 92.6 56.8 43.6

BHLHA9 53.22 20.685 28.3 22.875

BHLHB9 32.3 501 60.6 623

BHLHE22 4.16 6.39 6.04 8.16

BHLHE23 7540 9440 7360 7560

BHLHE40 265 559 389 149

BHLHE41 68.1 77.3 11.8 181

BHMT 7.18 23 4.88 7.48

BHMT2 2170 4640 4.95 3.94

BICC1 107 83.8 147 9.4

BICD1 22.4 24.3 57.5 29

BICD2 1890 2980 2860 2970

BID 21400 25600 8740 10025

BIK 125 54.5 312 176

BIN1 673 1510 2760 573

BIN2 2.61 4.24 5.24 4.86

BIN3 867 1630 1560 1330

BIRC2 1630 1450 3540 1540

BIRC3 958 140 6120 72

BIRC5 20160 35560 34750 50430

BIRC6 355 321 289 574

BIRC7 21.5 30.7 227 55.6

BIRC8 2.64 4.27 3.87 3.24

BIVM 1023.6 230.2 945.2 624.3

BLCAP 660.5 694.5 1001.5 1094.5

BLID 5.81 6.07 3.75 3.15

BLK 3.75 5.99 5.54 4.42

BLM 2470 4750 2310 4900

BLMH 3110 7770 4040 6550

BLNK 7.68 40.725 7.83 11.175

BLOC1S1 1030 1110 1280 1500

BLOC1S2 4190 6810 1890 6990

BLOC1S3 1091.15 971.5 1035.45 1555.3

BLVRA 5400 11500 4730 11200

BLVRB 8790 12200 37200 5600

BLZF1 360.2 335.433333333333 358.5 379.666666666667

BMF 615.75 294.1 11.01 81.9

BMI1 905.5 857 131.45 1485.5

BMP1 137 146 254 47.6

BMP10 12.9 13.8 13.5 13.5

BMP15 5.262 7.897 6.915 6.321

BMP2 2470 7120 189 506

BMP2K 157 232 140.333333333333 148.666666666667

BMP3 3.52 5.495 50.55 4.375

BMP4 228 852 59.2 255

BMP5 7.81 7.4 10.6 43.1

BMP6 35.4 43.4 341 388

BMP7 6.56666666666667 8.89 9.50333333333333 3726

BMP8A 17.8 18.9 4.87 10.1

BMP8B 7054.01 6552.76 5354.17 5933.45

BMPER 4.635 47.15 4.855 69.01

BMPR1A 729.666666666667 794 609 634.666666666667

BMPR1B 8.03 6 37.2 26.8

BMPR2 80.7 110 24.9 241

BMS1 1193.2 2706 2506.2 2820

BMS1P1 109 134 65.1 199

BMS1P4 54.7 74.4 60 81.9

BMX 2.95 8.14 4.55 3.55

BNC1 3.02 4.83 4.41 41.6

BNC2 3.77 5.42333333333333 81.3666666666667 101.9

BNIP1 679 736 755 1320

BNIP2 2545 3195 1758.5 3210

BNIP3 6620 14300 2870 6480

BNIP3L 190.85 1212.5 875 742

BNIPL 19.78 19.88 11.805 48.86

BOC 5.74 9.74 16.1 18.6

BOD1 4510 4100 3550 5940

BOD1L 1670.66666666667 1913 582.333333333333 1328

BOD1P 21.37 22.07 33.4 22.85

BOK 1830 524 650 94.7

BOLA1 2200 1140 1270 1790

BOLA2B 33493.3333333333 15930 28900 42153.3333333333

BOLA3 16515 25925 16605 29385

BOLL 4.50363636363636 7.06818181818182 4.93818181818182 9.14909090909091

BOP1 5760 3760 6110 7820

BPA-1 2.61 4.23 3.83 14.8

BPESC1 4.02 6.26 11.9 4.83

BPGM 1390 2050 1680 4740

BPHL 2340 1550 2150 1190

BPI 3.395 5.465 5.04 4.14

BPIL1 201 161 116 97.2

BPIL2 3.81 4.59 6.04 3.5

BPIL3 2.98 4.82 4.41 46.8

BPNT1 1400.45454545455 808.636363636364 1121.63636363636 1258.81818181818

BPTF 258 146 121 642

BPY2B 3.53 6.5 5.3 4.69

BRAF 371.3 441.6 354.4 297.6

BRAP 294 421 642 538

BRCA1 1316 1695 2126 3136

BRCA2 246.6 308.9 345.4 286.5

BRCC3 2578.66666666667 2785 1917 6933.33333333333

BRD1 1850 2370 1250 1940

BRD2 29800 12500 23900 57700

BRD3 1124 1260.33333333333 988.666666666667 1700

BRD4 1919.33333333333 974.666666666667 608.333333333333 1883

BRD7 6860 7810 10600 14800

BRD7P3 134 128 122 292

BRD8 1521.68 1842.675 2616.9 3406.35

BRD9 936 748 1500 1050

BRDT 3.82 1050 5.61 4.47

BRE 372 825 1170 1500

BREA2 48.4 26.6 75.8 219

BRF1 1776 2070.5 2849 3811

BRF2 469 970 446 482

BRI3 20500 25700 31650 14120

BRI3BP 1240 599 1880 1260

BRIP1 217 150 204 327

BRIX1 4470 7190 5990 6410

BRMS1 5620 6030 2005 2545

BRMS1L 232 570 246 865

BRP44 35110 17730 17160 8494

BRP44L 6162.72727272727 9945.45454545455 2416.90909090909 8147.27272727273

BRPF1 234 276 344 306

BRPF3 271.6 190.5 114.15 151

BRS3 7.48 5.97 10.7 30.3

BRSK1 20.6 29.1 20.2 23.2

BRSK2 442.5 80.5 44.305 250

BRUNOL4 9.355 11.9 12.755 244.55

BRUNOL5 25.15 22.9 32.9 36.45

BRUNOL6 2000 966 1480 1130

BRWD1 342.42 395.3 460.12 430.2

BRWD2 1100 1440 1150 1890

BRWD3 122 5.17 70.8 280

BSCL2 297 700 674 587

BSDC1 944.366666666667 1391.33333333333 728.566666666667 2458.66666666667

BSG 5430 3740 9180 4740

BSN 17.2 16.4 8.1 180

BSND 13.6 13.3 4.08 14.7

BSPH1 9.31 14.1 4.87 9.21

BSPRY 677 17.4 6.3 215

BST1 29.7 42 13.7 26.6

BST2 25.735 4.94 19.7 12.02

BSX 28 26.5 13.4 19.5

BTAF1 661 951 884 1240

BTBD1 3810 4130 4150 7210

BTBD10 385 564 789 647

BTBD11 2.75 4.44 7.16 252

BTBD12 622 923 978 1060

BTBD16 261 898 2590 37.5

BTBD17 7.45 4.25 10.4 63.2

BTBD18 3.52 5.71 5.23 4.14

BTBD19 18.42 10.865 5.635 8.565

BTBD2 1410 652 1280 883

BTBD3 1050 1900 545 1840

BTBD6 13200 8320 21700 18700

BTBD7 345.575 488.225 434 629.5

BTBD8 15.705 15.15 17.45 23.7

BTBD9 266.066666666667 131.576666666667 493.233333333333 703.5

BTC 206 34.4 29.3 3.35

BTD 342 175 213 114

BTF3 10600 12100 21500 22300

BTF3L1 11290 13160 21290 21880

BTF3L4 384.6 1003.05 726.25 1143

BTG1 1300 1320 2600 1130

BTG2 2470 1180 490 1280

BTG3 2650 5450 13100 9110

BTG4 3.41 5.48 5.09 4.1

BTK 4.265 4.94 15.295 17.93

BTLA 5.07 5.6 8.95 6.27

BTN1A1 12.855 20.865 18.525 23.11

BTN2A1 738.5 683 178 773

BTN2A2 483 243 54.4 643

BTN2A3 200 174 114 379

BTN3A1 128.3 64.2333333333333 36.8333333333333 131.5

BTN3A2 893 391 67.4 1020

BTNL2 93.2 116 56 81

BTNL3 3.09 4.93 4.54 3.74

BTNL8 18.73 21.99 24.67 26.5

BTNL9 3.395 5.41 260.745 83.5

BTRC 225 466 259 785

BUB1 3410 3900 3350 4860

BUB1B 6990 4240 4190 3800

BUB3 5525 6475 5775 8010

BUD13 2100 2430 4750 2910

BUD31 3980 6950 6580 4420

BVES 106 284 29.5 362

BYSL 7440 5570 5680 11300

BZRAP1 6.02 11.2 3.9 5.81

BZW1 3550 6636.66666666667 3316.66666666667 4053.33333333333

BZW2 24400 11115 11645 10400

C10orf10 623 326 6.05 517

C10orf104 262 402 420 309

C10orf105 2.91 4.63 4.26 3.54

C10orf107 2.63 4.27 3.86 4.11

C10orf108 12.9 17.6 31.1 28.2

C10orf11 243.9 77.03 133.3 7.297

C10orf110 96.1 60 72.7 190

C10orf111 3.4 5.54 5.05 6.2

C10orf112 6.24 6.06 7.4 4.47

C10orf113 2.6 4.22 3.83 3.2

C10orf114 212 165 712 947

C10orf116 10700 14.7 355 13.9

C10orf118 91.3 203 207 132

C10orf119 3830 3930 6110 7120

C10orf12 174 204.7 162.2 252.9

C10orf120 14.265 17.7 14.52 19.455

C10orf122 3.24 5.11 4.78 5.43

C10orf125 2020 2650 1760 2660

C10orf128 2.82 4.54 4.13 3.44

C10orf129 10.365 13.78 18.86 15.835

C10orf131 3.22 5.19 4.79 3.92

C10orf134 2.99 4.86 4.41 3.65

C10orf137 376.333333333333 629.333333333333 587 989

C10orf140 11.55 30.305 49.535 171.26

C10orf18 783 1350 2830 1530

C10orf2 622 687 588 931

C10orf25 6.71 17.4 25.5 28.1

C10orf26 568 845 520 613

C10orf27 54.7 59.7 56.3 44.6

C10orf28 106.7 178.5 138 234.5

C10orf32 2490 2650 597 2540

C10orf35 1130 3340 2120 2350

C10orf4 314.3 792.85 573.4 944.2

C10orf40 2.82 4.56 4.14 3.44

C10orf41 120 50.8 373 1580

C10orf44 5.85 10.1 10.9 9.47

C10orf46 534.5 1128.5 955 1169.5

C10orf47 119.75 173.5 91.95 198

C10orf50 9.07 6.74 10.2 10.9

C10orf53 37.5 32.52 15.575 19.31

C10orf54 336 1790 452 259

C10orf55 3.36 5.41 6.99 4.1

C10orf57 259.3 267.633333333333 339.933333333333 474.133333333333

C10orf58 3832 5085 9.26 2006.5

C10orf62 14.5 13.7 15.2 16.2

C10orf67 3.355 10.7 5.9 28.58

C10orf68 3.635 5.31 8.525 4.235

C10orf71 3.25666666666667 5.24 4.79 3.88333333333333

C10orf72 4.365 5.535 5.145 9.295

C10orf75 50.5 151 139 188

C10orf76 1030 2250 1300 2380

C10orf78 341 1560 901 1200

C10orf79 3.055 4.975 24.25 12.7

C10orf81 10.585 9.67 7.175 10.285

C10orf82 16 26.6 218 210

C10orf84 384.636363636364 671.636363636364 613.090909090909 902.636363636364

C10orf88 872 1550 589 1310

C10orf90 17.165 10.45 17.83 5.735

C10orf91 49 26.3 62 24.2

C10orf92 11.8366666666667 11.5466666666667 6.18 14.48

C10orf93 2.555 4.135 3.755 13.45

C10orf95 66.2 40.7 91.1 145

C10orf96 206 261 211 272

C10orf99 4.43 4.5 15.6 3.41

C11orf1 160.46 259.35 935.9 363.765

C11orf10 25300 25800 28600 25500

C11orf16 2.6 4.21 3.81 3.2

C11orf17 1510 994 1280 1510

C11orf2 5560 2540 2040 4350

C11orf20 90.4 311 361 234

C11orf21 59.1 56.9 41.3 50

C11orf24 21500 14700 3980 5470

C11orf30 309 379 459 494

C11orf31 20800 71050 22500 37900

C11orf34 17.8 9.11 18.2 7.95

C11orf35 382 446 749 435

C11orf36 10 5.98 5.54 31.9

C11orf40 12.7 12 7.08 4.23

C11orf41 4.04 5.22 6.50666666666667 56.8666666666667

C11orf42 118 107 182 99.8

C11orf45 18.4 11.1 10.1 85

C11orf46 1820 3200 988 3070

C11orf48 12900 11000 9720 9910

C11orf49 426.5 755.5 827.5 895.5

C11orf51 346 723 959 1900

C11orf52 727 542 631 155

C11orf53 4.56 53.4 368 4.2

C11orf54 839 859.5 657.5 564

C11orf57 353 507 504 1070

C11orf58 7924.5 8135.5 5466.5 8650

C11orf59 614 1550 1300 1720

C11orf60 135.1 190.9 1648 1445

C11orf61 81.8 142 230 160

C11orf63 3.135 5.085 6.17 90.55

C11orf64 6.18 6.88 11.1 11.3

C11orf65 36.4 32 134 134

C11orf66 3.78 4.86 23.8 23.7

C11orf67 2460 4210 1790 6280

C11orf68 520 385 1280 556

C11orf70 4.81 180 5.08 141

C11orf71 311 534 685 794

C11orf73 7530 8490 14700 13400

C11orf74 2200 2450 1190 2910

C11orf75 9210 8270 6280 3400

C11orf76 309 283 438 280

C11orf80 210 213 108 33.6

C11orf82 1170 2570 3160 2240

C11orf83 18300 13700 25300 22400

C11orf84 263 262 262 375

C11orf85 22.3 30 44.9 31.9

C11orf86 6.275 9.38 6.835 6.4

C11orf87 8.8 8.52 8.59 7.09

C11orf88 6.09 5.83 10.8 4.96

C11orf9 6936 2909.5 8088.5 543.15

C11orf90 3.53 5.67 5.3 4.38

C11orf91 9.18 4.26 11.8 7.05

C11orf92 5.74 23.5 4.95 4.1

C11orf93 151 176.9 5.266 3.789

C11orf94 47.1 29.4 44.1 33.3

C11orf95 694 948 567.5 1245

C12orf10 1130 2000 2510 2200

C12orf11 1930 1975 4335 2900

C12orf12 3.885 5.065 4.815 5.055

C12orf23 4920 9000 5930 7670

C12orf24 861 2110 7490 9470

C12orf26 471 733 576 1080

C12orf27 207 174 898 11.1

C12orf28 50.9 73.3 109 52.4

C12orf29 502 1090 673 1540

C12orf30 795 1290 1520 1870

C12orf32 3020 2940 3240 3170

C12orf33 43.5 6.05 5.68 4.61

C12orf34 612 1880 609 936

C12orf35 1600 2290 1910 974

C12orf36 30.3 28.5 44.2 33.5

C12orf39 12.575 376.65 5.1 41.8

C12orf4 1610 1620 1830 1580

C12orf40 7.195 7.435 6.97 7.15

C12orf41 3824.5 4394.5 7418.5 6410

C12orf42 14.6 13.8 4.24 6.38

C12orf43 342 457 796 902

C12orf44 1310 2320 2890 1510

C12orf45 2740 3860 6620 5340

C12orf47 477 813 924 1240

C12orf48 1129.03333333333 1114.52666666667 2438.90666666667 1289.07

C12orf49 649 487 544 780

C12orf5 67.1 280 245 806

C12orf50 3.48 5.59 5.155 5.845

C12orf51 524.5 631.5 641 790.5

C12orf52 1208 2356 2432.5 2439.5

C12orf53 6.43 8.69 5.63 65.6

C12orf54 15.6 28.1 12.5 6.65

C12orf56 91 1010 217 14.1

C12orf57 5920 8520 16200 5820

C12orf59 4.4 12.4 9.83 6.4

C12orf60 226 361 396 312

C12orf61 13.7 15.7 25.3 15.1

C12orf62 20900 25800 34300 29300

C12orf63 2.62 4.25 3.85 3.22

C12orf65 2000 1680 5490 3270

C12orf66 44.13 106.85 160.55 97.65

C12orf67 11.1 4.52 4.12 3.42

C12orf68 3.78 5.9 5.69 7.1

C12orf69 3.8 4.13 3.81 4.5

C12orf70 77 35.6 126 124

C12orf71 36.6 5.25 4.85 3.98

C12orf72 12.275 25.705 43.125 38.09

C12orf73 989 886 2550 2150

C12orf74 3.43 5.5 5.07 4.09

C12orf75 2950 8460 24800 2970

C12orf76 668 1050 1150 1560

C12orf77 3.37 5.36 4.95 4.1

C13orf1 105 234 169 421

C13orf15 632 288 13.1 559

C13orf16 18.3 18.3 39.9 19.3

C13orf18 3.53 123 5.28 166

C13orf23 1214.5 1155 1162 2848.5

C13orf26 3.79 6.08 5.58 4.45

C13orf27 5650 3350 8990 2460

C13orf28 3.41 5.54 5.1 4.16

C13orf29 43 35.2 35.9 29.9

C13orf30 3.56 5.09 4.65 3.76

C13orf31 10.2 21.7 3.81 18.4

C13orf33 17.8 6.21 5.69 4.59

C13orf34 455 909 1500 2800

C13orf35 14.2 12.2 26 17.3

C13orf36 3.65 5.73 5.4 36

C13orf37 1180 3090 4840 11600

C13orf38 85.8 5.5 5.05 4.08

C13orf39 3.27 5.31 4.85 3.99

C14orf1 1188.15 822.85 833.25 938.05

C14orf101 1430 1350 801 1130

C14orf102 237 96.5 97.5 423

C14orf104 771.5 850.5 1355 1431

C14orf105 39.2 33.6 75.9 4.87

C14orf106 640 496 567 629

C14orf109 691 658 1110 852

C14orf115 5.51 5.24 4.82 3.92

C14orf118 131.3 236.3 175.85 191.75

C14orf119 4.89 5.28 4.87 3.99

C14orf126 1110 4500 520 1150

C14orf128 70.5 2125 301.5 386

C14orf129 2000 3080 3520 3150

C14orf132 315 32.7 139 360

C14orf133 1060 1270 1360 1240

C14orf135 1240 971 521 741

C14orf138 335 416 366 499

C14orf139 941 1310 558 1090

C14orf142 170 233 610 352

C14orf143 538.54 710.4 878.01 880.3

C14orf145 200.05 402 304 522

C14orf147 2860 4520 3295 3500

C14orf148 5.01 7.47 5.37 4.37

C14orf149 1070 854 283 649

C14orf153 2620 2580 3030 3190

C14orf156 41500 42600 53200 70900

C14orf159 182 7.92 690 339

C14orf162 49.5 55.9 67.8 154

C14orf165 9.395 10.665 9.765 8.165

C14orf166 17600 19700 27700 30600

C14orf166B 2.82 4.56 4.13 3.44

C14orf167 154.05 225.6 392.85 334

C14orf169 2170 2360 1910 10.5

C14orf174 4.76 7.73 43.7 77.8

C14orf176 33.7 26.8 28.7 63.9

C14orf177 3.43 4.76 4.33 3.53

C14orf178 3.52 5.66 13.5 6.55

C14orf179 1910 4050 3040 4620

C14orf180 17.5 13.2 16.1 18.3

C14orf181 3151.5 3305.55 1938.7 3066.2

C14orf182 31.275 26.15 95.65 24.375

C14orf183 3.55 5.69 5.27 5.78

C14orf184 3.38 5.37 5.05 4

C14orf2 3480 5140 3420 9710

C14orf21 188 182 123 210

C14orf22 4.4 8.04 5.1 11.6

C14orf23 3.57333333333333 38.34 20.19 127.3

C14orf25 3.48 4.41 7.46 3.91

C14orf26 3.06 4.99 4.53 3.69

C14orf28 29.7 24.6 28.2 31.6

C14orf33 114 242 582 238

C14orf34 3.52 5.69 5.27 4.34

C14orf37 6.48 5.805 100.6 170

C14orf38 4.13 6.26 5.9 4.9

C14orf39 3.55 5.71 5.29 4.32

C14orf4 88.5 107 105 88.3

C14orf41 45.8 63.3 24.7 19.9

C14orf43 1090 1270 1580 809

C14orf45 7.18 99.8 62.75 44.8

C14orf48 15.78 16.0533333333333 13.1133333333333 13.4666666666667

C14orf49 23.5 31.6 39.9 15.1

C14orf50 12.1 10 24.8 6.05

C14orf53 3.245 5.205 4.79 3.945

C14orf64 3.24 8.77 5.55 7.71

C14orf68 54.7 44.2 36.6 37.7

C14orf70 987 1160 1050 897

C14orf72 9.24 65.3 741 36.9

C14orf73 451 180 116 25.8

C14orf79 21.6 7.34 58.4 43.7

C14orf80 3855 3024 5545 4470

C14orf81 2.59 4.19 3.8 3.19

C14orf86 2.55 4.13 3.74 3.14

C14orf91 3.45 5.62 5.17 4.17

C14orf93 734 890 602 880

C14orf99 4.88 4.23 3.83 3.21

C15orf17 193 156 181 250

C15orf2 116 72 104 110

C15orf23 19400 14900 15300 12800

C15orf24 18000 16700 19000 14400

C15orf26 5.89 8.95 5.06 17.7

C15orf27 3.37 55.8 5.77 1060

C15orf28 13400 13300 12200 14400

C15orf29 115 108 68.8 79.2

C15orf32 4.32 4.55 4.94 3.97

C15orf33 8.63 52.105 4.21 48.955

C15orf34 26 9.31 19.3 22.8

C15orf37 65.3 55.8 23 78.2

C15orf38 172 373 395 354

C15orf39 5460 8140 1550 6340

C15orf40 2070 2110 1630 3770

C15orf41 842 454 362 543

C15orf42 476 720 749 998

C15orf43 14.2 345 5.47 5.22

C15orf44 1340 1430 1350 3400

C15orf48 36.9 18.1 9.02 173

C15orf5 143 119 60.6 89.8

C15orf50 22.4 28 91.3 28.8

C15orf51 99.452 11.538 11.532 45.38

C15orf52 4180 533 1740 333

C15orf53 3.23 5.25 4.79 3.84

C15orf54 2.64 18.2 3.87 3.25

C15orf55 7.15 5.86 6.975 5.045

C15orf56 2.55 4.13 3.74 3.15

C15orf57 745 466 779 783

C15orf58 151 114 348 379

C15orf59 189 205 389 1350

C15orf60 3.9 18.4 5.67 4.77

C15orf61 3300 3570 2940 3770

C15orf62 89.1 83.7 83.7 68.28

C15orf63 16320 6685 13310 17460

C16orf10 16.8 13 15.4 23.2

C16orf11 3.43 5.51 5.09 21.9

C16orf13 6790 6770 10700 15400

C16orf3 32.9 39.3 36.9 42.5

C16orf35 5210 5430 12700 6550

C16orf38 8.09 5.98 8.95 4.47

C16orf42 1710 2240 4530 4160

C16orf45 3.08 95.3 4.53 154

C16orf46 3.98 6.18 5.75 4.69

C16orf48 467 708 2420 1370

C16orf5 2947 2935 751 3015.5

C16orf52 712 620 376 688

C16orf53 261 1030 1410 974

C16orf54 3.48 5.49 5.08 4.14

C16orf55 86.4 194 385 250

C16orf57 7680 10900 8100 13900

C16orf58 820 1040 1610 991

C16orf59 2810 4070 4620 7030

C16orf61 7669 11470 10730 7679

C16orf62 3.79 164 531 449

C16orf63 11700 11500 10800 11200

C16orf65 2.54 4.11 3.73 3.13

C16orf67 346 128 260 370

C16orf68 1220 882 1350 1090

C16orf7 23114 26640 24876.5 25094

C16orf70 1720 1850 1760 2590

C16orf71 4.58 6.61 12 8.65

C16orf72 1036.705 767.765 624.965 1082.06

C16orf73 1940 10.4 1840 12.1

C16orf74 33.6 75.3 493 249

C16orf75 4960 4000 5640 6290

C16orf78 17.5 21.3 22.7 21.6

C16orf79 99.1 248 799 223

C16orf80 2230 4520 6340 6640

C16orf81 3.98 62.6 5.79 4.74

C16orf82 6.81 4.13 5.9 3.37

C16orf86 22.1 33.4 16 37.6

C16orf87 564 904 997 2070

C16orf88 3250 2850 5390 5200

C16orf89 44.2 75.3 79.7 85.2

C16orf90 60.7 55.4 60.7 78.2

C16orf91 5950 5190 9560 12500

C16orf92 6.55 8.69 11.1 15.4

C16orf93 27.2 38.2 195 188

C17orf100 176 39.1 78.1 107

C17orf101 412 363 996 573

C17orf102 4.57 4.43 4.02 4.67

C17orf103 22.4 17 20.5 26.8

C17orf104 8.865 14.055 20.65 25.1

C17orf105 28.8 62.3 25.1 51.1

C17orf106 2760 1930 3070 2340

C17orf107 216 73.2 161 33.1

C17orf108 55.3 172 1380 525

C17orf13 7.14 4.57 21.5 7.92

C17orf28 1200 134 407 242

C17orf37 7970 6570 16100 14900

C17orf39 944 2720 2130 2940

C17orf42 1330 1320 2070 1770

C17orf44 10.1 5.13 6.41 3.85

C17orf46 48.3 50.2 36.4 43.7

C17orf47 3.56 5.73 5.35 4.34

C17orf48 722 408 313 459

C17orf49 2890 3460 6700 4840

C17orf50 76.3 71.6 105 88.4

C17orf51 1270 3770 1070 3360

C17orf52 27.9 16.5 25.7 24.5

C17orf53 127 165 146.5 219.5

C17orf54 67.25 37.77 100.55 94.225

C17orf55 633 833 455 323

C17orf56 4960 3670 4310 7590

C17orf57 4.195 5.505 62.51 4.025

C17orf58 698 564 775 1410

C17orf59 498 361.5 578 511

C17orf6 4.02 6.33 5.93 5.11

C17orf60 2.56 4.15 3.76 3.15

C17orf61 6240 11600 9730 12800

C17orf62 692.090909090909 640.545454545455 929.909090909091 607.363636363636

C17orf63 2443.5 3920 2609.5 1959.5

C17orf64 5.38 6.94 11 10.1

C17orf65 294 254 277 218

C17orf66 5.47 5.68 4.11 6.08

C17orf67 21.45 101.8 118.2 154.15

C17orf68 215 221 225 146

C17orf69 47.4666666666667 36.3 56.7 454.333333333333

C17orf70 684 407 883 1080

C17orf71 1640 1440 1380 2880

C17orf72 3.34 10.3 4.92 162

C17orf73 3.565 6.9 4.98 4.58

C17orf74 117 149 159 128

C17orf75 718 1040 1120 1360

C17orf76 139.5 274 254 224

C17orf77 9.44 9.33 10.6 7.57

C17orf78 3.58 5.81 5.35 8.6

C17orf79 22100 22300 36900 31300

C17orf80 912 823.5 1002.5 1496

C17orf81 1390 3050 2740 1550

C17orf82 626 63.3 64.7 98.9

C17orf85 473 570 563 578

C17orf86 224 64.1 148 458

C17orf87 10.78 11.08 11.6833333333333 14.7066666666667

C17orf88 78.4 34.1 98 149

C17orf89 28100 23950 61400 58150

C17orf90 10600 5190 19400 16800

C17orf91 834 846 482 416

C17orf93 416 452 246 398

C17orf95 1990 2160 2800 2040

C17orf96 2530 2780 2250 5080

C17orf97 335 58.9 281 120

C17orf98 10.4 6.3 15.2 32.4

C17orf99 30.6 4.26 65.2 3.24

C18orf1 16.2 57.1 5.1 103

C18orf10 3380 5260 10400 17900

C18orf15 2.73 5.28 6.94 14.8

C18orf16 12.9 5.09 5.16 3.62

C18orf18 3.83 409 5.77 23.3

C18orf19 519 1180 238 545

C18orf2 14.57 17.99 5.185 45.7

C18orf20 3.24 7.33 4.81 9.26

C18orf21 1981 1406 2358 7625

C18orf22 3450 3130 4650 3480

C18orf23 40100 46400 41600 46900

C18orf25 379 641 987 772

C18orf26 4.81 5.67 5.2 4.22

C18orf32 775 933 1010 365

C18orf34 15.8166666666667 12.7866666666667 5.39666666666667 18

C18orf45 280 378 313 599

C18orf54 83.1 252 279 241

C18orf55 3230 4720 7390 4490

C18orf56 867 125 5900 235

C18orf57 2.65 4.3 3.92 3.26

C18orf62 8.24 7.37 9.54 3.74

C18orf8 638 711 987 1260

C19orf10 109000 27900 27500 38200

C19orf12 857.5 929 519.5 674.5

C19orf18 128 14.6 28.2 4.61

C19orf2 930.5 1064.5 1449 1813

C19orf20 181 107 220 171

C19orf21 92 324 2780 5.98

C19orf22 3060 1960 1100 1350

C19orf23 403 222 206 1280

C19orf24 30600 10700 21600 19000

C19orf25 1275.5 511.5 645 1147.5

C19orf26 58.9 41 63.4 39.4

C19orf28 522 365 643 824

C19orf29 4650 3220 1830 3850

C19orf30 8.915 8.64 9.035 14.135

C19orf33 31.3 4360 28200 81.6

C19orf34 10.7 5.24 8.55 5.58

C19orf36 16.5 14.8 39.8 25.3

C19orf38 10.9 9.8 27.1 14.4

C19orf39 44.3 48.5 63 60.8

C19orf40 69.8 94.2 56.1 86.8

C19orf41 5.938 5.535 6.923 6.754

C19orf42 2720 2505 2185 2950

C19orf43 1880 1030 1770 2150

C19orf44 630 511 548 1030

C19orf45 12.2 23.3 12.5 34.1

C19orf46 759 368 976 84.6

C19orf47 75.1 153 121 156

C19orf48 7580 12900 11100 13600

C19orf50 1060 1160 1530 1760

C19orf51 13.5 7.73 275 677

C19orf52 2670 1300 2890 4130

C19orf53 68.2 55.9 134 120

C19orf54 57.1 27.6 82 75.9

C19orf55 81.2 99.5 114.75 124.15

C19orf56 11000 5570 8660 11200

C19orf57 86.7 10.9 146 687

C19orf59 19.3 23.4 26.2 16.8

C19orf6 24800 10800 13200 14500

C19orf60 2680 3110 3580 4325

C19orf61 317 355 564 418

C19orf62 4350 3720 4390 4980

C19orf63 14.62 1234.75 10489 7919.5

C19orf66 2030 1570 1050 986

C19orf67 16 13.3 18.8 22.9

C19orf68 1570 1220 3280 1070

C19orf69 326.5 311.84 385.12 272.63

C19orf70 6380 2030 6320 9260

C19orf71 7.93 5.07 8.83 8.51

C19orf73 87.75 91.75 215 186.5

C19orf75 3.27 5.32 4.86 3.87

C19orf76 3.42 5.49 5.07 4.1

C19orf77 26.8 7.41 845 24.7

C1D 1673.5 1929 1642.5 2083.5

C1GALT1 3210 2040 1220 537

C1GALT1C1 2550 4280 1660 6760

C1QA 11.2 20.8 29.1 21.9

C1QB 7.05 4.17 8.62 4.59

C1QBP 94700 84800 92800 139000

C1QC 3.59 6.33 9.29 6.65

C1QL1 7.6 20.7 35.3 63.2

C1QL2 3.4 5.49 5.03 4.04

C1QL3 6.739 5.799 4.851 4.503

C1QL4 38.9 91.6 502 108

C1QTNF1 31.9 19.3 6.07 17.4

C1QTNF2 14.8 12.5 12.1 24.7

C1QTNF3 64.43 36.04 6.069 5.342

C1QTNF4 489 219 4.57 10.1

C1QTNF5 114 50.4 62.2 25.1

C1QTNF6 402 58.1 553 102

C1QTNF7 2.76 4.49 4.07 3.38

C1QTNF8 3.74 10.7 24.4 4.59

C1QTNF9 3.73 5.915 320.5 8.165

C1R 830.5 848.5 69.25 464.5

C1RL 95.85 116.9 39.19 13.37

C1S 657.7 892.3 21.42 71.465

C1orf100 7.23 16.3 5.92 11.1

C1orf101 23.8 13.5 21.7 35.4

C1orf103 344 238 275 225

C1orf104 1260 1200 842 835

C1orf105 74.3 7.92 4.64 3.83

C1orf106 432.2 1332 1114 73.64

C1orf107 152 123 123 436

C1orf109 593 702 781 1660

C1orf110 2.8 4.56 4.15 3.41

C1orf111 12.3 8.49 26 17.4

C1orf112 5057 3750 1991 4977

C1orf113 34.2 78.95 57.8 74.65

C1orf114 49.6 191 104 1170

C1orf115 309 189 157 146

C1orf116 146 7.47 402 14.4

C1orf118 13.05 20.15 8.99 65.1

C1orf120 2.92 4.66 4.27 3.54

C1orf122 5550 6710 10700 8740

C1orf123 1810 2990 3650 3260

C1orf124 452 294.5 370.5 1150

C1orf125 3.83 6.09 240 6.02

C1orf126 2.55 4.14 3.75 3.15

C1orf127 10.4 10.8 18.2 10.4

C1orf128 333 510 920 692

C1orf129 2.975 4.73 4.35 3.595

C1orf130 23.6 23.7 5.72 4.61

C1orf131 2220 1180 1260 3590

C1orf133 3.34 5.43 30.2 397

C1orf135 418 1390 2210 2450

C1orf138 12.4 5.06 4.62 3.74

C1orf14 6.97 5.65 5.2 4.43

C1orf141 3.255 5.14 7.915 4.71

C1orf143 19.1 10.2 97.1 17.2

C1orf144 4244.36363636364 6688.18181818182 6160.90909090909 4673.18181818182

C1orf146 3.7 5.96 5.56 4.57

C1orf150 2.78 4.51 4.09 3.38

C1orf151 13525.7 12785.7 30176.05 14371.8

C1orf152 11077.5 9177.5 9497.5 5847.5

C1orf156 610 497 474 819

C1orf157 2.9 4.72 4.28 3.51

C1orf158 532 555 346 433

C1orf159 408.7 342.033333333333 605.8 297.266666666667

C1orf161 50.4 88.4 56.8 46.1

C1orf162 20.3 51.6 16.6 40.2

C1orf163 4070 4850 3320 6230

C1orf167 18.8 9.21 16.8 14

C1orf168 43.8 5.49 5.03 4.13

C1orf170 12.6 11.1 18.2 20.4

C1orf172 1182.05 200.6 502.3 73.1

C1orf173 3.74 5.95 5.49 4.41

C1orf174 5410 7710 8020 4890

C1orf175 215 170 245 162

C1orf177 31 32.6 34.4 97.5

C1orf180 2.92 4.67 4.28 3.54

C1orf182 536 569 314 1080

C1orf183 448 561 287 309

C1orf185 5.36 4.325 3.925 3.28

C1orf186 558.055 634.62 2809 647.1

C1orf187 16.2 11.6 31.2 24

C1orf189 22.2 15.2 17.1 25.1

C1orf190 10.5 16.8 34.1 115

C1orf192 3.83 5.47 10.9 4.13

C1orf194 3.33 5.84 7.73 6.15

C1orf195 15 5.7 16.6 4.42

C1orf198 12600 4870 7170 10300

C1orf200 359 294 236 227

C1orf201 75.6 76.8 200 247

C1orf203 34.8 69.9 47.35 86

C1orf204 2.84 4.61 4.18 3.48

C1orf21 17.7036363636364 12.4527272727273 71.9272727272727 304.090909090909

C1orf210 27.7 4.91 37 3.66

C1orf211 36.3 77.1 87.7 137

C1orf212 791 1040 684 1190

C1orf213 54.835 87.65 283.5 158.75

C1orf216 118 122 298 141

C1orf217 56.6 75.6 56.7 77.8

C1orf220 179 79.4 65.9 125

C1orf223 5.91 6.4 5.63 12.8

C1orf226 816.636363636364 297.181818181818 999.090909090909 758.627272727273

C1orf227 6.215 36.7 13.75 40.55

C1orf228 17.7 22.6 44.7 55

C1orf229 1250.1 1378.35 1421.85 947.1

C1orf230 66.3 49.6 75.2 93.6

C1orf231 35.2 25.1 13.6 30.5

C1orf25 235 172 96.7 364

C1orf26 136 156 43.7 98

C1orf27 3310 1590 1220 1420

C1orf31 22600 9410 9180 23500

C1orf35 5635 3085 4590 12825

C1orf38 20.4 102 34.6 109

C1orf43 8420 7315 5885 9960

C1orf46 40.5 47.2 29.2 41.6

C1orf49 5.045 5.04 7.015 4.355

C1orf50 1710 1650 2340 2600

C1orf51 5.01 7.76 19.1 7.15

C1orf52 654.5 1416 740.5 2135

C1orf53 1660 920 947 1350

C1orf54 170 91.3 274 1810

C1orf55 862 457 561 1330

C1orf56 1887.5 1868.5 769.5 2045

C1orf57 2490 2450 822 3290

C1orf58 456 202 280 280

C1orf59 6.84 12.7 12.7 14.1

C1orf61 6.09 6.87 4.65 28.2

C1orf63 633 531 1030 589

C1orf64 172 38.9 9.64 18.1

C1orf65 2.8 4.55 17.5 3.42

C1orf66 1040 1550 1330 1210

C1orf68 29.1 36.1 18.5 39.2

C1orf69 377 84 233 779

C1orf70 63.55 55.6 60.6 63.9

C1orf74 64.39 46.28 140.7 233

C1orf77 4710 7680 3720 9900

C1orf81 102 45.1 152 8.95

C1orf83 107.8 199.75 97.6 473

C1orf84 3.38 5.51 6.17 4.01

C1orf85 2969 2289 682 1272.5

C1orf86 769.355 494.665 1034.78 499.06

C1orf87 6.11 6.31 6.04 5.19

C1orf88 32.4 33.2 35 34.7

C1orf89 61.75 55.5 134.4 133.75

C1orf9 13290 6062 2587 3037

C1orf91 158 301.5 293.5 570

C1orf92 3.57 7.78 15 4.37

C1orf93 4800 276 11800 1230

C1orf94 11.6 7.86 24.2 8.88

C1orf95 16.85 13.84 16.35 29.115

C1orf96 916 1145 1229.5 2219.5

C1orf97 1139 2011.81818181818 84.7 1174.81818181818

C2 1564.5 357 66.45 61.2

C20orf103 5.71 8.31 5.73 23.2

C20orf106 37.6 99.2 292 149

C20orf107 4.82 23.4 75.2 53

C20orf108 3090 2280 938 2070

C20orf11 4330 8510 4130 4650

C20orf111 3390 3320 3540 2890

C20orf112 35.9333333333333 55 24.5666666666667 14.7066666666667

C20orf114 3.06 4.98 4.52 3.73

C20orf117 30.7 30.4 24.6 27.1

C20orf118 2.56 4.15 3.76 3.17

C20orf12 59.8 107 225 217

C20orf123 2.72 4.37 3.98 9.27

C20orf132 42.18 19.84 61.35 21.2

C20orf134 50.9 94.3 44.7 54.2

C20orf135 17.8 30.3 18.4 15.9

C20orf141 1190 10700 6190 6740

C20orf144 142 120 125 198

C20orf151 41.9 43.8 43.4 43.7

C20orf152 4.03 6.29 5.91 4.86

C20orf160 20851.96 24303.065 22774 24302.31

C20orf165 103 64 63.3 71.4

C20orf166 2.86 4.6 4.19 3.48

C20orf173 4.24333333333333 8.36333333333333 6.74666666666667 3.71666666666667

C20orf177 1461.85 4065 1379.1 3924.5

C20orf185 6.03 4.93 26.7 3.82

C20orf186 4.08 4.25 3.84 3.22

C20orf194 479 438 773 422

C20orf195 4.01 6.22 99.7 4.7

C20orf196 97.2 341.866666666667 163.233333333333 331.6

C20orf197 7.7 4.6 4.98 3.47

C20orf199 18000 23400 20700 10900

C20orf20 2810 5010 3560 2870

C20orf200 15.35 10.25 10.15 8.33

C20orf201 2359.28 2397.2 2221.2 1955.7

C20orf202 3.74 5.99 5.49 4.39

C20orf24 56120 56420 28430 23610

C20orf26 9.74666666666667 11.0433333333333 5.75333333333333 17.1433333333333

C20orf27 20035 35905 22550 33440

C20orf29 1690 1680 613 1550

C20orf3 623 1490 1060 709

C20orf30 5515 7015 2390 4905

C20orf4 2840 2980 2400 4080

C20orf43 5590 7320 7240 6050

C20orf46 161 138 23.2 281

C20orf54 3.01 4.9 52.9 3.63

C20orf56 5260 6170 54.9 3.79

C20orf7 3930 4810 2550 3450

C20orf70 3.26 5.3 4.82 3.91

C20orf71 4.04 6.235 6.855 6.645

C20orf72 8450 13100 6560 10800

C20orf79 4.16 6.27 5.92 4.94

C20orf85 24.4 20.7 30.9 18.1

C20orf94 1180 1480 1570 1080

C20orf95 132 57.55 65.3 59.65

C20orf96 176 268 161 168

C21orf111 118 11.7 4.72 3.79

C21orf116 4.52 18.6 3.88 11.6

C21orf117 92.2 110 130 117

C21orf119 135 128 809 528

C21orf121 4.03 9.46 7.33 8.86

C21orf122 11.9 33.7 52.4 136

C21orf125 14.1 7.16 38.6 3.79

C21orf128 3.09 5.04 4.58 3.72

C21orf129 2.58 4.18 3.8 3.18

C21orf130 3.47 12.3 5.17 4.17

C21orf131 10.8 24.4 10 4.89

C21orf135 12.7 8.21 12.6 12.9

C21orf15 4.64 5.13 67.4 18.1

C21orf2 521.3 264.8 501.4 439.7

C21orf29 5.84 6.575 6.285 5.44

C21orf33 4290 2566 4853 7834

C21orf34 45.8333333333333 119.033333333333 110.866666666667 67.0833333333333

C21orf45 1800 3040 4400 7800

C21orf49 23.4 12.8 11.2 26.6

C21orf54 9.23 4.44 10.1 7.01

C21orf56 70.2 203 296 316

C21orf57 252.166666666667 126.433333333333 1656.66666666667 707.333333333333

C21orf58 470.5 522 696.5 637.5

C21orf59 7010 8490 12800 20800

C21orf62 5.82 4.46 7.055 7.63

C21orf63 34.45 36.3 42.25 47

C21orf66 325.5 331.5 387 648

C21orf67 22.95 38.6 143.5 146.6

C21orf7 27.875 36.155 24.48 19.82

C21orf70 929.333333333333 1125.33333333333 2346.66666666667 2636.66666666667

C21orf71 4.22 9.37 14.3 49.4

C21orf74 5.95 5.42 5.12 4.05

C21orf81 44.4 12.4 83.8 120

C21orf82 9.96 11.5 12.4 4.88

C21orf84 11.9 4.6 4.22 3.52

C21orf87 63.9 58.4 50.9 51.5

C21orf88 458.9425 504.125 432.635 487.92

C21orf89 19.85 20.77 21.15 21.15

C21orf90 168 35.8 597 253

C21orf91 196 151 313 335

C21orf93 256 286 216 283

C21orf94 3.825 6.035 5.645 4.615

C21orf96 2.81 4.54 8.4 3.42

C21orf99 5.96 7.78666666666667 8.13333333333333 9.13666666666667

C22orf13 3809 6838 3946.5 3720

C22orf15 143 131 153 132

C22orf23 10.8 14.9 26.5 75.5

C22orf24 10.7 4.75 11.1 20.1

C22orf25 2085 4284.5 1933.5 1759.5

C22orf26 3.48 5.66 5.19 4.11

C22orf27 962 1180 176 455

C22orf28 8520 10100 8420 13500

C22orf29 2510 3630 1490 2610

C22orf30 28.3 41.1 16.8 37.2

C22orf31 3.43 4.38 7.39 3.3

C22orf32 11.7 16.5 18.6 38.4

C22orf33 2.88 4.62 4.23 3.5

C22orf34 11.045 6.07 10.525 62.275

C22orf36 399 778 489 564

C22orf39 385 629 440 415

C22orf40 1640 1895 1755 2740

C22orf41 16.55 15.55 39.1 47.85

C22orf42 15.4 22.3 19.1 17.3

C22orf43 367 407 1010 429

C22orf46 127 83.5 68.7 135

C22orf9 940 678 555 747

C2CD2 160 231 481 82.5

C2CD2L 1430 1860 5650 2420

C2CD3 263.25 699.45 790.5 830.8

C2CD4A 40.4 5.35 6.97 4.14

C2CD4B 2.65 24.6 4.76 3.28

C2CD4C 724 468 335 547

C2CD4D 11.1 13.1 20.6 9.88

C2orf14 561.765 532.815 506.2 542.205

C2orf15 93.9 19.5 89.6 147

C2orf16 170 7.99 5.55 3.53

C2orf18 545 682 1360 1040

C2orf24 356 276 384 231

C2orf27A 12.9 23.9 57.3 152

C2orf27B 2.74 4.46 4.04 3.34

C2orf28 895 1760 2780 1340

C2orf29 2444 3866 1881 1932

C2orf3 1110.88545454545 1132.72727272727 696.76 1485.54545454545

C2orf34 164.05 142.35 478.8 309

C2orf39 5.38 5.92 4.9 4.4

C2orf40 4.1 6.27 5.91 4.88

C2orf42 551 691 318 580

C2orf43 999 932 1000 727

C2orf44 713 907 574 1810

C2orf46 8.85 8.27 11.6 8.95

C2orf47 8680 6130 5120 5540

C2orf48 14.8 38 8.4 99.8

C2orf49 557.5 675 578 551.5

C2orf50 111.25 117.325 132.6 106.625

C2orf51 18.2 12.8 20.7 16.3

C2orf52 11.3 136 81.2 27.3

C2orf53 14 11.8 9.44 19.2

C2orf54 416 5.72 5.6 4.77

C2orf55 19.95 9.84 17.505 20.55

C2orf56 2170 1940 3030 2850

C2orf57 2.9 4.68 4.26 3.6

C2orf58 2.9 4.65 4.26 3.53

C2orf60 141 337 126 296

C2orf61 23.2 19.5 32.3 32.6

C2orf62 5.7 4.8 9.59 9.95

C2orf63 181 27.9 130 114

C2orf64 1436.5 1920 1711.5 1592

C2orf65 4.71 29.6 6.49 10

C2orf66 43.6 22.6 41.8 29.9

C2orf67 23.7 18.2 16.8 18.8

C2orf68 472.5 366.5 260 477

C2orf69 637 1235 986.5 1076.5

C2orf7 1320 1310 1060 890

C2orf70 24.4 25.8 272 33.6

C2orf71 3.32 5.38 4.91 3.94

C2orf72 2774.5 1695.5 69.355 81.3

C2orf73 8.7 8.09 18.8 11.9

C2orf74 298 411 678 389

C2orf76 704 932 1240 1540

C2orf77 2.91 4.74 4.3 7.37

C2orf78 3.69 5.82 5.56 4.59

C2orf79 4080 2650 2260 5640

C2orf80 3.21 5.22 4.77 3.91

C2orf81 46.8 25.6 117 138

C2orf82 1220 670 157 241

C2orf83 6.77 5.54 8.13 8.99

C2orf84 3.35 5.44 5.01 31.2

C2orf85 15.9 16.9 16.9 11.7

C2orf86 44.9 43.05 71.35 94.35

C2orf88 338 38.5 135 48

C2orf89 555.5 28.4 39.75 26.4

C2orf90 3.01 4.76 4.42 3.63

C3 13500 743.5 24.705 74.1

C3AR1 701 866 593 653

C3P1 2.96 4.71 4.35 3.59

C3orf1 12000 16300 26200 22700

C3orf10 17679.5 14261.3333333333 20603.7333333333 12486.6333333333

C3orf14 1510 4.7 1700 4100

C3orf15 8.615 5.73 5.435 4.71

C3orf16 2.67 4.33 3.92 3.28

C3orf17 187 334 163 629

C3orf18 39.6 309 28.2 627

C3orf19 2705 1970 2225 1880

C3orf20 11.2 6.57 19.6 24.7

C3orf21 999 566.5 2400 627

C3orf22 85.3 96.9 81 101

C3orf23 336.95 1171.5 418.5 803.8

C3orf24 4.57 5.67 12.4 6.93

C3orf25 181 427 182 136

C3orf26 10300 8610 21300 23500

C3orf27 7.73 9.38 8.37 5.03

C3orf30 3.08 4.93 6.87 3.74

C3orf31 3310 2870 3680 6590

C3orf32 439 172 80.1 192

C3orf33 201 181 141 498

C3orf34 77.6 162 217 183

C3orf35 32.8 39.35 44.2 39.1

C3orf36 3.46 4.47 5.81 3.37

C3orf37 742 865 1840 1040

C3orf38 1210 5730 831 1770

C3orf39 290 470 684 746

C3orf42 23.4 30.2 30.3 63.4

C3orf43 26.8 32.5 37.6 30.5

C3orf45 9.45 9.02 14.1 7.11

C3orf47 20.3 11.4 15.1 114

C3orf48 2.82 4.53 101 5.21

C3orf49 3.22 5.19 4.79 3.92

C3orf50 105.5 102.5 4.99 71.35

C3orf51 163 199 168 196

C3orf52 692 921 1070 1030

C3orf54 14400 14100 15200 15100

C3orf55 107 5.9 5.75 4.97

C3orf57 123 515 23.2 4.16

C3orf58 222 280 374 330

C3orf59 38.1 338 103 516

C3orf62 29.4 60.8 34.8 52.5

C3orf63 429 491 654.1 1070

C3orf64 34.1 33.9 38.4 51

C3orf65 121 124 110 126

C3orf66 2.9 4.67 4.25 3.52

C3orf67 26.6 14.2 54.6 144

C3orf70 4.41 99.4 4.72 27.6

C3orf71 18.2 44.7 61.4 53

C3orf72 2.83 7.15 21.7 36.9

C3orf74 2.8 4.55 4.12 5.03

C3orf75 1955 5190 3920 6215

C3orf77 3.92 6.19 5.67 4.6

C3orf79 3.67 5.7 5.56 4.83

C4B 149 45.7 36.9 42.3

C4BPA 642 209 25.5 20.8

C4BPB 1900 137 8.79 4.18

C4orf10 4.86 6.32 5.96 4.93

C4orf11 8.16 8.79 8.95 12.8

C4orf12 27.9 82.15 614.7 128.7

C4orf14 7790 2910 3770 5550

C4orf17 15 25.9 17.8 17.6

C4orf19 1470 520 232 3.86

C4orf21 118.14 190.165 111.935 253.505

C4orf22 2.99 7.66 7.57 16.8

C4orf23 683 785.5 676.3 632

C4orf26 3.06 20.2 18 28.8

C4orf27 557 1340 423 1640

C4orf29 200 218 199 261

C4orf3 7370 7185 4420 6275

C4orf31 7.373 8.947 6.242 56.34

C4orf32 327 693 490 1000

C4orf33 437 304 354 1050

C4orf34 2319 2035 335.9 127

C4orf35 3.82 6.02 5.78 4.9

C4orf36 3.49 9.33 18.5 24.9

C4orf37 3.29 5.3 4.89 4

C4orf38 48.1 65 58.6 28.4

C4orf39 16.315 15.01 55.325 11.865

C4orf40 7.83 11.975 9.725 8.225

C4orf41 187 187 119 318

C4orf42 1580 1310 439 1060

C4orf43 1100 2630 1740 2870

C4orf44 37.5 29.575 47.85 35.515

C4orf45 5.06 6.19 5.83 4.85

C4orf46 1996.5 2925 1388.5 2241

C4orf47 40.8 48.4 45.5 50.2

C4orf48 13100 11900 10500 14200

C4orf49 21 101 11.6 1570

C4orf50 13.9 18.1 10.4 21.4

C4orf51 3.08 5.01 4.56 3.76

C4orf52 4400 5630 2270 1570

C4orf6 3.33 5.42 4.94 3.95

C4orf7 4.22 4.92 4.98 3.67

C5 952 2600 103 10.6

C5AR1 2.88 4.66 41.3 12.1

C5orf13 7601 2994.5 949.7 2394.5

C5orf15 1678 2850 3745 4770

C5orf17 862 953 991 870

C5orf20 16.2 12.9 28.4 22.3

C5orf22 597 500 669 576

C5orf23 3.06 4.96 4.5 3.68

C5orf24 747 937 1300 1290

C5orf25 358 518 334 2670

C5orf26 6690 6040 6160 15800

C5orf27 46.3 56.1 68.7 115

C5orf28 404.6 987.5 1098 316.5

C5orf30 1452 1399 784.4 1439

C5orf32 9710 6400 24900 5310

C5orf33 1870 688 365 260

C5orf34 229 370 401 314

C5orf35 986 717 1270 907

C5orf36 9.175 25.75 10.8 9.53

C5orf37 504 723 974.5 888

C5orf38 3.95 4.34 8.77 8.17

C5orf39 322 843 168 455

C5orf4 1150 1450 59.7 50.3

C5orf40 88.4 102 86.3 84.5

C5orf41 270 282 396 327

C5orf42 158.966666666667 228.333333333333 389 312.666666666667

C5orf43 1480 2340 2080 2140

C5orf44 316.166666666667 319.083333333333 485.666666666667 580.916666666667

C5orf45 677 1280 2570 3060

C5orf46 2.64 4.29 3.88 3.24

C5orf47 3.53 5.64 5.26 4.26

C5orf48 3.72 5.68 5.61 4.87

C5orf49 3.35 8.15 16.7 22.4

C5orf50 7.94 231 7.54 11.3

C5orf51 2080 3220 1610 2400

C5orf52 11.3 14 16.5 10.3

C5orf53 46.7 12.6 244 165

C5orf54 176 256 322 438

C5orf55 137 122 350 237

C5orf56 2.85 4.57 4.17 4.48

C5orf58 1410 1430 1020 516

C5orf60 34.6 4.99 9.26 17.6

C5orf62 177 1410 1080 1350

C6 316 24.5 24 3.66

C6orf1 5850 3840 6300 5870

C6orf10 7.85 9.135 10.72 9.37

C6orf103 3.91 6.15 5.67 4.58

C6orf105 3.85 6.14 5.62 4.5

C6orf106 1790 1307 1013 832.5

C6orf108 18900 7980 22900 36400

C6orf114 91.4 29.6 29.4 91.7

C6orf115 13500 6890 5100 17700

C6orf118 102 4.17 3.8 13.1

C6orf120 245 589 235 410

C6orf122 16.5 9.07 7.31 132

C6orf123 7.775 5.62 5.04 4.08

C6orf124 129 41.8 26.4 31.6

C6orf125 16100 5430 6530 9510

C6orf126 21.4 35.5 79.9 554

C6orf127 6.455 7.08 16.4 7.635

C6orf129 15450 7580 6745 12080

C6orf130 4420 3760 2960 2970

C6orf132 151.666666666667 16.1066666666667 324 7.75666666666667

C6orf134 172 27.8 5.21 143

C6orf136 1270 837 1100 868

C6orf138 17 14.6 48.4 14.4

C6orf140 13.88 15.38 18.765 16.64

C6orf141 65.1 151 4.38 33.6

C6orf142 42.2 73.9 7.31 4.02

C6orf145 669 337 41.1 48.3

C6orf146 3.33 5.27 4.97 3.94

C6orf147 7.18 17.9 4.3 3.54

C6orf15 3.05 4.97 10.2 3.68

C6orf150 12.9 18.3 9.86 18.4

C6orf153 18400 14700 16900 21900

C6orf154 21.2 43.1 101 89.2

C6orf155 3.71 5.95 501 18.7

C6orf162 108 150 128 261

C6orf163 145 85.2 50.2 113

C6orf164 6.585 5.33 4.48 27.045

C6orf165 3.75 5.99 20.1 24.7

C6orf167 661.5 754.5 800 932

C6orf168 96.4 9.14 7.42 231.5

C6orf170 14 44.75 33.35 103.25

C6orf173 8800 7930 7980 14200

C6orf174 3.85 6.01 5.81 158

C6orf176 3.395 5.83 10.75 9.975

C6orf182 410 322 480 465

C6orf185 80.6 125 72.1 104

C6orf186 44.5 9.16 5.1 18.5

C6orf191 2.89 4.66 4.24 3.53

C6orf192 5994.5 4554 2690.5 5186.5

C6orf195 6.8 4.64 4.27 3.54

C6orf201 3.67 5.92 5.48 4.48

C6orf203 2130 1400 2830 1730

C6orf204 39.29 34.7933333333333 13.79 205.433333333333

C6orf208 115 75 30.2 53.2

C6orf211 1791 2831.5 841.5 1724

C6orf217 3.77 5.94 5.42 4.31

C6orf218 2.62 4.25 3.85 3.23

C6orf221 3.61 5.83 5.42 16.7

C6orf222 3.45 4.89 4.51 3.72

C6orf223 10.2 4.78 20 6.29

C6orf225 14.8 8.8 68.4 152

C6orf226 556.5 349.5 2003.5 407

C6orf227 3.68 5.8 22.3 4.59

C6orf25 44.45 39.38 42.55 40.25

C6orf26 90.3 302 254 346

C6orf27 832 606 523 634

C6orf35 366.5 632 159.5 551

C6orf38 6.55 8.37 6.67 9.91

C6orf41 3.39 62 9.4 55.1

C6orf47 658.3 406.5 481.7 423.2

C6orf48 24309.0909090909 34818.1818181818 6650 11478.1818181818

C6orf52 147 66.5 712 77.2

C6orf57 1220 1370 2040 577

C6orf58 15.5 14.7 26.3 12.4

C6orf59 16.1 5.29 13.2 399

C6orf62 11500 11500 4390 10300

C6orf64 696 142 309 898

C6orf70 631 974 608 1120

C6orf72 1059.5 2135 2640 3170

C6orf81 4.64 5.27 16.3 11.2

C6orf89 2308.66666666667 1395 1911 1679.33333333333

C6orf94 10.4 112 10.6 9.51

C6orf97 9.965 7.35 5.55 13.83

C7 45.8 42.5 36.5 35.4

C7orf10 260 286 66.3 90.5

C7orf11 6280 6080 3070 3410

C7orf13 62.2 85 136.35 81.9

C7orf16 4.285 5.385 4.905 4.01

C7orf20 11200 10700 3600 4120

C7orf23 386 450 2030 1260

C7orf25 443 289 436 381

C7orf26 2720 2000 1540 2050

C7orf27 22400 11900 9680 16500

C7orf28A 4462.66666666667 3349.33333333333 3285 3903

C7orf28B 358 377 222 310

C7orf29 6850 3160 25.5 2370

C7orf30 18300 11600 11000 9930

C7orf31 5.54 22.1 4.17 20.9

C7orf33 3.02 8.65 4.39 3.92

C7orf34 31.8 27.6 20.8 34.5

C7orf36 3150 2090 987 2030

C7orf38 275 361 162 319

C7orf4 3.81 6.12 5.59 4.46

C7orf40 2420 9170 9210 9840

C7orf41 290.5 236 647.5 1621

C7orf42 5670 14200 3890 6060

C7orf43 298 618 330 436

C7orf44 2140 3590 4300 4150

C7orf45 4.24 6.28 7.28 5.08

C7orf46 4.30333333333333 50.1666666666667 219.133333333333 59.8666666666667

C7orf47 11900 16600 11400 15600

C7orf49 1610 3420 3850 3510

C7orf50 1680 3200 3490 2080

C7orf51 10.6 6.58 5.02 7.3

C7orf52 3.63 5.81 5.47 4.47

C7orf53 349 418 456 228

C7orf54 59.95 79.45 85.4 65.15

C7orf55 2070 1810 5340 6520

C7orf57 3.96 6.19 5.72 4.65

C7orf58 3.21666666666667 9.94 11.8033333333333 5.1

C7orf59 11500 18800 27900 29800

C7orf60 23.45 158.8 158.2 227.8

C7orf61 45 48.6 58.2 55.4

C7orf62 2.66 4.32 3.92 3.26

C7orf63 3.59 16.1 5.41 27.7

C7orf64 158 217 155 273

C7orf65 13.965 8.245 16.685 6.94

C7orf66 2.75 4.47 4.04 3.37

C7orf68 2670 4440 2680 5380

C7orf69 2.94 4.69 4.33 3.55

C7orf70 2630 1360 1230 1510

C7orf71 20 12.5 4.36 4.88

C7orf72 7.3 56.235 4.405 3.615

C8A 496 176 11.6 16.9

C8B 640 23 5.62 4.93

C8G 2820 83.85 121.5 96.5

C8ORFK29 17.5 13.6 46.3 23.3

C8orf12 23.2 24.9 26.2 52.7

C8orf22 3.825 7.98 4.465 3.665

C8orf23 3.01 4.86 4.42 3.67

C8orf24 6.03 25.2 17.1 3.41

C8orf25 3.67 5.95 5.43 4.3

C8orf26 12.9 20.3 21.9 17.2

C8orf28 2.85 4.65 4.21 3.47

C8orf29 2.87 19.1 6.44 7.48

C8orf30A 2680 2330 1870 2740

C8orf31 2.73 4.42 535 863

C8orf33 569 483 586 1330

C8orf34 10.3 5.06 4.61 21.2

C8orf37 121.85 100.45 152.65 143.5

C8orf38 1067 944.5 1090.5 1111

C8orf39 14.9 20.2 26.1 25.9

C8orf4 325 842 43.5 4.07

C8orf40 944 1400 2770 901

C8orf41 293 295 391 190

C8orf42 111 60.9 72.8 3.76

C8orf44 163 89.4 891 224

C8orf45 11.36 50.65 4.955 4.175

C8orf46 12.1 5.64 5.32 4.2

C8orf47 2730 205 123 1530

C8orf48 88.4 45.75 4.69 84.3

C8orf50 24.5 29.6 23.6 54.6

C8orf51 8.35 17 178 126

C8orf55 2800 485 7760 1140

C8orf56 225 220 361 135

C8orf58 1952 2261.5 2265 1916.5

C8orf59 2523.9 3500.5 3801 5279.5

C8orf60 30.8 56.8 133 66.2

C8orf66 9.42 9.17 23.4 5.71

C8orf67 46.2 65.4 84.4 56.2

C8orf68 4.64 10.7 10 82.8

C8orf71 9.18 10.15 13.1 11.45

C8orf73 207 142 187 156

C8orf74 26.6 16.8 23.8 19.4

C8orf75 8.94 5.59 5.78 9.6

C8orf76 3950 3290 3030 5240

C8orf77 3.63 5.75 5.41 5.67

C8orf78 182 209 235 211

C8orf79 3.61 5.61 5.48 12.1

C8orf80 15.775 21.85 18.35 23.865

C8orf81 8.4 5.94 12.7 10.3

C8orf82 12800 6310 12200 12800

C8orf83 470 787 511 499

C8orf84 3.7 5.38 14.2 8.73

C8orf85 13.4 9.64 20.1 156

C8orf86 3.18 5.18 4.73 3.84

C9 3.7 5.97 5.55 16.7

C9orf100 291 232 245.5 358.5

C9orf102 117.2 141.3 153.466666666667 186.133333333333

C9orf103 1508 546.6 1743 219.4

C9orf106 39.5 34.8 66.4 40.9

C9orf109 29 57 14.5 8.88

C9orf11 18.8 12.3 22.5 8.11

C9orf110 26.7 43.9 9.09 4.78

C9orf114 279 255 354 312

C9orf116 261.85 151.4 690 826

C9orf117 13.9 4.54 16.8 51.2

C9orf119 971 2880 2510 2400

C9orf122 72.5 5.68 1260 523

C9orf123 47.7 23 67.9 209

C9orf125 3980 61.1 4.75 5790

C9orf128 4.04 6.26 5.91 7.54

C9orf129 3.87 6.07 5.67 4.62

C9orf130 64.0466666666667 121.216666666667 402.833333333333 253.133333333333

C9orf131 49.9 42.4 31.6 31.8

C9orf135 3.82 6.13 5.6 4.47

C9orf139 13.5 15 11.1 15.7

C9orf140 2570 1300 2610 1030

C9orf142 1080 2460 2250 4090

C9orf144 3.68333333333333 5.81666666666667 5.41333333333333 4.45333333333333

C9orf144B 6.02 14.7 8.82 8.5

C9orf148 3.74 5.98 9.23 17.9

C9orf150 289 237 196 19

C9orf152 3.84 6.13 60.2 13.8

C9orf153 305 393 345 355

C9orf156 353 350 349.5 652.5

C9orf16 292 301 902 567

C9orf163 1210 1250 1480 1150

C9orf167 1950 2980 2560 93.8

C9orf169 75.75 37.55 138.5 242

C9orf170 9.77 171 14.1 14.9

C9orf171 2.79 4.53 4.11 210

C9orf172 2.66 4.38 6.3 3.77

C9orf173 563 584.5 508.5 519

C9orf21 487 872 1580 993

C9orf23 5670 4450 13100 25100

C9orf24 268 37.9 45.9 33.3

C9orf25 715.45 704.7 636.6 775

C9orf27 3.289 9.47 5.196 3.974

C9orf3 1110 1310 983 380

C9orf30 1110 2140 1400 952

C9orf37 111 213 125 468

C9orf38 23.2 12.4 31.4 25.5

C9orf4 91.8 5.76 9.45 55.6

C9orf40 830.5 451 1890 2135

C9orf41 75.1 190 89.5 158

C9orf43 40.5 46.8 76.2 17.6

C9orf44 11.8 12 10.3 23.4

C9orf45 94.7 104 32.9 110

C9orf46 610 734 1520 1460

C9orf47 16.15 28.3933333333333 46.3133333333333 40.7133333333333

C9orf5 1265 2520 1966.5 1159

C9orf50 41.5 47.9 74.2 56.8

C9orf53 25 30.9 27.1 31.1

C9orf57 2.97 4.84 4.4 23.8

C9orf6 809 2110 1090 1730

C9orf64 547 457 443 425

C9orf66 18.8 11.4 4.35 18.3

C9orf68 150 57.4 4.78 166

C9orf69 1200 651 1030 2680

C9orf7 8030 5700 2920 3100

C9orf70 5.15 6.1 6.07 4.62

C9orf71 57.6 116 123 50.9

C9orf72 12.4 17.5 28.4 57.9

C9orf73 3.42 4.15 3.77 10.6

C9orf75 26100 10600 9920 9340

C9orf78 1870 2830 2790 4150

C9orf79 3.63 4.5 4.1 4.67

C9orf80 864.5 1335 848.5 1325

C9orf82 480 280 293 865

C9orf84 2.94 4.78 4.34 3.57

C9orf85 279.5 343.5 496 568.5

C9orf86 165 163 502 578

C9orf89 1780 4550 13700 6000

C9orf9 22.2 47.5 662 309

C9orf91 394 194 392 885

C9orf93 13.55 5.945 31.1 55.65

C9orf95 227.5 208.5 2560 558

C9orf96 24.4 20.6 35.2 20.6

C9orf98 12.9 16.9 41.5 62.2

CA1 10.2 16.6 27.6 30

CA10 2.7 4.38 3.97 3.3

CA11 9 56 392 309

CA12 1520 204 5070 161

CA13 91.9 6.49 590 522

CA14 446 81.9 85 196

CA2 1310 5270 28000 32500

CA3 3.86 13.1 19.2 14.6

CA4 6.48 6.3 42.6 5.13

CA5A 67.5 226 60.4 43.9

CA5B 172 277 88.2 128

CA5BP 140.5 274.5 149.5 269

CA6 176.91 171.3 127.585 136.72

CA7 8.15 13.1 13.1 14.4

CA8 10 19.6 11.2 4310

CA9 64 27 3.99 3.33

CAB39 1353 1003.5 1517.5 830.5

CAB39L 63.5333333333333 146.566666666667 101.133333333333 96.6

CABC1 2530 661 529 2250

CABIN1 533.4 576.95 413.25 874.5

CABLES1 2860 2140 2450 6810

CABLES2 170 259 167 246

CABP1 2.55 4.13 73.5 28.8

CABP2 3.75 5.93 5.49 4.43

CABP4 69.9 86.6 81.3 77.7

CABP5 366 263 299 325

CABP7 3.56 5.75 5.26 23

CABYR 569.95 646 1338.95 870.3

CACHD1 64.5 119 176 345

CACNA1A 3.06 10.5 5.72 6.25

CACNA1B 19.8 21.65 26.5 46.65

CACNA1C 198.0675 228.3 135.8425 257.2575

CACNA1D 19.5 6.04 62.5 11.6

CACNA1E 13.5 20.2 34.3 13.6

CACNA1F 20.2 12.8 24.3 14.3

CACNA1G 9.97 76.725 33.165 20.57

CACNA1H 4200 2020 14.5 764

CACNA1I 33.245 27.835 33.18 32.95

CACNA1S 19.8 12.6 37.5 20.2

CACNA2D1 12.835 50.5 68.4 132

CACNA2D2 8.25 12.6 17.3 238

CACNA2D3 12.17 9.18 14.09 149.55

CACNA2D4 3.38 18.7 5.04 4.12

CACNB1 13.5 18.5 22.8 22

CACNB2 45.3 12.65 7.895 123.85

CACNB3 32.2 134 513 217

CACNB4 2.66 4.31 8.79 199

CACNG1 14 11.1 10.4 17.3

CACNG2 5.74 5.46 4.99 3.97

CACNG3 37.3 36.6 36.8 37

CACNG4 48.45 37.95 18.64 12.76

CACNG5 17 9.25 15.3 17.8

CACNG6 3.58 19.6 7.93 57.3

CACNG7 11.875 14.65 16.79 41.2

CACNG8 8.1 9.13 11 21.3

CACYBP 7612.33333333333 5459 8467.66666666667 8614.33333333333

CAD 2770 1430 3900 5480

CADM1 3.17 2011 422.8 2683.5

CADM2 5.39 4.835 4.42 4.56

CADM3 19 4.8 14.1 7.9

CADM4 46.4 116 14.3 31.7

CADPS 21.895 10.785 4.74 3.75

CADPS2 23 40.7 33.3 115

CAGE1 17.8 18 4.85 5.18

CALB1 3.85 5.58 21900 4.88

CALB2 2.63 4.27 994 3.23

CALCA 9.5 7.98333333333333 6.77333333333333 1448.21666666667

CALCOCO1 94.3 172 137 338

CALCOCO2 280 360 75.7 289

CALCR 10.035 9.4 8.38 4.54

CALCRL 7.81666666666667 9.82666666666667 28.7 24.8733333333333

CALD1 13000 24300 1820 7040

CALHM1 27.8 22.7 16.9 21.1

CALHM2 3.33 10.9 4.94 55.8

CALHM3 8.54 36.3 12.5 34.5

CALM1 448 878 1590 679

CALM2 30500 38150 30650 21200

CALM3 3250 5415 5965 4425

CALML3 624 889 806 608

CALML4 612.05 618.55 486.05 905.9

CALML5 33.2 36.6 34.9 49.9

CALML6 12.1 19.7 28.7 28.5

CALN1 8.68 5.97 5.44 4.32

CALR 99200 84900 79000 90400

CALR3 12.2 24.6 9.58 20.6

CALU 1539 3771 2764 2224

CALY 29598.85 29837.55 26240.75 29447.25

CAMK1 328 632 260 359

CAMK1D 6.58 6.09 6.08 268.65

CAMK1G 3.45 5.56 5.16 4.97

CAMK2A 3.12 5.055 7.025 11.595

CAMK2B 6.29 5.52 8.4 40.8

CAMK2D 888 917.5 173.45 300.5

CAMK2G 792.55 1919.5 1674 1519

CAMK2N1 778.7 4266 4190 368

CAMK2N2 405 638 1790 343

CAMK4 5.55 22.6 14.4 103

CAMKK1 12.35 28.135 33.3 202.9

CAMKK2 666 506 382 604

CAMKV 6.69 9.98 10.885 121.25

CAMLG 10.2 34.4 22.1 124

CAMP 2.65 4.3 3.89 3.26

CAMSAP1 144 211 155 248.5

CAMSAP1L1 686.5 1127 1147 1210

CAMTA1 63.5 4.86 17.885 46.355

CAMTA2 95.2 55.5 114 165

CAND1 2160 2450 1890 2100

CAND2 378 687 5.89 2970

CANT1 398.5 486 497 499

CANX 1277 1526.5 1229 1750.5

CAP1 1725.5 2655 2250 1683

CAP2 505 514 334 187

CAPG 1272.5 1892 865.5 118.4

CAPN1 919 671 1030 399

CAPN10 448.066666666667 641.966666666667 526.666666666667 556.733333333333

CAPN11 2.77 4.47 4.06 3.39

CAPN12 36.1 173 93.7 12.1

CAPN13 11.09 14.82 12.865 11.26

CAPN14 3.735 5.9 5.485 4.505

CAPN2 5580 7230 7110 14000

CAPN3 52.7 24.5 16.1 49.6

CAPN5 251 1000 478 379

CAPN6 19.1 96.7 57.7 153

CAPN7 345 453 289 300

CAPN8 3.45 5.52 5.1 4.15

CAPN9 2.94 12 11.5 13.2

CAPNS1 5510 9900 20900 5600

CAPNS2 283 513 892 261

CAPRIN1 403.5 283 365 571

CAPRIN2 246.4 312.5 778 465.5

CAPS 58.4 22 36.9 34.8

CAPS2 24.5 95.7 87.4 270

CAPSL 9.21 14 5.45 14.6

CAPZA1 5940 9090 6090 9650

CAPZA2 1550.5 2100 2985 1143

CAPZA3 2.97 4.68 4.32 7.42

CAPZB 2890 3650 3345 1795

CARD10 10700 12800 15100 3240

CARD11 22.35 20.65 18 30.4

CARD14 191.9 199 214.1 178.45

CARD16 5.41 241 4.71 3.78

CARD17 4.19 107 7.28 5.8

CARD18 3.62 319 5.35 4.26

CARD6 6.79 1174.5 15.61 285.1

CARD8 718 846 1010 981

CARD9 215.65 41.2 35.9 99.25

CARHSP1 3850 3150 2650 2000

CARKD 1171.5 514.45 812 687.75

CARM1 6890 3180 8180 10400

CARS 550 460 320 617

CARS2 20000 10700 10900 16800

CARTPT 4.46 4.57 8.99 3.43

CASC1 3.495 5.57 5.225 10.185

CASC2 6.48333333333333 8.75666666666667 65.1333333333333 64.1666666666667

CASC3 3600 5810 4790 10600

CASC4 1172 1552.15 1478.71 1063.695

CASC5 1692.18181818182 1800.17272727273 1300.00909090909 1627.07272727273

CASD1 133.05 277.5 105.2 110.45

CASK 726 1655 396.5 1343.5

CASKIN1 8595 8934.5 8460 9310

CASKIN2 328 369 877 326

CASP1 3.22 5.85 4.8 3.92

CASP10 419.73 411.715 2280.7025 493.2275

CASP12 3.53 5.69 5.29 4.36

CASP14 12.1 17.3 18.1 19

CASP2 691.5 1019 1595 1447

CASP3 9322.27272727273 4861.90909090909 1904.54545454545 4992.63636363636

CASP4 12500 10900 70.5 16.9

CASP5 3100 2530 21.5 32.7

CASP6 3800 3630 816 1900

CASP7 1850 4210 2360 3300

CASP8 176.25 164.25 194.05 69.35

CASP8AP2 497 337 552 587

CASP9 620 900 609 837

CASQ1 42.2 34.7 41.2 43.7

CASQ2 3.71 5.69 5.58 4.78

CASR 3.79 19.5 5.7 13.2

CASS4 45.45 42.6 55.2 49.8

CAST 3840 5055 2665 4480

CASZ1 138.6 59.05 60.55 93.7

CAT 3370 1788 1729 944.2

CATSPER1 2.54 4.11 17.3 3.13

CATSPER2 44.5 29.75 123.15 223.5

CATSPER3 66.8 59.1 107 70.4

CATSPER4 3.54 5.28 4.83 9.17

CATSPERB 39.3 14.5 3.92 8.28

CATSPERG 292 292 252 259

CAV1 292 1428.18181818182 5179.09090909091 453.363636363636

CAV2 349.872727272727 744 420.481818181818 200

CAV3 39.6 147 251 51.4

CBARA1 956 1320 1510 1150

CBFA2T2 223 363 428.5 294.5

CBFA2T3 36.3 5.44 21.6 41.8

CBFB 2112 2502 3253 3718

CBL 385 581 1420 831

CBLB 1019.9 507.9 151.65 249.25

CBLC 228 8.36 4.39 52.5

CBLL1 510 733 675 549

CBLN1 3.5 5.59 5.17 4.2

CBLN2 93.9 4.61 4.17 3.72

CBLN3 66.8 22.5 47.4 81.9

CBLN4 3.27 5.32 4.88 3.95

CBR1 1340 4100 9.55 7620

CBR3 10.3 10.5 137 251

CBR4 2300 1110 346 2960

CBS 85600 28100 47500 67800

CBWD5 7136.22 4323.48 4932.64 6756.2

CBX1 8050 9473.33333333333 7900 8993.33333333333

CBX2 2833.85 3074.6 1275.25 6636.15

CBX3 34962.5 21143.5 20724.5 21833.5

CBX4 525 396 465 299

CBX5 1840 2350 3745 1910

CBX6 3960 21700 20100 29300

CBX7 200.05 462.5 882.5 503

CBX8 306 365 253 503

CBY1 354 358 376 509

CBY3 3.23 5.2 4.77 3.92

CC2D1A 422.8 193.35 555.35 813

CC2D1B 327.9825 374.795 341.6975 414.475

CC2D2A 248 182.533333333333 60.9333333333333 365

CC2D2B 5.32 4.83 7.25 9.3

CCAR1 2493 3290 2798 4246

CCBE1 3.37 5.32 4.99 14.7

CCBL1 329 552 304 448

CCBL2 751.5 357.5 713 852

CCBP2 8.92 6.27 14.3 7.97

CCDC101 789 876 1140 1130

CCDC102A 275 474 1240 1110

CCDC102B 12.05 12.36 22.5 6.365

CCDC103 24.595 35.55 173.4 229.7

CCDC104 943.5 1248 2365 4140

CCDC105 2.82 4.58 4.15 3.46

CCDC106 3160 5300 2990 5340

CCDC107 1605 2675 2245 2200

CCDC108 5.76 6.89666666666667 6.48333333333333 7.10666666666667

CCDC109A 369 503 509 261

CCDC109B 439 477 1410 2030

CCDC11 6.07 23.4 34.4 17.8

CCDC110 3.23 14.3 4.72 24.4

CCDC111 265 255 136 290

CCDC112 150 71.2 339 361

CCDC113 3.74 5.74 70.2 64.4

CCDC114 5.895 14.43 21.4 18.9

CCDC115 1396.5 863.5 1696.5 1481.5

CCDC116 10.9 75.2 137 28

CCDC117 3340 4510 1980 5060

CCDC12 3820 7410 5990 7400

CCDC120 921 1720 643 1160

CCDC121 82.9666666666667 90.9866666666667 93.8766666666667 163.8

CCDC122 59.2 115 142 437

CCDC123 128 269 437 162

CCDC124 1570 1670 2820 5540

CCDC125 1739 1374.33333333333 1495.66666666667 1718.33333333333

CCDC126 318 227 98.5 152

CCDC127 1520 1730 3620 2070

CCDC129 4.13 6.36 6.01 4.97

CCDC13 3.86 5.59 5.09 22.4

CCDC130 3669 2031 2652 4326

CCDC132 60.1 59.9 118 125

CCDC134 150 122.4 202 193

CCDC135 5.13 11.1 6.56 3.78

CCDC136 20.24 70.285 238.9 1140

CCDC137 3730 4570 7030 6320

CCDC138 326 449 623 735

CCDC14 1792 2476.5 2462 3505

CCDC140 3.28 5.27 4.86 38.7

CCDC141 16.2 9.75 36.4 3.81

CCDC142 788 881 532 1320

CCDC144A 230.735 7.515 17.005 31.195

CCDC144B 222 4.45 12.9 30.3

CCDC144NL 3.88 6.18 5.85 4.89

CCDC146 30.3 10.9 105 149

CCDC147 3.275 6.495 8.28 8.715

CCDC148 37.85 24.4 38.05 9.72

CCDC149 496 239.8 179.3 12.4766666666667

CCDC15 78.8 16.9 149 146

CCDC150 648 385 246 456

CCDC151 11.8 18 28.8 82.1

CCDC152 3.51 7.44 5.19 4.16

CCDC153 13.6 117 213 175

CCDC154 249 186 365 259

CCDC155 11.4 18.4 13.5 10.7

CCDC157 13.4 16.8 22.3 26.9

CCDC158 9.435 22.8 7.97 9.92

CCDC159 21 21.9 21.6 29.1

CCDC160 4 9.05 98 52.2

CCDC17 23.2 13.7 5.48 36

CCDC18 251.3 318 528.7 889.35

CCDC19 85.5 15.9 54.1 77

CCDC21 262 398 512 422

CCDC22 468 1200 487 1460

CCDC23 981 1148.5 1955 2690

CCDC24 21 53.4 93.3 52.5

CCDC25 329 750 548 687

CCDC26 3.46 5.57 5.16 4.08

CCDC27 8.51 4.48 7.07 7.69

CCDC28A 4020 2120 1660 5320

CCDC28B 308 363 144 972

CCDC29 24.1 33.8 21.5 82.5

CCDC3 2.96 4.83 4.38 5230

CCDC30 2.65 4.29 8.18 19.7

CCDC33 68.35 71.4 57.6 74.85

CCDC34 1040.76666666667 3111.46666666667 1698.7 1633.46666666667

CCDC36 2.81 4.57 4.14 3.44

CCDC37 2.85 4.63 4.19 3.47

CCDC38 4.79 8.83 12.8 9.23

CCDC39 2.67 4.33 3.92 5.98

CCDC40 25.51 18 50.135 44.03

CCDC41 277.5 480 929 725.5

CCDC42 6.88 4.56 10.6 10.9

CCDC42B 3.84 5.13 15.4 15.7

CCDC43 392 373 445 721

CCDC45 2735 1790 2475 3750

CCDC46 5.3 74 23.1 296

CCDC47 2360 2240 2640 2620

CCDC48 5.44 14.31 6.29 5.935

CCDC49 371 373 857 578

CCDC50 5470 3240 9040 909

CCDC51 1330 3760 4680 3910

CCDC52 11.9 12.6 14.9 30.8

CCDC53 623 1290 1100 1440

CCDC54 4.739 11.475 10.385 10.925

CCDC55 718.5 1571 1291.5 851.5

CCDC56 8450 9810 9350 14900

CCDC57 279 217 2160 461

CCDC58 2390 4730 3870 2650

CCDC59 2792.72727272727 2980.90909090909 4318.18181818182 5317.27272727273

CCDC6 4538 6746.5 7289 6266

CCDC60 5.9 7.32 12.3 8.98

CCDC61 323 417 412 271

CCDC62 4.28 4.33 5.13 13.4

CCDC63 3.81 5.96 5.74 4.84

CCDC64 35.6 56 12.2 20.1

CCDC64B 101 66.1 92.8 297

CCDC65 6.86 5.01 4.56 16.2

CCDC66 82.2 125 147 352

CCDC67 3.56 5.69 5.36 4.42

CCDC68 318 639 538 24.2

CCDC69 137.35 247.5 114.75 168.45

CCDC7 11.455 4.35 68.6 18.95

CCDC70 3 4.85 4.41 3.63

CCDC71 175 557 240 583

CCDC72 5190 17200 12200 12000

CCDC73 26.55 5.74 5.355 80.57

CCDC74B 8.64 47.75 601.5 582

CCDC75 592.5 800.5 865.5 1685

CCDC76 327 643 321 1310

CCDC77 3090 3680 2100 3460

CCDC78 4380 4970 4120 4530

CCDC79 3.28 5.35 4.89 3.95

CCDC8 37.8 106.15 1320.5 1725

CCDC80 18.97 5.885 59.5 67.65

CCDC81 11.7 7.37 20.3 10.1

CCDC82 562 710 1180 848

CCDC83 3.64 5.63 5.51 4.81

CCDC84 2980 3330 7310 4150

CCDC85A 3.585 5.665 11.58 4.62

CCDC85B 867 1150 615 2240

CCDC85C 2619.85 1704 1778.7 2907

CCDC86 33000 51900 52800 50400

CCDC87 17.3 28.6 17.8 27.7

CCDC88A 614 1590 762 1040

CCDC88B 1374.5 293.45 950.5 339.3

CCDC88C 854.166666666667 284.933333333333 295.133333333333 379.7

CCDC89 3.22 5.19 4.78 135

CCDC9 34700 32000 23100 19500

CCDC90A 6500 6635 9555 9620

CCDC90B 3090 3360 7650 5280

CCDC91 894 662 1020 1060

CCDC92 1110 1120 692 991

CCDC93 1440 1220 2090 1180

CCDC94 12500 3710 4350 10100

CCDC96 138 207 170 323

CCDC97 5010 7430 6640 10100

CCDC99 691.545454545455 1882.72727272727 1790.90909090909 2527.27272727273

CCHCR1 652 574 909 863

CCIN 90.8 103 102 146

CCK 3.05 4.86 4.45 3.66

CCKAR 4.02 8.09 6.75 4.72

CCKBR 3.62 5.87 127 58.1

CCL1 16 9.62 9.35 15

CCL11 3.25 8.98 5.91 4.42

CCL13 3.12 4.96 4.59 3.77

CCL14 223 60.9 5.03 4.03

CCL15 1740 483 61.4 71.6

CCL16 397.515 365.345 431.23 306.5

CCL17 18 15.1 25 22.9

CCL18 2.89 4.67 4.24 3.5

CCL19 16.15 18.65 13.55 17.9

CCL2 6.628 5.645 6.091 12.688

CCL20 16100 10800 5.07 3.14

CCL21 11.04 9.635 12.845 9.96

CCL22 3.48 5.55 5.17 4.19

CCL23 3.62 5.78 5.4 4.27

CCL24 5030 4410 4300 6570

CCL25 5.935 5.485 9.58 11.475

CCL26 4.79 10.9 9.39 16.1

CCL27 12.3 44.3 73.2 129

CCL28 2.58 15.3 42.1 3.18

CCL3 3.65 7.26 8.39 4.53

CCL3L3 19.8 19.995 20.6 20.85

CCL4 12.07 8.99 10.64 11.72

CCL4L1 15.1 11.5 14.7 16.5

CCL5 3.76 5.96 10.7 4.45

CCL7 10.3 13.6 4.55 3.69

CCL8 4.34 4.55 4.16 3.47

CCM2 3110 2500 2180 799

CCNA1 4.14 6.32 2700 4.96

CCNA2 2648 2239 1876 2858

CCNB1 9594.54545454545 11586.3636363636 22074.5454545455 13558.1818181818

CCNB1IP1 7520 4480 3290 5620

CCNB2 19700 14500 20300 13500

CCNB3 25.2 3880 740 220

CCNC 1850 1680 1300 2190

CCND1 1074 2564 2485 780.1

CCND2 2.64 4.28 3.87 4140

CCND3 779 512 1840 1520

CCNDBP1 850.1 1033.8 1018.5 1039.7

CCNE1 8690 9730 12500 7070

CCNE2 730.5 303.3 882 514

CCNF 154 154 271 239

CCNG1 724 746 1275.5 1895

CCNG2 53.2666666666667 19.9066666666667 52.1666666666667 83.2333333333333

CCNH 4380 4480 10700 6040

CCNI 14900 22000 18600 26500

CCNI2 5.34 45.9 5.19 34.7

CCNJ 1100 1340 410 1720

CCNJL 548 495 653 73.4

CCNK 1086 1515 987.5 2350

CCNL1 1495 661.5 816 911.5

CCNL2 1034.66666666667 587.666666666667 2016.66666666667 910

CCNO 168 30.2 3130 496

CCNT1 60.8 64.5 86.7 110

CCNT2 278 212 121 259

CCNY 836 626 1050 533

CCNYL1 472 463 428 163

CCNYL3 3.33 5.3 4.87 4.39

CCPG1 194 180 130 94.9

CCR1 3.56 262 5.3 4.3

CCR10 143 238 171 304

CCR2 2.86 4.63 5.68 3.48

CCR3 9.16 21.9 5.66 4.6

CCR4 5.68 7.95 6.15 4.55

CCR5 90.01 94.78 67.48 72.63

CCR6 953 2460 489 543

CCR7 3.59 24.4 48.7 29.1

CCR8 3.47 5.6 5.13 4.11

CCR9 3.63 5.7 5.51 4.65

CCRK 68.3 105.45 611.5 1012

CCRL1 5.66 4.49 6.35 4.22

CCRL2 3.615 57.775 5.415 4.495

CCRN4L 33.5 82.7 51.4 56.3

CCS 1680 3000 768 1060

CCT2 52320 89090 78710 91720

CCT3 75200 65800 50100 93300

CCT4 84963.6363636364 93081.8181818182 79463.6363636364 144872.727272727

CCT5 214 353 357 241

CCT6A 54800 42400 37700 46300

CCT6B 56.4 20.5 193 35.3

CCT7 27690 39420 31270 56050

CCT8 21700 25500 29700 30300

CCT8L2 3.54 5.72 5.31 4.35

CD101 3.45 5.6 5.13 13.3

CD109 419 873 4.44 837

CD14 250 187 72.2 110

CD151 43100 46700 40100 31600

CD160 3.17 5.15 4.71 3.78

CD163 6.04 12.6 11.7 9.93

CD163L1 9.25 13.9 6.77 16.6

CD164 3880 3620 3630 3645

CD164L2 234 221 193 234

CD177 10.41 12.785 15.055 5.965

CD180 2.78 4.51 4.09 3.38

CD19 22.18 130.4 22.09 343.9

CD1A 5.49 5.97 5.63 4.64

CD1B 8.13 15.2 17.4 11.6

CD1C 3.21 5.22 4.77 3.91

CD1D 3.83 5.94 5.77 4.95

CD1E 2.96 4.71 4.36 3.57

CD2 37.75 35.55 40.5 29.4

CD200 5.32818181818182 6.93454545454545 7.44363636363636 6.57545454545455

CD200R1 10.655 5.57 9.41 12.36

CD207 3.48 6.63 13.1 8.31

CD209 21.29 27.9366666666667 33.2266666666667 36.4

CD22 3.54 4.64 4.26 3.51

CD226 8.79 6.16 5.63 4.5

CD24 44500 28400 111000 106

CD244 32 14.4 100.1 103.68

CD247 3.54 15.4 5.32 4.32

CD248 12.5 16.6 15 12.9

CD27 40.6 40.6 65.5 44.6

CD274 12.9 7.675 4.19 4.365

CD276 7905.5 12821.5 5536 10475.5

CD28 27.1 26.04 19.415 15.335

CD2AP 424 555 380 610

CD2BP2 800 491 642.5 1197

CD300A 61.865 52.055 45.3 49.605

CD300C 3.99 5.93 6.81 7.3

CD300E 365.9 395.395 460.24 413.6

CD300LB 79.1 75.2 78.8 78.6

CD300LD 4.06 6.26 5.89 4.85

CD300LF 3.64 5.81 5.48 4.48

CD300LG 4.19 4.555 10.295 7.835

CD302 371.5 1980 4.475 788.5

CD320 7040 3250 7830 5860

CD33 9.88 6.16 13.2 39.8

CD34 9.775 7.415 13.2 15.75

CD36 26.15 233 20.623 6.713

CD37 3.19 11.1 14 5.82

CD38 3.39 79.2 5.03 4.11

CD3D 37.6 1490 97.9 46.8

CD3E 34.8 33.6 79.4 38.7

CD3EAP 2870 4150 4380 5420

CD3G 9.13 9.62 6.55 7.91

CD4 8320 8680 8330 7580

CD40 8.23818181818182 11.5809090909091 95.5 67.1909090909091

CD40LG 3.97 6.2 5.83 4.79

CD44 42.1 1150 9410 119

CD46 427 387 360 354

CD47 4180 2010 5670 4940

CD48 3.45 5.57 5.16 4.19

CD5 5.02 6.3 6.04 5.2

CD52 9.39 5.44 55.3 16.8

CD53 2.95 4.8 4.36 3.6

CD55 285 327 317 1340

CD58 279.5 424 491.5 544.5

CD59 232 87.1 160 191

CD5L 5.28 10.6 6.51 13.5

CD6 3.4 14.3 5.08 10.8

CD63 34300 56400 49400 25200

CD68 330 313 84.2 44.2

CD69 3.94272727272727 5.39272727272727 5.00636363636364 4.75272727272727

CD7 728 393 15 13.4

CD70 15.8 19 24.1 733

CD72 2.95 4.72 8.73 10.9

CD74 18.1 5.83 9.4 4.38

CD79A 3040 2530 3050 1980

CD79B 6.45 8.17 16.8 11.4

CD80 11.7 9.03 8.95 10.8

CD81 8325 7085 12400 18650

CD82 58.7 65.9 159 257

CD83 616 83.8 978 671

CD84 3.165 5.02 4.725 20.84

CD86 319 965 155 231

CD8A 76 141 65.85 161.5

CD8B 8.31 1958.33333333333 9.90666666666667 9.00666666666667

CD9 2.59 723 4460 1200

CD93 3.93 5.59 5.15 4.2

CD96 3.87 27.8 5.52 4.66

CD97 2150 1710 5460 1550

CD99 944 1530 763 3510

CD99L2 884 3250 2350 10400

CDA 35.9 69.2 51.3 74.8

CDADC1 90.5 276.5 379.35 435

CDAN1 290 371 269 228

CDC10L 758 734 784 605

CDC123 4810 4460 8630 10100

CDC14A 9.81 38.5 20.5 24

CDC14B 176.8 183.75 183 276.5

CDC14C 343 360.666666666667 341.766666666667 374.666666666667

CDC16 13400 5830 7060 9670

CDC2 6378 9754 8514 6645

CDC20 2330 3800 4870 2840

CDC20B 20.3 5.96 5.32 7.69

CDC23 2180 3340 4900 6530

CDC25A 1270 3570 2370 3960

CDC25B 2740 4620 7950 2200

CDC25C 851 627 686 903

CDC26 4100 6610 6230 6590

CDC27 1565.5 1788.5 2099.5 2296

CDC2L1 4553.33333333333 2313.33333333333 2860 2376.66666666667

CDC2L2 17800 9640 10900 8940

CDC2L5 1103.33333333333 490.4 725.333333333333 788.333333333333

CDC2L6 2470 1430 2720 1840

CDC34 6430 4880 3500 4920

CDC37 6890 3940 8650 10100

CDC37L1 594.5 403 653.5 389

CDC40 1640 1280 1585 2010

CDC42 6554.5 6435.06666666667 8234.96666666667 5568.66666666667

CDC42BPA 527 352 604 536

CDC42BPB 1980 3600 4750 3660

CDC42BPG 171 12 18.7 26

CDC42EP1 57700 23500 11100 20400

CDC42EP2 220 1600 1050 972

CDC42EP3 3.52 5.7 5.26 10.5

CDC42EP4 575 749 1870 325

CDC42EP5 40300 42500 41000 40500

CDC42SE1 1030 515 730 900

CDC42SE2 4010 4980 9190 2830

CDC45L 7520 17000 9250 21200

CDC5L 1716.32 1319.31 1556.935 3224.3

CDC6 253 351 146 265

CDC7 921 2270 2180 7440

CDC73 329 224 168 225

CDCA2 3360 6260 8700 4360

CDCA3 2520 3870 8980 4310

CDCA4 376 411 580 700

CDCA5 11500 10800 10710 14760

CDCA7 2137.5 3645.5 5664.5 5661.5

CDCA7L 2586.5 1548 1705 1325.5

CDCA8 9580 14100 7860 13300

CDCP1 3.06090909090909 29.6272727272727 6.84454545454545 3.68545454545455

CDCP2 139 163 182 153

CDGAP 2.69 4.37 29.8 41.1

CDH1 1704 1821 80.93 188.5

CDH10 34.2 5.63 8.63 74

CDH11 2.63 4.26 3.86 5.73

CDH12 2590 1640 11.3 370

CDH13 4.16 6.35 6 4.99

CDH15 41.4 38.9 22.1 24.6

CDH16 7.74 4.5 11300 3.67

CDH17 6.085 360.6 4.697 5.112

CDH18 6.25 5.06 4.59 45.3

CDH19 2.98 293 4.37 3.6

CDH2 1203 6555 5285 3320

CDH20 2.95 4.77 4.34 3.61

CDH22 6070 7270 5940 6470

CDH23 8.46333333333333 22.4666666666667 13.6666666666667 29.8333333333333

CDH24 173 314 101 165

CDH26 7.86 5.14 24.8 38.8

CDH29 12.91 9.17 8.255 7.07

CDH3 30.8 30.5 31.6 84

CDH4 3.38 5.43 5.01 4.09

CDH5 98.3 116 158 113

CDH6 2000 103 29.5 3.8

CDH7 34.5 24.9 30.6 24.9

CDH8 3.23 5.28 4.81 3.89

CDH9 10.5 39.9 11.5 7.79

CDIPT 552 530 968 1010

CDK10 2432.5 1836.5 2540 2301

CDK2 222.7 247.5 403.4 474.6

CDK2AP1 14600 14500 43400 17800

CDK2AP2 44100 31100 21500 12600

CDK3 729 785 559 1020

CDK4 11400 14700 15000 19500

CDK5 2370 2610 3580 6690

CDK5R1 633 592 499 936

CDK5R2 135 150 150 160

CDK5RAP1 4337.5 5374.5 3421.5 4504

CDK5RAP2 1953 2528 1786.5 1548

CDK5RAP3 642 613 1580 1410

CDK6 20900 7220 2810 6710

CDK7 3350 3230 5290 3590

CDK8 280 329.5 254 383

CDK9 1195 1436 1450 1732

CDKAL1 673.5 270 46.4 422.5

CDKL1 71.35 45.05 303.5 260

CDKL2 3.54 5.72 5.31 40.4

CDKL3 105.57 138.4 321.6 144.8

CDKL4 11.105 7.795 10.415 6.3

CDKL5 637 1990 102 551

CDKN1A 8.05 36.1 16.8 26.8

CDKN1B 1176.45454545455 1698.63636363636 2550 1257.09090909091

CDKN1C 19800 396 421 1000

CDKN2A 17600 6210 5880 22805

CDKN2AIP 736 562 247 843

CDKN2AIPNL 989.5 2165 3580 2135

CDKN2B 20.9 89.5 11.7 30.4

CDKN2BAS 22.6 12.5 5.83 4.85

CDKN2C 259 163 1970 5990

CDKN2D 1260 804 3540 1420

CDKN3 2760 4260 6650 2695

CDNF 7.1 21.8 21.7 28.8

CDO1 267 704 5.68 1320

CDON 326 171 187 656

CDR1 2.61 8.15 3.83 10.2

CDR2 1760 1140 1030 1140

CDR2L 927.9 1366.3 7751 1218.15

CDRT1 14.59 35.95 22.75 18.35

CDRT15 2.66 4.29 3.9 3.27

CDRT15L1 6.8 5.74 9.62 8.17

CDRT15P 3.03 4.85 4.45 3.68

CDRT3 353 326 274 266

CDRT4 15.8 94.4 15.8 60.6

CDRT7 10.8 4.54 4.14 3.42

CDRT8 13.6 12.1 8.31 13.1

CDS1 188.5 66.5 742 852

CDS2 985 2340 1410 991

CDSN 14.6 23.5 16.3 12

CDT1 6290 6700 7590 7770

CDV3 10960 11375 11390 9305

CDX1 262.35 264.7 280.1 208.25

CDX2 160 1220 5.99 4370

CDX4 9.495 5.058 8.528 6.021

CDY2A 3.37 5.45 4.99 4.1

CDYL 1110 549 1160 729

CDYL2 5.35 64.8 3.81 23.8

CEACAM1 3622 2221.5 49.85 16.35

CEACAM16 4.94 7.51 5.76 4.7

CEACAM18 9.03 9.04 7.29 6.42

CEACAM19 1440 1420 917 1090

CEACAM20 910 1530 1590 767

CEACAM21 3.33 5.44 4.95 7.61

CEACAM3 119.5 68.2 4.65 3.82

CEACAM4 23.4 26 17.9 13.4

CEACAM5 3.71 6 10.3 4.56

CEACAM6 3.47 26.5 5.18 4.2

CEACAM7 9.66 13.52 17.075 13

CEACAM8 3.36 5.73 32 17.5

CEBPA 18900 31000 77.4 1200

CEBPB 3523 6214 4457 2695

CEBPD 14500 18000 16500 16400

CEBPE 7.58 10.2 10.2 14.1

CEBPG 6900 11000 2380 5820

CEBPZ 2931 2852 3078 5708

CECR1 14.9 15.3 20.5 13.3

CECR2 129.153333333333 523.7 170.686666666667 430.9

CECR3 50.1 50.8 53.5 42.2

CECR4 146 87 123 118

CECR5 2160 6560 2440 5410

CECR6 13.6 30 74.9 131

CECR7 2.85 13.5 4.23 3.58

CECR9 12.7 18.2 15.6 4.92

CEL 187 150.75 142.5 134.65

CELA1 3.78 5.83 5.7 4.96

CELA2A 50.5 37.4 41.3 55.5

CELA2B 36.3 44.1 44.1 54.3

CELA3A 58.75 22.65 56.8 19.65

CELA3B 33.97 12.215 33.6 3.9

CELSR1 14648.6333333333 17283.2333333333 16861.3666666667 16081.8666666667

CELSR2 123 203 687 370

CELSR3 197 83.2 49.7 194

CEMP1 14.5 9.09 43.5 16.3

CEND1 156 157 223 96.4

CENPA 1180 1160 2400 1690

CENPB 721 1450 1200 1180

CENPBD1 834.5 1615 872.5 970.5

CENPC1 1360 894 716 1070

CENPE 2023 5060 781.3 4143

CENPF 14535 6505 13930 11175

CENPH 601.2 1255 1891 2080

CENPI 89.15 340 98.2 249.5

CENPJ 1990 2450 2630 3070

CENPK 647 1290 1510 1050

CENPL 317 301 265 301

CENPM 6775 8390 14180 16130

CENPN 2759.9 10556.9333333333 4011 5683.93333333333

CENPO 895 1066 893 936

CENPP 420.2 576.666666666667 817.1 941.666666666667

CENPQ 854.5 894.2 1189 1113

CENPT 107 200 119 227

CENPV 1450 1840 5.42 1310

CEP110 121.466666666667 139.766666666667 199.666666666667 216.633333333333

CEP120 296 599 523 479

CEP135 1080 1060 510 2130

CEP152 402 263 673 415

CEP164 105.255 159.3 235.2 245.3

CEP170 872 689.25 661.3 1002

CEP192 1030 2490 1900 1110

CEP250 466.5 792 376.5 672

CEP290 258 624 570 1290

CEP350 827 787.5 479.5 742

CEP55 1454.27272727273 2274.45454545455 6303.63636363636 1719.81818181818

CEP57 791 796 1700 1460

CEP63 673 1080 1780 673

CEP68 96.55 154 129.5 222

CEP70 571 526 607 522

CEP72 1580 1260 3310 1730

CEP76 363.3 1088 608.2 356

CEP78 673 1430 1170 1920

CEP97 54.3 99.1 173 147

CEPT1 259 270 601 512

CER1 8.43 4.64 4.25 5.72

CERCAM 98.85 527 712 2078.5

CERK 1310 1400 558 1300

CERKL 3.22 5.175 4.815 3.95

CES1 56.55 644.6 51.0466666666667 56.2333333333333

CES2 194 238 767 426

CES3 23.4 19.5 44.2 69.3

CES4 6.97 57.2 4.23 4.89

CES7 3.395 5.321 5.199 4.115

CES8 3.34 5.22 4.98 3.94

CETN1 2.83 4.55 4.15 3.46

CETN2 2750 2420 1330 5720

CETN3 1190 3860 2860 3450

CETP 9.16 6.68 4.38 3.58

CFB 99 233 20 4.15

CFC1 8.82 13.1 15.1 12.2

CFD 2470 595 5230 2260

CFDP1 914 1620 2550 2600

CFH 54.0366666666667 4269 97.3666666666667 96.48

CFHR1 4.58 5.49 5.11 4.31

CFHR2 8.09 12.4 4.67 3.82

CFHR3 4.01 1312 39.71 4.639

CFHR4 3.27 14.1 4.87 93.6

CFHR5 2.74 55.2 8.04 3.35

CFI 1446 786 18.285 8.535

CFL1 13950 13800 12350 11100

CFL2 1800 2010 422 2180

CFLAR 502.5 620.55 771 182.3

CFLP1 8.37 13 12.7 15.5

CFP 69.8 49.4 103 131

CFTR 4.498 143.4 6.115 4.43

CG012 77.8 46.1 22.6 138

CG030 74 63.5 37.7 114

CGA 221 4.51 4.1 88.8

CGB 21.1 28.3 39 43.9

CGB2 30.6 43.9 37.9 33.7

CGGBP1 473 597 610 1180

CGN 4340 7640 218 3200

CGNL1 3210 1970 17.5 657

CGREF1 296 343 199 4.43

CGRRF1 138 249 283 336

CH25H 3.16 5.14 4.7 3.82

CHAC1 2873 281.65 143.55 351.35

CHAC2 884 846 1310 1850

CHAD 39.3 23 46 33.1

CHADL 240 46.1 31.8 32.3

CHAF1A 6088.5 3450.5 5480 7600.5

CHAF1B 3621 3078 4405 6426

CHAT 170 198 60.7 184

CHCHD1 10300 14400 12400 12100

CHCHD10 6080 12600 14400 14600

CHCHD2 22400 11200 12500 12800

CHCHD3 1830 2720 4800 3300

CHCHD4 6570 6090 10500 7560

CHCHD5 1330 1750 2000 1510

CHCHD6 368 298 988 867

CHCHD7 1431.5 1715 2100 5090

CHCHD8 9280 8850 11500 12100

CHD1 988 1540 741 1710

CHD1L 2319 2870 745 2916

CHD2 527.066666666667 340.133333333333 309.726666666667 302.91

CHD3 1150 2780 2890 2370

CHD4 1760 2010 2180 3440

CHD5 8.48 9.50333333333333 9.16 10.1933333333333

CHD6 510.85 699.3 452.55 678.7

CHD7 2030 1860 782 2160

CHD8 4480 5270 3370 5690

CHD9 336 599 1482.5 1107

CHDH 947.5 480 739 460.5

CHEK1 1280 1690 4620 1740

CHEK2 895.5 895 564.5 980.5

CHERP 716 503 522 908

CHFR 463 553 233 418

CHGA 3.27 5.33 33.7 218

CHGB 5.47 13 4.97 9.58

CHI3L1 3.82 11.8 4.69 5.81

CHI3L2 3.316 5.204 4.8 3.953

CHIA 14 27.3 24.1 23.1

CHIC1 30.5 11.335 55.8 80.6

CHIC2 448.8 504.9 126 355.5

CHID1 449 627 1390 1250

CHIT1 276.85 272.49 322.19 237.82

CHKA 17600 9360 4330 2720

CHKB 725 462 380 589

CHL1 3.16 5.11 4.67 3.85

CHM 301.05 5.255 189.35 516

CHML 1560 1590 598 2310

CHMP1A 1074 1504.5 1806.5 1605.5

CHMP1B 344 479 624 304

CHMP2A 15000 3810 14800 9590

CHMP2B 743 782 420 1080

CHMP4A 6550 5980 1870 4790

CHMP4B 935 2800 1660 2480

CHMP4C 1090 378 916 221

CHMP5 5335 4110 5620 7270

CHMP6 497 468 530 720

CHMP7 311 464 624 460

CHN1 41.5 428 160 1170

CHN2 3305 1461.5 79.4 206.5

CHODL 5300 36.1 76.2 43.8

CHORDC1 2999.5 2640 4660 5155

CHP 33540 27620 17170 10750

CHP2 11.3 5.29 10.3 76.9

CHPF 5200 4760 8470 7010

CHPF2 266 292 408 494

CHPT1 1292 1532 1274 2036

CHRAC1 824 484 789 660

CHRD 219.7 158.35 71.75 103.7

CHRDL1 6.195 10.72 7.635 232.15

CHRDL2 21.4 13.205 8.17 6.92

CHRFAM7A 53 48.6 56.4 160

CHRM1 4.34 5.2 4.93 3.91

CHRM2 7.745 5.19 5.22 9.855

CHRM3 19.2 7.08 6.79 29.3

CHRM4 37.3 14.3 28 19.1

CHRM5 8.827 9.876 6.715 6.4

CHRNA1 7.51 17.4 9.865 12.7

CHRNA10 120 86 248 194

CHRNA2 3.88 51.1 17.7 7.88

CHRNA3 3.77 5.79 5.68 82.1

CHRNA4 20.8 11.2 5.34 10.3

CHRNA5 444 858 1240 3090

CHRNA6 17.9 21.7 15.8 28

CHRNA7 7.14 5.81 17.1 645

CHRNA9 3.1 6.05 4.55 3.71

CHRNB1 140 159 51.4 237

CHRNB2 3.5 5.64 5.16 4.14

CHRNB3 65.92 63.18 56.67 65.26

CHRNB4 7.43 15.6 19.5 8.02

CHRND 25.6 27.7 20 20

CHRNE 12.7 23.7 22.7 19.2

CHRNG 3.2 5.2 4.76 3.89

CHST1 7.005 5.45 5.05 5.57

CHST10 60.2666666666667 69.4333333333333 58.9 184.333333333333

CHST11 3.22 64.8 48.5 279

CHST12 2370 2590 2000 1690

CHST13 2221 1948 730.6 591.75

CHST14 56.8 83.3 115 94.8

CHST15 9.92 120 1210 620

CHST2 4 6.27 2010 502

CHST3 149 229 347 93.8

CHST4 63.4 62.3 93.1 211

CHST5 43.9 55.4 53.9 44

CHST6 1335.7 1784 1383 1453

CHST7 122 451 409 659

CHST8 71.4 66.5 41.3 396

CHST9 582 423 67.6 60.8

CHSY1 1040 1190 2920 5490

CHSY3 57.7 49.9 656 89.2

CHTF18 3040 3000 2430 8340

CHTF8 1460 2020 2240 1900

CHUK 7170 9800 4530 7260

CHURC1 174 227 131 462

CIAO1 1490 2360 1100 1810

CIAPIN1 1750 2545 4280 4475

CIB1 3660 6270 14300 3630

CIB2 573 2350 398 3990

CIB3 42.5 38 33.6 47.6

CIB4 2.63 4.27 3.86 3.24

CIC 1900 1610 1540 1100

CIDEA 7.9 9.09 5.6 6.93

CIDEB 676 380 41.5 23.9

CIDEC 875 2712 857 434.5

CIDECP 482 882 514 304

CIITA 7.985 11.4 11.21 8.925

CILP 2.94 4.77 9.09 12.7

CILP2 4.05 42.1 23.4 285

CINP 698 870 1180 1310

CIR1 671 449 386 726

CIRBP 1657.5 916 1524.5 2045

CIRH1A 4120 4480 8660 6670

CISD1 3964.5 8007 4734 6864.5

CISD2 1780 2310 720 3310

CISD3 11800 15100 11800 14600

CISH 210 310 79.9 307

CIT 889.8 1279.4 2245.5 2398.7

CITED1 8.38 6.04 28.9 3320

CITED2 820.55 3736.5 1058.15 1456.75

CITED4 3830 2830 20900 1300

CIZ1 610.5 877 827 1121.5

CKAP2 569.573333333333 1134.41333333333 1518.81333333333 2397.63333333333

CKAP2L 965 872 1120 796

CKAP4 2240 11300 2330 5360

CKAP5 5740 4330 4620 6200

CKB 5270 5030 212 22700

CKLF 1215.58333333333 2346 5110.83333333333 1596.75

CKM 66.5 5.7 5.24 42.5

CKMT1A 7.555 35.98 217.5 6925

CKMT2 3.78 6.01 5.55 4.47

CKS1B 25950 45300 26495 20140

CKS2 24700 27400 30500 26200

CLASP1 650.5 510 556.5 490

CLASP2 1192.75 1558.65 1729.7 2146.6

CLC 11.304 7.214 5.33 12.452

CLCA1 3.72 6.01 5.5 4.36

CLCA2 3.39 5.54 12.8 4.09

CLCA3P 3.4 4.99 4.52 3.68

CLCA4 4.8 22.5 3.82 7.26

CLCC1 591 794.333333333333 624.533333333333 1059

CLCF1 171 326 650 18.7

CLCN1 69.3 62 80.3 73.2

CLCN2 200 248 577 133

CLCN3 424 753 279 935

CLCN4 226 156 416 4.075

CLCN5 803 1107 405 522

CLCN6 564.15 741.75 504.35 335.1

CLCN7 534 336 567 453

CLCNKA 16.5 25.7 23.6 21.9

CLCNKB 20.3 58.1 16.8 97.9

CLDN1 11900 13700 12700 9.6

CLDN10 3.63 18.6 5.37 224

CLDN11 2510 279 38.6 6.81

CLDN12 675.85 584 362.7 773.75

CLDN14 80.7 14 4.96 11.3

CLDN15 138 71.8 96.9 134

CLDN16 37.1 141 77.3 33.2

CLDN17 41.3 6.21 11.6 9

CLDN18 2.75 4.48 7.16 8.81

CLDN19 509.25 494.15 431.51 409.84

CLDN2 219 492 66.1 45.5

CLDN20 4.27 5.67 5.22 4.29

CLDN22 23.59 27.615 28.12 26.935

CLDN23 739 1140 423 77.3

CLDN25 12.1 10.5 19.3 12.1

CLDN3 91 151 7.8 44.8

CLDN4 149 1750 475 326

CLDN5 11 5.91 5.12 4.19

CLDN6 8.7 2100 13.5 14

CLDN7 63.8 130 218 91.8

CLDN8 3.21 5.22 4.74 3.85

CLDN9 49.4 41.25 44.95 51.85

CLDND1 2000 5400 5130 5360

CLDND2 16.2 9.94 49.3 17

CLEC10A 44.8 33 41.2 37.5

CLEC11A 5.23 4.71 8920 2140

CLEC12A 3.34 5.36 4.98 6.63

CLEC12B 63.33 55.8 52.7 55.93

CLEC14A 9.4 12.5 11.3 12.9

CLEC16A 1540 936 1910 1580

CLEC17A 8.53 17.9 12 7.62

CLEC18B 8.81 38.4 132 140

CLEC18C 5.51 4.29 20.3 19.3

CLEC1A 4.06 15.2 6.38 7.1

CLEC1B 7.74333333333333 8.10333333333333 32.1633333333333 6.4

CLEC2A 3.14 5.04 4.66 3.82

CLEC2B 15.9 24.2 19.7 15.5

CLEC2D 4072.16333333333 6775.16666666667 6238.20666666667 9115.16666666667

CLEC2L 3.11 5.08 4.62 3.75

CLEC3A 3.78 6.03 5.7 4.72

CLEC3B 13.7 11 21.5 146

CLEC4A 35.9 19.9 155 93.1

CLEC4C 3.74 5.91 5.48 6.39

CLEC4D 3.345 5.365 6.5 11.1

CLEC4E 2.71 4.39 3.98 3.32

CLEC4F 10.4 8.88 17 18.5

CLEC4G 21.75 25.3 46.55 21.9

CLEC4GP1 2.8 5.38 6.94 3.41

CLEC4M 74.135 97.355 83.75 75.85

CLEC5A 3.96 6.27 5.86 4.78

CLEC6A 10.1 5.26 5.42 4.3

CLEC7A 4.91 7.05 5.465 4.47

CLEC9A 19.9 5.54 15.1 27

CLECL1 2.8 4.52 4.11 3.42

CLGN 2100 2260 381 692

CLIC1 32100 25200 30300 9010

CLIC2 3.83 584 5.79 47.6

CLIC3 66 77.7 319 10.6

CLIC4 1224 1436.66666666667 1946.66666666667 717.666666666667

CLIC5 4002.27 3186.46 4214.78333333333 1040.53333333333

CLIC6 8.3 5.7 5.23 4.27

CLINT1 2880 3090 9090 5530

CLIP1 1550 2350 2080 1770

CLIP2 336.1 563.2 350.1 54.1

CLIP3 20.3 27.9 42.5 9.58

CLIP4 15.0666666666667 718 281.333333333333 304

CLK1 499.5 385.5 382 609

CLK2 354 580 330 921

CLK3 4070 5560 2960 4020

CLK4 217 190 200 471

CLLU1 3.1 5.04 4.59 3.78

CLLU1OS 17.1 5.29 11.9 10.3

CLMN 815 268.45 63.95 306.5

CLN3 1600 654 603 682

CLN5 497 788 311 1440

CLN6 1000 1540 816 1690

CLN8 210 49.4 92.4 126

CLNK 4.66 5.76 5.43 4.42

CLNS1A 11300 10000 13200 22800

CLOCK 408 255.5 306.5 257.5

CLP1 1030 2100 580 1130

CLPB 493 576 2400 2060

CLPP 29200 10100 32400 36300

CLPS 88.1 93.9 104 94.2

CLPTM1 869 1500 1590 1130

CLPTM1L 8307 7259 7964 8348

CLPX 3270 3210 1630 2070

CLRN1 4.53 11.21 8.31 9.69

CLRN1OS 222 232 208 221

CLRN2 3.82 6.13 9.59 8.85

CLRN3 5.45 2380 5.34 4.37

CLSPN 394 559 763 753

CLSTN1 835 3320 9720 1970

CLSTN2 3.9 6.17 5.67 4.57

CLSTN3 2204.5 1751.5 501.1 1075.35

CLTA 21900 23600 23300 21800

CLTB 362 577 892 695

CLTC 4.45 5.19 4.74 3.85

CLTCL1 455 309 228 395

CLU 19000 19800 7910 1090

CLUAP1 472.5 820.5 1925 1410

CLUL1 9.35 12.5 9.84 7.87

CLVS1 2.76 4.47 4.06 3.38

CLVS2 7.93 5.29 4.88 6.17

CLYBL 575 70 337 204

CMA1 19.63 30.5 21.745 28.4

CMAH 5.83333333333333 7.48333333333333 4.66333333333333 4.84666666666667

CMAS 675 1410 1260 1860

CMBL 4.01 6.26 5.89 15.2

CMC1 6150 8000 12700 5020

CMIP 830 945 286 380

CMKLR1 6.72363636363636 10.9463636363636 12.7136363636364 10.8381818181818

CMPK1 1427.27272727273 3409.09090909091 1244 1014.45454545455

CMPK2 28.2 22.6 16 25.2

CMTM1 22.79 58.76 201.8 95.33

CMTM2 3.15 5.12 6.61 3.79

CMTM3 8585 9350 44.55 6135

CMTM4 3220 2130 3550 4410

CMTM5 7.27 20.2 6.96 9.44

CMTM6 1810 4210 6130 1790

CMTM7 3679.5 5134 9965 5035.5

CMTM8 7930 4570 3020 2090

CMYA5 13.2 33 4.7 53.6

CN5H6.4 208 274 451 288

CNBD1 2.81 4.52 7.35 3.43

CNBP 10455 12315 14960 13310

CNDP1 9.21 11.2 20.3 13.3

CNDP2 1460 1640 3650 1400

CNFN 243 365 533 1040

CNGA1 23.1 5.33 6.47 8.35

CNGA2 2.97 4.84 4.39 3.63

CNGA3 6.225 8.49 7.915 6.195

CNGA4 2.85 4.59 4.19 3.47

CNGB1 160.2 150.3 160.2 190.75

CNGB3 3.59 10.6 5.41 4.4

CNIH 18800 17200 15200 17200

CNIH2 88.4 55.8 33.2 40.1

CNIH3 3.66 5.74 182 102

CNIH4 19000 8830 20700 32000

CNKSR1 1250 182 1230 66.4

CNKSR2 13.6 38.8 4.53 3.7

CNKSR3 2351 2523.5 1062.8 1194.4

CNN1 10.9 1360 4.29 56.6

CNN2 11000 9990 5490 1350

CNN3 3910 8460 3140 4820

CNNM1 386 360 182 60.5

CNNM2 56.1 106.1 81.95 117

CNNM3 1460 1500 1290 1660

CNNM4 2483 2836 5191 3032

CNO 1690 1610 619 1780

CNOT1 271 254 746 458

CNOT10 1849 2918 4635 4511

CNOT2 2593.305 3248.915 2895.1 3416.35

CNOT3 5240 8330 6930 8500

CNOT4 465.6 709.375 614.575 1079.325

CNOT6 392 1160 764 1620

CNOT6L 244 222.5 212.5 537

CNOT7 2710 4540 3950 2830

CNOT8 457.3 516.2 800 650.4

CNP 16891.3 15240 18489.5 16152

CNPY1 27.4 21.205 19.035 504.5

CNPY2 36500 56300 46700 56000

CNPY3 516 466 735 631.5

CNPY4 68.3 199 189 179

CNR1 2.68 13.1 11 43.5

CNR2 23.4 20.1 28.1 32.3

CNRIP1 3.72 5.915 5.6 398.65

CNST 381.666666666667 198.733333333333 197 144.1

CNTD1 203 230 206 226

CNTD2 65.1 74.7 73.1 70.2

CNTF 39.3 21.4 42.3 61

CNTFR 3.55 20.1 5.34 51.9

CNTLN 216.525 117.975 154.05 294

CNTN1 4.58 8.425 5.91 55.05

CNTN2 9.6 6.15 5.85 4.9

CNTN3 16.5 6.3 5.9 4.83

CNTN4 6.09 14.8 79 31.8

CNTN5 2.94 18.3 4.35 5.51

CNTN6 47.2 55.7 48.1 46.9

CNTNAP1 96.3 189 98 137

CNTNAP2 3.69 493 5.43 966

CNTNAP3 3.545 17.81 146.65 90.6

CNTNAP4 3.475 158.2 5.185 4.275

CNTNAP5 16.675 15.83 10.8 13.375

CNTROB 757 877 1170 1060

COASY 3990 3830 3620 4510

COBL 1633.5 254.75 35.45 252.9

COBLL1 7120 4840 9490 1350

COBRA1 2550 2830 3010 3300

COCH 3230 11400 5.37 1860

COG1 2780 3090 5070 4500

COG2 2750 1720 2590 4700

COG3 583 928 652 816

COG4 461.5 531.5 853.5 458.5

COG5 746 1910 1500 1680

COG6 616.39 617.26 752.055 1306.695

COG7 850 672 1270 1140

COG8 3544.5 2996.5 4720 6052.5

COIL 4060 2610 3100 8040

COL10A1 99.1 102 135 128

COL11A1 4.27 6.33 6.05 5.16

COL11A2 165.966666666667 178.066666666667 206.733333333333 178.866666666667

COL12A1 19.72 639.6 620.1 61.665

COL13A1 3.488 5.575 5.165 172.3

COL14A1 23.59 45.8 15.705 33

COL15A1 3.366 5.379 4.999 4.124

COL16A1 174.25 422 163.69 94.95

COL17A1 3.85 6.09 5.59 4.55

COL18A1 14200 9950 33900 4080

COL19A1 7.35666666666667 7.97333333333333 7.83666666666667 11.33

COL1A1 2.91 1080 75.7 597

COL1A2 7.932 372.6 5.874 5.737

COL20A1 18.5133333333333 21.02 17.49 19.9666666666667

COL21A1 25.5 194 4.46 62.4

COL22A1 3.81 6.03 5.63 4.6

COL23A1 24.6 17.6 15.6 49.8

COL24A1 22 111 218 50.5

COL25A1 10.7 8.95 9.18 120

COL27A1 4231 2815 676.5 880

COL28A1 3.61 18.1 5.4 4.36

COL29A1 3.26 6.7 4.84 3.93

COL2A1 38.7 23.5 4.25 474

COL3A1 2.87 4.59 5.25 8.85

COL4A1 61.5 42.8 2480 474

COL4A2 150 180 14700 6570

COL4A3 3.45 5.6 5.13 4.21

COL4A3BP 1085.5 1285 2385 1770

COL4A4 17.6 15.1 259 87

COL4A5 3.97 3540 211 1760

COL4A6 3.73 2140 5.62 1790

COL5A1 28.8633333333333 25.5033333333333 31.3866666666667 134.68

COL5A2 482 10600 51.9 736

COL5A3 3.77 6.09 5.56 4.42

COL6A1 68.7 1770 325 3960

COL6A2 7.385 14.125 12.205 50.65

COL6A3 3.085 4.985 4.56 3.755

COL6A4P2 4.215 5.41 5.11 4.32

COL6A6 3.79 6.06 5.58 4.47

COL7A1 15.6 151 46.2 111

COL8A1 3.55 5.78 5.27 11.2

COL8A2 83.95 22.885 19.005 25.15

COL9A1 33.6 42.4 45.9 39.9

COL9A2 67.79 105.595 26.695 27.59

COL9A3 1570 1130 160 1420

COLEC10 2.55 4.13 4.38 3.15

COLEC11 169.7 11.128 257.9 325.5

COLEC12 16.4 56.4 4.22 336

COLQ 2.67 18.7 30 43

COMMD1 4480 4940 3980 4040

COMMD10 689 1480 2790 1070

COMMD2 945 675 1160 791

COMMD3 10700 8860 3650 6910

COMMD4 14285 13935 14805 22190

COMMD5 2750 1350 2290 2530

COMMD6 2165.7 6713.35 7271.95 11575.1

COMMD7 637 654 701 669

COMMD8 3990 5375 4360 3350

COMMD9 2140 1530 1390 2260

COMP 1640 231 40.7 249

COMT 3590 2920 2190 3540

COMTD1 2560 1460 4090 6730

COPA 1120 642 781 886

COPB1 9680 10300 10700 9060

COPB2 14400 15200 18200 17100

COPE 1150 1530 1060 1220

COPG 1290 1530 1310 775

COPG2 249 243 372 822

COPG2IT1 107 31.5 79.9 4.27

COPS2 4750 4450 2790 4370

COPS3 10100 16300 9150 15900

COPS4 3140 5350 3820 9020

COPS5 10100 15200 16900 17700

COPS6 7190 18300 20700 20900

COPS7A 572 767 1600 1390

COPS7B 148 158.5 119.5 234

COPS8 1164 1429.5 1430 2680

COPZ1 6520 12100 15800 9190

COPZ2 3.399 35.04 5.611 4.415

COQ10A 281 728 911 1200

COQ10B 216.636363636364 279.090909090909 298.181818181818 173.363636363636

COQ2 265 323 404 734

COQ3 512.3 425.1 435.9 699.1

COQ4 300 494 500 746

COQ5 1240 2374.54545454545 3660.90909090909 2725.45454545455

COQ6 341 397 985 781

COQ7 1190 438 627 1910

COQ9 1190 866 2700 2780

CORIN 5.53 8.28 10.8 10.8

CORO1A 143 88.8 49.7 117

CORO1B 1005 630.5 513 317

CORO1C 2920 4380 4340 3420

CORO2A 1099.5 2100 1125 585.5

CORO2B 19.34 18.94 27.47 30.08

CORO6 165 306 141 362

CORO7 788 404 1080 1820

CORT 149 179 318 253

COTL1 279 2700 18700 4560

COX10 394 443 395 314

COX11 3336.33333333333 2958.33333333333 7439 4608

COX15 212.5 259.5 302 259.5

COX16 8360 11900 11300 16100

COX17 26500 22600 4850 28100

COX18 16.1 25.1 13.3 37.9

COX19 423 525 331 384

COX4I1 26200 24700 25900 32900

COX4I2 22.7 22.6 27.4 18.3

COX4NB 7016 9151 13070 12680

COX5A 27100 25300 16200 29300

COX5B 16000 17800 25600 20000

COX6A1 104000 101000 113000 164000

COX6A2 74.2 47.6 73.9 83.9

COX6B1 31200 33100 33100 29000

COX6B2 5146.6 5351 4925.7 4575.5

COX6C 34400 23900 31800 29800

COX7A1 2.72 4.42 13 6.85

COX7A2 83200 81000 60400 64800

COX7A2L 5815 5670 5185 5755

COX7B 14900 16900 13400 34700

COX7B2 2.94 172 4.29 6.04

COX7C 71120 63940 95590 103720

COX8A 58800 43300 68800 58800

COX8C 2.88 6.71 23.7 5.57

CP 240.5 246 36.95 14.375

CP110 273 585 1280 1600

CPA1 2.92 4.52 4.1 3.42

CPA2 5.05 5.59 27.6 12.5

CPA3 22.8 26 30.4 32.6

CPA4 3.38 5.49 5.02 4.12

CPA5 20.3 20.1 24 29.4

CPA6 3.18 242 4.73 3.79

CPAMD8 22.05 16.495 23.85 31.95

CPB1 9.9 7.45 10.4 11.7

CPB2 302 418 5.45 4.49

CPD 1810 967 1070 622

CPE 2420 3060 555 5010

CPEB1 172 4.16 357 287

CPEB2 40.6 76.2 71.5 69.5

CPEB3 35.1 11 47.1 60.6

CPEB4 1350 1750 707 977

CPHL1 3.44 27.6 5.27 4.63

CPLX1 987 164 10.7 189

CPLX2 24.7 20.735 20.945 15.575

CPLX3 12.395 13.755 21.02 13.743

CPLX4 3.6 5.79 5.31 4.25

CPN1 539 616 31.5 16.8

CPN2 183 20.5 5.12 4.18

CPNE1 5210 12650 5980 4940

CPNE2 129.93 360.8 27 271.955

CPNE3 414 471 681 1110

CPNE4 4.27 4.28 3.9 7.69

CPNE5 25.8 32.8 35.5 48.8

CPNE6 11.7 9 10 4.89

CPNE7 4540 908 1450 1100

CPNE8 658 517 1160 397

CPNE9 3.47 5.52 9.48 6.57

CPO 3.008 4.943 5.802 4.131

CPOX 2100 4580 4680 6560

CPPED1 3380 927 1260 382

CPS1 46.3 3040 676 1230

CPSF1 470 341 332 505

CPSF2 66.9 51.2 17.9 102

CPSF3 2220 3080 3130 3790

CPSF3L 6484.5 4600.5 7420 3167.5

CPSF4 3200 5340 3330 4280

CPSF4L 31.1 22.7 26.6 22.5

CPSF6 3510 3790 3580 5630

CPSF7 8370 11300 10900 10700

CPT1A 617 103.63 1061 246.7

CPT1B 152 345 196 409

CPT1C 8.3 104.7 113.75 1418.5

CPT2 1020 1770 2180 1580

CPVL 638 1570 40.3 1250

CPXCR1 2.98 4.78 32.3 4.19

CPXM1 14.7 18.2 17.3 17.3

CPXM2 3.54 5.815 6.08 4.07

CPZ 3.32 5.39 4.92 4.92

CR1 3.25 5.3 4.82 3.91

CR1L 5.36 4.59 5.41 5.11

CR2 2.925 4.635 4.285 187.35

CR848007.8 3.24 5.21 4.82 8.35

CRABP1 55.85 192.5 42.645 54.965

CRABP2 31.1 130 57 404

CRADD 1580 1630 1140 1310

CRAMP1L 63.8 59.3 61.1 126

CRAT 3770 6240 6090 1970

CRB1 5.08909090909091 6.37 5.66909090909091 11.3009090909091

CRB2 3.54 5.66 8.51 4.23

CRB3 5430 935 2150 66.5

CRBN 1600 1130 1220 1380

CRCP 924 1620 447 756

CRCT1 3.84 5.59 19.6 23

CREB1 719 963 471 1070

CREB3 630 1120 1050 817

CREB3L1 1650 1740 1340 1780

CREB3L2 1673.5 5920.5 1219.5 2324

CREB3L3 1840 1630 28.7 17.9

CREB3L4 581.5 798 763 795.5

CREB5 1630 233 30 543

CREBBP 1360 1380 1630 2270

CREBL2 110 331 188 380

CREBZF 366 270 503 657

CREG1 3550 4220 8550 9890

CREG2 5.2 11.4 7.95 8.12

CRELD1 503.3 342.25 771.5 510.6

CRELD2 6450 10700 9080 11100

CREM 238.5 2290 483 527.5

CRH 3.31 5.31 14.6 3.97

CRHBP 3.56 5.67 5.31 4.29

CRHR1 77.85 65.45 78.45 106.7

CRHR2 56.9 51.3 55.4 67.5

CRIM1 32.6 97 33.1 9.39

CRIP1 834.5 1106.5 330.85 99.085

CRIP2 805 49.8 2340 32

CRIP3 3.505 22.85 6.34 126.3

CRIPAK 1350 1130 269 950

CRIPT 1500 652 1240 1660

CRISP1 3.84 6.07 5.61 4.52

CRISP2 3.15 235 4.69 3.86

CRISP3 3.02 41.4 4.47 3.63

CRISPLD1 2.84 11.3 79.6 130

CRISPLD2 6.401 20.063 7.019 7.974

CRK 744 581 819 538

CRKL 1290 1910 740 1500

CRKRS 600 438 549 594

CRLF1 6380 4130 182 471

CRLF2 38.95 38.9 31.9 49.65

CRLF3 123 294 319 267

CRLS1 11900 9000 8900 8760

CRMP1 17.4 6.32 14.4 26.9

CRNDE 1240 1710 4710 6840

CRNKL1 398 661 317 545

CRNN 23.57 30.02 36.35 24.3

CROCC 153.35 94.95 484.15 294.45

CROCCL1 1425.5 1779 2110.5 1852

CROCCL2 5.9 10.6 33.4 30.2

CROT 78.55 23.85 11.025 55.7

CRP 9.325 10.16 10.45 50.185

CRTAC1 9.4 6.14 8.22 15.8

CRTAM 3.35 5.37 4.96 4.05

CRTAP 13.7 12.9 67.9 47.2

CRTC1 508.5 532.1 603.5 724.5

CRTC2 3169 5043 3822 5337

CRTC3 290 512 198 548

CRX 7.44 6.32 9.8 12.2

CRY1 262 684 820 743

CRY2 2280 3140 832 1790

CRYAA 107.6 110.825 117.64 117.58

CRYAB 120 151 252 35.7

CRYBA1 9.82 15 22.3 12.8

CRYBA2 141 99.1 171 144

CRYBA4 6.61 5.63 10.6 4.92

CRYBB1 4.01 6.2 5.81 4.76

CRYBB2 1380 859 312 983

CRYBB2P1 6810 10700 7230 10100

CRYBB3 5.35 4.3 3.9 3.26

CRYBG3 284 617 705 559

CRYGA 34.3 38.6 35.2 50.2

CRYGB 72.8 6.07 16.1 4.17

CRYGC 7.62 52.6 4.44 6.72

CRYGD 30.3 18.2 23.2 2150

CRYGN 26.8 18.8 29.4 19.5

CRYGS 171 102 75.5 64.3

CRYL1 1220 744 1640 488

CRYM 1650 515 617 559

CRYZ 1192 3700 4085 854

CRYZL1 80.1 174 206 415.5

CS 22900 23800 33500 34300

CSAD 40 43 51.6 70.6

CSAG1 1540 1110 30.1 8400

CSAG2 604 502 10.7 3550

CSAG3 67.6 74.3 48.6 350

CSDA 2385 2960 8740 10775

CSDC2 7.21 11.2 5.28 29.7

CSDE1 11600 12200 11700 15000

CSE1L 15300 22350 20050 22500

CSF1 142.566666666667 396 118.886666666667 96.6366666666667

CSF1R 12.405 13.3 15.196 23.98

CSF2 3.27 5.32 4.86 3.99

CSF2RA 15.94 18.695 39.8 28.45

CSF2RB 34.35 38.9 35 32.74

CSF3 3.25 5.255 4.815 3.94

CSF3R 124 536 126 113

CSGALNACT1 43.5 18.2 20.45 14.2

CSGALNACT2 83.6266666666667 149.966666666667 85.2 196.3

CSH1 62.5666666666667 67.4666666666667 75.5333333333333 78.6333333333333

CSH2 26.8 29.4 24.6 37.4

CSHL1 24.9 22.5 32 38.4

CSK 1340 1260 1120 1390

CSMD1 4.36333333333333 7.1 4.83333333333333 11.04

CSMD2 10.115 13.485 10.645 14.155

CSMD3 2.56 7.66 3.75 3.15

CSN1S1 25.92 24.364 25.34 30.662

CSN1S2A 4.13 6.36 6 4.97

CSN1S2B 2.87 4.65 4.22 3.48

CSN2 9.89 9.29 8.24 3.49

CSN3 4.63 6.69 6.95 8.71

CSNK1A1 344 384 642 527

CSNK1A1L 616 563 1280 699

CSNK1A1P 11.5 6.91 6.47 4.8

CSNK1D 3000 2320 2290 1740

CSNK1E 15766.5 15775.5 8869.5 63040

CSNK1G1 246 206 298 202

CSNK1G2 4850 2635 2640 4400

CSNK1G3 426 748 955.5 1085.5

CSNK2A1 528 780.272727272727 456.363636363636 640.090909090909

CSNK2A2 288 390 705 1030

CSNK2B 23100 12850 14850 13050

CSPG4 2.74 4.46 201 64.7

CSPG4LYP1 3.08 7.07 4.54 5.99

CSPG5 15.4 18.7 85.9 114

CSPP1 518 570 960 714

CSRNP1 168.5 492 130.65 134

CSRNP2 402 1020 891 766

CSRNP3 45.6333333333333 93.4666666666667 6.48333333333333 78.9666666666667

CSRP1 409.98 917.36 1362.175 10.005

CSRP2 13700 18100 10200 54400

CSRP2BP 4000 5230 2250 3330

CSRP3 3.12 5.06 4.63 3.75

CST1 13.8 14.8 9.63 8.98

CST11 3.784 5.944 4.817 4.08

CST2 10.6 8.3 10.5 9.54

CST3 1925 5234.5 2675.5 2296

CST5 957 2380 1300 1050

CST6 3.38 5.47 22 4.11

CST7 2.56 4.14 11 3.16

CST8 3.47 5.6 5.18 4.31

CST9 9.39 4.72 5.4 3.6

CST9L 3.29 5.36 4.91 13.7

CSTA 3.8 322 5.74 5.47

CSTB 11500 10100 39900 17400

CSTF1 3950 6290 5930 5050

CSTF2 2160 7030 2200 7330

CSTF2T 772 794 932 1170

CSTF3 2186 2302 1985 3420

CSTL1 13.2 11.655 7.975 11.685

CT45A1 4.34 5.4 4.96 15.1

CT45A5 3.72 21.4 13.9 147

CT47A11 886.935 1108.065 822.86 955.8

CT62 3.04 4.94 4.5 3.69

CTAG1A 3.74 123 5.5 99.9

CTAG2 82 130 153 152

CTAGE1 85.01 36.2966666666667 44.1333333333333 25.46

CTAGE4 1734 778 1818.5 460.5

CTAGE5 575.676666666667 243.046666666667 512.053333333333 128.546666666667

CTBP1 1603.5 1560.5 650.5 1207.5

CTBP2 296.333333333333 3039.56666666667 7789.7 7671

CTBS 222 610 567 334

CTCF 887 1240 2110 1520

CTCFL 4.2 6.43 6.09 5.08

CTD-2514C3.1 1570 1680 1660 1610

CTDP1 299 218.5 396 325.5

CTDSP1 9751 7790 8325 5152.5

CTDSP2 4543.5 6585.5 7091 4328.5

CTDSPL 97.5 765 2630 546

CTDSPL2 836 931 1060 636

CTF1 14 109 718 112

CTGF 1120 12200 274 318

CTH 1100 1510 1340 3540

CTHRC1 772.3 39.64 6.067 4.594

CTLA4 3.685 5.865 5.445 4.465

CTNNA1 4607 3858.5 5065 2961

CTNNA2 13.4 4.5 4.08 3.38

CTNNA3 10.89 5.46 10.995 5.755

CTNNAL1 15350 12705 10620 9940

CTNNB1 6372.72727272727 13155.4545454545 6628.18181818182 7474.54545454545

CTNNBIP1 1920 1717.5 927.5 912

CTNNBL1 5610 4958.5 7715 6650

CTNND1 1005 771 507.25 399.45

CTNND2 2924 675.2 54.69 11.687

CTNS 67.4 45.2 70.8 66.9

CTPS 3870 13200 11305 18750

CTPS2 678.181818181818 587.363636363636 505.090909090909 454.181818181818

CTR9 2540 3630 2440 5550

CTRB1 2.71 4.38 5.65 6.11

CTRB2 2730 2590 2640 2340

CTRC 32.5 29.595 25.3 20.15

CTRL 32.1 90 49.9 86.6

CTSA 13425 6575 8545 4345

CTSB 1080 1420 1260 631

CTSC 1610 1201.33333333333 1474.66666666667 732

CTSD 3200 2480 1730 1440

CTSE 93.73 109.67 86.8 72.34

CTSF 765 4820 20.3 2840

CTSG 24.1 26.1 15.6 19.3

CTSH 1050 6160 9720 4340

CTSK 341 331 590 249

CTSL1 1600 14800 7600 8830

CTSL2 1520 4620 3980 4180

CTSL3 4.64 15.9 6.29 6.94

CTSO 499 376 307 126

CTSS 3.36 5.5 5.02 4.05

CTSW 8.32 6.1 8.96 7.3

CTSZ 1483 1395.5 957 35.75

CTTN 24502 11131 8440.5 7696

CTTNBP2 74.8 307 3110 308

CTTNBP2NL 135 344 273 471

CTU1 2478.5 1529 2882 3085

CTU2 350 561 1020 1000

CTXN1 2830 1660 6300 2470

CTXN3 2.96 4.82 4.37 3.58

CUBN 35.2 5.7 5.28 10.1

CUEDC1 155 417 845 340

CUEDC2 1180 2030 4190 1850

CUGBP1 1940 2190 1550 2090

CUGBP2 4.16909090909091 6.14818181818182 5.55090909090909 88.9272727272727

CUL1 1030 1890 1590 4220

CUL2 425 2380 841 568

CUL3 7015.5 7218.15 7255.2 7739

CUL4A 6115 2401.5 4255.5 1804.5

CUL4B 747.5 938.5 428.5 2255

CUL5 1170 1650 1790 2690

CUL7 413 101 244 152

CUL9 1260 1210 2030 1350

CUTA 45500 19900 30200 40400

CUTC 609 1290 820 1550

CUX1 385.8 937.666666666667 793.5 451.8

CUX2 231.15 5.69 11.51 305.5

CUZD1 76.9 237 262 65.5

CWC15 4430 3650 6420 7510

CWC22 743 800 578 884

CWF19L1 750 1400 924 1700

CWF19L2 621 723 480 1040

CWH43 3.2 6.145 5.429 4.499

CX3CL1 87.5 43.8 40.7 13.9

CX3CR1 4.25 6.41 6.1 18.1

CXADR 1735 789 999 609.5

CXCL1 1454 316 43.185 39

CXCL10 90.45 8.85 4.445 3.64

CXCL11 3.12 4.85 4.45 3.69

CXCL12 1820.99230769231 30.8530769230769 17.4569230769231 18.9469230769231

CXCL13 3.362 5.488 6.888 4.742

CXCL14 9.04 18.6 40.7 23

CXCL16 2210 655.5 78.55 260

CXCL17 8.78 22 7.26 16

CXCL2 329 260.5 92.595 73.35

CXCL3 140 108 298 22.1

CXCL5 8720 5350 1210 1370

CXCL6 235 35.3 5.12 14.7

CXCL9 26.7 10.5 7.77 5.56

CXCR3 14.6981818181818 16.5581818181818 18.5245454545455 20.3718181818182

CXCR4 207.6 6.495 10590 752.7

CXCR5 11.1 6.95 9.49 3.81

CXCR6 3.648 9.916 4.967 28.156

CXCR7 29.3 31.1 4.06 11

CXXC1 615 700 1090 862

CXXC4 16.89 68.26 6.203 125.1

CXXC5 10200 6440 4220 1480

CXorf1 3.77 6.025 7.12 4.575

CXorf15 1530 3680 2560 5610

CXorf18 4.08 6.29 5.92 4.89

CXorf21 4.93 5.41 4.98 4.08

CXorf22 3.48 5.585 6.215 4.28

CXorf23 63.2 161 136.3 110.5

CXorf25 13.9 15.5 15.2 8

CXorf26 288 5.5 412 812

CXorf27 4.07 137 11.8 3.46

CXorf30 4.69 11.635 16.06 22.95

CXorf36 10.7775 8.1175 7.9425 8.31

CXorf38 425 698 1140 1260

CXorf39 4490 12000 3090 8570

CXorf40B 2660 3470 2465 10125

CXorf41 3.76 6.05 5.65 7.76

CXorf42 123 105 85.2 531

CXorf46 6.49 4.16 8.56 7.18

CXorf48 58.9 337 56.5 74.5

CXorf49B 80.5 102 58.7 149

CXorf50 11.35 8.79 7.63 190.1

CXorf51 2.68 4.35 3.95 3.28

CXorf56 177.5 287.5 222.5 916.5

CXorf57 606 4920 5.18 11600

CXorf58 3.09 5.03 4.58 3.69

CXorf59 3.29 5.28 4.89 5.34

CXorf61 2.92 125 4.33 3.55

CXorf62 13.3 40.4 17.5 21.9

CXorf64 30.2 30.9 31.9 25.3

CXorf65 3.04 33.7 7.05 11.6

CXorf66 3.16 5.14 4.7 3.81

CXorf67 2.95 8.94 4.34 69.2

CYB561 445 284 1650 912

CYB561D1 528 932 1080 1480

CYB561D2 2120 1350 2960 3160

CYB5A 13055.4545454545 7637.27272727273 9276.36363636364 6246.36363636364

CYB5B 35.1 508 223 901

CYB5D1 1342.1 697.6 240.01 2154.5

CYB5D2 315 220 686 499

CYB5R1 3350 4690 2970 3550

CYB5R2 151.3 1858.5 501.5 11.05

CYB5R3 57828 46567.5 35981 50775

CYB5R4 450 427 835.5 714.5

CYB5RL 84.75 173.65 196.75 681

CYBA 4420 6400 14800 859

CYBASC3 561 597 843 284

CYBB 3.8 6.04 5.63 13

CYBRD1 33.92 43.425 36.705 169

CYC1 8930 8180 12400 11600

CYCS 14950 18695.6666666667 8261 12161

CYCSP52 30.6 55.1 21.2 22.7

CYFIP1 8250 4170 6220 3540

CYFIP2 37.3 9.25 51.15 259

CYGB 17300 17800 16000 19200

CYHR1 874.666666666667 464.333333333333 589.333333333333 441

CYLC1 6.7 4.95 4.55 3.75

CYLC2 2.89 4.64 4.25 3.5

CYLD 102 77.7 108 243

CYMP 10.4 7.4 10.3 8.32

CYP11A1 66.2 40.1 35.4 34.7

CYP11B1 10.6 17.4 7.74 3.48

CYP11B2 2.68 4.35 3.94 3.28

CYP17A1 266 232 248 208

CYP19A1 9.04 12.8233333333333 5.86 9.44333333333333

CYP1A1 8910 9040 1920 127

CYP1A2 246 224 226 177

CYP1B1 325 465 1610 834.5

CYP20A1 795.5 1357 341 1087

CYP21A2 28.2 26.8 31.35 28.7

CYP24A1 8.1 13.1 10.54 4.155

CYP26A1 21.4 67.2 12.7 2100

CYP26B1 17.6 36.7 66.5 7.86

CYP26C1 15.1 20.5 13.9 16.8

CYP27A1 8350 1320 135 317

CYP27B1 97.4 110 260 212

CYP27C1 3.655 5.69 5.515 216

CYP2A13 54.3 51.1 66.1 65.6

CYP2A7 8.7 5.57 5.14 9.29

CYP2B6 185 1875 3343.5 147.5

CYP2C18 26.9 121 310 29

CYP2C19 6.26 22.1 7.36 11.2

CYP2C8 4.2 10.8 4.73 6.94

CYP2C9 43.4 107 126 19.6

CYP2D6 52.3 36.7 54.7 56.2

CYP2E1 3.4 5.49 5.03 8.53

CYP2F1 8.36 12.5 8.89 57.3

CYP2J2 2883 1973 265 1303

CYP2R1 2460 3920 2920 2390

CYP2S1 8.46727272727273 71.5818181818182 266.090909090909 134.654545454545

CYP2U1 74.5 15.5 113 140

CYP2W1 196000 208000 241000 237000

CYP39A1 91 221 15.1 102

CYP3A4 32.8 23.4 27.6 16.1

CYP3A43 11.2 9.96 10.8 5.21

CYP3A5 20.85 496.65 12.17 15.14

CYP3A7 106.4 478.3 12.53 12.48

CYP46A1 3.32 6.77 4.01 6.21

CYP4A11 40.3 42.15 39.35 44.45

CYP4A22 17.6 5.32 4.89 6.93

CYP4B1 6.484 13.787 8.492 7.418

CYP4F11 10.66 5.58 149.5 18.085

CYP4F12 2.96 31.9 58.9 3.62

CYP4F2 3.405 12.895 14.535 4.07

CYP4F22 3.51 5.67 5.25 10.3

CYP4F3 18.6 173 3.89 3.25

CYP4F8 123 226 223 134

CYP4V2 71.1 22.2 81.6 191

CYP4X1 2.72 4.41 3.99 14.9

CYP4Z1 9.37 9.18272727272727 6.27636363636364 7.37818181818182

CYP4Z2P 9.5 4.75 6.51 14.2

CYP51A1 8430 13100 6350 6340

CYP7A1 38.6 6.2 44.4 10.5

CYP7B1 111 35.2 9.64 16.4

CYP8B1 3.43 5.56 5.07 4.05

CYR61 7870 53500 16100 6990

CYS1 3.17 5.11 919 18.8

CYSLTR1 30 33.5 32.3 31.5

CYSLTR2 3.72 6.02 11.8 4.57

CYTH1 1627 1328.93333333333 1366.33333333333 1612

CYTH2 596 412 813 348

CYTH3 3220 1930 1770 3960

CYTH4 12.28 5.24 4.94 4.11

CYTIP 3.15 48.1 38.4 5.61

CYTL1 5.87818181818182 7.62090909090909 5.62727272727273 5.31545454545455

CYTSA 1734 5033.5 1332.05 3623

CYTSB 1165 1410 516.5 982.5

CYYR1 2.92 4.75 4.32 3.53

CYorf15A 3.43 5.6 5.13 4.13

CYorf15B 15.72 15.055 21.18 17.52

D21S2088E 210 201 364 189

D21S2089E 2.59 155 3.82 3.19

D21S2090E 3.79 5.84 5.72 4.97

D21S2091E 13.3 5.37 4.73 6.75

D2HGDH 1650 455 1860 2200

D4S234E 3.22 5.22 4.78 296

DAAM1 607 434 183 381

DAAM2 24.3 141 36.8 591

DAB1 124 323 119 111

DAB2 4680 4610 8.43 664

DAB2IP 456.472727272727 416.4 779.6 508.327272727273

DACH1 71.75 413 104.75 5435

DACH2 155 4.87 565 148

DACT1 9.11 13.4 6.53 716

DACT2 159 984 87.7 3.94

DACT3 27 58.4 66.5 510

DAD1 19690 29850 20980 26400

DAD1L 123 151 50 151

DAG1 1440 2810 3060 1310

DAGLA 891 415 73.6 254

DAGLB 529 245 276 293

DAK 3017 2836.5 5608 4045.5

DALRD3 441 1160 1400 737

DAND5 39.2 6.04 37.6 42.5

DAO 10.7 56.9 4.99 4.07

DAOA 3.47 5.565 5.135 4.165

DAP 3580 3690 4440 1590

DAP3 48400 65700 50100 39000

DAPK1 99.1 68.4 244 92.7

DAPK2 383 161 7.04 82.6

DAPK3 18000 8620 5320 11300

DAPL1 3.67 5.865 14.77 4.475

DAPP1 19.3 4.81 18.1 16.5

DARC 10.658 19.572 13.937 10.734

DARS 13200 11000 16600 16900

DARS2 2000 1640 1400 3060

DAXX 2140 1610 1960 1970

DAZ2 13.84 7.33333333333333 5.40666666666667 4.06333333333333

DAZAP1 3056.35 2527.75 2353.4 3881.3

DAZAP2 3918.05 7620.7 9389.5 8598.45

DAZL 3.32 5.38 4.92 4.04

DBC1 3.88 6.11 5.71 4.67

DBF4 868.65 800.2 947.75 1084.5

DBF4B 116.55 213.5 242.85 351

DBH 9.179 7.353 11.628 9.083

DBI 70600 87100 190000 34000

DBN1 14800 20800 15200 8020

DBNDD1 311 332 642 356

DBNDD2 3520 597 5370 2440

DBNL 493.55 229.5 540.5 437.55

DBP 42.3 85.4 293 333

DBR1 981 766 1180 897

DBT 299 864 371 1660

DBX1 16 6.87 14.9 13

DBX2 3.61 5.79 5.37 9.94

DCAF10 419.6 889 421.405 386.765

DCAF11 1106 914.7 939.8 1014.1

DCAF12 554 390 544 821

DCAF12L1 3.37 5.45 30.4 4.1

DCAF12L2 236 172 167 248

DCAF13 4670 4960 6440 5055

DCAF15 307 152 271 315

DCAF16 4960 5010 1140 5580

DCAF17 1310 1340 692 1640

DCAF4 169.3 593 632 1224

DCAF4L1 4.93 5.64 4.85 7.16

DCAF4L2 7.105 545.9 13.305 6.49

DCAF5 212 564 158 466

DCAF6 964 797.5 662 696.5

DCAF7 2000 3010 3250 3670

DCAF8 298.5 172 158.5 385.5

DCAF8L1 6.5 5.42 6.985 19.1

DCAF8L2 3.69 26.9 5.52 43.5

DCAKD 95.4 104.7 353 349.5

DCBLD1 225 427.5 153.5 245

DCBLD2 995.666666666667 2943.66666666667 2701 965

DCC 3.29 5.29 4.9 3.99

DCD 6.51 5.22 11.7 8.35

DCDC1 4.19 6.24 17.4 12.8

DCDC2 11000 2490 1940 200

DCDC2B 8.53 13.1 8.33 11.5

DCHS1 25.5 67.4 9.53 68.2

DCHS2 13.4166666666667 13.4933333333333 11.32 13.3733333333333

DCI 36000 18800 51200 34200

DCK 824 314 317 341

DCLK1 4.12666666666667 5.27333333333333 4.96333333333333 67.9333333333333

DCLK2 11.075 22.55 16.75 37

DCLK3 3.09 4.87 4.54 4.31

DCLRE1A 2110 2710 2790 3500

DCLRE1B 394 498 420 950

DCLRE1C 324.3 312.6 575.6 393.9

DCN 3.99 8.205 5.87 10.305

DCP1A 2850 2285 2175 5435

DCP1B 665 1170 890 2670

DCP2 1090 1480 2780 3010

DCPS 3020 3790 8850 4820

DCST1 15.5 15.7 15.7 22

DCST2 83.2 87.2 50.5 88.9

DCT 82.4 87.1 63.3 90.2

DCTD 1350 842 641 1070

DCTN1 2260 2140 2060 3290

DCTN2 5640 7560 7390 8990

DCTN3 2320 1660 3120 5200

DCTN4 2620 2470 3120 6050

DCTN5 696 397 586 574

DCTN6 1850 2840 3390 1830

DCTPP1 315 11200 24000 28300

DCUN1D1 852 694 612 370

DCUN1D2 341.15 128.5 104.2 143.85

DCUN1D3 2490 3440 1400 1530

DCUN1D4 2440 2910 662 1210

DCUN1D5 10800 9810 17000 21200

DCX 8.925 7.4 7.335 7.775

DCXR 4730 5580 7670 7270

DDA1 1210 939 1100 1190

DDAH1 5280 7110 8060 4200

DDAH2 9210 8350 4010 919

DDB1 14000 17500 15800 18300

DDB2 954 663 2120.5 1096.5

DDC 7010 7090 4840 95.2

DDEF1IT1 217 152 120 158

DDHD1 207 118 170 318

DDHD2 307 459 649 594

DDI1 2.78 4.51 4.1 3.38

DDI2 96.8 99 77.8 118

DDIT3 352 336 278 135

DDIT4 3119 882.9 823.9 2779

DDIT4L 295.5 35.05 191 388

DDN 50.9 42.5 64.7 37.7

DDO 3.44 5.53 5.09 4.2

DDOST 7120 6830 12900 7160

DDR1 95.3 113.25 408.95 149.6

DDR2 8.76 7.11 4.83 205

DDRGK1 1960 3440 1950 2100

DDT 39200 15300 11100 32100

DDTL 413 4.14 33.1 633

DDX1 9948 23550 24930 30790

DDX10 2765.5 3366.5 4646.5 6514

DDX11 526.566666666667 839.933333333333 1277.33333333333 1296.66666666667

DDX17 1642.5 1157.5 768.5 3800.5

DDX18 4620 4600 5460 6310

DDX19A 3450 5280 4290 6570

DDX19B 541 663 1290 1100

DDX20 2040 2490 2140 5620

DDX21 11800 15500 12800 21400

DDX23 7000 8960 11500 11500

DDX24 573 483 785 849

DDX25 2.69 4.37 3.96 7.56

DDX26B 3.3 5.48 133.2 475

DDX27 11800 12600 13000 13900

DDX28 2566 3303 5214 7003

DDX31 208.15 503.5 210 301.5

DDX39 31200 12600 14200 23400

DDX3X 2660 4330 2550 4170

DDX3Y 52.595 97.17 55.005 95.01

DDX4 5.39 50.6 20.4 11.2

DDX41 1820 1670 2740 2830

DDX42 4680 4510 4920 6010

DDX43 327 1710 4.96 4.02

DDX46 408 689 464 955

DDX47 9380 14800 27100 20600

DDX49 2040 2540 2400 2810

DDX5 32100 25900 28600 42500

DDX50 36.1 65.4 52.6 92.5

DDX51 896.5 1062.5 1011 1421.5

DDX52 165 149 421 463

DDX53 15.2 5.95 5.52 4.53

DDX54 6216 10290 8892.5 13655

DDX55 1219.5 1885 2740 3670

DDX56 35500 18700 35800 31500

DDX58 206.5 110.4 399.5 149.05

DDX59 377 336 280 456

DDX6 587 1360 1385 1640

DDX60 19.2 21.2 27.8 31.1

DDX60L 72.2 214 12.2 3.97

DEAF1 1074.72727272727 857 1994.45454545455 4295.45454545455

DEC1 3.2 5.2 4.75 3.89

DECR1 5410 2160 5840 1150

DECR2 3783 3080.5 3285 1531.5

DEDD 1040 870 998 1260

DEDD2 1420 1450 2710 1530

DEF6 94.5 61.55 22.95 123.2

DEF8 4895 6200 6405 10100

DEFA10P 3.56 5.55 5.37 4.52

DEFA11P 3.61 5.62 5.45 4.58

DEFA3 9.78 8.43 9.83 4.44

DEFA4 3.26 5.26 4.81 3.96

DEFA5 15.7 4.71 4.38 3.59

DEFA6 3 4.73 4.41 3.61

DEFA7P 10.8 17.5 78.1 21.5

DEFA8P 6.58 6.85 6.92 6.25

DEFA9P 3.45 10.1 4.64 5.29

DEFB1 1080 33.6 41.1 32.2

DEFB103A 4.29 6.31 6.04 5.19

DEFB103B 7.31 9.54 7.78 4.66

DEFB104B 7.38 4.81 5 8.87

DEFB105B 3.87 6.13 5.85 4.92

DEFB106B 3.49 5.59 5.21 4.12

DEFB107A 4.05 5.98 5.45 5.43

DEFB108B 29.7566666666667 29.2033333333333 23.0066666666667 32.2933333333333

DEFB109P1 11.9 8.56 4.94 27.2

DEFB109P1B 3.52 5.69 5.25 4.31

DEFB110 6.305 5.47 5.115 4.235

DEFB112 3.56 5.73 5.33 4.32

DEFB113 8.68 8.75 9.26 11.3

DEFB114 3.85 6.09 5.62 4.53

DEFB115 10 24 12.8 11.8

DEFB116 3.21 5.18 4.76 3.87

DEFB118 3.8 6.02 5.99 4.85

DEFB119 9.265 4.81 9.53 46.405

DEFB121 19.44 21.05 20.42 24.215

DEFB122 5.42 9.14 6.27 9.765

DEFB123 21.1 36.8 26.9 30.3

DEFB124 100 101 113 148

DEFB125 2.61 4.24 3.84 3.21

DEFB126 2.74 4.45 4.03 3.35

DEFB127 2.94 4.75 4.35 3.57

DEFB128 21 24.9 24.2 24.8

DEFB129 14.076 17.29 17.54 16.351

DEFB130 6.185 9.81 16.5 11.02

DEFB131 2.9 4.73 4.29 23.3

DEFB132 46.5 48.8 38.7 52.2

DEFB133 3.11 4.97 4.61 3.77

DEFB134 3.71 5.95 5.45 4.35

DEFB135 3.99 6.18 5.79 4.74

DEFB136 5.92 7.62 13.9 24.4

DEFB4 85.8 66.9 62 53.9

DEFT1P2 3.78 5.95 5.72 4.84

DEGS1 3610 1270 2340 9190

DEGS2 1140 778 660 611

DEK 9290 6180 9680 9010

DEM1 1350 1170 304 575

DENND1A 177 279.5 198.75 166.5

DENND1B 159 185 141 126

DENND1C 36 34.9 46.2 79.7

DENND2A 30.6 26 22.4 48.4

DENND2C 513.566666666667 16.62 4.67666666666667 457.226666666667

DENND2D 3.16 96.8 4.7 3.84

DENND3 175.266666666667 259.966666666667 641.333333333333 452.366666666667

DENND4A 167 162 13.9 56.9

DENND4B 10800 12600 6670 8990

DENND4C 1091.5 588 1035 1063.5

DENND5A 2070 3020 2100 4520

DENND5B 571 1266.5 1520 550

DENR 53.7 74.1 119 283

DEPDC1 1532.6 2525.5 2430.5 5596.5

DEPDC1B 1250 1280 717 881

DEPDC4 80.1 55.4 145 166

DEPDC5 89.7666666666667 88.0333333333333 75.1333333333333 98.9333333333333

DEPDC6 115 62.4 1360 241

DEPDC7 521 322 17.5 818

DERA 5650 8180 3920 3090

DERL1 1160 809 1140 1230

DERL2 4425 5132 7502 3798

DERL3 3458.05 3382.385 3292.44 3447.35

DES 389 360 430 448

DET1 758 204 888 2790

DEXI 3237.55 2862 4480.15 3859.4

DFFA 915.6 1434 1961 2626

DFFB 135.55 110.3 191.5 349

DFNA5 140 1040 4.95 92.9

DFNB31 47.8 37.2 78.5 132

DFNB59 111 27.6 164 82.8

DGAT1 759 898 842 820

DGAT2 186 1070 333 164

DGAT2L6 3.87 6.19 5.84 4.86

DGCR10 4.44 9.04 20.6 21.8

DGCR11 402 694 607 475

DGCR14 168 993 402 589

DGCR2 9270 13500 8930 9060

DGCR5 13.9 6.84 103 32.1

DGCR6L 1200 1350 1630 2910

DGCR8 1250 1220 610 1800

DGCR9 3.84 5.58 18.8 13.6

DGKA 34.5 47.6 87.5 106

DGKB 4.156 5.482 4.894 4.002

DGKD 4670 4220 3440 3160

DGKE 2.86 4.58 4.18 44.9

DGKG 15 19.1 6.42 4.02

DGKH 22.6 6.96 38 19.7

DGKI 3.09 4.99 4.58 4.11

DGKK 8.41 779 6.01 12.5

DGKQ 1760 1030 802 1280

DGKZ 1838.75 1870.5 2045.85 3830.5

DGUOK 4220 6150 4160 5940

DHCR24 18800 18400 8310 7450

DHCR7 19100 28700 9370 15000

DHDDS 139.4 191.4 318.5 136.1

DHDH 100 73.7 173 140

DHDPSL 30.7 15.65 130.75 91

DHFR 1720 1905 4460 4860

DHFRL1 195.5 269 204.5 353

DHH 3.73 7.03 36.3 6.46

DHODH 1340 1880 3770 1930

DHPS 1020 551 1370 1090

DHRS1 428 527 435 436

DHRS11 318 282 782 885

DHRS12 153 167.5 173.5 309

DHRS13 1390 1930 1160 1340

DHRS2 35.8 26.5 1010 198

DHRS3 2720 2730 1680 335

DHRS4 326 1580 979.666666666667 1693.33333333333

DHRS4L1 414.55 2311 1149.5 2064

DHRS4L2 1550 8760 4060 7670

DHRS7 3473 2364 173.9 2391

DHRS7B 1740 914 1820 1880

DHRS7C 3.99 6.19 5.81 4.76

DHRS9 3.32 24 17.5 10.5

DHRSX 773.2 685.75 1375.3 866.45

DHTKD1 447 615 1650 1130

DHX15 14400 16700 7530 10700

DHX16 2610 2250 3090 3160

DHX29 878 1000 1250 1640

DHX30 6031.8 13778 8455 14942

DHX32 591 301 479 595

DHX33 917 820 1000 1210

DHX34 1434.05 1935.9 1584.2 2442.2

DHX35 2330 2210 1810 2270

DHX36 6390 4260 6870 7260

DHX37 2070 2850 3890 6430

DHX38 516 635 796 827

DHX40 101 123 244 429

DHX57 154.25 230.05 194.65 475.9

DHX58 21.6 32.5 86.3 118

DHX8 1710 1370 2010 2560

DHX9 3560 2850 2990 5200

DIABLO 24760 28835 25625 32150

DIAPH1 9642.5 8548.5 8496.5 11561.5

DIAPH2 192 84.8 205 230

DIAPH3 597.5 825 1353.5 1171.5

DICER1 2110 1730 1240 3090

DIDO1 761.5 900.75 777.25 1120.75

DIMT1L 5260 11900 12200 9890

DIO1 10.1 5.15 6.59 3.9

DIO2 9.365 19.53 6.535 13.12

DIO3 9.475 74.1 7.47 18.515

DIO3OS 14.635 50.25 10.9 12.345

DIP2A 104 66.1 140 243

DIP2B 1810 2390 1120 1220

DIP2C 57.8 37.5 321.633333333333 75.6333333333333

DIRAS1 538 277 25.1 2260

DIRAS2 3.77 5.96 5.56 4.53

DIRAS3 3.45 5.55 5.15 27.9

DIRC1 481 3780 2460 1670

DIRC2 647 1588 1258 630

DIRC3 8.54 17.3 5.66 141

DIS3 6.76 30.8 10.8 165

DIS3L 1370 2980 1760 2770

DIS3L2 87.1333333333333 89.6 103.333333333333 85.5666666666667

DISC1 486.066666666667 113.726666666667 55.8433333333333 299.5

DISC2 8.52 4.74 8.9 3.61

DISP1 313 310 96.3 429

DISP2 121 150 314 380

DIXDC1 916 732 351 903

DKC1 11600 13200 9840 22800

DKFZP434H168 2.58 4.18 3.8 3.19

DKFZP434I0714 26.6 27.8 58.6 69.1

DKFZP434K028 7.23 5.84 7.09 8.24

DKFZP434L187 62.3 19.32 5.26 123.135

DKFZP547J0410 10.5 13.4 3.87 31

DKFZP564C152 1880 2320 1440 2960

DKFZP564C196 8.83 4.47 23.9 8.97

DKFZP586B0319 154 166 236 110

DKFZP586I1420 530 304 362 604

DKFZP586K1520 3.75 6.01 5.6 8.14

DKFZP686I15217 5.43 9.19 30.8 9.87

DKFZP761C1711 21.1 23.9 50.8 18.9

DKFZP779L1853 4.09 6.37 5.99 4.94

DKFZp434E1119 3.85 6.01 5.81 4.96

DKFZp434F142 1800 2000 1710 2330

DKFZp434G179 3.23 5.18 4.76 3.91

DKFZp434J0226 3.33 10.6 4.95 4.07

DKFZp434L192 12.5 13.2 13.9 12.1

DKFZp451A211 20.8 19.1 19.6 21.6

DKFZp451B082 2.79 4.5 4.11 3.44

DKFZp547G183 443 370 499 945

DKFZp547J222 33 23.4 16.4 28.2

DKFZp566F0947 3.7 5.92 5.5 4.49

DKFZp566H0824 16.7 22 35.3 15.8

DKFZp667E0512 162 103 94.3 127

DKFZp667F0711 3.12 4.96 7.31 3.75

DKFZp686A1627 3.18 5.17 4.73 3.85

DKFZp686D0853 7.06 4.62 4.23 3.53

DKFZp686F0839 3.05 4.97 4.53 6.21

DKFZp686K1684 2.9 4.65 4.24 100

DKFZp686L13185 2.79 4.54 13.6 11.8

DKFZp686L14188 354 198 116 141

DKFZp686M1136 13.6 80.7 7.75 93.2

DKFZp686O1327 35200 31100 23800 19700

DKFZp686O24166 96.1 228 55.3 1070

DKFZp761E198 59.8 102 107 120

DKFZp779M0652 28.2 13.8 21.5 35

DKK1 21200 28500 21100 117

DKK2 3.6 5.82 5.32 4.24

DKK3 51.05 943.5 277.95 38.4

DKK4 18.8 6.8 10.9 15.3

DKKL1 2.86 21.7 43.7 3.46

DLAT 2550 1840 2160 2570

DLC1 93.6772727272727 139.160909090909 107.164545454545 61.54

DLD 251 876 222 682

DLEC1 3.04 4.9 4.45 4.78

DLEU1 471 2220 3390 3450

DLEU2L 101.66 946.5 571.9 762

DLEU7 33.1 30.5 36.8 48.2

DLG1 1518.5 1533 1795.5 1014.5

DLG2 6.39333333333333 6.32666666666667 28.6333333333333 6.45333333333333

DLG3 567 6.26 1400 2060

DLG4 137 215 568 127

DLG5 2170 2900 918 1540

DLGAP1 3.49 5.67 5.2 4.27

DLGAP2 3.67 5.79 5.53 4.57

DLGAP3 288 60.2 39.5 66.8

DLGAP4 3290 2140 1690 492

DLGAP5 4070 3060 5470 5350

DLK1 3974 7085 35.5 61.5

DLK2 744 1140 21.3 1160

DLL1 2.99 4.87 4.41 245

DLL3 9 218 1580 1870

DLL4 2.56 4.14 3.76 3.16

DLST 2550 3740 3970 6840

DLX1 13.4 199 320 1340

DLX2 46.1 91.4 79.7 3750

DLX3 2.77 4.51 4.09 12.7

DLX4 105 90 27.7 597

DLX5 14.6 30.5 170 1220

DLX6 581 629 1660 801

DLX6AS 79.3 125 114 43.7

DMAP1 520 635.5 401 860.5

DMBT1 3.48 5.385 12.86 5.515

DMBX1 6.64 12.4 28.9 108

DMC1 2.74 64.1 23 322

DMD 402.253333333333 1069.25666666667 8.82333333333333 466.32

DMGDH 55.4 78 13.4 3.55

DMKN 5633.33333333333 421.666666666667 16320 10923.3333333333

DMP1 3.16 5.15 4.69 3.79

DMPK 956 361 804 911

DMRT1 23.2 25.9 13.4 23.3

DMRT2 8.945 22.21 26.68 8.65

DMRT3 3.5 5.06 4.63 20.4

DMRTA1 259 297 262 24.9

DMRTA2 19.1 26.9 33.4 107

DMRTB1 3.45 5.55 5.13 4.18

DMRTC1 3.01 4.9 4.46 3.65

DMRTC2 2.87 4.65 4.22 3.48

DMTF1 715.5 848.6 863.5 1489

DMWD 270 332 385 328

DMXL1 371 366 338 472

DMXL2 39.9 132 76.9 110

DNA2 213 359 211 360

DNAH1 25.4 24.8 6.58 32.8

DNAH10 12.355 10.815 11.73 46.2

DNAH11 511 344 336 250

DNAH12 8.908 46.692 5.822 11.668

DNAH14 679.333333333333 526.033333333333 781.266666666667 1674

DNAH17 6.3 6.37666666666667 6.38 7.49

DNAH2 18.9 113 19.3 32.6

DNAH3 17.6 4.89 4.45 3.61

DNAH5 3.155 5.13 61.6 3.77

DNAH6 7.5025 22.5875 9.0275 9.7825

DNAH7 22.5 16.8 34.6 18.7

DNAH8 6.47 23.2066666666667 6.72666666666667 4.69

DNAH9 3.2 5.16 11 5.38

DNAI1 9.845 22.55 10.68 12.975

DNAI2 4.83 5.445 5.415 4.11

DNAJA1 20000 13400 18000 20700

DNAJA2 554 1270 411 1890

DNAJA3 7439 6315 11310 9324

DNAJA4 14.2 963 61.9 4.76

DNAJB1 3750 2140 3840 3120

DNAJB11 12100 9780 10000 5670

DNAJB12 680 1690 1030 2030

DNAJB13 10.3 17.2 24.6 55.9

DNAJB14 1170 1730 907 915

DNAJB2 3967.5 3462 542.1 1511

DNAJB3 8.68 5.55 10.5 4.09

DNAJB4 298 557 175 362

DNAJB5 244.2 764.5 449 378.5

DNAJB6 3583.33333333333 7497.33333333333 7233.33333333333 8753.33333333333

DNAJB7 2.98 4.85 4.4 3.6

DNAJB8 7.69 4.64 9.95 7.84

DNAJB9 2820 9100 1740 1830

DNAJC1 1850 2205 1720 2900

DNAJC10 3880 2815 3590 4110

DNAJC11 1342.5 1382 1379.5 1660

DNAJC12 27.8 5.11 10 763

DNAJC13 1210 2070 1760 1090

DNAJC14 841.1 1136.1 1323.35 1450.95

DNAJC15 3.08 627 691 9.49

DNAJC16 179 607 482 391

DNAJC17 250 310 290 337

DNAJC18 32.8 126 40 704

DNAJC19 1240.36363636364 986.181818181818 1331 1232

DNAJC2 10800 14600 16200 16300

DNAJC21 1400.5 3133 2099.5 1455.5

DNAJC22 1630 1200 1000 68

DNAJC24 491 879 386 801

DNAJC25 308 520 375 302

DNAJC27 70.55 100.25 109.8 191

DNAJC28 63.1 36.3 83.9 131

DNAJC3 1630 1020 755 1320

DNAJC30 540.5 717.5 809 1001.5

DNAJC4 566 373 533 480

DNAJC5 1432.5 2394.5 1724 1194.5

DNAJC5B 5.89 4.8 4.37 5.5

DNAJC5G 9.11 33.1 18.4 17.2

DNAJC6 321 286 174 2640

DNAJC7 2350 4990 5580 8450

DNAJC8 915 748 1220 1970

DNAJC9 6447 9656 8149 8643

DNAL1 192 691 822 821

DNAL4 133 148 167 181

DNALI1 4.2 10.4 75.7 5.03

DNASE1 151 121 117 151

DNASE1L1 399 326 1200 1130

DNASE1L2 32.6 47.4 49.2 57.2

DNASE1L3 3.26 5.12 4.81 3.9

DNASE2 722 175 581 558

DNASE2B 3.76 5.96 5.52 4.45

DND1 355.55 211.85 726 529.9

DNER 3.5 5.63 5.24 4.35

DNHD1 32.9866666666667 51.2333333333333 40.3333333333333 36.9633333333333

DNLZ 3915 2440 6330 4110

DNM1 28708.4 31510.45 27510.35 31907.55

DNM1L 769.5 1515 1920 1750

DNM1P35 7.13 4.82 5.4 3.65

DNM2 2430 982 3280 2140

DNM3 74.95 10.055 201 210

DNMBP 102 483 626 354

DNMT1 6160 2450 4410 6670

DNMT3A 1496.03333333333 1197 135 318.1

DNMT3B 1019.795 1162.24 858.82 2584.445

DNMT3L 9.84 4.83 4.47 3.67

DNPEP 230.3 319.5 317.5 258

DNTT 3.45 5.62 5.17 4.17

DNTTIP1 4040 3660 6090 7520

DNTTIP2 5424 9107 6744 11490

DOC2A 6.8 9.96 10.1 66.1

DOC2B 68.9 13.25 172 18.7

DOCK1 388 532 1230 729

DOCK10 10.3 13.4 16.8 13.8

DOCK11 77.1 643 63.5 1960

DOCK2 3.31 5.375 5.56 4

DOCK3 135 92.3 140 110

DOCK4 547 962 112 321

DOCK5 125 167.2 145 46.9

DOCK6 384 140 119 291

DOCK7 1526 1993 715 1694

DOCK8 1507.4 274.75 53.195 172.775

DOCK9 99.6 111.133333333333 334.333333333333 181.766666666667

DOHH 1080 629 737 1010

DOK1 117 3240 3050 2740

DOK2 8.51 14.5 7.64 5.48

DOK3 172.7 261.333333333333 225 316.333333333333

DOK4 957 1670 2810 338

DOK5 11.4 4.16 285 12.4

DOK6 217 51 89.3 4.93

DOK7 53.8 7.23 2710 317

DOLK 1263 2323 1994 2028

DOLPP1 3050 5810 2630 4660

DOM3Z 681 1220 555 1810

DONSON 1570 1190 2030 2300

DOPEY1 427 148.6 276 401

DOPEY2 152 66.3 42 59.6

DOT1L 616.5 382.5 343 446

DPAGT1 962 1510 2720 1540

DPCR1 3.49 6.29 12.031 5.418

DPEP1 7.15 20.1 4.97 21.1

DPEP2 7.68 4.8 7.92 6.83

DPEP3 1560 1690 778 1580

DPF1 202 250 320 469

DPF2 3300 1850 5750 2990

DPF3 24.0875 22.3375 18.495 125.5

DPH2 2970 4020 3930 5670

DPH3 10260 7805 4452 5535

DPH3B 6050 3900 2870 3700

DPH5 1090 2040 1630 3440

DPM1 7960 7920 10400 7170

DPM2 1330 1820 2110 3410

DPM3 3127.5 4854 7062 6149

DPP10 3.825 6.01 5.68 4.725

DPP3 8100 5380 1470 1940

DPP4 3170 3120 7980 42.5

DPP6 6.5 4.535 9.165 4.58

DPP7 956 472 1690 83.9

DPP8 3910 5100 2680 3440

DPP9 3095.5 1347.9 1520.5 1556.5

DPPA2 2.83 265 6.57 3.46

DPPA3 9.35 4.65 4.25 13.3

DPPA4 3.17 5.12 4.71 3.88

DPPA5 3.56 5.73 6.51 4.48

DPRX 22.7 16.6 16.3 17.2

DPRXP4 5.76 5.59 228 85.6

DPT 118 107 123 135

DPY19L1 723.5 521.5 911.5 637.5

DPY19L1P1 115 82.3 264 110

DPY19L2 3.75 5.79 5.66 10.9

DPY19L2P1 3.89 6.11 5.71 4.67

DPY19L2P2 3.88 6.12 16.2 16.4

DPY19L2P3 3.73 16.2 182 80

DPY19L3 50.55 96.55 60.85 62.95

DPY19L4 666.5 500.5 1161 390.5

DPY30 6240 8890 10100 10700

DPYD 117.3 394.3 970 4.36

DPYS 2.88 4.62 4.22 3.5

DPYSL2 165 716 2570 414

DPYSL3 34.4 88.4 71 3460

DPYSL4 941.445 797.345 1097.125 990.5

DPYSL5 3.38333333333333 366.68 1015.6 2346.46666666667

DQX1 41.7 30.9 73.8 44.8

DR1 4240 4710 3790 3710

DRAM1 2150 7260 410 33.7

DRAM2 1100 1410 1580 1600

DRAP1 8760 14700 20400 11500

DRD1 3.8 73.5 23.8 41.4

DRD2 2.66 4.31 9.66 14.4

DRD3 14.6 7.98 8.53 8.37

DRD4 449 265 291 539

DRD5 265 34.15 11.565 102.2

DRG1 6680 8750 6950 12800

DRG2 748 3310 1680 3280

DRGX 29.7 19.5 27.2 17.1

DRP2 864 733 613 750

DSC1 3.9 6.1 8.35 4.66

DSC2 142 58.3 27.3 229

DSC3 2.975 4.77 4.355 267

DSCAM 9.165 10.65 15.095 10.065

DSCAML1 3.45 5.61 297 4.07

DSCC1 1045.3 1381 1799 1642

DSCR10 229 205 162 184

DSCR3 675.666666666667 348.033333333333 1028 713.333333333333

DSCR4 11.7 17.6 9.94 15.2

DSCR6 37.2 22 10.4 88.5

DSCR8 6.53 615 5.23 790

DSCR9 122 135 135 92.4

DSE 3180 802 203 6770

DSEL 86.4 55.05 408.5 4.125

DSG1 4.26 5.31 4.86 4.72

DSG2 295 107 819 673

DSG3 3.79 98.9 5.54 4.48

DSG4 3.97 6.17 6.03 4.71

DSN1 2200 3350 3090 3370

DSP 17690 4275 9315 5975

DSPP 7.8 19.045 19.705 17.1

DST 425 272.975 33.37 167.2

DSTN 15200 54100 34400 26400

DSTYK 191 250 476 687

DTD1 4680 7000 10900 8730

DTHD1 3.01 4.83 4.43 3.66

DTL 3024 2658 2406 3369

DTNA 797 584 211.715 672.3

DTNB 12.7 16.2 4.38 69.3

DTNBP1 606.5 369.5 1348.5 1036

DTWD1 640.5 560.5 692.5 560.5

DTWD2 144 145 312 310

DTX1 2.83 4.61 4.18 3.44

DTX2 240 296 265 268

DTX3 27.1 50 11.3 39.6

DTX3L 468 287 122 33.1

DTX4 5.87 97.1 7.83 9.39

DTYMK 35600 43400 34900 32800

DULLARD 3924.5 3996.5 4460 2878.5

DUOX1 4.86 6.16 5.69 6.27

DUOX2 13.6 21.8 20.9 15.3

DUOXA1 56.7 66 56.1 72

DUOXA2 7.33 17.8 17 3.77

DUPD1 8.83 28.9 24.6 6.91

DUS1L 10520 7685 13700 19240

DUS2L 146 374 385 334

DUS3L 7920 2400 2920 8520

DUS4L 176 324 476 288

DUSP1 1395 1710 483.5 1213

DUSP10 297 158 39.3 23.6

DUSP11 456 671.5 501.5 776

DUSP12 7110 7840 7030 8260

DUSP13 29.43 298.6 22 18.282

DUSP14 1940 2490 3090 4970

DUSP15 1420 1620 1460 1440

DUSP16 186 155 67.1 121

DUSP18 38.3 76.6 57.4 147

DUSP19 57.3 29.8 74.1 167

DUSP2 14.6 26.6 20.5 81.2

DUSP21 4.1 5.32 4.84 3.92

DUSP22 279.3 286.7 282.55 506

DUSP23 5820 60.7 492 148

DUSP26 41.8 196 10.7 158

DUSP27 2.75 4.45 125 3.37

DUSP28 607 950 760 1140

DUSP3 3880 3730 2230 2620

DUSP4 348 2210 303 273

DUSP5 75.7 2380 943 78.1

DUSP5P 916 277 267 216

DUSP6 2780 24300 23400 65

DUSP7 6.79 32.9 17.2 27.8

DUSP8 2619.66666666667 1308.73333333333 632.666666666667 1112.23333333333

DUSP9 2240 3360 181 657

DUT 6320 11095 24230 18935

DUX3 4.75 14 4.72 3.77

DUX4 7585.3 9432.95 8987.38333333333 9161.21666666667

DUX4C 4.23 6.38 6.03 5.02

DUXA 12.1 10 13.8 17.6

DVL1 8750.5 5278.5 7628 6881

DVL2 3020 3600 2110 3420

DVL3 1030 1120 677 491

DYDC1 4.23 5.26 4.86 3.99

DYDC2 15.5 5.91 5.5 4.49

DYM 3710 3370 4920 4410

DYNC1H1 12600 15800 12900 13300

DYNC1I1 11.7 122 22.6 83.1

DYNC1I2 6500 5300 2400 5810

DYNC1LI1 573 1470 1020 963

DYNC1LI2 497.5 786.5 1018.5 611.5

DYNC2H1 16.9 5.25 409 2370

DYNC2LI1 130 144 304 273

DYNLL1 19850 70050 66750 60900

DYNLL2 3060 3700 3390 3910

DYNLRB1 2020 3750 3920 2500

DYNLRB2 6.14 4.19 3.81 14.8

DYNLT1 13800 18500 11300 20900

DYNLT3 465.5 13.015 443.5 842

DYRK1A 408.3 420.666666666667 496.933333333333 451.966666666667

DYRK1B 567 237 219 279

DYRK2 608 1300 2110 2100

DYRK3 4.2 75.8 255 314

DYRK4 198 260 1340 1640

DYSF 13.1 22.6 9.73 14.1

DYSFIP1 33.8 18.745 34.3 10.545

DYTN 2350 2430 2570 2220

DYX1C1 21.3 44 132 102

DZIP1 63.04 298.5 42.3 1900

DZIP1L 52.9 102 86.3 170

DZIP3 672.5 792.5 1369 856

E2F1 1051.9 3908 1534 2473

E2F2 2700 1720 10200 4540

E2F3 2377 857 417.35 2160.5

E2F4 458 708 856 944

E2F5 1160 412 1120 1660

E2F6 1471.66666666667 2754.66666666667 1501.66666666667 2185.66666666667

E2F7 694.85 729.1 1398.25 1181.2

E2F8 393 920 1280 681

E4F1 529 602 1160 1350

EAF1 1810 1170 744 722

EAF2 487 636 523 647

EAPP 3250 3020 2980 4390

EARS2 977 743 1930 1370

EBAG9 1650 1100 2500 1480

EBF1 3.58 9.46666666666667 130.4 28.3633333333333

EBF2 8.46 4.47 6.74 19.5

EBF3 3.2 771 4.76 1080

EBF4 55.3 74.3 38.9 89.2

EBI3 20.019 20 103.09 23.77

EBNA1BP2 28460 54660 49680 48010

EBP 40800 109000 15000 28800

EBPL 5525 11505 15585 15505

ECD 2153 2390 2231 2845

ECE1 151.7 225 785.5 117.55

ECE2 4617.43 4818.215 4228.125 2077.67

ECEL1 7.21 19.8 11.3 168

ECEL1P2 17.1 11.4 16.8 20.8

ECH1 1980 5090 6200 1870

ECHDC1 4120 3360 2095 4010

ECHDC2 5080 8870 77.8 48.3

ECHDC3 974 418 34 68.4

ECHS1 13280 16290 14930 13520

ECM1 16.9 29.5 23.9 30

ECM2 3.015 16.96 4.44 3.665

ECSCR 3.78 5.97 5.72 4.81

ECSIT 3710 2330 3200 5110

ECT2 3300 1780 3780 1400

ECT2L 3.95 6.15 5.73 4.66

EDA 31.78 28.315 232 290

EDA2R 5.47 7.105 7.435 102.8

EDAR 3.174 13.879 20.79 3.863

EDARADD 15500 19600 28100 19000

EDC3 1840 1860 611 2420

EDC4 4043 5597.5 3123.5 8191.5

EDEM1 194 196 221 147

EDEM2 773 1170 809 568

EDEM3 2488.33333333333 1131 1159.63333333333 1048.66666666667

EDF1 20100 20700 19800 25800

EDIL3 16.5 23.7 121 59.4

EDN1 386.245454545455 3700.81818181818 201.981818181818 43.5

EDN2 3.44 38.4 162 4.06

EDN3 19.02 18.675 24.33 32.48

EDNRA 19.25 14.375 18.2 93.4

EDNRB 3.22 4.62 5.43 10.5

EEA1 1280 2110 450 1110

EED 5890 8150 10700 11600

EEF1A1 99983.3333333333 110233.333333333 90733.3333333333 83666.6666666667

EEF1A2 2910 4110 1430 4540

EEF1B2 113000 92400 59600 98200

EEF1D 36800 30800 22900 32400

EEF1DP3 1590 1210 922 1180

EEF1E1 10200 8170 13900 6730

EEF1G 89000 72000 76300 149000

EEF2 31900 16700 16300 31900

EEF2K 870 1460 965 2080

EEFSEC 147 187 173 213

EEPD1 1480 933 359 388

EFCAB1 4.435 6.765 8.56 5.045

EFCAB10 3.63 22.5 5.41 267

EFCAB2 643 1130 301 301

EFCAB3 40.1 55.2 92.4 77.7

EFCAB4A 1780 1870 2310 3580

EFCAB4B 74.2 49.5 53.05 113.75

EFCAB5 36.16 43.4533333333333 26.1933333333333 97.6766666666667

EFCAB6 3.5 5.67 5.225 18.4

EFCAB7 286 805 437 1810

EFCAB8 3.67 5.61 5.61 4.9

EFCAB9 2.76 4.46 7.79 3.37

EFEMP1 510 18700 5.64 779

EFEMP2 907 1120 93.8 178

EFHA1 3270 4260 4990 5890

EFHA2 88.1 155 271 205

EFHB 3.32 10.02 10.04 5.185

EFHC1 9.56 17.7 13.8 31

EFHC2 5.36 11.205 6.035 7.85

EFHD1 52.14 44.8 37.8 490.5

EFHD2 2130 2140 3890 3310

EFNA1 803.6 649.3 213 193.4

EFNA2 173 53.4 36.5 36.8

EFNA3 2.7 19.1 25.3 66.5

EFNA4 747 1270 687 377

EFNA5 10.1 15.2 135 84.5

EFNB1 377 72.8 1750 2480

EFNB2 767 72.9 699 1520

EFNB3 9.34 32.4 195 385

EFR3A 2230 3350 3310 2510

EFR3B 6.34 49.34 20.32 530.166666666667

EFS 4.1 184 22.9 9.09

EFTUD1 1054.45 770.15 464.15 1212

EFTUD2 20700 18000 24700 34800

EGF 9.59 126 26.8 11.4

EGFL6 2.72 4.42 10.3 81

EGFL7 464 1160 415 615

EGFL8 514 643 729 1010

EGFLAM 11.8 52.1 28.6 107

EGFR 2018.33333333333 873.016666666667 431.366666666667 96.4666666666667

EGLN1 8970 1570 1910 2810

EGLN2 1270 1170 1650 951

EGLN3 29.5 21.9 11.4 132

EGOT 15.7 12.9 5.22 4.14

EGR1 2230 228 387 151

EGR2 17.5 13.5 10.2 23.5

EGR3 2.55 4.14 3.75 3.15

EGR4 2.96 4.78 4.38 6.85

EHBP1 657.5 1296 376.5 483.5

EHBP1L1 431 222 1170 50.9

EHD1 6100 8430 9510 5190

EHD2 539 200 2140 141

EHD3 17.2 21.2 26.2 369

EHD4 3510 1940 2330 1260

EHF 3.19 5.15 129 5.04

EHHADH 205 203 44.1 43.8

EHMT1 634 726 763.666666666667 756.666666666667

EHMT2 1460 1340 1370 1180

EI24 2420 4090 8640 7700

EID1 49.1 58.5 54.5 133

EID2 1456 3375 3470 3625

EID2B 36.95 180 242.5 210.5

EID3 171 459 195 80.4

EIF1 191000 252000 169000 202000

EIF1AD 4120 4170 3170 4280

EIF1AX 10700 26080 13663.3333333333 25953.3333333333

EIF1AY 3216.99 6753.135 4042.94 6402.405

EIF1B 2330 5910 2630 3730

EIF2A 1443.85 821.25 939 1074.25

EIF2AK1 6280 3120 3700 3760

EIF2AK2 1153.5 1421.5 3089 2146

EIF2AK3 464 290 180 172

EIF2AK4 976 1790 1780 1540

EIF2B1 4250 4706 6696 10114

EIF2B2 4070 4330 6020 8620

EIF2B3 1328.33333333333 2320.83333333333 1610 3471.66666666667

EIF2B4 1310 1510 1520 2110

EIF2B5 1185 1570 2027 1131

EIF2C1 2965 3438 2351.5 2187

EIF2C2 1979 1164 2668 2350

EIF2C3 778 825.5 1059 1415

EIF2C4 92 119 73.5 152

EIF2S1 651 1500 1350 1290

EIF2S2 2187 2860.5 2299 2639.5

EIF3A 51200 70400 47100 75700

EIF3B 40900 25700 20000 24200

EIF3C 95400 89300 134000 156000

EIF3CL 2320 2060 3080 3450

EIF3D 33300 28000 29300 52400

EIF3E 43300 31400 38400 50600

EIF3F 6170 5280 6740 7850

EIF3G 6410 3770 3710 7380

EIF3H 84100 52100 59200 61200

EIF3I 15100 24700 21200 41100

EIF3IP1 8.53 6.53 22.7 7.71

EIF3J 3680 3030 2570 2960

EIF3K 19200 21800 32300 29300

EIF3L 10300 11200 5800 11900

EIF3M 31700 33600 30200 43800

EIF4A1 31370 40266.6666666667 36980 37233.3333333333

EIF4A2 44400 14900 32800 11200

EIF4A3 21800 32100 66000 46800

EIF4B 4790 5000 3356.66666666667 7270

EIF4E 2530.33333333333 3620 1789 3033.33333333333

EIF4E1B 3.92 6.22 5.81 4.74

EIF4E2 16900 14365 12645 15160

EIF4E3 15.1 49.8 29.1 186

EIF4EBP1 17400 15200 14100 18000

EIF4EBP2 11562.7272727273 13539.0909090909 5670 9380

EIF4ENIF1 790.5 737.5 690.5 1009

EIF4G1 6110 5490 7600 6700

EIF4G2 15778.1818181818 26567.2727272727 20613.6363636364 26051.8181818182

EIF4G3 713.5 819.5 1046 1271.5

EIF4H 27300 33200 35500 40600

EIF5 444 506 389 2120

EIF5A 13900 6450 15600 11100

EIF5A2 141 690 117 369

EIF5AL1 221 209 186 157

EIF5B 2942 3563 2559 2856

EIF6 14900 14800 13100 9210

ELAC1 318 469 1050 255

ELAC2 23125 29410 24585 26120

ELANE 8.28 14.7 13.5 14.8

ELAVL1 1080 826 1180 1520

ELAVL2 3.91 6.2 5.67 109

ELAVL3 4.17 6.3 5.96 4.97

ELAVL4 3.72 6.01 30 6.39

ELF1 960.5 763.5 450.5 383

ELF2 125 269 127 220

ELF3 2070 704 1160 5.82

ELF4 2500 4070 3230 4440

ELF5 54.5 8.86 5.07 4.15

ELFN1 11200 13700 15300 11200

ELFN2 27.3 185 75.8 110

ELK1 37.7333333333333 61.7666666666667 55.4333333333333 118.533333333333

ELK3 30.6 81.8 257 53.7

ELK4 243 277.5 239.5 269.5

ELL 247.7 301.5 352 355

ELL2 1140 5110 191 2040

ELL3 200 25 109 309

ELMO1 9.413 80.61 6.844 25.27

ELMO2 535.35 1138.5 1146 1039.5

ELMO3 95.8 59.6 99.1 75.8

ELMOD1 2.91 4.63 4.26 703

ELMOD2 1885.5 3430.5 727.5 2692.5

ELMOD3 258.775 324.675 190.2 505.5

ELN 65.75 75 119.65 76.4

ELOF1 2310 1910 2990 4280

ELOVL1 609 898 1290 689

ELOVL2 16100 2350 5.79 1560

ELOVL3 10.8 21.1 28.2 15.6

ELOVL4 3.86 65.7 5.63 628

ELOVL5 2200 1630 2240 2450

ELOVL6 377.5 419 341.7 444.5

ELOVL7 435 593 689 378

ELP1P 307 361 396 377

ELP2 8540 5260 15100 22900

ELP2P 35.8 8.16 242 66

ELP3 2320 1920 3460 1540

ELP4 1870 2650 3140 2000

ELSPBP1 9.8 4.85 10.1 67.7

ELTD1 5.97 7.42 8.955 7.83

EMB 55.6 167.1 4.31 1255

EMCN 3.27 5.27 4.86 3.98

EMD 249 753 534 2950

EME1 376 592 647 639

EME2 91.1 43.65 68.3 120.45

EMG1 7090 7630 9990 10100

EMID1 59 256 23.4 179

EMID2 30.6 171 569 48.8

EMILIN1 3250 4440 3370 4050

EMILIN2 2180 3420 5.14 4980

EMILIN3 159 173 153 353

EML1 12.7 389 66.1 1105

EML2 207 211 1410 442

EML3 191 150 377 138

EML4 32300 16945 13140 13040

EML5 16.51 12.635 31.23 167.5

EML6 3.57 16.335 5.375 9.6

EMP1 4.3 59.4 19800 169

EMP2 125.845454545455 290.772727272727 2594.54545454545 3483.63636363636

EMP3 128 3590 4860 502

EMR1 53.9 49.3 47.1 60.3

EMR2 3.45 5.46 5.12 25.6

EMR3 2.9 4.66 4.27 5.41

EMR4P 4.22 6.35 6.03 5.06

EMX1 1050 755 1620 1170

EMX2 8.3 4.9 2540 410

EMX2OS 3.16 5.14 2220 425

EN1 14.7 21 13.6 121

EN2 18900 24700 19800 23100

ENAH 9627.5 4347 1265.2 8014.5

ENAM 3.37 5.42 5.03 4.07

ENC1 253 960 268 39

ENDOD1 81 116 3070 1170

ENDOG 1800 1690 3600 1950

ENG 20.3 21.4 17.8 23.8

ENGASE 170.75 193.7 237.3 537.9

ENHO 54.55 81.25 52.65 872

ENKUR 38.5533333333333 72.0733333333333 95.2833333333333 55.3633333333333

ENO1 25500 29300 48300 29800

ENO2 5.97 76.9 332 146

ENO3 2970 5930 408 293

ENOPH1 4246 3476 2731 3691

ENOSF1 1950 4280 5260 1940

ENOX1 3.48 15.2 159 477

ENOX2 465 1000 736 1800

ENPEP 2.94 4.7 4.31 3.57

ENPP1 2411 4875 223.15 2390

ENPP2 806 170 5.12 209

ENPP3 12.5 28.3 4.89 20

ENPP4 1560 293 1410 1720

ENPP5 29.7 7.08 212 71.2

ENPP6 3.56 5.68 5.32 4.2

ENPP7 3.88 6.14 8.5 4.69

ENSA 1910 880 1490 2990

ENTHD1 3.145 5.105 4.645 3.765

ENTPD1 4.94 5.515 5.215 6.685

ENTPD2 175 67.8 93.4 44.9

ENTPD3 3.53 31.5 5.3 26.7

ENTPD4 260.5 630 203 231

ENTPD5 248.2 167.9 119.4 63.31

ENTPD6 3460 5680 3200 2730

ENTPD7 166 249 123 224

ENTPD8 259 9.95 189 21.2

ENY2 27700 21700 24800 21800

EOMES 4.04 6.06 16.6 143

EP300 1080 1060 618 1280

EP400 323 448 375 689

EP400NL 68 77.3 145 124

EPAG 4.19 8.79 6.34 11.9

EPAS1 27600 22000 528 2470

EPB41 47.2 23.6 13.155 74.75

EPB41L1 19 758.9 3552 1681.2

EPB41L2 1600 1330 1210 1420

EPB41L3 5.56 8.61 10.3 1390

EPB41L4A 10.66 5.135 1099.5 119.35

EPB41L4B 1366.365 1552.205 1227.01 467.185

EPB41L5 129 966 1000 841

EPB42 21 21.5 21.6 23.5

EPB49 2233.55 1334.6 6557 392.685

EPC1 782 733 507 565

EPC2 1440 1390 1460 1900

EPCAM 45400 5840 15700 382

EPDR1 1710 1710 9790 1550

EPGN 2.99 4.83 4.44 3.66

EPHA1 158 4.48 7.44 16.6

EPHA10 15.3933333333333 17.0333333333333 17.98 42.2333333333333

EPHA2 543 1040 1880 69.2

EPHA3 3.35 5.42 13.195 136.145

EPHA4 5.297 7.151 482.3 846

EPHA5 4.15 267 7 16.5

EPHA6 3.195 5.16 4.71 13.65

EPHA7 11.8 8.05 17.35 204.5

EPHA8 5.325 7.995 8.15 46.505

EPHB1 3.54 5.66 5.33 12.8

EPHB2 45.3533333333333 46.27 75.8666666666667 168.333333333333

EPHB3 3.53 24.6 4.75 44.8

EPHB4 895.5 1568 1118.5 724

EPHB6 12.8 19.2 8.28 22.5

EPHX1 1960 389 765 616

EPHX2 2080 3150 1550 993

EPHX3 4600 4290 2970 2450

EPHX4 157 339 4.25 660

EPM2A 14.5 15.9 26 13.5

EPM2AIP1 134 111 118 3.38

EPN1 33100 31500 47600 29500

EPN2 950.8 2527 2866.5 2414.5

EPN3 60 183 38.7 3.27

EPO 7.76 17.9 5.31 10.1

EPOR 92.8 75.3 154 153

EPPK1 481 111 56.4 123

EPR1 35.5 55.8 139 75.5

EPRS 26100 9570 21800 26700

EPS15 369 569 302 1190

EPS15L1 801 601 888 1910

EPS15L2 63.9 85.2 31.4 54

EPS8 4960 3200 2100 1120

EPS8L1 163.955 120.01 723.5 484.25

EPS8L2 1070 828 1310 28.3

EPS8L3 3370 6750 464 239

EPSTI1 6.034 25.808 7.567 38.47

EPX 2.81 4.52 6.22 3.45

EPYC 3.85 6.06 5.82 4.94

ERAL1 1510 2520 1970 1990

ERAP1 270.39 822.9 216.85 160.97

ERAP2 44.5 35.2 144 149

ERAS 2.74 4.43 4.02 3.36

ERBB2 215.863636363636 693.754545454545 2741.37272727273 1635.20909090909

ERBB2IP 3270 2730 4740 2850

ERBB3 869.7 595.666666666667 197.933333333333 50.0666666666667

ERBB4 38.3 24.54 6.84 68.32

ERC1 393 383 552 692.5

ERC2 3.06 4.94 4.52 251.5

ERCC1 1560 1960 3355 1740

ERCC2 295.5 311 619.5 325

ERCC3 1590 1740 1850 1790

ERCC4 119.95 138.7 129.75 290.5

ERCC5 2834 877.7 2169 1690

ERCC6 261 192 177 416

ERCC6L 261 937 653 1220

ERCC8 383 648 295 549

EREG 2550 53.1 4.35 3.61

ERF 1610 2150 876 600

ERG 3.8 10.7 5.73 5.56

ERGIC1 3251.25 7143.5 5463 4009.5

ERGIC2 2030 4157 3328 3799

ERGIC3 2950 4420 3540 3460

ERH 18400 22000 18300 27400

ERI1 1100 2160 2650 1090

ERI2 243.433333333333 225.033333333333 309 386.333333333333

ERI3 401 794 943 1390

ERICH1 685 1330 3880 2010

ERLEC1 3505 4270 2700 2475

ERLIN1 214 401 396 343

ERLIN2 406.793333333333 569.793333333333 466.683333333333 506.77

ERMAP 47.4 57.4 71.4 35.3

ERMN 3.385 5.32916666666667 4.91833333333333 4.98583333333333

ERMP1 608 379 555 379

ERN1 21.2 25.4 9.88 16.6

ERN2 17.8 11.4 18.5 16.9

ERO1L 3219 1494 1859 1939

ERO1LB 27.3 89.4 16.8 92.9

ERP27 131 12.2 3.73 3.13

ERP29 3530.5 5002.5 5755 3536

ERP44 99.4 143 95.9 207

ERRFI1 6600 7960 2370 1550

ERV3 32.8 31.5 43.1 37.4

ERVWE1 3.37 5.49 5 10.6

ESAM 45.4 230 65.3 67.8

ESCO1 326 363 392 481

ESCO2 880 1138 1945 604.5

ESD 2400 2800 6140 7180

ESF1 2680 4130 1510 2910

ESM1 3.78 5.96 5.53 4.49

ESPL1 3920 3260 4030 5990

ESPN 418.729090909091 50.4572727272727 294.481818181818 29.3790909090909

ESPNL 38.675 126.565 18.99 801.95

ESR1 13.1975 20.0833333333333 16.4666666666667 18.2908333333333

ESR2 7.52727272727273 8.32272727272727 7.59909090909091 64.7545454545455

ESRP1 212 30.5 28.3 15.5

ESRP2 4188 2888 3529 103.21

ESRRA 7000 3960 4054 3726

ESRRB 36.4 37.5 25.6 65.4

ESRRG 119 23 24.9 32.5

ESSPL 40.5 5.36 4.87 3.94

ESX1 8.69 5.24 4.83 477

ESYT1 1160 1720 2470 1420

ESYT2 468 474 766 2460

ESYT3 90.45 41.75 4.055 3.935

ETAA1 1110 992 1350 1300

ETF1 3220 3380 5090 5810

ETFA 5480 6360 2230 4190

ETFB 16590 51880 34870 17210

ETFDH 2520 2650 2380 3500

ETHE1 278 631 2340 419

ETNK1 659.1 1017.66666666667 1222.33333333333 1074

ETNK2 321.7 754.9 246.95 1493.5

ETS1 3.93 8.44 105 4.91

ETS2 801 650 916 251

ETV1 163.4 248.175 366.925 23.0375

ETV2 155.1 106.05 358.65 215.8

ETV3 44.3333333333333 57.9 43.5333333333333 57.4

ETV3L 2.59 4.2 3.82 5.43

ETV4 77.6 391 1090 17.5

ETV5 39.1 332 800 14.8

ETV6 118.65 104.7 218 123.65

ETV7 17.2 4.46 20.2 6.04

EVC 104 18.1 82.9 71.9

EVC2 78 12.05 5.085 4.14

EVI2A 12.3 6.59 8.95 11.1

EVI2B 6.15 7.76 5.45 11.2

EVI5 138.8 359 222.4 440.5

EVI5L 674 304 823 709

EVL 2270 1720 88.8 5520

EVPL 1040 1020 4230 978

EVPLL 16.165 35 32.95 87.85

EVX1 2420 3140 1870 2590

EVX2 38.1 29.9 30.1 35.9

EWSR1 2363 3155.66666666667 2362.66666666667 4032.33333333333

EXD1 3.65 5.88 11.4 4.45

EXD2 80.8 33 48.6 166

EXD3 43.755 29.605 27.275 42.16

EXO1 1881 1351 1907 4645

EXOC1 299.5 189.5 129.9 358.2

EXOC2 276 200 260 285

EXOC3 1070 883 682 782

EXOC3L 116 131 113 160

EXOC3L2 19.2 21.1 24.7 22.3

EXOC4 60.6 117 156 94

EXOC5 258 380 292 367

EXOC6 2702 2908.5 1468.7 2929.5

EXOC6B 70.05 180 63.75 119.85

EXOC7 2558 2955 4635 4455

EXOC8 693.5 457.5 413 1152

EXOG 90.7 157.9 141.85 361

EXOSC1 3180 5530 4670 6220

EXOSC10 4600 4550 5840 5270

EXOSC2 1030 1740 1435 2465

EXOSC3 2620 6961 3065 4364

EXOSC4 7640 5220 7300 8630

EXOSC5 809 1240 1670 1410

EXOSC6 738 720 1140 1020

EXOSC7 2570 4410 7720 4540

EXOSC8 8980 12700 13000 16100

EXOSC9 5830 5530 4670 9270

EXPH5 156 20.8 307 1110

EXT1 815 485 1150 664

EXT2 2470 3660 3180 4700

EXTL1 13.3 17 24.8 31.5

EXTL2 238 294.2 161 293.8

EXTL3 8310 10700 11100 7860

EYA1 71.55 63.6 44.525 132.45

EYA2 9.71 5.89 9.27 553

EYA3 393.35 436.75 891.6 1180.9

EYA4 3.61 784 41.4 610

EYS 55.436 64.39 40.004 53.174

EZH1 260 285 474 712

EZH2 3230 4040 5460 6870

EZR 7130 13800 11000 5650

F10 849 812 28.7 36.4

F11 615.5 39.89 17.69 15.5

F11R 15000 3470 4027 2228

F12 839 1010 9730 2860

F13A1 7.44 8.77 13.31 11.15

F13B 111 31 5.21 4.13

F2 23570 17420 20.71 10.285

F2R 178.5 244.35 15.965 303

F2RL1 383 633 615 883

F2RL2 3.42 1320 30.7 4.14

F2RL3 583 938 376 1100

F3 654 1490 565 246

F5 240 260 4.79 3.92

F7 2666 381.8 19.75 16.879

F8 38.6 37.5 83.2 87.5

F8A1 1549 2751 2365 5412

F8A2 615 857 970 1780

F9 3.61 5.82 5.38 4.25

FA2H 2.77 6.98 39.5 238

FAAH 70.15 26.6 111 33.25

FAAH2 38.94 11.62 73.42 388.8

FABP1 25200 20000 3.8 3.19

FABP12 3.39 5.51 5.05 8.17

FABP2 3.75 6.06 5.63 5.49

FABP3 16.3 36.2 495 9.78

FABP4 3.69 5.62 5.57 4.93

FABP5 7823.33333333333 15500 4983.33333333333 8460

FABP6 46 17.7 21.4 150

FABP7 2.99 16.1 4.4 94.1

FABP9 2.68 4.33 3.94 37.6

FADD 22400 14000 10200 12400

FADS1 5600 10200 6950 3240

FADS2 1260 14400 1760 1090

FADS3 180 490 682 190

FADS6 5.82 20.2 9.58 15.9

FAF1 2580 4450 4690 5210

FAF2 2400 3680 4030 4800

FAH 2230 2610 1490 2420

FAHD1 989.954545454545 664.181818181818 1073.06363636364 1126.32727272727

FAHD2A 761.633333333333 1614.6 1071.96666666667 1734.83333333333

FAIM 3050 6910 6480 10800

FAIM2 23.6 28.7 28.6 27.8

FAIM3 36 24.9 26.4 31.6

FAM100A 681 854 820 1330

FAM100B 13100 4810 11000 7740

FAM101A 25.24 32.6 52.4 31.49

FAM101B 868 710 7150 3070

FAM102A 2532 2569 7059 1724

FAM102B 92.12 142.7 84.9 490.05

FAM103A1 3530 2820 1450 3370

FAM104A 3990 5780 4310 3630

FAM104B 16.3 15.9 18.1 69.8

FAM105A 334.65 305.15 37.865 149.675

FAM105B 314 262 182 172

FAM106A 76.3 6.57 18.2 20.6

FAM107A 2.54 4.11 12.3 3.13

FAM107B 5206.66666666667 2670 812.666666666667 1013.66666666667

FAM108A1 648.25 486.975 910.725 903.4

FAM108A5 219 238 425 381

FAM108B1 1212.5 1166 1690.5 1737.5

FAM108C1 280 42.4 627 1000

FAM109A 473 739 456 311

FAM109B 50.1 142 58.1 60.2

FAM110A 3590 2280 3320 1840

FAM110B 382 237 7.5 293

FAM110C 177.15 5.34 266.4 17.25

FAM111A 95 187 415 3.73

FAM111B 1190 125 42.7 54.6

FAM113A 146.5 131.1 109.75 178

FAM113B 172.5 614.5 69.5 62.8

FAM114A1 228 474 109 45.5

FAM114A2 163 124 180 374

FAM115A 573.333333333333 1143.66666666667 394.333333333333 818

FAM115C 30.3 20.7 39.1 67

FAM116A 1300 1120 2890 3660

FAM116B 54.6 55 68.3 53.6

FAM117A 1300 1930 2580 3720

FAM117B 1110 546 1910 1540

FAM118A 447.272727272727 478.090909090909 527.545454545455 479.454545454545

FAM118B 382 506 814 638

FAM119A 2966.66666666667 2743.33333333333 2622.66666666667 2616

FAM119B 281 245 332 253

FAM120A 3045 3440 5155 4125

FAM120AOS 2300 3870 4520 5120

FAM120B 334 482 129 456

FAM120C 371.5 694.5 587.5 976

FAM122A 295 569 846 1400

FAM122B 769.666666666667 1160.5 1140.5 4035

FAM122C 17 16.31 14.68 51.3

FAM123A 11.2 4.85 7.26 16.4

FAM123B 277.9 178.835 172.795 650.45

FAM123C 5.44 5.75 5.31 4.28

FAM124A 3.61 5.8 5.39 85.8

FAM124B 14.185 6.7 11.75 11.77

FAM125A 347.9 196.85 417.6 370.05

FAM125B 1267.6 1608.4 44.76 1141.5

FAM126A 386.5 143.37 159 322.35

FAM126B 491 578 566 893

FAM127B 711 917 677 2700

FAM127C 308 479 4.04 1340

FAM128B 50607.5 49479 58651 42469.5

FAM129A 48 10.4 6.03 250

FAM129B 4210 9690 9890 7620

FAM129C 16.25 17.35 13.175 11.79

FAM12A 3.19 5.14 4.68 3.83

FAM12B 2.94 4.71 4.33 5.96

FAM131A 82.8 106 21.6 66.4

FAM131B 3.12 5.09 4.64 3.78

FAM131C 3130 2890 2780 2860

FAM132A 104 54.9 947 73.2

FAM133A 4.09 6.36 5.98 66.6

FAM133B 1736.66666666667 2067 1459.66666666667 3043.33333333333

FAM134A 341 500.5 352 339

FAM134B 3.32 20.8 4.86 552

FAM134C 670 824 749 1070

FAM135A 243.5 743.5 250.5 315

FAM135B 2.86 5.575 4.21 3.48

FAM136A 11180 18320 9115 15140

FAM136B 945 1470 980 1600

FAM138A 203 198 268 362

FAM138D 5.76 4.33 3.93 3.29

FAM138E 11.3 15.5 5.5 4.89

FAM13A 291 549 156 562

FAM13AOS 23.2 11.6 5.43 22.5

FAM13B 469 479 620 857

FAM13C 3.15 4.32 3.94 4.09

FAM149A 250 6.81 330 120

FAM149B1 92.265 383.915 175.215 200.33

FAM150A 135 8.49 4.78 571

FAM150B 6.53 5.095 4.645 15.895

FAM151A 39.4 1853 44.65 35.6

FAM151B 13.1 24.1 77.5 71

FAM153A 38.5 36.3 22.1 14.4

FAM153B 3.75 6.01 5.61 4.51

FAM153C 2.72 4.39 3.99 3.34

FAM154A 9.21 17 20.5 9.12

FAM154B 35.45 6.495 8.9 10.205

FAM155A 45.1 64.9 4630 4.02

FAM155B 552 5.64 1510 1590

FAM156A 2450 3250 5420 8060

FAM156B 137 147 304 431

FAM157A 3.43 5.42 49.1 17

FAM158A 3520 1660 3820 3960

FAM159A 64 22.125 17.25 26.9

FAM159B 43.3 6.9 4.21 596

FAM160A1 11.6933333333333 18.97 52.39 55.5333333333333

FAM160A2 4150 2090 2380 2850

FAM160B1 179 261 120 193

FAM160B2 215.7 454.2 646 225.3

FAM161A 343 523 703 865

FAM161B 15.5 18.2 85.7 54.3

FAM162A 16200 9840 18300 16200

FAM162B 86.3 3260 5.06 4.23

FAM163A 9.63 4.9 4.51 73.6

FAM163B 30.9 25.3 32.8 38.6

FAM164A 507.9 650.5 1447 720.95

FAM164C 132 111 42.8 51.9

FAM165B 1140 750 1350 2580

FAM166A 436 568 527 485

FAM166B 11 8.69 16.1 13.1

FAM167A 2.86 4.655 4.23 5.76

FAM167B 19.5 26.6 57.2 36.3

FAM168A 2.91 7.68 6.98 7.87

FAM168B 923 845 916.5 981

FAM169A 1082 961 1400 1485

FAM169B 7.7 9.58 9.64 28.7

FAM170A 3.3 6.72 7.88 5.1

FAM170B 3.85 4.23 35.5 32.9

FAM171A1 853 1241.5 930.5 1642

FAM171A2 90.3 75.9 59.8 114

FAM171B 28.8 50.1 111.95 102.3

FAM172A 495 554 547 877

FAM173A 2240 1580 4010 3040

FAM173B 1030 1020 1250 836

FAM174A 639 1020 944 881

FAM174B 72.6 556 92.1 199

FAM175A 217.8 472.4 257.4 263.8

FAM175B 360 600 419 714

FAM176A 1287.54545454545 793.272727272727 1017.72727272727 63.2909090909091

FAM176B 8.85 5 4.06 4.35

FAM177A1 6930 3990 3840 2390

FAM177B 261 300 160 218

FAM178A 86.9 205.1 250.666666666667 226.666666666667

FAM178B 229.963333333333 249.303333333333 200.32 193.406666666667

FAM179A 2.83 4.61 4.18 51.8

FAM179B 873 547 146 839

FAM180A 46.5 56.1 81.8 97.2

FAM180B 109 118 147 167

FAM181A 167 167 167 161

FAM181B 15.6 17.7 31.1 44.3

FAM182A 2.87 4.61 4.21 3.49

FAM182B 3.8 6.05 5.75 4.77

FAM183A 5.4 68.9 5.8 73.7

FAM183B 4.79 22 4.49 44.7

FAM184A 1390 349 403 2290

FAM184B 26.4 10.3 15.5 18.9

FAM185A 367 352 871 843

FAM186A 3.23 5.25 9.4 3.94

FAM186B 14.2 17.4 25.6 18

FAM187B 8.46 10.7 8.1 8.82

FAM188A 446.65 428.15 1043 1690

FAM188B 120.45 61.75 109.9 133.85

FAM188B2 3.37 5.41 4.99 4.08

FAM189A1 18.3 16.3 3170 222

FAM189A2 3.93 4.8 15.56 109.8

FAM189B 3970.66666666667 4321 3053.66666666667 7187

FAM18A 5.76 8.52 6.16 17

FAM18B 3200 5610 2870 1960

FAM18B2 1710 3390 725 1310

FAM190A 19.9833333333333 6.84666666666667 4.61 9.19333333333333

FAM190B 395 1056.5 505.5 673.5

FAM192A 556.545454545455 677.909090909091 664 1148.18181818182

FAM193A 315.5 222.9 118.8 203.1

FAM193B 150 199 159 290

FAM194A 7.06 7.36 17.5 8.88

FAM194B 3.19 5.22 4.73 3.84

FAM195A 6390 5610 11675 6485

FAM195B 10700 11600 7530 19800

FAM196A 3.61 5.33 4.86 4

FAM196B 3.35 5.24 5.01 3.96

FAM197Y2 6.44 4.21 3.81 3.19

FAM198A 8.455 9.94 34.7 148.85

FAM198B 91.1 301 4.44 3.61

FAM19A1 3.15 5.085 4.655 4.015

FAM19A2 7.55 5.55 5.19 7.29

FAM19A3 2.63 4.26 3.86 3.23

FAM19A4 155.45 41.48 7.485 4.51

FAM19A5 10.3 10 6.64 7.03

FAM20A 512.4 78.51 38.865 7.415

FAM20B 6830 5220 4310 5740

FAM20C 5130 216 656 258

FAM21C 2960.33333333333 3237.33333333333 2343.33333333333 3020.66666666667

FAM22A 32.15 64.2 47.4 60.55

FAM22B 30.6 80.9 17 25

FAM22D 125.85 85.7 108.135 82

FAM22F 37 208.95 29.25 195.1

FAM22G 169 125 159 139

FAM23A 13.55 7.155 6.505 11.095

FAM24A 3.82 6.11 5.69 4.62

FAM24B 324 899 642 141

FAM25A 23.7 33.1 444 54.1

FAM25C 20.5 10.3 28.7 17.5

FAM26D 10.4 5.24 4.83 3.97

FAM26E 3.04 19.8 4.51 29.5

FAM26F 140 20.3 5.67 45.7

FAM27A 39.86 56.5933333333333 547.353333333333 2470.46666666667

FAM27L 4.67 5.18 4.75 6.31

FAM32A 2960 1350 2540 2740

FAM35A 1403.5 2120 934.5 1369.5

FAM36A 3408 4575 4860 1476.5

FAM38A 1580 563 1130 741

FAM38B 1562 329 16.9 24.835

FAM3A 206 211 120.6 485.5

FAM3B 43.8 14.4 5.06 41

FAM3C 534 1440 2090 1320

FAM3D 13.4 153 7.4 126

FAM40A 398 906.5 754.8 1060.5

FAM40B 27.2 57.4 102 167

FAM41AY1 24.4 5.5 5.08 4.11

FAM41C 25.3 24.3 14.1 26.6

FAM43A 478 47.1 15 148

FAM43B 4.63 161 194 86

FAM45A 649.5 2032.5 1347.5 990

FAM46A 854.5 2095 217.5 604.5

FAM46B 35.9 70 100 36.3

FAM46C 201 227 9.21 1000

FAM46D 3.01 4.76 4.42 3.63

FAM47A 7.775 11.46 10.56 7.31

FAM47B 4.59 14.7 5.24 4.23

FAM47C 5.8 4.65 8.32 5.24

FAM47E 47.6 746 9.65 4.77

FAM48A 343 372 388 447

FAM48B1 61.5 64.9 70.6 86.3

FAM48B2 6.43 5.29 4.91 4.47

FAM49A 4.03 250 9.25 25

FAM49B 5310 3920 9230 4510

FAM50A 2540 3480 1680 5130

FAM50B 1570 2500 1950 804

FAM53A 25.3 4.28 5.99 14.9

FAM53B 375.5 248.926666666667 169.463333333333 506.83

FAM53C 1270 1820 1770 2260

FAM54A 2160 1850 1440 2340

FAM54B 416 531 557 536

FAM55A 4.42 4.18 3.8 3.19

FAM55B 3.4 5.47 5.085 6.37

FAM55C 35 175 55.2 237

FAM55D 7.19 4.66 4.25 3.49

FAM57A 1480 656 3250 844

FAM57B 637 909 679 821

FAM58A 2513.5 4127.5 2716 8640

FAM58B 508 837 639 1980

FAM59A 633 287 2390 1070

FAM5B 3.82 9.39 5.58 4.49

FAM5C 8.521 94.79 6.085 287.2

FAM60A 5185 7115 2635 7380

FAM63A 2030 812 464 1450

FAM63B 348 404 1170 371

FAM64A 895 860 1210 642

FAM65A 2680 3400 4210 1680

FAM65B 3.07 4.995 4.545 3.69

FAM65C 3.125 6.29 237.095 5.72

FAM66A 42.745 41.17 56.83 132.15

FAM66C 3.34 5.295 13.085 84.05

FAM66D 226 210 462 158

FAM69A 816.45 1114 466.95 804.2

FAM69B 1570 3870 597 9070

FAM69C 3.63 5.82 5.4 4.41

FAM70A 13 6.13 5.62 124

FAM70B 10.4 20.3 14.7 17.4

FAM71A 9.01 8.77 11.2 9.89

FAM71B 3.61 5.85 5.37 8.07

FAM71C 8.5 5.08 6.09 8.19

FAM71D 5.68 106 5.71 4.91

FAM71E1 2.83 270 502 1950

FAM71E2 63.805 79.15 90.25 87.68

FAM71F1 14.7 12 27 37

FAM71F2 18.7 13.4 33.7 37.4

FAM72A 971 1250 1770 1540

FAM72D 1410 1760 2290 2280

FAM73A 1070 1940 1190 1940

FAM73B 137 213 355 294

FAM74A1 6.42 5.67 5.245 6.655

FAM74A3 2.72 4.42 4 3.33

FAM74A4 227000 223000 251000 239000

FAM75A2 3.63 5.89 5.38 4.26

FAM75A3 6.51 4.615 9.545 6.425

FAM75B 9.97 4.94 10.7 9.2

FAM76A 90.3666666666667 132.666666666667 201.566666666667 396.3

FAM76B 512 483 1170 1580

FAM78A 27.3 17.7 99.5 171

FAM78B 26.7 63.8 74.6 37.9

FAM7A1 12.6 5.05 33.1 361

FAM81A 15.6 181 26.9 338

FAM81B 3.38 5.45 5.04 4.11

FAM82A1 79.4 133 150 115

FAM82A2 2922 2987 3955 3428.5

FAM82B 2540 997 4110 2210

FAM83A 4.895 4.93 6.36 3.725

FAM83B 90.7 15.6 52.6 77.8

FAM83C 8.23 5.28 16.5 8.31

FAM83D 3860 4990 7450 2920

FAM83E 21.6 47 53.9 35.3

FAM83F 10.5 5.17 16.2 10.6

FAM83G 6085.5 4957.5 1730.65 1062.35

FAM83H 2110 677 3200 555

FAM84A 104.22 191.383333333333 134.45 82.42

FAM84B 10.8 5.54 3610 123

FAM86A 1567.5 1603.5 3237 2179

FAM86B1 27.9 52.5 119 72.3

FAM86B2 2900 2840 7966.66666666667 4530

FAM86D 25.9 55.3 73.8 58.4

FAM89A 5340 4170 1140 5350

FAM89B 3970 2730 3060 2810

FAM8A1 564 159 187 423

FAM90A1 2.63 4.27 100 101

FAM90A10 17.8 24.1 42.3 33.1

FAM90A7 3 4.775 32.08 51.645

FAM91A1 370.566666666667 303.666666666667 423.666666666667 292.666666666667

FAM92A1 131 366 308 2210

FAM92A3 25.9 46.3 53.1 261

FAM92B 3.83 6.12 5.71 4.62

FAM95B1 16.51 42.82 36.865 54.7975

FAM96A 12300 9160 9140 10000

FAM96B 7250 8700 20200 12700

FAM98A 1230 1490 1420 2590

FAM98B 45.06 45.815 46.385 79.55

FAM98C 660 1070 3290 1280

FAM99A 296.155 290.185 264.45 429.515

FAM99B 4.21 6.25 5.94 5.02

FAM9A 3.57 5.8 5.29 4.19

FAM9B 3.65 5.91 13.8 4.48

FAM9C 3.35 5.365 4.98 4.43

FANCA 415.833333333333 724.333333333333 693.833333333333 1257.91666666667

FANCB 181 637 153 485

FANCC 1210 293 1240 2060

FANCD2 1279.93333333333 918.233333333333 1590.66666666667 1289.66666666667

FANCE 4100 4170 5510 9610

FANCF 1415.5 1663.5 452.5 215.1

FANCG 2370 4450 4590 3960

FANCI 3940 5090 3880 6520

FANCL 3370 2700.5 2043.5 4420

FANCM 1830 1800 1800 2840

FANK1 3.47909090909091 5.39090909090909 11.1181818181818 20.7181818181818

FAP 3.28 5.12 4.88 3.87

FAR1 3.085 4.985 261.5 1054

FAR2 8.72 8.54 632 677

FARP1 1491.33333333333 427.333333333333 586.466666666667 309.8

FARP2 39.65 21.55 34.7 76.85

FARS2 494 299 713 511

FARSA 3250 1590 2940 5310

FARSB 1570 2170 1650 2680

FAS 6.71333333333333 26.6408333333333 1822 176.066666666667

FASLG 3.86 5.246 5.181 6.749

FASN 14800 16500 11000 10600

FASTK 9090 21300 15700 23900

FASTKD1 1620 2430 2370 3360

FASTKD2 3970 4020 3840 3880

FASTKD3 1040 1350 1780 1600

FASTKD5 7340 11200 5160 9620

FAT1 5800 2730 2460 1360

FAT2 2.83 4.61 4.18 3.45

FAT3 522.26 702.35 1002.145 1082.82

FAT4 30.5 53.5 513.5 64.4

FATE1 2.82 4.58 4.15 3.46

FAU 110000 80500 92600 137000

FBF1 17.87 28.395 70.05 59.55

FBL 88700 101000 175000 133000

FBLIM1 2030 1293.5 327.6 118.8

FBLL1 1027.5 896.55 1338.6 882

FBLN1 13150 9753.33333333333 783.333333333333 2324.66666666667

FBLN2 29.4 52.3 6.14 46.1

FBLN5 439 619 5.08 688

FBLN7 13.4 21.5 40.4 18.2

FBN1 3.01 4.91 4.46 25.2

FBN2 201 552 846 1000

FBN3 521 5.56 5.13 6.14

FBP1 236 119 5.33 31.9

FBP2 11.7 5.21 5.86 3.94

FBRS 5681 5997.5 8512 15045

FBRSL1 7385 7195.5 4105 7785

FBXL12 325 168 287 298

FBXL13 16.9 24.8 60.3 27.9

FBXL14 44.6 45 68.1 39.5

FBXL15 2580 2460 2980 4610

FBXL16 123 53.1 186 151

FBXL17 71.4333333333333 49.0333333333333 186.466666666667 105.066666666667

FBXL18 4070 2182.5 1934.5 1731.5

FBXL19 704 570 888 1250

FBXL2 99.2 85.5 384 374

FBXL20 95.9 109 198 511

FBXL21 575 93.3 4.3 95

FBXL22 44.9 40.8 82.4 86

FBXL3 665 937 865 3060

FBXL4 2080 1390 1670 1500

FBXL5 2270 4150 1720 2580

FBXL6 3880 4880 7070 3360

FBXL7 25.95 34.95 27.6 39.35

FBXL8 120 105 134 230

FBXO10 427 1870 480 1670

FBXO11 413.5 338 376 391

FBXO15 11.3 14.2 135 22

FBXO16 2.53 333 546 353

FBXO17 285 171.2 1107 339.3

FBXO18 226.9 240.1 660 394

FBXO2 377 3790 232 126

FBXO21 1530 1430 3000 3400

FBXO22 1629 2262 1573.3 2234

FBXO22OS 183 192 139 174

FBXO24 49.985 76.315 79.675 108.82

FBXO25 16300 5960 6360 4100

FBXO27 8.75 50.5 890 108

FBXO28 1335 784.5 949 1925

FBXO3 172.5 126 186 320

FBXO30 1680 2830 830 2370

FBXO31 393.65 314.25 392.35 449.95

FBXO32 47 5.81 81.3 442

FBXO33 493 275 444 900

FBXO34 1920 1330 1600 1890

FBXO36 136.75 149.2 126.55 53.55

FBXO38 698 451 641 689

FBXO39 2.75 4.46 4.04 3.37

FBXO4 256 171 461 212

FBXO40 2.96 4.77 4.34 3.61

FBXO41 938 1280 19.8 960

FBXO42 1410 1420 2130 1370

FBXO43 212 154 245 15.3

FBXO44 78.3 101.2 86.15 110

FBXO46 2200 1810 2460 2630

FBXO47 3.59 5.78 5.3 4.24

FBXO48 45 53.8 34.7 56.1

FBXO5 3520 4090 2570 3060

FBXO6 314 168 36.8 66.5

FBXO7 9940 9420 8940 12400

FBXO8 577 534 240 1080

FBXO9 5020 6000 5290 8360

FBXW10 5.93 34.5 6.91 41.1

FBXW11 9110 8500 6280 8630

FBXW12 30.85 49.7 64.6 76.05

FBXW2 305 706 924 377.5

FBXW4 734 606 1350 715

FBXW5 3030 3470 2970 2870

FBXW7 952 1610 593 1430

FBXW8 102 237 218 141

FBXW9 287 105 609 646

FCAMR 8.255 8.39 16.6 13.65

FCAR 7.99 10.96 11.665 10.55

FCER1A 3.416 5.223 4.782 3.91

FCER1G 1310 1450 1260 1010

FCER2 11.4 4.33 9.12 10.6

FCF1 463 656 582 1020

FCGBP 130 82.8 6940 52.9

FCGR1B 3.59 5.75 5.41 4.4

FCGR2A 15.1 19.645 29.15 30.65

FCGR2B 14.1 4.88 12.4 20.7

FCGR2C 8.64 11.2 20.9 13.5

FCGR3A 3.04 4.97 4.5 3.67

FCGRT 15600 8300 9550 3010

FCHO1 195 503 15500 1570

FCHO2 254.5 355.5 693.5 506

FCHSD1 37.7 30.1 43.3 41.5

FCHSD2 46.9333333333333 157.933333333333 525.466666666667 330.2

FCN1 19.31 18.55 21.45 44.2

FCN2 52.5 10.8 4.48 10.4

FCN3 49.9 39.6 44.8 57.8

FCRL1 14.025 23.17 23.115 18.08

FCRL2 23.22 10.41 4.95 10.68

FCRL3 5.129 14.642 5.978 8.679

FCRL4 3.856 7.016 4.631 4.068

FCRL5 6.97 10.8866666666667 10.64 7.74333333333333

FCRL6 22.705 30.15 25.23 29.17

FCRLA 4.63 8.12 4.565 5.99

FCRLB 18.2 146.5 299.2 357.75

FDFT1 10100 11900 12900 7420

FDPS 47600 46700 21600 20300

FDPSL2A 7690 8590 3810 3590

FDX1 210 173 212 460

FDX1L 3780 2175 3945 4205

FDXACB1 19.7 26 49.6 47.1

FDXR 2360 2180 8060 7340

FECH 411.55 387.9 351.95 770.5

FEM1A 467.8 107.75 162.3 350.45

FEM1B 154 323 135 533

FEM1C 353 323 672 497

FEN1 3480 3860 4410 4970

FER 86.3 70.1 256 245

FER1L4 26.64 31.48 18.325 75.145

FER1L5 22.7 24.1 21.2 19.6

FER1L6 3.33 5.29 8.22 4.01

FERD3L 20.7 13.7 14.8 15.3

FERMT1 141.95 453.05 40.35 416.6

FERMT2 707 1402.5 2073.5 2113.5

FERMT3 16.3 15.7 20.5 14.4

FES 277 387 4.5 42.9

FETUB 188 27 5.55 4.58

FEV 13.5 16.4 18.3 18.8

FEZ1 2.69 4.34 4.44 180

FEZ2 817.833333333333 862.233333333333 1125 1097

FEZF1 2.84 4.38 3.97 3.31

FEZF2 3.95 28.415 6.155 11.535

FFAR1 30.5 32.3 32.5 41.1

FFAR2 23.9 27.8 132 29

FFAR3 8.41 6.02 8.31 9.24

FGA 1501.66666666667 1325.66666666667 5.69666666666667 10.02

FGB 851 9220 5.33 4.34

FGD1 44.6 161 38.4 235

FGD2 23.9 23.3 24.7 21.4

FGD3 10.2 49.8 11 142

FGD4 1439 573.5 2975 217.35

FGD5 3.44 9.91 6.27 25

FGD6 416 517 145 227

FGF1 20.7966666666667 24.9433333333333 35.8466666666667 21.08

FGF10 3.4 5.46 5.03 4.07

FGF11 6.77 49.8 39.3 123

FGF12 176 7.69 4.37 4.67

FGF13 2.64 4.26 3.87 662

FGF14 3.38 5.38 4.99 4.21

FGF16 9.91 11.9 13.7 9.48

FGF17 3.28 5.22 4.85 3.95

FGF18 18.3 16.4 6010 19.4

FGF19 90.1 1080 3.73 3.13

FGF2 39.3 370 131.1 147

FGF20 2.735 4.37 9.435 6.01

FGF21 22.8 5.92 11.1 13.3

FGF22 13.535 9.96 20.905 15.095

FGF23 4.589 5.404 5.187 20.162

FGF3 61.9 57.6 76.7 63.6

FGF4 6.34 8.35 7.83 10.2

FGF5 7.67333333333333 8.87 12 17.4133333333333

FGF6 6.595 5.848 5.672 6.633

FGF7 18.88 6.06 26.74 61.55

FGF8 6.96 6.45 17.4 9.4

FGF9 13.97 22.35 12.97 173.8

FGFBP1 3.42 5.48 122 423

FGFBP2 2.97 4.7 4.36 4.6

FGFBP3 57.6 299 42.8 332

FGFR1 119.666666666667 588.026666666667 544.7 631.3

FGFR1OP 3760 4900 3380 2190

FGFR1OP2 276.1 610.333333333333 468.333333333333 681.333333333333

FGFR2 288.95 13.815 271.5 372.7

FGFR3 9705 7418 3569.5 3089

FGFR4 839 695 660 153

FGFRL1 1840 1960 1240 651

FGG 21400 16800 9.5 13.6

FGGY 226 878 964 407

FGL1 48000 26400 16 10.7

FGL2 2.78 4.5 6.51 3.38

FGR 62.03 18.15 27.77 10.637

FH 5410 2950 5580 3340

FHAD1 3.655 6.45 6.355 22.345

FHDC1 21.4 32.1 36.2 12.7

FHIT 55.9 5.74 49.6 106

FHL1 2120 3470 823 8030

FHL2 82.6333333333333 1537 245.741666666667 2614.58333333333

FHL3 387 345 265 205

FHL5 3.36 4.98 4.52 3.74

FHOD1 154 388 1330 1860

FHOD3 28.5 6.2 145 1180

FIBCD1 103.75 98.3 97.1 106.9

FIBIN 3.86 6.11 5.84 5.26

FIBP 1100 822 1380 848

FICD 153 313 472 286

FIG4 249 150.5 206.1 356

FIGF 42 58.5 16.2 43.8

FIGLA 3.9 6.17 5.67 6.11

FIGN 446 428.4 507 349.7

FIGNL1 375 233 497 547

FIGNL2 861.3 1614 449.633333333333 473.126666666667

FILIP1 3.31 22.15 21.8 23.8

FILIP1L 646.355 322.5 4.895 7.51

FIP1L1 5570 4440 3380 5170

FIS1 2830 7360 5620 5430

FITM1 11.4 4.64 10.3 14.7

FITM2 381 392 221 581

FIZ1 297 292 299 208

FJX1 619 1130 1620 5460

FKBP10 2170 4790 4540 6090

FKBP11 212.5 15700 678 697.5

FKBP14 2460 1660 684 1700

FKBP15 1200 1280 1820 1660

FKBP1A 1483 2578 2838 2210.4

FKBP1B 679 996 97.4 2340

FKBP2 26200 23800 38500 26800

FKBP3 10292 7212 8268 17110

FKBP4 3510 3820 4045 4290

FKBP5 639 349 312 637

FKBP6 6.47 5.01 4.58 4.23

FKBP7 633 1550 81.6 1220

FKBP8 883 744 1290 1190

FKBP9 5851.775 2972.845 4407.62 1572.105

FKBPL 183 183 162 279

FKRP 529 435 404 743

FKSG2 1090 1860 849 1040

FKSG29 3.76 5.87 5.67 4.76

FKSG73 15.9 24.8 24.5 25.7

FKSG83 23.8 23 26.3 20.8

FKTN 25.3 51.8 46.45 53.4

FLAD1 2790 2890 1700 4220

FLCN 434.62 655.203333333333 996.8 424.826666666667

FLG 9.97 10.7633333333333 45.5 11.3966666666667

FLG2 2.93 4.73 4.34 3.58

FLI1 3.21 5.13 4.74 324

FLII 3810 3770 6070 3570

FLJ10038 47.8 44.4 54.3 22.2

FLJ10088 13.5 15 5.05 21.9

FLJ10213 660 406 497 1120

FLJ10357 1110 4170 2640 1240

FLJ10489 2.76 4.49 4.07 3.37

FLJ11235 2.98 4.86 111 4.82

FLJ11292 6254.155 9996.95 7054.515 7904.175

FLJ11710 284 315 501 332

FLJ12334 33.5 30.1 24.7 23.6

FLJ12825 3.84 5.99 16 11.4

FLJ13197 44.2 18.5 10.3 4.69

FLJ13224 24.75 23.9 29.85 28.7

FLJ13439 15.1 75.7 16.7 22.2

FLJ13744 41.7 9.89 4.02 42.5

FLJ13773 139000 144000 158000 157000

FLJ14107 27.1 36.1 38.7 38

FLJ16124 15.6 12.2 4.97 11.8

FLJ16341 3.23 5.22 4.75 4.27

FLJ16734 3.53 4.86 4.41 3.66

FLJ16779 7.05 4.79 4.4 39.3

FLJ20184 12.8 14 11.3 7.54

FLJ21369 13.4 12.9 24.7 19.7

FLJ21408 25 13.1 21.6 23.7

FLJ22184 23095 24915 20035 23585

FLJ22536 120 57.5 5.74 4.9

FLJ22763 17 5.77 5.35 4.36

FLJ23152 8.94 5.4 4.99 78.1

FLJ23834 13.105 8.415 21.3 36.8

FLJ23867 167 167 111 68.1

FLJ25006 86.8 465 561 210

FLJ25328 4.27 6.38 6.09 5.18

FLJ25363 7.94 16.7 8.91 3.89

FLJ25694 269.26 333.2 358.966666666667 360.433333333333

FLJ25758 4.1 6.19 5.89 5

FLJ25917 147 77.8 113 40.3

FLJ26086 3.56 13.8 5.32 559

FLJ26245 2.64 84.3 3.88 3.26

FLJ26332 18.1 9.08 43.8 34.2

FLJ26484 14.7 24.5 31.4 9.75

FLJ26850 2.76 6.04 24.5 16.2

FLJ27255 2.95 4.77 4.37 3.62

FLJ27351 139 148 117 158

FLJ27352 87.1 79.3 146 84.1

FLJ27502 3.61 5.79 5.32 4.27

FLJ30307 3.84 140 5.79 4.84

FLJ30430 4.96 6.05 9.79 7.33

FLJ30679 4.96 5.43 12.3 7.41

FLJ30698 178 166 181 168

FLJ30838 61.4 82.1 49.6 53

FLJ30901 15.9 18.9 13 46.2

FLJ31104 8.34 9.81 72.4 7.57

FLJ31183 3.59 40.1 14 129

FLJ31306 171 159 163 214

FLJ31356 3.11 4.9 16.4 5.04

FLJ31662 2460 3870 1440 2010

FLJ31713 51 39.4 60.1 52.9

FLJ31715 32.5 26.8 54.9 117

FLJ31813 249.7 205.55 163.2 242.65

FLJ31945 3.54 5.72 17.3 4.36

FLJ32063 5.89 5.95 4.05 39.7

FLJ32065 676.66 1682.65 1367.435 3791.995

FLJ32224 6.26 4.85 32.9 12.4

FLJ32255 21.7 38 5.07 13.2

FLJ32742 2.96 4.82 4.38 3.58

FLJ32756 3.62 5.69 5.47 4.55

FLJ32810 135.6 377.333333333333 153.566666666667 109.066666666667

FLJ32955 2.65 4.28 3.89 4.35

FLJ33065 22.1 6.42 8.78 54.6

FLJ33360 11.3 11.7 9.13 19.5

FLJ33534 14.1 4.81 4.39 9.48

FLJ33544 4.21 6.42 6.09 5.11

FLJ33581 4.02 6.21 5.78 4.73

FLJ33630 189.5 193 172.5 147

FLJ34208 163 179 130 127

FLJ34223 3.26 5.26 4.86 3.99

FLJ34503 3.52 5.67 5.27 4.24

FLJ34690 3.47 5.59 6.86 6.34

FLJ34747 2.9 4.69 4.27 3.55

FLJ35024 111 9.39 186 10.5

FLJ35220 61.0333333333333 37.8 172.5 68.5666666666667

FLJ35390 34.56 57.2266666666667 40.0633333333333 45.4266666666667

FLJ35409 2.59 22 24.9 3.19

FLJ35776 2920 1910 1570 1260

FLJ35816 14.7 24.2 33.4 23.2

FLJ35946 37.2 18.8 384 94.8

FLJ36000 61.7133333333333 60.8766666666667 45.49 61.7

FLJ36031 80.3 595 944 2210

FLJ36644 4.89 4.43 22.8 3.39

FLJ36777 182 169 153 156

FLJ36848 10.5 14.3 27.3 4.93

FLJ37035 2.83 4.56 4.16 3.45

FLJ37060 2.93 4.66 5.35 3.56

FLJ37201 24.6 7.99 28 23.1

FLJ37396 4.235 6.795 4.795 23.15

FLJ37505 2.63 7.16 3.87 3.23

FLJ37543 5.13 6.4 9.12 5.09

FLJ37638 3.3 5.22 4.87 3.96

FLJ37644 401 42.2 128 15.7

FLJ37798 441 550 767 570

FLJ38028 3.27 5.24 4.86 3.94

FLJ38109 28.1 38.9 53.5 64.8

FLJ38122 2.88 4.6 175 3.51

FLJ38379 4.6 14.7 4.67 16.6

FLJ38717 30.5 20.5 32.9 65.9

FLJ38723 24.2 18.9 16.7 41.9

FLJ38773 20.7 15.7 10.2 12.6

FLJ39051 10.8 29.8 69.5 43.5

FLJ39061 2.83 4.59 4.16 3.46

FLJ39080 2.55 22.1 3.75 54.9

FLJ39095 26 9.72 9.42 8

FLJ39303 25.1 27 14.8 37.2

FLJ39534 266 5.47 80.4 11.5

FLJ39582 35.9 95.5 98.85 137.25

FLJ39609 31.3 17.4 25.5 131

FLJ39632 285 101 439 728

FLJ39639 14.6 5.51 19.4 26.2

FLJ39739 7.55 6.935 9.395 10.665

FLJ39824 823 721 716 637

FLJ40125 63.15 132.15 844 760

FLJ40292 21.1 25.7 22.3 18.4

FLJ40330 624 826 1460 2210

FLJ40434 1100 1150 913 1020

FLJ40453 3.54 5.71 5.28 4.17

FLJ40504 27200 55700 24700 3630

FLJ40606 17.7 18.7 22.5 29.2

FLJ40712 2.82 4.58 4.16 3.43

FLJ40852 8.53 24.1 4.24 54.2

FLJ41130 2.68 4.36 3.95 3.28

FLJ41170 3.76 19.3 5.81 4.76

FLJ41200 2.92 4.67 4.29 3.55

FLJ41278 4.41 5.51 5.04 4.14

FLJ41309 37.6 16.9 36.6 49.7

FLJ41350 2.88 4.69 4.26 288

FLJ41455 57.5 33.6 90.8 54.3

FLJ41481 43.2 56.7 29.6 55.6

FLJ41562 3.33 5.42 4.95 4.07

FLJ41603 12.3 16.9 82.7 53.7

FLJ41649 13.7 18 11.4 21.2

FLJ41733 18.9 20.7 29.3 7.52

FLJ41856 3.61 5.62 5.12 4.07

FLJ41941 4.12 6.36 6.01 4.99

FLJ42022 30.3 23.5 15.2 17.6

FLJ42094 25.6 42.4 3.86 3.24

FLJ42200 15200 18900 15300 19000

FLJ42258 14.1 27.4 13.5 14.5

FLJ42289 7.415 10.755 7.265 35.65

FLJ42351 5.73 5.54 7.92 4.17

FLJ42392 61.7 71.6 48.3 55.6

FLJ42393 74.15 93.65 75.25 51.96

FLJ42418 36.025 40.355 34.37 47.355

FLJ42627 107.45 164.5 92.8 226.5

FLJ42709 74.52 337.7 178.55 175.865

FLJ42842 3.3 5.33 4.92 4.02

FLJ42875 115.235 19.345 15.2 55.54

FLJ42969 2.87 45.4 4.23 3.48

FLJ43315 426 152 70 679

FLJ43390 3.03333333333333 4.89333333333333 4.47333333333333 3.70666666666667

FLJ43585 2.61 5.27 12.8 3.21

FLJ43663 88.8 135 84.9 269

FLJ43681 45700 50500 41000 46000

FLJ43859 3.64 5.71 5.5 4.58

FLJ43860 136 174 126 135

FLJ43879 6.47 5.83 5.41 4.38

FLJ43903 3.57 5.74 5.32 4.33

FLJ43944 17.5 14.7 26.9 28.3

FLJ44006 3.8 6.03 5.56 4.5

FLJ44054 7.83 17.2 5.47 10.2

FLJ44082 4.06 6.22 5.78 4.77

FLJ44087 3.62 5.85 5.41 4.41

FLJ44124 44.5 73.5 55.2 53.2

FLJ44253 377 238 79.9 258

FLJ44255 4.16 6.4 6.05 5.04

FLJ44342 2860 1480 1480 7570

FLJ44385 56.7 32.1 43.6 58.9

FLJ44450 3.09 4.98 4.55 3.76

FLJ44477 211 230.455 242.715 247.305

FLJ44511 2.73 5.67 4.01 3.34

FLJ44606 34138.6666666667 42139.6666666667 76761 71183.6666666667

FLJ44635 3.29 5.35 4.92 3.99

FLJ44674 2.72 4.4 4.02 3.34

FLJ44715 4.03 6.415 8.495 5.26

FLJ44817 11.8 9.33 7.93 7.67

FLJ44874 2.75 4.46 4.04 3.37

FLJ44896 3.67 5.9 5.48 4.44

FLJ45079 9.35 8.03 9.37 10.2

FLJ45121 61.7 59.8 83.4 106

FLJ45139 3.67 5.84 5.43 4.43

FLJ45244 39.2 5.25 161 296

FLJ45248 187 356 221 152

FLJ45256 13.2 17 4.49 5.73

FLJ45445 2022.66666666667 2447 2198 3556.66666666667

FLJ45482 594.35 325.75 464.5 650.5

FLJ45671 5110 5100 5470 7800

FLJ45721 21 5.4 4.95 3.99

FLJ45872 2.79 4.49 4.09 5.69

FLJ45949 5700 6910 6650 6910

FLJ45950 3.58 5.56 5.4 23.1

FLJ45974 2.75 49.1 4.04 3.37

FLJ45983 430 131 10.6 36.5

FLJ45994 17.6 34 5.06 17.2

FLJ46010 3.60666666666667 5.21333333333333 4.81666666666667 3.95666666666667

FLJ46020 257 256 174 151

FLJ46066 3.32 5.34 4.94 4.04

FLJ46111 9.66 14.5 37.7 13.8

FLJ46120 2.73 9.14 4.01 4.79

FLJ46134 17.2 14.3 10 19.3

FLJ46284 2.97 4.82 4.37 3.63

FLJ46320 2.62 4.25 3.85 3.22

FLJ46321 4.17 6.4 6.05 5.06

FLJ46358 10.9 8.98 578 30.6

FLJ46361 3.3 5.36 4.87 9.86

FLJ46365 3.05 4.89 4.51 3.72

FLJ46446 7.32 15.5 6.02 16.6

FLJ46552 4.95 10.3 5.64 14.2

FLJ46875 33.1 31 12.4 26.2

FLJ46906 814 400 1170 2090

FLJ90680 2.65 4.28 3.89 3.27

FLJ90757 363 854 1500 1240

FLNA 1817 4485 3294.5 2244.5

FLNB 2910 2380 518 1330

FLNC 67.6 311 286 128

FLOT1 4640 4660 5030 2200

FLOT2 1438 2979 3366.5 1397.2

FLRT1 4.3 4.87 4.16 187

FLRT2 212 56.5 96.4 3.33

FLRT3 1050 2840 1340 584

FLT1 3.56 5.67 13.9 17.6

FLT3 8.06 7.05 5.54 23.8

FLT3LG 15.2 8.49 4.41 12.2

FLT4 37.2 24.2 33.3 855

FLVCR1 5780 3010 2244 2814

FLVCR2 89.2 77.1 10.7 29.4

FLYWCH1 235.03 127.5 439.3 205

FLYWCH2 25.2 102 182 189

FMN1 64.5 83.1 105 61

FMN2 3.91333333333333 6.61 5.39666666666667 24.8333333333333

FMNL1 29.9 20.5 76.05 16.3

FMNL2 1510 1040 1090 721

FMNL3 79.29 113.37 129.85 81.165

FMO1 666 37 31.8 28.9

FMO2 3.8 5.95 5.74 4.83

FMO3 3.22 5.18 4.78 3.93

FMO4 186 56.4 5.43 109

FMO5 268.95 196.3 7.96 44.35

FMO6P 30.4 18.9 17.8 14.6

FMO9P 2.76 4.46 4.04 3.37

FMOD 6.118 655.9 350.7 35.84

FMR1 587.5 1290 598 2185

FMR1NB 6.02 15.3 12.1 13.8

FN1 401 975 5.2 10.6

FN3K 36.9 24.9 18.8 10.6

FN3KRP 3110 6750 10300 7130

FNBP1 572 468 83.9 708

FNBP1L 5140 4710 2530 2560

FNBP4 1200 1280 1040 1760

FNDC1 2.975 4.83 4.405 17.92

FNDC3A 956.45 1622.845 1080.6 1846.55

FNDC3B 4205 2820 1974.5 471

FNDC4 188 52.4 5.28 176

FNDC5 18.3 15.2 5.7 66

FNDC7 3.74 5.11 4.74 5.31

FNDC8 2.91 4.75 4.15 6.18

FNIP1 537 459 441 289

FNIP2 1600 2820 475 1320

FNTA 9090 8170 5280 6330

FNTB 903 522 847 1400

FOLH1 49.3 64.095 9.465 36.7

FOLH1B 36.4 64.2 4.61 32.1

FOLR1 71.4 97.8 1130 445

FOLR2 30.85 29.25 37.4 34.8

FOLR3 15.4 12.6 39.7 5.96

FOLR4 21.2 13.7 24.8 32

FOS 42.6 78.3 1010 74.2

FOSB 25.8 43 57.7 56.5

FOSL1 53 210 1170 37.6

FOSL2 34.9 57.3 939 25.9

FOXA1 3107 3563 350.3 373.2

FOXA2 5840 8130 878 3.45

FOXA3 1000 929 42.8 62.4

FOXB1 1200 1470 1320 1110

FOXB2 21.5 8.21 10.5 8.69

FOXC1 159 99.5 1160 5220

FOXC2 2.63 4.27 3.86 3.24

FOXD1 12.2 59.5 1070 2790

FOXD2 15.5 183 48.7 154

FOXD3 2.71 4.39 3.99 693

FOXD4 62.6 90.2 166 638

FOXD4L2 4.27 6.53 21.1 29.2

FOXE1 8.73 215 3.94 32.1

FOXE3 72.6 244 86 94.9

FOXF1 14.06 13.345 12.21 210

FOXF2 3.198 252 14.297 788.3

FOXG1 2.96 211 1870 3970

FOXH1 93.8 128 124 143

FOXI1 2.91 5.54 8.15 3.52

FOXI2 36400 33800 27300 20900

FOXI3 48.1 65.1 30.1 71.8

FOXJ1 155 59.8 29.3 4.15

FOXJ2 21.3 25.8 107 82

FOXJ3 2660 3180 3100 3660

FOXK1 1060 725 1130 1200

FOXK2 1267.33333333333 1696 1923.66666666667 1869.33333333333

FOXL1 7.55 4.47 40.2 51.6

FOXL2 3.58181818181818 74.5172727272727 5.55545454545455 4.32909090909091

FOXM1 3700 3360 5790 3800

FOXN1 34.1 57.1 53.8 68.4

FOXN2 4995 4010 2110 1435

FOXN3 509 361 135 391

FOXN4 3.68 22.5 5.55 241

FOXO1 1050 777 1220 1020

FOXO3 2407.66666666667 1306 627.1 3938.66666666667

FOXO4 59.7 22.895 39.6 103.1

FOXO6 19.5 15.9 26.4 27.7

FOXP1 5527 4217.33333333333 1661.1 3687.66666666667

FOXP2 14.0333333333333 57.9 6.82 34.5666666666667

FOXP3 10.795 13.695 10.745 17.25

FOXP4 35147 41674 37697 40824

FOXQ1 4990 12800 644 72.7

FOXR1 3.26 53.7 4.87 5.55

FOXR2 6.68 15.5 9.36 4.7

FOXRED1 259 493 856 468

FOXRED2 826.45 600.4 745.5 1721

FOXS1 4.59 4.22 3.75 4.29

FPGS 511.5 519.5 962 762.5

FPGT 229 353.5 371.5 572

FPR1 26.6 23.8 25.6 23.3

FPR2 3.59 5.57 5.44 4.73

FPR3 10.9 5.68 13.8 12.4

FRAS1 4354.95 1054.35 537.75 927.35

FRAT1 178.8 219.8 45.85 98.4

FRAT2 7070 8320 785 5930

FREM1 42.9 8.08 8.55 70.6

FREM2 106 6.32 86.7 152

FREM3 4.19 6.35 6.01 5.02

FREQ 270 420 710 645

FRG1 2950 2540 4770 3810

FRG1B 1655 3095 1142.5 2000

FRG2 9.54 5.13 15.3 16.4

FRG2B 9.64 7.13 5.26 4.24

FRG2C 9.025 5.09 4.93 8.53

FRK 434 858 187 31.9

FRMD1 83.2 67.4 67.9 75.8

FRMD3 120.7825 700.125 445.075 43.445

FRMD4A 8.675 9.28 336 46.9

FRMD4B 57.6 127 1210 242

FRMD5 4.18 6.42 93.8 178

FRMD6 279 336 680 590

FRMD7 3.84 6.05 5.61 4.54

FRMD8 2491.35 1509.8 2411.5 1595.2

FRMPD1 2.56 6.21 4.71 5.75

FRMPD2 2.68 4.34 3.93 3.28

FRMPD3 4.83 39.6 4.63 3.75

FRMPD4 6.045 9.43 17.235 10.45

FRRS1 963 561 139 277

FRS2 410 574 303 545

FRS3 43.5 22.9 18.3 21.8

FRY 540 295.743846153846 53.2776923076923 62.5492307692308

FRYL 857 682 620 514

FRZB 44.8 54.9 34.8 28.5

FSCB 2.79 4.51 66 3.45

FSCN1 30600 22200 24000 11600

FSCN2 59.5 49.7 82.2 62.9

FSCN3 15.1 11.6 11.3 9.53

FSD1 51.7 438 820 1220

FSD1L 51.7 94.6 249 57.9

FSD2 25.1 32.4 33.7 33.8

FSHB 3.43 5.58 5.13 29.9

FSHR 11.3490909090909 12.0663636363636 13.2063636363636 9.13

FSIP1 10 15.2 24.4 43.4

FST 808 1766 4.752 7.972

FSTL1 56.6 562 536 5200

FSTL3 669 693 4900 214

FSTL4 6.21 12.2 7.22 4.05

FSTL5 3.56 69.6 5.31 4.33

FTCD 768 19.5 21.55 27.8

FTH1 190333.333333333 169666.666666667 174666.666666667 74833.3333333333

FTHL17 81600 56900 58100 14700

FTL 148000 94100 75500 6145

FTMT 84 68.1 64.8 48.1

FTO 797.981818181818 1349.36363636364 2144 2551.63636363636

FTSJ1 3030 16100 5120 8010

FTSJ2 960 751 695 1060

FTSJ3 6040 7480 6750 10600

FTSJD1 228 263 958 483

FTSJD2 7925 534.5 471 986

FUBP1 2130 4365 4200 4085

FUBP3 987.666666666667 825.666666666667 708.333333333333 1143.33333333333

FUCA1 870.1 713.4 597.1 1307

FUCA2 6955 6440 3083 3908

FUK 899 850 831 909

FUNDC1 904 1650 1350 2090

FUNDC2 742.35 950 320.2 1765.4

FURIN 645 1260 174 130

FUS 4639 2932 3130 6787

FUSSEL18 3.51 5.64 5.24 4.14

FUT1 392.5 24.17 87.91 65.49

FUT10 275 149 174 179

FUT11 306 632 669 358

FUT2 6.23 5.1 4.64 3.74

FUT3 24.6 1830 5.41 4.28

FUT4 697 101 945 530

FUT5 2.88 28.3 4.24 50.3

FUT6 110006.8 117547.75 136002.11 131004.2

FUT7 33.1 40.3 37 43.8

FUT8 544 1830 1500 910

FUT9 4.205 9.28 24.715 4.09

FUZ 196 65.7 74.5 1550

FXC1 3420 4200 2930 6450

FXN 2445 1855 3855 4610

FXR1 9740 7325 9440 5690

FXR2 59.7 248 248 364

FXYD1 62.3 47.6 60.2 56.2

FXYD2 18.85 13.85 13175 17.85

FXYD3 13.515 15.0425 18.75 17.1075

FXYD4 15.9 11.3 20.9 15

FXYD5 17 26.8 2320 924

FXYD6 41.6 36.7 380 324

FXYD7 18.4 18.3 15.3 14.7

FYB 3.68 5.67 32 4.87

FYCO1 635 1640 1230 1060

FYN 10.746 354.3 557.3 4431

FYTTD1 784 1680 1860 1340

FZD1 322 416 3220 623

FZD10 2.72 4.41 4 450

FZD2 82.8 261 1920 1070

FZD3 6.13 632 159 876

FZD4 1880 3900 6710 1130

FZD5 9181 3116 854.1 689.5

FZD6 281 169 884 552

FZD7 83.8 319 469 1260

FZD8 127 89.8 172 518

FZD9 168 240 438 626

FZR1 1060 714 805 920

G0S2 211 282 4.45 238

G2E3 528.65 3597 405.55 647.5

G30 3.53 5.68 5.31 4.38

G3BP1 6603.5 7316.5 13605 13066

G3BP2 10800 10400 6820 12600

G6PC 10.7 4.8 4.35 3.57

G6PC2 3.8 6.02 5.55 4.5

G6PC3 123 220 445 292

G6PD 315 1660 221 273

GAA 10800 15000 15900 14000

GAB1 88.9 68.4 11.9 28.2

GAB2 537 994 1330 164

GAB3 3.74 5.945 5.49 5.715

GAB4 2.57 4.17 3.78 3.17

GABARAP 19700 16200 9330 9000

GABARAPL1 390.65 2660.5 714 1587

GABARAPL2 2960 5460 4100 6990

GABBR1 10.885 34.11 18.965 106.65

GABBR2 3.12 5.06 4.59 98.9

GABPA 711 672 731 1820

GABPB1 777 1176 984 805

GABPB2 431 189 524 679

GABRA1 2.91 4.63 4.26 3.53

GABRA2 660 825 4.52 3.66

GABRA3 43.8 25 25 53.6

GABRA4 2.78 4.5 4.08 3.39

GABRA5 3.18 4.35 3.94 3.3

GABRA6 9.23 4.82 4.39 3.58

GABRB1 64.1 92.3 5.87 4.87

GABRB2 37.9 5.24 12.9 3.48

GABRB3 6.32 10.3 12.49 8.545

GABRD 6.79 5.3 11.2 16.7

GABRE 35.4 909 20.5 57

GABRG1 3.41 42.1 5.07 4.14

GABRG2 65.5 61.9 33.2 58.6

GABRG3 3.76 5.94 5.53 4.49

GABRP 30.6 27 26.5 27.1

GABRQ 6.28 5.335 16.795 27.65

GABRR1 2.85 51.6 11.7 3.2

GABRR2 2.73 4.44 4.03 3.34

GABRR3 12.7 14.5 25.2 11.6

GAD1 215.1 22.235 4.98 390

GAD2 4.635 5.96 6.57 8.515

GADD45A 2190 3080 1310 2380

GADD45B 4340 5540 1830 1590

GADD45G 588.5 402.2 59.4 331.55

GADD45GIP1 7440 3090 6300 7900

GADL1 4.02 6.3 5.91 4.85

GAFA1 3.22 6.635 7.885 4.92

GAFA2 22.4 24.3 21.9 17

GAFA3 10 8.61 4.66 19.3

GAGE1 9.64 4.74 4.33 4.37

GAGE7 13.8 15.1 22.9 75.8

GAK 3090 2390 1200 2180

GAL 164 19100 1920 33600

GAL3ST1 556.8 3784 11040 237.3

GAL3ST2 25.8 27.6 17.9 16.5

GAL3ST3 2.54 4.11 3.73 3.14

GAL3ST4 17.4 21.9 13.3 11.5

GALC 842.4 985.6 5.74 228.25

GALE 549 607 1280 197

GALK1 1300 943 818 1060

GALK2 2140 2480 1160 1560

GALM 944.333333333333 832.666666666667 1833 1087

GALNS 227 325 311 217

GALNT1 5615 1945 294 2350

GALNT10 913 2100 2590 638

GALNT11 185 425 471 854

GALNT12 22.4 5.4 81.3 148

GALNT13 45.1 20.4 22.1 19.2

GALNT14 2.91 4.71 15900 360

GALNT2 761 837 522 850

GALNT3 141 4.68 39.1 38.9

GALNT4 47 79.1 278 76.6

GALNT5 3.09 88.4 17.2 48.6

GALNT6 4.09 14.7 5.81 445

GALNT7 1110 3100 1060 2080

GALNT8 3.8 5.98 5.56 38.7

GALNT9 7.6 5.29 4.87 5.34

GALNTL1 29.5 22.3 17.3 1180

GALNTL2 3.28 5.25 6.84 3.97

GALNTL4 4.1 7.38 842 935

GALNTL5 2.76 4.47 4.06 19

GALNTL6 3.49 5.675 5.21 5.775

GALP 3.47 5.62 5.16 4.23

GALR1 4.09 6.29 5.92 4.89

GALR2 15.5 20 16.5 11.5

GALR3 21400 23700 22800 24800

GALT 457 377 157 475

GAMT 2090 2020 104 827

GAN 92.3 216 82.9 294

GANAB 6470 7870 6470 7810

GANC 398 122 380 275

GAP43 2.9 4.64 4.24 105

GAPDH 61020 88820 116700 62730

GAPDHS 3.04 4.96 4.49 3.67

GAPT 14.3 52.4 46.2 12.8

GAPVD1 379 606.5 466.5 856.5

GAR1 11500 16800 13400 29900

GARNL3 42.075 22.43 50.25 125.485

GARS 7660 2820 2370 4430

GART 5260 3560 7005 9405

GAS1 8.27 4.75 97.2 1560

GAS2 2590 1410 4.97 4.04

GAS2L1 804 1480 1350 258

GAS2L2 15.75 26.42 30.385 7.05

GAS2L3 607 2630 2290 350

GAS5 69400 68500 37000 72900

GAS6 3.37 5.41 5 30.7

GAS7 3.13 4.84 4.64 13.8

GAS8 380 1040 1450 1490

GAST 35.2 41.7 38.1 33.3

GATA1 4.58 5.82 7.92 14.4

GATA2 397 611 331 407

GATA3 311 160 4.23 551

GATA4 701.333333333333 730.666666666667 322 298

GATA5 3.82 6.14 5.74 99.6

GATA6 1420 883 403 1730

GATAD1 772 571 963 1340

GATAD2A 1870 3135 1995 2300

GATAD2B 178 288 190 664

GATC 287 536 564 2030

GATM 134 2360 5.81 7.17

GATS 211.333333333333 644.466666666667 303.5 232.633333333333

GATSL3 220 224.5 2345 620

GBA 2380 1830 1070 1050

GBA2 303 335 224 390

GBA3 173 269 13 11.5

GBAP 763 580 406 362

GBAS 1040 432 1140 1560

GBE1 1996 2066 2012 1389

GBF1 3790 7180 4080 5810

GBGT1 146.25 169.85 172.55 161

GBP1 3.58 138 5.32 9.63

GBP2 81.1 748 4.12 3.34

GBP3 3.45 47.2 35.8 4.22

GBP4 67.7 133 83.5 113

GBP5 11.2 17.8 9.04 15.3

GBP6 3876.425 3727.325 4002.105 3606.735

GBP7 4.04 4.31 3.94 3.3

GBX1 3.46 5.58 5.17 35.2

GBX2 7.44 7.54 44.4 188

GC 59500 5440 5.41 4.34

GCA 3430 686 2230 2130

GCAT 343 529 338 397

GCC1 633 672 595 766

GCC2 1104.66666666667 1218 721.666666666667 1118.66666666667

GCDH 1440 700 1300 2170

GCET2 13.595 15.145 24.18 20.56

GCG 36.625 45.09 47.37 43.135

GCGR 11.7 67.9 8.39 3.48

GCH1 431 392.5 56.3 284.5

GCHFR 4250 4200 3420 1120

GCK 82.7 71.5 86.9 80.6

GCKR 92.48 6.506 5.296 4.369

GCLC 9840 5010 2470 2700

GCLM 7990 10200 8130 8550

GCM1 33.9 32.6 55 76.8

GCM2 3.79 6 5.73 4.81

GCN1L1 19100 17700 24500 29600

GCNT1 8.86 90.7 136 301

GCNT2 230 127 119.6 16.475

GCNT3 66.2 328 8.21 4.45

GCNT4 30.33 21.86 5.303 5.042

GCNT6 11.2 4.95 8.6 3.66

GCNT7 2.78 4.51 5.34 3.88

GCOM1 352.7675 369.5875 207.15 365.265

GCSH 26300 23000 28000 31000

GDA 753.5 1028 4345 3.91

GDAP1 31.4 38.6 32.1 285

GDAP1L1 6.815 7.03 9.37 9.835

GDAP2 241 349 200.272727272727 301.636363636364

GDE1 1490 1430 2670 2960

GDEP 2.88 4.63 4.22 3.5

GDF1 1080 1130 783 575

GDF10 4.03 27.9 4.64 69.2

GDF11 434 682 269 1200

GDF15 116000 29200 4080 134

GDF2 15.4 11.8 14.8 12.3

GDF3 5.72 6.33 5.99 6.46

GDF5 54.1 47.3 61.1 62.5

GDF6 4.49 8.32 6.22 39.8

GDF7 50 55.3 64.7 55.2

GDF9 3.3 5.32 17.2 5.6

GDI1 1400 3000 2050 5380

GDI2 2275 1853 3920 3005

GDNF 19.1533333333333 5.64333333333333 5.24333333333333 11.55

GDPD1 34.9 65.5 13.6 250

GDPD2 3.52 5.19 4.74 4.71

GDPD3 154 11.6 10.8 29.8

GDPD4 19.79 45.85 14.415 22.505

GDPD5 53.55 47.75 116.5 51.1

GEFT 27.15 164.35 10.14 267.1

GEM 6.88 354 140 103

GEMIN4 3530 3560 5420 6700

GEMIN5 1920 2900 4360 3430

GEMIN6 10300 10200 6490 22700

GEMIN7 4330 4050 3710 6020

GEMIN8 161 489 228 442

GEN1 509 261 423 289

GFAP 2.88 4.66 4.245 5.23

GFER 442 474 333 1060

GFI1 694 233 22.9 337

GFI1B 188 92.9 245 62.3

GFM1 4170 3170 6900 3860

GFM2 3452 2917 5007 4749.5

GFOD1 1738.18181818182 812.181818181818 642.454545454545 2704.54545454545

GFOD2 1070 1220 1200 1880

GFPT1 1653.5 3912 3495.5 895.5

GFPT2 3.48 12.7 51.2 924

GFRA1 649 845.5 275.8 626

GFRA2 64.3 59.9 82.2 65.2

GFRA3 221 83.7 219 76.3

GFRA4 76.1 83.1 43.8 38

GFRAL 2.61 4.24 6.31 3.21

GGA1 5168.33333333333 3293 3161 4461.33333333333

GGA2 1160 895 796 2280

GGA3 1700 1540 1820 2050

GGCT 23300 17200 18500 19800

GGCX 2310 2080 539 1120

GGH 17500 18700 12500 15000

GGN 345 207 199.25 308

GGNBP1 31.7 20.1 22.9 28

GGNBP2 2990 2810 2490 2020

GGPS1 89.1 77.4 47.1 186

GGT1 234.6 568 337.5 277.5

GGT3P 52.5 262 146 85.2

GGT5 27.7 64.9 27.1 34.5

GGT6 24.2 15.4 27 21.7

GGT7 99.55 121.2 93.5 214

GGT8P 27.8 63.9 70.5 30.1

GGTA1 6.9 12.678 5.844 11.539

GGTLC1 92.1 846 478 168

GGTLC2 124 855 402.5 165

GH1 33.8 30 23.2 43.9

GH2 3.33 5.43 4.96 16.4

GHDC 549 365 603 819

GHITM 7865.55 14023.55 12412.45 12881.35

GHR 586.5 125.7 65.6 73.55

GHRH 10.095 12 4.685 26.225

GHRHR 21.6 17.78 12.6 12.355

GHRL 20.44 69.34 28.43 30.85

GHRLOS 355 218 345 446

GHSR 123 132 126 119

GIF 2.69 4.36 62.2 3.29

GIGYF1 65.7 133 70.8 124

GIGYF2 3550 3350 2340 3620

GIMAP1 2.57 4.16 3.78 3.18

GIMAP2 2.68 4.34 3.94 3.3

GIMAP4 3.58 5.73 5.35 4.33

GIMAP5 4.16 6.28 5.94 4.95

GIMAP6 8.72 9.02 6.91 3.5

GIMAP7 3.74 5.94 5.53 4.52

GIMAP8 12.6 5.95 5.6 4.56

GIN1 157 240.5 152.5 257.5

GINS1 4330 17600 9230 9860

GINS2 2252 3639 7628 10322

GINS3 4280 3470 6040 11300

GINS4 3390 9430 6640 8470

GIP 13.5 18.7 21.9 32.8

GIPC1 22800 11000 41500 23600

GIPC2 60.7 56.9 4.33 14.4

GIPC3 18.28 35.605 15.85 39.35

GIPR 175 229 200 252

GIT1 77.05 82.85 182 83.2

GIT2 636 1495.35 776 1226.15

GIYD1 2250 606 2320 1820

GJA1 105 9160 733 3650

GJA10 3.6 5.73 5.42 4.45

GJA3 7.83 12.6 30 384

GJA4 20.3 20.9 27 23.1

GJA5 3.3 5.37 4.93 4.01

GJA8 12.5 7.67 6.02 3.29

GJA9 11.2 5.66 5.24 10.3

GJB1 566 318 36.1 3.48

GJB2 312.4 88.51 5.118 113.42

GJB3 3.54 5.66 39.2 4.4

GJB4 43.755 42.5 38.135 39.695

GJB5 3.35 5.45 4.97 3.97

GJB6 3.67 5.81 5.57 4.64

GJB7 3.7 347 4.925 150.5

GJC1 5650 4690 6500 7690

GJC2 565 115 418 326

GJC3 7.81 11.6 5.39 7.34

GJD2 2.95 4.7 4.34 3.56

GJD3 15.14 24.3 15.1 22.4

GJD4 3290 3500 3910 3760

GK 281.5 278.5 68.9 166.5

GK2 3.07 4.93 4.51 3.73

GK5 53.8 68.4 175 102

GKAP1 188 301 206 639

GKN1 2.91 4.64 24 4.49

GKN2 12 14.4 14.7 11.1

GLA 10100 47200 6620 38000

GLB1 3960 3380 6470 3320

GLB1L 23.5 45.6 61.7 56.3

GLB1L2 7.83 5.24 108 133

GLB1L3 2.83 23.3 4.15 41.7

GLCCI1 3480 3050 699 1930

GLCE 144 374 291 339

GLDC 5352 4838 1405 1786

GLDN 15.4 18.3 5.58 32.2

GLE1 92.7 138 162 238

GLG1 8300 10100 13600 7910

GLI1 8.43 51.3 26.2 170

GLI2 11.5 11.335 27.45 111.85

GLI3 2.76 45.2 248 2090

GLI4 441 259 726 804

GLIPR1 7.57 209 6.43 3.4

GLIPR1L1 3.96 6.22 5.82 4.77

GLIPR1L2 3.055 6.915 118.75 23.05

GLIPR2 495 383 66.25 970

GLIS1 48.4 50.3 52.5 300

GLIS2 8.19 337 154 119

GLIS3 29.1 28.9 26.8 4.29

GLMN 378 699 686 1540

GLO1 1610 2690 1870 5580

GLOD4 3570 2460 3580 3770

GLOD5 10.1 21.3 11.1 8.43

GLP1R 3.75 6.28 5.67 4.72

GLP2R 17.05 25.87 18.3133333333333 13.6466666666667

GLRA1 4.65 4.76 4.33 3.53

GLRA2 3.84 6.01 5.8 4.91

GLRA3 3.34 5.37 4.96 4.06

GLRA4 15 5.99 5.56 5.48

GLRB 3.52 5.66 5.25 4.15

GLRX 1320 10400 882 3410

GLRX2 5140 4130 6810 5180

GLRX3 7250 13300 10400 12600

GLRX5 12500 13600 16600 21700

GLS 1740 822 4440 1239.5

GLS2 25.1 11.5 31.2 142

GLT1D1 554 39.1 6.8 4.54

GLT25D1 1004.5 558.5 1263.5 1068.5

GLT25D2 62.09 5.435 45.5 100.19

GLT6D1 3.82 6.03 5.78 4.89

GLT8D1 927 1064 2240 1860

GLT8D2 3.13 5.47 276.185 263.28

GLT8D3 300 569 344 474

GLT8D4 7.35 177 7.55 553

GLTP 61.5 89.9 157 278

GLTPD1 4566.5 2255.75 3423.25 1399

GLTPD2 967 518 56.2 68.5

GLTSCR1 187.5 171.5 137 198

GLTSCR2 21870 13665 15215 20655

GLUD1 18800 12100 8390 5070

GLUD2 6110 3550 2850 1250

GLUL 8840 3400 694 4380

GLYAT 8.67 5.02 54.13 20.48

GLYATL1 1360 201 4.36 7.39

GLYATL2 5.4 4.77 4.41 3.63

GLYCAM1 3.73 8.11 11.9 4.8

GLYCTK 708.5 640 232 93

GLYR1 451 565 448 644

GM2A 1270 1401 2409.5 2907

GMCL1 645 825 1020 720

GMCL1L 3.4 6.62 5.09 4.1

GMDS 1670 467 974 436

GMEB1 634 874 678 1200

GMEB2 392 469 325 369

GMFB 1569.5 1351.5 1060 1500.5

GMFG 24.8 58.6 150 208

GMIP 476 714 445 432

GML 40.1 76.1 63.7 73.1

GMNN 23700 14100 3950 21000

GMPPA 532 664 385 470

GMPPB 390 561 610 813

GMPR 7.42 6.75 10.9 129

GMPR2 7060 5703.5 4314.5 8660

GMPS 10900 6960 19500 15100

GNA11 851 825 970 987

GNA12 2451 3332.5 1461.5 1491

GNA13 1701 2517.5 1498.5 1893.5

GNA14 3.51 8.72 5.23 8.97

GNA15 18.7 19.2 30.9 18.5

GNAI1 1019.1 899 970.5 764.8

GNAI2 17600 21700 28300 14100

GNAI3 513.5 548.5 425 819

GNAL 94.4 114 101 120

GNAO1 24.9566666666667 18.6266666666667 19.5433333333333 28.6

GNAQ 439 671 981 483

GNAS 24103.2025 27127.725 25652.52 29627.0625

GNASAS 405 92.7 5.45 1400

GNAT1 3.42 5.53 5.08 4.16

GNAT2 4.84 6.55 19.8 31.6

GNAT3 3.67 5.84 5.43 4.43

GNAZ 61.3 1680 332 2290

GNB1 9440 7070 9060 4250

GNB1L 2110 4380 4000 5210

GNB2 1620 2920 1890 2270

GNB2L1 50800 46800 45500 81700

GNB3 126 110 169 115

GNB4 814 1710 2440 4650

GNB5 457 563 727 317

GNE 3280 7000 3290 3650

GNG10 2980.5 8705 6365 9260

GNG11 3.569 603.3 30170 973.8

GNG12 1190 1350 2830 1380

GNG13 76.9 88.7 58.2 61.9

GNG2 3.64 4.71 12.6 84.7

GNG3 3.4 5.49 5.03 4.13

GNG4 507.65 735.25 7.715 913.4

GNG5 13900 17600 11600 12800

GNG7 126 144 369 718

GNG8 124 139 132 90.1

GNGT1 3.087 42.94 19.63 203.5

GNGT2 5.31 4.25 3.85 3.23

GNL1 771 564.5 426.5 687.5

GNL2 4520 6520 5050 8240

GNL3 11100 7760 8440 17800

GNL3L 4471 11890 3904 8450

GNL3LP 511 1380 582 1010

GNLY 4.08 5.84 8.695 4.645

GNMT 12 12.9 25.8 30

GNPAT 7420 3850 3690 4850

GNPDA1 595 488 887 629

GNPDA2 409 684 456 205

GNPNAT1 2140 2460 2160 4370

GNPTAB 462.5 898.5 1250.5 1182.5

GNPTG 4010 1600 4010 2580

GNRH1 102 83.3 202 98.9

GNRH2 41.65 69.9 63.2 81.15

GNRHR 2.98 6.74 5.04 3.6

GNRHR2 40.8 43.6 37.9 117

GNS 3460 5860 5720 5460

GOLGA1 188 345 169 293

GOLGA2 520 908 545 595

GOLGA2L1 646 628.5 738 374.5

GOLGA2LY1 257 210 125 504

GOLGA3 1670 3380 3400 5080

GOLGA4 1550 3030 2180 1680

GOLGA5 619 927 845 931

GOLGA6A 28.5933333333333 32.2133333333333 31.34 33.5266666666667

GOLGA6L1 10.9 8.92 18 35.3

GOLGA6L10 369 346.5 252.5 1049

GOLGA6L2 30.6 14.1 27.5 31.2

GOLGA6L5 5.315 7.325 11.935 11.54

GOLGA6L6 7.16 14.33 16.9366666666667 233.77

GOLGA6L9 3147.66666666667 3247.66666666667 2194.33333333333 7656.66666666667

GOLGA7 2546 5246.66666666667 2320.66666666667 3312.66666666667

GOLGA7B 6.04 6.24 5.84 13.3

GOLGA8A 199.5 325.5 299.5 478

GOLGA8E 311.333333333333 320.666666666667 224.666666666667 482.333333333333

GOLGA8F 72.4 101 71.9 104

GOLGA9P 181 237 197 339

GOLGB1 477.7 725.8 477.5 860.85

GOLIM4 2790 2580 2330 2320

GOLM1 24.165 2160 2440 2435

GOLPH3 4830 3240 2860 2130

GOLPH3L 2200 1390 1600 2890

GOLSYN 144 61.8 255 481

GOLT1A 2560 298 57.4 13.4

GOLT1B 861 1560 1340 1100

GON4L 2405 3033.66666666667 1678 2421.66666666667

GOPC 1900 2640 1490 3460

GORAB 266.3 131.75 107.45 147.6

GORASP1 89.5 109 181 125

GORASP2 8560 7560 5430 8510

GOSR1 905 1440 1240 995

GOSR2 1760 2186.5 1034 1433

GOT1 15100 7660 5270 4950

GOT1L1 3.475 4.64 4.205 3.465

GOT2 34800 37700 61000 63100

GP1BA 16 24.9 29.3 25.3

GP2 3.22 5.92 4.71 3.85

GP5 2.76 4.47 4.09 3.4

GP6 5.54 5.46 31.6 22.6

GP9 67.5 106 77.6 97.7

GPA33 2.71 8.02 34.4 90.5

GPAA1 18190 9821.5 15285.5 14530

GPAM 342 2720 1060 2190

GPAT2 103.34 135.303333333333 92.8633333333333 114.966666666667

GPATCH1 414 550 410 423

GPATCH2 395 319.5 313.5 414

GPATCH3 799 870 613 553.65

GPATCH4 7005 8050 5595 15950

GPATCH8 741 698 705 1300

GPBAR1 50.9 69.7 89.1 64.5

GPBP1 4320 2750 3840 7640

GPBP1L1 2380 2050 2080 2440

GPC1 1780 195 3640 1100

GPC2 317 356 144 448

GPC3 17850 14220 397 968.5

GPC4 216 45.1 2160 2190

GPC5 6.70181818181818 5.94818181818182 5.94181818181818 11.7263636363636

GPC6 3805 1187.5 102.95 1591

GPCRLTM7 15.2 4.93 4.51 3.68

GPD1 25.6 14.2 38.3 16.2

GPD1L 1410 933 2830 2050

GPD2 621.5 1114 980 502

GPER 4661.75 1461.25 36.175 215.2

GPHA2 14.2 17.3 40.2 34.2

GPHB5 95.9 99 79.7 80.4

GPHN 1640 1290 1850 2740

GPI 28300 27900 45500 29600

GPIHBP1 45.1 51.3 42.2 52.5

GPKOW 2719 9558 2859 4554

GPLD1 13.9 19.475 5.76 33.3

GPM6A 17.5 5.379 19.34 166.4

GPM6B 4.03 10.505 5.03 461.5

GPN1 4240 3825 4935 6440

GPN2 1910 2440 3050 2010

GPN3 3370 6950 7220 6250

GPNMB 221 79.4 39.8 84.8

GPR1 3.59 5.66 5.35 4.24

GPR101 3.61 5.57 5.43 4.61

GPR107 2140 3820 2570 2750

GPR108 1050 477 333 562

GPR109A 29.9 95.7 24.6 32.8

GPR109B 542 3240 4.78 5.43

GPR110 3.295 8.41 1011.1 4.005

GPR111 3.08 4.93 4.5 3.7

GPR112 6.7 30.2 29.8 5.91

GPR113 28.6 37.9 30.1 29.5

GPR114 70 25.5 21.8 25.8

GPR115 2.88 4.68 6.37 3.52

GPR116 3.26 5.235 4.8 3.885

GPR119 363 360 250 293

GPR12 3.69 5.8 5.59 4.72

GPR120 82.4 96.7 114 67.3

GPR123 7.87 8.96 4.62 4.49

GPR124 28.9 26.3 255 321

GPR125 5669.5 6079 1411.5 1580

GPR126 2870 5860 851 202

GPR128 16.7 15.4 5.47 4.36

GPR132 43.8 10.1 13.3 34

GPR133 22.62 12.1666666666667 176.3 14.6166666666667

GPR135 131 113 455 546

GPR137 245 186 323 365

GPR137B 123.8 119.9 228 271.5

GPR137C 63.4 78 146 174

GPR139 3.61 5.1 4.64 3.74

GPR141 2.58 4.18 3.78 3.17

GPR142 36 41.9 48.1 40.1

GPR143 1890 26.2 9.97 5.82

GPR144 59.1 46.8 50.1 51.8

GPR146 57.3 47.4 46.1 55.6

GPR148 3.27 5.34 4.86 3.93

GPR149 9.42 7.04 14.8 11

GPR15 33.7 37.9 34.5 43.5

GPR150 11900 14600 12500 13100

GPR151 19.9 29.1 21.2 19.5

GPR152 974 1010 878 777

GPR153 3.77 5.81 9.72 8.79

GPR155 78722.9333333333 82013.3666666667 87019.9666666667 84188

GPR156 12.4 14 16.3 20.9

GPR157 29.3 21.4 20 22.4

GPR158 3.26 547 267 3.97

GPR160 1320 513 2070 1240

GPR161 221 238.5 127.8 274

GPR162 109 126 574 465

GPR17 19.4 18.3 7.7 14.7

GPR171 12.4 29.6 44.3 69.9

GPR172A 15000 10200 29500 9990

GPR172B 28.9 49.6 41 33.4

GPR173 32.3 24.5 34 33.9

GPR174 3.194 6.883 4.731 3.912

GPR176 44.7 35.3 38.6 105

GPR177 700 217 952 2195.5

GPR179 37.4 52.4 10.8 23.1

GPR18 5.05 5.73 5.32 4.33

GPR180 6.6 521 1020 1660

GPR182 14.3 32.1 6.94 12.5

GPR183 18.5 17 20.8 23

GPR19 13.3 217 57.7 76.1

GPR20 27.5 23.7 283 114

GPR21 5.82 6.62 8.65 5.36

GPR22 12.7 52.7 4.15 20.3

GPR25 207 178 169 221

GPR26 3.475 6.265 4.65 63.84

GPR27 2.89 4.66 6.51 38.7

GPR3 76.7 46.5 141 35.1

GPR31 14.6 8.62 7.25 7.25

GPR32 20.5 14.7 25 27.3

GPR34 5.747 22.086 9.65 12.55

GPR35 1290 170 462 78

GPR37 18.5 123 4.33 3.56

GPR37L1 27.8 23.7 12.1 10.7

GPR39 46.7 103 378 24.2

GPR4 3.24 5.29 16.8 5.16

GPR44 92.5 50.6 4.5 3.79

GPR45 19.3 49.6 50.8 28.2

GPR50 24 23.9 21.8 20.3

GPR52 14.2 51.1 6.98 32

GPR55 5.35 7.3 8.82 8.39

GPR56 2990 460 14600 10.3

GPR6 2.57 4.17 3.78 3.17

GPR61 20 20.5 19 17.9

GPR62 28.7 36.7 26 57.2

GPR63 26.2 41.2 68.8 128

GPR64 3.085 4.985 4.605 52.71

GPR65 3.21 5.18 4.78 3.91

GPR68 18.5 23.6 4.85 31.7

GPR75 26.5 37.6 18.6 154

GPR77 3.18 5.16 4.7 3.88

GPR78 13 10.5 24.8 15.8

GPR81 8.975 17.94 6.605 6.745

GPR82 3.48 5.64 5.18 4.25

GPR83 11.7 5.83 5.47 4.47

GPR84 5.42 4.49 15 11.5

GPR85 3.47 5.63 5.17 4.09

GPR87 6.125 8.636 6.546 9.199

GPR88 223.275 226.6 225.145 194.745

GPR89B 1420 907 1100 1290

GPR97 226.55 278.5 254.45 210.75

GPR98 129 850 54.8 612

GPRASP1 91.5 181 34.1 133

GPRASP2 92.9 1440 46.5 2590

GPRC5A 21.1 76.7 554 33

GPRC5B 1140 1620 7180 857

GPRC5C 15000 23300 93900 1760

GPRC5D 20.779 26.173 25.28 25.48

GPRC6A 24.5 14.9 16 16.2

GPRIN1 268.18 177.235 415.765 453.7

GPRIN2 3.77 60.9 5.69 4.85

GPRIN3 3.42 15.2 5.08 26.5

GPS1 3730 3220 4690 5280

GPS2 10000 9900 8370 9850

GPSM1 1305.225 843.025 1063.35 2034.775

GPSM2 544 601 1300 971

GPSM3 180 289 168 211

GPT 51.7 4.92 4.47 3.62

GPT2 2870 1260 654 1960

GPX1 16845 46200 56600 54750

GPX2 2740 5370 159 42.7

GPX3 640 1795 9.44 477.5

GPX4 31000 14400 20400 11900

GPX5 58.6 46.6 42.5 34.1

GPX6 7.35 8.51 4.145 6.86

GPX7 369 338 7.17 8.26

GPX8 24.5218181818182 114.127272727273 2402.72727272727 2846.36363636364

GRAMD1A 99.9 179 182 156

GRAMD1B 5.29 6.22 306 51.1

GRAMD1C 47.9 92.8 251 230

GRAMD2 18 20.2 109 3.35

GRAMD3 171 193 1490 47.4

GRAMD4 1910 950 824 1360

GRAP 7.57 6.435 7.68 5.57

GRAP2 7.5 12.9 7.77 12.6

GRAPL 3.58 10.1 9.22 8.34

GRASP 61.3 87.7 358 308

GRASPOS 102 114 111 115

GRB10 5445.45454545455 1668.18181818182 119.881818181818 2275.45454545455

GRB14 7960 2770 6570 2230

GRB2 1580 1570 3170 1550

GRB7 258 219 596 78.9

GREB1 430.55 372.55 115.15 155.8

GREB1L 16.9363636363636 219.727272727273 13.88 218.545454545455

GREM1 5.73 58.15 4.775 122.63

GREM2 2.79 4.54 4.46 27.3

GRHL1 370.15 831.85 210.65 552.05

GRHL2 9.92 12.48 13.475 4.825

GRHL3 8.97 12.3 17.3 31.2

GRHPR 2350 9140 1340 5010

GRIA1 3.44 8.65 5.14 4.06

GRIA2 3.03 22.575 4.495 3.64

GRIA3 4.62 29.7 5.63 9.97

GRIA4 84.92 79.015 69.4 59.445

GRID1 3.61 5.38666666666667 205.303333333333 77.16

GRID2 11.5 8.3 12.8 16.1

GRID2IP 131.51 118.355 92.22 64.31

GRIK1 3.591 5.869 5.09 4.151

GRIK2 7.3125 22.925 41.975 9.3675

GRIK3 14.89 15.36 18.3933333333333 19.5133333333333

GRIK4 3.80636363636364 5.10636363636364 5.38181818181818 5.92181818181818

GRIK5 52.8 62.3 40.4 66.1

GRIN1 6.945 20.15 7.97 8.385

GRIN2A 34.0675 45.9625 24.6625 32.4225

GRIN2B 3.73 5.93 5.51 4.41

GRIN2C 21.9 18.55 23.45 48.85

GRIN2D 18.3 153 603 473

GRIN3A 67.9 58.1 41.9 58.1

GRIN3B 18.4 5.41 15.9 7.42

GRINA 1600 1370 1190 1260

GRINL1A 6.17 5.9 5.74 7.27

GRIP1 5.35545454545455 6.77 7.58545454545455 41.0090909090909

GRIP2 7.31666666666667 7.82 9.70333333333333 6.33

GRIPAP1 345.65 1921.35 295.35 839.7

GRK1 10.165 63.1 14.325 10.95

GRK4 15.2 5.11 12 66.7

GRK5 691 5060 462 1270

GRK6 457 1141.5 1101 1690.5

GRK7 3.545 5.615 5.32 4.38

GRLF1 771.5 490.5 811.5 311

GRM1 10.22 17.72 16.32 18.465

GRM2 4.26 6.4 8.1 5.16

GRM3 3.06 5 4.54 5.07

GRM4 11.4 13.3 35.5 25

GRM5 9.26 17.135 8.455 30.82

GRM6 4.85 4.57 4.18 3.48

GRM7 5.26 5.675 5.24 5.13

GRM8 10.235 5.305 5.155 7.99

GRN 46000 38700 61300 43000

GRP 9.717 12.159 13.174 30.82

GRPEL1 6990 4170 3090 6900

GRPEL2 1930 2690 2790 5220

GRPR 13.6 36.3 5.33 9.78

GRRP1 36.1 37.4 39.8 39.4

GRTP1 758 189 275 349

GRWD1 6520 7300 7420 11500

GRXCR1 10.1 31.7 11.2 12.5

GRXCR2 3.18 5.13 5.47 6.59

GS85 10.1 8.75 28.6 5.82

GSC 2.54 4.11 5.21 1030

GSC2 10.4 9.93 9.65 11.3

GSDMA 5.63 7.93 5.78 3.83

GSDMB 1020 563 864 445

GSDMC 2.71 10.9 5.48 3.33

GSDMD 335 259 564 8.27

GSG1 28.2633333333333 27.69 37.9666666666667 33.4333333333333

GSG1L 3.83 6.14 5.61 4.48

GSG2 322 407 494 478

GSK3A 10100 13000 11300 11400

GSK3B 1593 1684 1893 1296

GSN 365 1023 1213 456.5

GSPT1 2480 1560 2580 2020

GSPT2 948 144 112 966

GSR 2002.5 3560 1990 1164.5

GSS 9270 13700 7410 7390

GSTA2 23.9 392 12.6 11.4

GSTA3 11.8 12.9 8.63 18

GSTA4 1385.45454545455 2450 50.9272727272727 2508.18181818182

GSTA5 39.7 1380 5.09 4.16

GSTCD 159.5 268.5 143.9 203

GSTK1 3383.5 5313.5 8005 7585

GSTM1 941.9 1109 58.73 948.4

GSTM2 65.5 203.5 10.075 468

GSTM3 20040 14290 21.701 17920

GSTM4 273 1360 75.3 87.5

GSTM5 135 128 119 134

GSTO1 27000 26800 9130 12100

GSTO2 98.3 109 13.8 518

GSTP1 28.33 192300 126200 85360

GSTT1 2290 5.88 13.7 4.86

GSTT2 333 373 26.3 7.86

GSTT2B 151 176 15.9 12.3

GSTTP1 3.145 5.07 4.65 3.77

GSTTP2 14.8 14.3 21.3 15.9

GSTZ1 280 420 569 1210

GSX1 46.6 49.3 73.4 62.1

GSX2 3.04 4.94 5.76 3.71

GTDC1 453.8 342.4 385.145 254.78

GTF2A1 572 649 735 971

GTF2A1L 3.41 5.49 5.08 4.12

GTF2A2 12650 13100 13000 11410

GTF2B 699 925 841 1530

GTF2E1 2460 3080 4980 1970

GTF2E2 1540 1600 2940 1670

GTF2F1 4280 1810 3040 4670

GTF2F2 1930 4600 3720 6930

GTF2H1 2240 2760 3090 2890

GTF2H2D 6810 9490 9500 12500

GTF2H3 720 704 1750 1420

GTF2H4 576 337 644 489

GTF2H5 6970 10400 6120 12600

GTF2I 33900 38100 21400 31400

GTF2IRD1 1494 1800 2035 1552

GTF2IRD2 36.7333333333333 11.0933333333333 20.6133333333333 6.93666666666667

GTF3A 23700 15500 11600 24400

GTF3C1 235 243 172 423

GTF3C2 5380 5480 3430 6880

GTF3C3 1720 1690 1750 3080

GTF3C4 990.5 1325 911 1592

GTF3C5 3720 4560 4000 7270

GTF3C6 36900 21200 34600 39700

GTPBP1 468 701 840 1420

GTPBP10 137 183 106 253

GTPBP2 1553.5 765.2 826.5 1049.65

GTPBP3 954 743 922 2220

GTPBP4 10800 7510 17100 18100

GTPBP5 659 1130 652 1060

GTPBP6 1937.93333333333 1487.36666666667 4498 4462.6

GTPBP8 9910 7100 6840 14000

GTSE1 1960 1590 2210 2700

GTSF1 41 236 4.26 5370

GTSF1L 3.52 5.64 5.2 4.19

GUCA1A 7.43 6.28 11.6 9.35

GUCA1B 176.45 163.15 86.35 27.75

GUCA1C 2.95 4.76 55.1 3.6

GUCA2A 11.3 29.4 16.7 19.3

GUCA2B 7.48 74.8 4.68 231

GUCY1A2 39.3 80 5.57 20.6

GUCY1A3 49.05 594.825 13.71 392.45

GUCY1B2 20.8 194 8.05 445

GUCY1B3 11.7263636363636 26.4909090909091 27.5290909090909 204.781818181818

GUCY2C 13 25.4 46.4 3.58

GUCY2D 3.34 5.35 4.94 4.04

GUCY2E 3.095 4.895 5.825 3.68

GUCY2F 3.95 6.25 5.84 4.78

GUCY2G 21.8 25.5 55.1 16.5

GUF1 2210 1610 561 942

GUK1 5895 3775 7145 10205

GULP1 224.133333333333 2337.33333333333 1211.33333333333 568.7

GUSB 14000 16220 6390 9273

GUSBL2 2750 3820 1970 3300

GVIN1 9.88 5.61 5.11 4.92

GYG1 1728 2303.5 3175 1971

GYG2 187.55 658.4 14.15 254.95

GYLTL1B 1704.5 246.2 266.85 242.95

GYPA 196.37 1472.215 652.01 1146.68

GYPB 3.04 4.925 4.49 3.685

GYPC 275 68.3 75.4 146

GYPE 10.8 4.87 5.49 5.9

GYS1 1340 1490 4050 2080

GYS2 4.27 6.83 5.46 3.16

GZF1 349.666666666667 599.333333333333 289.666666666667 362

GZMA 12.9 5.27 7.69 9.63

GZMB 51.06 55.8 47.77 60.93

GZMH 21.4 52.4 19 12.3

GZMK 8.9 5.44 4.58 3.76

GZMM 207 258 297 311

H19 17200 113 382 7.9

H1F0 2880 4850 17600 608

H1FNT 128 108 192 111

H1FOO 14.5 5.62 11.8 21.6

H1FX 302 861 1730 1080

H2AFB2 20.15 24.35 29.2 31.9

H2AFB3 16.8 17.2 5.33 34.1

H2AFJ 3307.45 4695.9 72.175 1082.8

H2AFV 32414 22801 40112.5 19110

H2AFX 8280 18500 31000 15300

H2AFY 16800 16700 25700 24500

H2AFY2 487 155 12.2 1040

H2AFZ 34100 52900 39100 58400

H2BFM 2.96 17 43.7 510

H2BFWT 3.43 5.51 5.12 40.1

H2BFXP 21.93 22.72 38.95 128.15

H3F3A 81400 58166.6666666667 65066.6666666667 130266.666666667

H3F3B 16148 21408.5 29627 19930

H6PD 30.1 6.49 5.02 7.98

HAAO 734.5 803 28.75 70.75

HABP2 387.3 123.5 7.905 5.964

HABP4 1870 5080 4580 3150

HACE1 267.5 203 260.5 380.5

HACL1 6608 6160 9259 6101

HADH 7630 6910 11100 15000

HADHA 1880 1990 2410 2840

HADHB 11200 10800 11500 15100

HAGH 11000 6940 12500 11200

HAGHL 347 342 256 300

HAL 46.3 45.6 4.68 3.82

HAMP 1250 2980 127 160

HAND1 6.45 9.6 16.4 845

HAND2 2.8 4.56 6.88 38.7

HAO1 80.8 35.7 19.2 12.8

HAO2 6.41 5.15 6.84 9.72

HAP1 66.9 64.5 85.3 76.4

HAPLN1 3.54 5.72 8.9 4.75

HAPLN2 4830 6380 5830 7260

HAPLN3 76.7 132 844 3580

HAPLN4 11.8 26.8 9.45 9.31

HAR1A 249 245 178 228

HAR1B 3 4.81 5.81 3.65

HARBI1 483 458 406 580

HARS 4260 4900 6650 7730

HARS2 2730 2810 2950 2780

HAS1 13.2533333333333 13.94 13.3466666666667 13.5833333333333

HAS2 69.45 103.06 5.061 9.328

HAS2AS 5.81 18.3 5.38 8.98

HAS3 164.95 85.5 567 104.25

HAT1 10300 13300 13200 14000

HAUS1 2950 3890 6650 5570

HAUS2 1077.5 1145 1016 717

HAUS3 80.6 94.6 27.5 95.1

HAUS4 336 844 738 642

HAUS5 780.4 1218 1367.5 2287.5

HAUS6 1920 1610 1560 3940

HAUS7 516 843.5 1297 2602

HAUS8 2351.5 3093.5 4916 2643.5

HAVCR1 22.3 877 1480 7.51

HAVCR2 3.7 5.93 5.44 4.35

HAX1 13600 13900 7730 18000

HBA2 25.4666666666667 34 252.633333333333 81.5333333333333

HBB 6.263 7.671 14.351 5.039

HBBP1 17.6 10.4 16.8 9.38

HBD 35.1 46.8 49.2 38.4

HBE1 2.6 17.9 6.48 11.8

HBEGF 90.4 176 69.3 132

HBG1 37.9190909090909 27.2181818181818 25.6554545454545 24.1718181818182

HBM 39.6 30.7 27.7 40.1

HBP1 192 424 365 177

HBQ1 269 254 144 287

HBS1L 1193.025 1546.1 758.415 1940.65

HBXIP 7943 16770 15460 19000

HBZ 139 152.665 119.76 139.65

HCCA2 581 610 589 918

HCCS 2390 4850 1730 4570

HCFC1 100 109 108 244

HCFC1R1 1930 5390 7170 4360

HCFC2 217.9 314.066666666667 173.3 393.666666666667

HCG11 2870 3740 3880 4370

HCG18 1070 793 187 1070

HCG22 3.37 5.37 5.01 4.27

HCG26 3.38 5.41 4.98 4.09

HCG27 43.8 13 40.8 4.37

HCG2P7 601 659 721 697

HCG4 9.64 18.5 12.4 27.6

HCG4P6 11.73 8.87 10.015 15.5

HCG8 12.7 22.1 22.1 12.4

HCG9 6.26 5.19 4.71 10.6

HCK 15.9 12.8 19 13.9

HCLS1 57.1 74.5 115 97.7

HCN1 18.6 4.85 4.45 3.69

HCN2 20356.2 22102.46 17004.065 18953.82

HCN3 265 213 299 368

HCN4 3.475 8.185 13.255 6.71

HCP5 25.1 28.6 27.8 192

HCP5P10 20 12.2 17.9 23.2

HCRP1 26.5 25.4 37.5 32

HCRT 20.1 27 18.4 19

HCRTR1 176.995 115.92 240.1 91.38

HCRTR2 3.69 5.84 5.46 4.38

HCST 122 116 243 135

HDAC1 8920 14690 20760 22640

HDAC10 47.6 78.7 73 75.7

HDAC11 147.8 80.1 106.733333333333 63.6

HDAC2 7815.18181818182 6436.27272727273 8383.45454545455 12513.6363636364

HDAC3 3900 6980 9210 10900

HDAC4 333.95 449.15 1659 298.5

HDAC5 289 136 113 380

HDAC6 279.75 1755.5 376.25 702

HDAC7 325.25 500.3 589.8 176

HDAC8 268.25 297.75 315.075 772.5

HDAC9 77.45 5.115 16.71 8.03

HDC 38.3 75.65 27.42 52.51

HDDC2 644.272727272727 615.272727272727 576.818181818182 1573.63636363636

HDDC3 2010 3090 1700 5170

HDGF 12230 12480 8575 7930

HDGF2 510 279 557 927

HDGFL1 201 185 212 336

HDGFRP3 1020 178 3270 2350

HDHD1A 911 2260 455 843

HDHD2 3900 6300 5560 3700

HDHD3 153 66.7 135 140

HDLBP 6080 4480 5480 4490

HDX 79.3 6.88 9.76 108

HEATR1 6160 3299 4291 11470

HEATR2 3475 2284.5 1538 2750

HEATR3 845 715 618 1190

HEATR4 12.1 13.3 14.6 10.2

HEATR5A 673 4805 414 667

HEATR5B 259 243 195 409

HEATR6 786 580 611 1430

HEATR7A 81.5666666666667 50.8333333333333 108.266666666667 50.7

HEATR7B1 5.075 6.04 40.25 6.395

HEATR7B2 3.41 5.57 12 4.11

HEBP1 2945 2698 15860 5062

HEBP2 4470 12900 5890 17200

HECA 1040 1040 1010 4570

HECTD1 12700 41200 4630 10100

HECTD2 133.833333333333 214.266666666667 49.9 237

HECTD3 339 323 417 285

HECW1 3.71 5.89 5.44 4.39

HECW2 42.4 131 3.8 198

HEG1 2650 8980 704 1190

HEJ1 19000 12800 14700 17400

HELB 53.1 119 109 196

HELLS 819.5 1745 2130 2230

HELQ 151 144 272 359

HELT 6.97 5.3 8.61 17.6

HELZ 1610 1390 1000 2620

HEMGN 4.94 5.71 5.25 4.3

HEMK1 3.61 8.65 8.31 27.7

HEPACAM 12.395 9.93 12.55 9.355

HEPACAM2 7.1 35.5 9.48 3.85

HEPH 75.5 120 71.9 88.6

HEPHL1 3.41 5.54 5.05 4.03

HEPN1 12.195 9.13 31.665 15.14

HERC1 1470 1780 912 1250

HERC2 305.666666666667 372 434.333333333333 397.666666666667

HERC2P2 1220 1100 1750 1860

HERC2P4 78.7 95.6 144 123

HERC2P7 135 179 220 66

HERC3 13.8 13.2 4.36 29.9

HERC4 434.333333333333 581.333333333333 454.666666666667 537.666666666667

HERC5 58.08 140.2 56.86 624.9

HERC6 2.83 4.55 9.52 20.75

HERPUD1 16400 9960 16700 14400

HERPUD2 341 227 367 213

HERV-FRD 2.63 8.76 3.88 4.8

HES1 202 254 454 152

HES2 3.28 4.22 732 4.82

HES3 5.09 10.2 6.01 7.43

HES4 19400 2920 26200 36800

HES5 18 12.5 191 225

HES6 6390.75 2304.25 2508.5 2767.75

HES7 16.8 4.77 14.2 87.3

HESRG 2.77 4.48 4.07 3.39

HESX1 10.481 10.224 67.66 50.98

HEXA 1510 2160 911 1560

HEXB 5590 5210 7630 3570

HEXDC 4080 4710 3050 5420

HEXIM1 262 125 1650 442

HEXIM2 4750 2930 10800 3870

HEY1 55.1 1060 83.6 25700

HEY2 9.52 4.28 3.88 268

HEYL 13.1 70.1 10.5 702

HFE 37.91 28.59 4.92 4.115

HFE2 389 253 5.35 4.37

HFM1 2.78 68.6 4.08 25.3

HGC6.3 3.99 6.21 5.71 4.67

HGD 10000 27100 1750 11.3

HGF 3.915 199.5 4.895 8.66

HGFAC 262 209 181 136

HGS 19000 14600 24100 16900

HGSNAT 1854.71818181818 1431.39090909091 991.972727272727 867.9

HHAT 488 39.6 44.4 163

HHATL 5.62 4.45 4.03 6.75

HHEX 2100 2750 375 223

HHIP 3.29 5.3 5.31 75.2

HHIPL1 21.5 42.6 12.565 14.055

HHIPL2 3.26 5.23 4.82 4.64

HHLA1 3.78 5.94 5.7 4.77

HHLA2 17.295 18.725 24.585 32.105

HHLA3 90.9 123 265 214

HIAT1 2020 2990 2000 3240

HIATL1 798 1068.5 1681 1150.5

HIBADH 2630 2280 2690 2020

HIBCH 1876 1962 2937 2564.5

HIC1 17.6 11.3 11 20.6

HIC2 4435 9725 866.5 5450

HIF1A 1762.09090909091 3069.18181818182 1810.45454545455 1201

HIF1AN 84.5 164 130 144

HIF3A 1989.17 2122.15666666667 963.97 1874.41333333333

HIGD1A 7589 14508 8247.55 8140.5

HIGD1B 9.5 4.45 9.45 8.09

HIGD1C 3.61 5.76 5.35 4.37

HIGD2A 3940 3750 6260 5800

HIGD2B 6.9 8.45 13.9 6.33

HILS1 7.54 4.88 18.3 17.3

HINFP 523 618 1260 910

HINT1 145000 186000 172000 126000

HINT2 1880 3340 3880 3290

HINT3 2930 2500 1300 3220

HIP1 166 295 370 331

HIP1R 896.1 878.236666666667 1552 1986.46666666667

HIPK1 1223.5 2361 1390 2115.5

HIPK2 315 317 1878 322.5

HIPK3 201 230 248 130

HIPK4 6.09 8.49 18.5 10.3

HIRA 6870 12900 5210 9670

HIRIP3 2302 931.95 3173 5224

HISPPD1 1600 2317 717.7 1261

HISPPD2A 66.1333333333333 49.8266666666667 243.566666666667 123.4

HIST1H1A 4340 21200 15.2 11.4

HIST1H1B 1360 3630 5 6.81

HIST1H1C 6610 33100 11500 18500

HIST1H1D 587 4680 1380 6440

HIST1H1E 4240 11200 5180 11000

HIST1H1T 4.01 6.21 5.77 4.72

HIST1H2AA 42 119 60.5 75

HIST1H2AB 921 2630 1360 1930

HIST1H2AC 533.666666666667 2283 551 885.766666666667

HIST1H2AD 3830 12800 5940 8040

HIST1H2AE 1550 5490 2960 1460

HIST1H2AH 4760 16000 6420 13000

HIST1H2AI 2.92 3260 1490 3010

HIST1H2AJ 5520 21400 6040 12400

HIST1H2AK 5340 18600 6960 13100

HIST1H2AL 353 2030 112 1040

HIST1H2AM 4050 8870 7940 10335

HIST1H2BA 2.88 11.9 4.25 8.42

HIST1H2BB 1040 5330 1070 2330

HIST1H2BC 1070 3840 1030 2080

HIST1H2BD 1680 6620 1650 3230

HIST1H2BE 20.4 1410 31.8 45.7

HIST1H2BF 504 1080 615 625

HIST1H2BG 965 3820 1140 1850

HIST1H2BH 4170 17900 4700 8000

HIST1H2BI 2750 13200 3030 6330

HIST1H2BJ 914 2670 1350 1860

HIST1H2BK 3085 18845 2945 6430

HIST1H2BL 5180 22600 6680 10800

HIST1H2BM 3120 15300 3920 7550

HIST1H2BN 616 1090 465 718

HIST1H2BO 3510 14700 3450 6890

HIST1H3A 12500 7660 11000 21100

HIST1H3B 9760 23200 7010 16100

HIST1H3C 13600 21700 26100 14800

HIST1H3D 11300 23500 13800 7870

HIST1H3E 14100 13200 9740 22900

HIST1H3F 4290 9250 6330 3720

HIST1H3G 5770 10900 9200 6380

HIST1H3H 1088.95 2819 1560.65 1454

HIST1H3I 600 1260 675 590

HIST1H3J 4530 5080 3230 4320

HIST1H4A 1641 3230.5 1050 2495.5

HIST1H4B 32000 45000 25300 53100

HIST1H4C 164000 220000 119000 252000

HIST1H4D 3680 7495 2470 8200

HIST1H4E 9410 16600 8070 13700

HIST1H4F 498 2340 1250 1570

HIST1H4G 19.8 37.2 20.6 24

HIST1H4H 2460 7640 3180 5500

HIST1H4I 1580 4700 2380 3820

HIST1H4J 1030 3020 970 2260

HIST1H4K 3142 9330 4420 7135

HIST1H4L 23309.5 31733.5 18380.55 42492

HIST2H2AA4 353 1590 943 655

HIST2H2AB 8180 17700 23300 19300

HIST2H2AC 5520 10700 18100 14800

HIST2H2BA 3.34 5.45 4.97 3.96

HIST2H2BE 73.14 142.5 142.15 104.47

HIST2H2BF 564.09 2029.40333333333 863.79 1156.06666666667

HIST2H3A 11300 33400 30300 30500

HIST2H3D 20300 35300 45400 38200

HIST2H4A 5.04 7.18 6.5 5.33

HIST2H4B 1790 7120 3910 5300

HIST3H2A 23.4 78.3 139 125

HIST3H2BB 59.1 215 564 534

HIST3H3 90000 78800 108000 117000

HIST4H4 32.2 120 4 98.8

HIVEP1 386 508 216 486

HIVEP2 298 614 647 803

HIVEP3 21.21 5.1 320.575 111.355

HJURP 19300 14100 16700 6930

HK1 3.89 24 1600 2340

HK2 3928.9 3519.85 1282.45 2770.45

HK3 2.73 4.4 5.34 6.35

HKDC1 4210 46.75 1415.5 77.725

HKR1 420 249 284 241

HLA-A 14696.6666666667 7784.83333333333 13242.5833333333 11438.3333333333

HLA-B 5050 2055 2115 11100

HLA-C 8765 3660 3095 27150

HLA-DMA 50 35.25 134.5 116.95

HLA-DMB 4.75 59.8 867 1190

HLA-DOA 10.725 16.73 18.075 950.55

HLA-DOB 54.2 68.7 66.8 133

HLA-DPA1 61.4 76.45 58.25 878

HLA-DPB1 194 46.975 6.6475 401.175

HLA-DPB2 48.6 18.5 26.4 59.6

HLA-DQA1 6.26 5.955 9 10.665

HLA-DQA2 5.77 5.54 9.47 13.4

HLA-DQB1 71 70.915 79 980.5

HLA-DQB2 62.6 32.4 49.8 226

HLA-DRA 3.43 5.5 5.12 14.7

HLA-DRB1 356.8 343.8 303.6 963

HLA-DRB3 325 331 324 400

HLA-DRB4 6.06 5.89 9.15 134

HLA-DRB5 11.4 9.29 6 590

HLA-DRB6 5.04 5.33 4.89 4.01

HLA-E 3840 1430 1190 7420

HLA-F 4301.9 792.1 661.905 3858

HLA-G 24000 4560 3040 26100

HLA-J 5102 1427 1089 6689

HLA-L 7.74 17.7 4.37 25.9

HLCS 792 778 1150 1460

HLF 739 606 41.3 121

HLTF 719 1580 3200 3090

HLX 10.5 4.2 21.2 72.9

HM13 2009.75 2128 1417.2 1939.75

HMBOX1 449.375 1421.55 808.2 741

HMBS 14300 10960 27400 25180

HMCN1 8.05 66.9 7.75 34.4

HMG20A 709 802 351 1020

HMG20B 6820 5213.33333333333 5813.33333333333 7860

HMGA1 18800 13800 27900 9280

HMGA2 2151.66666666667 3946 1627.33333333333 2878.7

HMGB1L1 21300 27000 41400 40000

HMGB2 11100 13100 5830 14700

HMGB3 827 1395 1679 4290

HMGB3L1 3390 4750 1660 13600

HMGB4 20.53 29.36 20.51 20.34

HMGCL 1050 1150 1420 520

HMGCLL1 30.5 18.75 24.05 54.95

HMGCR 12700 7170 6520 5330

HMGCS1 5240 8810 1780 1430

HMGCS2 15.019 21.04 137.5 7.934

HMGN1 14730 22220 21650 26650

HMGN2 26000 86900 111966.666666667 74466.6666666667

HMGN3 5090 5340 17100 10100

HMGN4 1380 938 185 1390

HMGN5 508.2 5.141 2113 2524

HMGXB3 4460 4580 6220 6890

HMGXB4 1440 1610 1080 2120

HMHA1 11200 6330 6940 935

HMHB1 442 533 419 568

HMMR 2990 6470 15300 6400

HMOX1 10383 9794 1838 15070

HMOX2 3760 2270 11100 6960

HMP19 22.6 13.1 6.6 16.3

HMSD 538 12.6 4.65 3.83

HMX1 6359.05 6155.2 5659.85 7170

HMX2 296 168 366 111

HMX3 91.3 88.9 84 112

HN1 74172.7272727273 82281.8181818182 215700 71354.5454545455

HN1L 3734.33333333333 3881 5950.66666666667 4191.33333333333

HNF1A 6144.85 5704 2316.3 23.1

HNF1B 99.5 90 321 50.5

HNF4A 182 105 10.1 4.61

HNF4G 25.825 11.765 24.02 4.125

HNMT 466.933333333333 684.2 52.6033333333333 4.38666666666667

HNRNPA0 1000 1770 817 3180

HNRNPA1 16966.6666666667 30306.6666666667 15476.6666666667 38986.6666666667

HNRNPA1L2 19580 33450 22570 43712

HNRNPA2B1 18280.2666666667 13506.4 15376.0666666667 20176.7333333333

HNRNPA3 11530 11348 9114 14794

HNRNPAB 3970 6310 8590 9670

HNRNPC 14420 16800 13916.6666666667 22256.6666666667

HNRNPCL1 1220 1350 1010 1390

HNRNPD 18651.5 17124 11954 31835

HNRNPF 5150 4190 4660 5540

HNRNPH1 18400 21700 24800 36100

HNRNPH2 1248.27272727273 2824.54545454545 993.181818181818 3123.63636363636

HNRNPH3 3353.63636363636 5337.27272727273 4677.27272727273 8575.45454545455

HNRNPK 11430 11365 9910 14950

HNRNPL 5180 7770 8910 9480

HNRNPM 12300 9870 10600 19200

HNRNPR 4820 6260 6640 8100

HNRNPU 10485 7045 8685 19200

HNRNPUL1 1770 2460 2180 2520

HNRNPUL2 277 205 199 235

HNRPDL 3805 5105 3275 8700

HNRPLL 479.666666666667 597.666666666667 1003.6 638.666666666667

HOMER1 192.3 193.5 420.5 352.5

HOMER2 326 5.76 648 1150

HOMER3 10449 11895.3333333333 2701.33333333333 2519.33333333333

HOMEZ 680 566 435 691

HOOK1 450 348.5 552.5 732.5

HOOK2 988 183 814 668

HOOK3 1440 2370 730 1170

HOPX 42.185 74.855 62.17 132.5

HORMAD1 3.31 5.36 4.92 4.035

HORMAD2 425 5.67 6.85 4.27

HOTAIR 3.56 16.1 5.25 101

HOXA1 10 7.65 16.2 22.2

HOXA10 2075.5 2169 1601.5 2595

HOXA11 3.24 72.2 81.7 3.89

HOXA11AS 3.26 90.2 118 3950

HOXA13 3.03 105 22.1 1250

HOXA2 142 88.2 862 164

HOXA3 1900 1310 4270 1290

HOXA4 273 149 2310 740

HOXA5 661 204 2860 3320

HOXA6 177 53.3 737 4420

HOXA7 1400 1790 107 2570

HOXA9 336.2 1205.5 248.95 7115

HOXB1 2.91 4.7 4.27 3.55

HOXB13 3.02 48.4 17.1 2970

HOXB2 6.125 30.71 3200 1180.5

HOXB3 2.67 4.34 271 139

HOXB4 17.1 19.4 2740 500

HOXB5 3.6 6.53 2570 660

HOXB6 2.69 73.4 43000 14900

HOXB7 3.16 5.13 176 73.2

HOXB8 2.79 4.55 2190 805

HOXB9 13.1 32.9 1370 16100

HOXC10 2.63 259 3.87 180

HOXC11 3.62 5.58 5.46 4.63

HOXC12 3.12 69.8 4.6 11.2

HOXC13 6.46 303 64.9 469

HOXC4 2.99 796 2400 3200

HOXC5 9.89 52.4 52.1 42.7

HOXC6 9.48 1570 29.5 2840

HOXC8 44.2 274 4.57 514

HOXC9 7.71 2390 4.19 4920

HOXD1 3.882 304.6 4.814 34.19

HOXD10 3.51 6 9.53 3110

HOXD11 5.27 6.03 12.2 455

HOXD12 2.57 4.15 3.78 3.17

HOXD13 3.73 38.6 7.77 11700

HOXD3 6.46 122 214 533

HOXD4 3.13 5.04 4.62 18.2

HOXD8 6.36 24.2 385 219

HOXD9 10.67 9.865 70.35 243.45

HP 220 597 43.8 57.3

HP1BP3 4800 3465 6145 2595

HPCA 3.84 6.13 5.61 17.4

HPCAL1 789 1110 1510 663

HPCAL4 3.38 81.4 17.1 115

HPD 1940 47.3 25.7 23.3

HPDL 19.2 22.9 21.9 29.3

HPGD 126 1450 3.98 24.7

HPGDS 10.019 18.81 11.278 10.259

HPN 1110 57.8 8.21 4.91

HPR 89.6 245 55.1 25.7

HPRT1 2917 3235 7235 22540

HPS1 252 570.666666666667 410 443.666666666667

HPS3 527.5 518.8 1146 451.4

HPS4 2095.05454545455 3216.09090909091 1732.7 3245.09090909091

HPS5 1010 1930 1310 1640

HPS6 4630 7480 9390 9260

HPSE 129 73 371 186

HPSE2 3.25 13.165 29.95 3.925

HPVC1 9.51 6.6 16 14.9

HPX 1140 574 19.6 11.5

HPX-2 9.485 15.135 11.365 12.425

HPYR1 3.87 6.13 5.85 4.92

HR 312.9 266.15 4370 224.15

HRAS 670.1 1074 2227 2392

HRASLS 5.88 10.3 35.3 1050

HRASLS2 5.995 43.55 16.2 10.284

HRASLS5 4817.5 4851 3447.5 2909

HRC 63.9 74.6 30.5 39.1

HRCT1 11.8 25 85.4 4.21

HRG 227 78.5 15.1 12.4

HRH1 35.3 41.4 115 37.2

HRH2 73.7 74.4 58.9 77.8

HRH3 27.96 40.145 40.945 38.04

HRH4 3.6 5.78 5.37 4.36

HRK 155 40.8 33 1570

HRNR 3.83 6.03 5.63 4.59

HRSP12 3970 6480 5550 4640

HS1BP3 555.65 251.75 279.75 773.5

HS2ST1 941.5 1546.5 688 943

HS3ST1 157 8.326 1795 4.207

HS3ST2 3.46 5.171 4.796 3.945

HS3ST3A1 79.1 163 4.76 335

HS3ST3B1 1921.15 486.9 6.055 297.9

HS3ST4 3.945 6.06 5.76 4.81

HS3ST5 10.955 16 5.93 4.3

HS3ST6 2.67 4.34 10.5 12.6

HS6ST1 6170 2620 39300 3660

HS6ST2 2844 777.5 1332 2354

HS6ST3 7.49 5.08 5.155 135.7

HSBP1 2101.81818181818 2664.54545454545 2968.18181818182 6391.81818181818

HSBP1L1 1200 1010 1600 61.4

HSCB 81150 87205 102580 94935

HSD11B1 2.59 4.19 3.8 3.18

HSD11B1L 187 78.6 202 237

HSD11B2 2180 312 435 168

HSD17B1 214 327 723 332

HSD17B10 9310 31300 10600 26000

HSD17B11 5050 5810 7940 2290

HSD17B12 7825 5345 3740 5055

HSD17B13 3.96 6.22 5.83 4.78

HSD17B14 50.2 240 71.2 112

HSD17B2 24.07 1320 15.263 6.431

HSD17B3 11.31 51.2 15.65 4.8

HSD17B4 5830 6930 4830 4870

HSD17B6 77.2 308 107 596

HSD17B7 677.916666666667 414.75 375.666666666667 479.75

HSD17B8 526 150 697 457

HSD3B1 3.45 5.62 8.51 6.65

HSD3B2 14.4 17.9 18.9 17.3

HSD3B7 5480 451 585 200

HSDL1 171 351 258 520

HSDL2 3100 3440 3830 2170

HSF1 15850.4 13787.5 17726.6333333333 18127.7666666667

HSF2 2060 2250 2390 6390

HSF2BP 108 25.3 63.1 17.6

HSF4 85.8 111 97.7 103

HSF5 3.09 4.94 4.57 3.75

HSFX1 13.9 18.4 19.9 22

HSFY2 3.345 5.325 4.92 4.055

HSFYP1 2.83 4.59 4.16 3.45

HSH2D 101.8 120.3 118.2 109.95

HSN2 112 58.2 151 45.8

HSP90AA1 77133.3333333333 79833.3333333333 93458.3333333333 92691.6666666667

HSP90AA2 37500 37200 48300 45400

HSP90AA5P 66.5 68.5 53 68.1

HSP90AB1 15000 15100 14540 16600

HSP90AB2P 15560 15360 14925 17175

HSP90AB4P 5.89 5.92 15.4 6.23

HSP90AB5P 16600 14500 14000 16700

HSP90AB6P 40.2 30.8 22.4 29.3

HSP90B1 26215 43470 41825 32140

HSP90B3P 4260 7300 7740 5050

HSPA12A 35.6 1270 5920 1400

HSPA12B 2.77 107 4.45 12

HSPA13 1680 1480 2470 1710

HSPA14 3500 5870 11600 10000

HSPA1A 5138 14630 96.64 152

HSPA1B 4930 4910 9300 78600

HSPA1L 60.7 65.9 48.2 48.6

HSPA2 79.6 1430 38.7 367

HSPA4 20025 18315 31300 24675

HSPA4L 597 693 712 1920

HSPA5 790 2340 1750 1500

HSPA6 46 33.4333333333333 29.1 38.6666666666667

HSPA8 18726.1 28923 31867.5 22974.5

HSPA9 9040 10600 12000 11200

HSPB1 30942 29059.5 32470 7142

HSPB11 4420 16000 10700 10000

HSPB2 3.35 5.37 4.99 9.77

HSPB3 3.58 5.77 5.28 114

HSPB6 63.8 66.7 66.1 58.7

HSPB7 3.47181818181818 5.54454545454545 5.13909090909091 4.45090909090909

HSPB8 174 463 6.35 138

HSPB9 49.4 53.1 46.8 109

HSPBAP1 620 553 789 806

HSPBP1 7120 7020 10800 8570

HSPC072 26.8 22.9 24.5 55.9

HSPC157 286 176 645 221

HSPC159 2490 4550 1650 3670

HSPD1 211000 180000 162000 174000

HSPE1 196000 153000 128000 119000

HSPG2 3231.725 2579.175 7953.6 160.015

HSPH1 15040 13635 18310 18460

HTA 19.3 13.1 15.5 18.8

HTATIP2 4426.5 5483.5 934.5 2843

HTATSF1 5000 9430 7300 28400

HTN1 3.22 5.16 4.74 3.9

HTN3 4.03 6.21 5.82 4.78

HTR1A 14.8 9.35 14.2 8.91

HTR1B 13.8 31.8 32.7 13.7

HTR1D 4.71 5.75 5.17 11.195

HTR1E 7.76 5.01 6.61 6.06

HTR1F 3.11 22.3 4.61 3.75

HTR2A 2.83 4.89 10.2 3.48

HTR2B 3.32 7.33 4.94 4.63

HTR2C 3.225 11.35 4.77 58.35

HTR3A 44.4 45 31.3 43.8

HTR3B 2.96 7.56 8.41 6.44

HTR3C 3.87 5.72 17.8 11

HTR3D 4.41 6.245 7.825 5.495

HTR3E 11.8 17.5 27.2 25.9

HTR4 6.21 6.56 6.21 6.835

HTR5A 3.08 5.01 4.55 3.76

HTR6 39.8 24.6 18.1 18.2

HTR7 30.4 20.1 980 222

HTR7P 41.95 39.55 501.55 159.35

HTRA1 108 8.81 34000 1770

HTRA2 2720 1950 1910 3750

HTRA3 8.965 51.005 13.34 51.06

HTRA4 27.4 4.54 18.4 3.39

HTT 1310 1300 284 1240

HULC 19100 6170 18.3 15.4

HUNK 4.61 4.53 8.44 24.4

HUS1 132 122 69.3 127

HUS1B 14.5 18.3 27.4 59.2

HUWE1 1061 2545 1191.5 2075

HVCN1 4.105 19.7 7.575 46.35

HYAL1 1420 189 327 32.2

HYAL2 25500 31200 23800 18200

HYAL3 231 619.5 1034.5 388.5

HYAL4 1551.66 1767.4 1579.2 1437.015

HYALP1 9.4 5.06 12.9 4.93

HYDIN 6.87285714285714 15.0257142857143 9.32714285714286 7.46142857142857

HYI 2380 3640 15.9 1596.5

HYLS1 260 852 1970 886

HYMAI 89.5 423 9.37 846

HYOU1 257 321 577 310

IAH1 3170 6070 8.73 3720

IAPP 169 167 152 147

IARS 3653.66666666667 6017.33333333333 5486.33333333333 7558.66666666667

IARS2 9880 6990 12200 10300

IBSP 3.25 5.29 4.82 3.97

IBTK 7440 4290 10200 4080

ICA1 2800 263.5 534 262.5

ICA1L 6.82 72.445 9.19 30

ICAM1 1189 1192 78.65 6.401

ICAM2 34.9 2150 9650 36.7

ICAM3 3120 1660 3840 928

ICAM4 71.8 160 9.74 462

ICAM5 47.2 56.57 55.59 186

ICK 1000 987 325 1020

ICMT 422 1890 1280 1280

ICOS 13.6 8.39 24.1 19.9

ICOSLG 257.7 48.805 214.9 149.3

ICT1 20600 20400 33200 22600

ID1 41500 151000 98200 11900

ID2 11720 16350 12065 20150

ID3 5810 9110 17900 5220

ID4 2090 2830 1580 5080

IDE 1690 2730 2680 2100

IDH1 24200 28400 5780 4070

IDH2 3610 2430 2810 688

IDH3A 540 2050 843 3170

IDH3B 2985 3535 3200 3875

IDH3G 708 1020 867 2720

IDI1 6180 4080 4670 2460

IDI2 12.9 18.5 59.1 23.1

IDO1 23.56 31.15 29.87 37.6

IDO2 6.48 6.85 4.99 4.04

IDS 94.8 409.4 225 835.5

IDUA 157 167 244 158

IER2 21000 13800 28400 11800

IER3 5595 10965 10335 472

IER3IP1 1860 2530 3260 2000

IER5 4450 8070 3140 14600

IER5L 1525.5 1905 7145 1574.5

IFFO1 2840 3590 175 7230

IFFO2 1070 5010 1020 1260

IFI16 20.3 19.7 11.9 11.1

IFI27 16.7 47.3 44.2 462

IFI27L1 1810 1020 4760 3890

IFI27L2 63.7 1360 6400 3050

IFI30 25200 7510 19500 5110

IFI35 405 406 115 232

IFI44 2.99 4.76 4.39 3.62

IFI44L 5.35 7.96 18.3 73.8

IFI6 327 162 312 244

IFIH1 893 18.7 70.4 40.6

IFIT1 49.7 163 1610 730

IFIT1L 3.87 6.12 15.7 4.94

IFIT2 26.7 36.63 109.95 11.055

IFIT3 17.6 41.7 149 34.2

IFIT5 13.3 1130 181 1220

IFITM1 6.57 10.37 3460 677

IFITM2 11900 1440 9560 3260

IFITM3 21700 2870 20200 2080

IFITM4P 6610 811 4810 925

IFITM5 4.39 7.3 17.2 13

IFLTD1 3.62 5.87 5.38 4.25

IFNA10 2.83 4.59 4.15 3.46

IFNA14 3.49 5.64 5.16 4.13

IFNA16 3.13 4.99 4.61 3.79

IFNA2 3.05 6.74 9.48 3.7

IFNA21 32.3 27.3 13.1 4.6

IFNA4 49.6 66.6 72.2 60.9

IFNA6 13.6 4.96 4.43 5.86

IFNA7 3.23 5.25 4.79 3.93

IFNA8 2.86 4.66 4.23 3.48

IFNAP22 2.61 4.23 3.83 3.2

IFNAR1 781 875 954 1230

IFNAR2 1760.8 2331.66666666667 400.05 2062.23333333333

IFNB1 5.45 5.49 5.04 6.1

IFNE 4.6 5.8 76.2 11

IFNG 5.574 7.454 6.692 4.203

IFNGR1 4130 1950 1170 3380

IFNGR2 818 648 861 877

IFNK 3.43 5.65 9.85 13.5

IFNW1 3.77 5.92 5.68 4.73

IFRD1 2220 3050 2030 1850

IFRD2 6130 7340 9820 10000

IFT122 299 670 808 526

IFT140 37.9 49.5 56.3 179

IFT172 828 683 1280 4750

IFT20 784 1770 911 1290

IFT52 1540 1550 2790 2960

IFT57 3.65 5.91 6.93 26.4

IFT74 44.33 80.575 116.7 199.8

IFT80 179 266 425 371

IFT81 183.3 441.65 802.5 947

IFT88 213 631 375 279

IGBP1 1771.33333333333 2338.33333333333 1135.33333333333 2623.33333333333

IGDCC3 3240 7860 56.7 1450

IGDCC4 287 146 10.2 270

IGF1 16.07 10.9536363636364 16.1109090909091 13.43

IGF1R 2260 2020 192 1450

IGF2 57794.5 11149.85 34.095 111.3

IGF2AS 228 131 81 106.8

IGF2BP1 6629 13870 13.156 12440

IGF2BP2 913.5 928 1428 633

IGF2BP3 20200 6740 7840 7410

IGF2R 5540 3160 771 1680

IGFALS 109 21.1 71.4 32.9

IGFBP1 471 215 897 3.23

IGFBP2 32.86 324.9 213.3 5265

IGFBP3 1246 465.2 3159 8.158

IGFBP4 15.3 722 14.3 29.3

IGFBP5 3.55 16.4 6.5 193

IGFBP6 21 258 1730 65.2

IGFBP7 94.3 5.67 25900 107

IGFBPL1 36.5 30.2 27.5 76.2

IGFL1 11.4 10.9 20.7 13.9

IGFL2 7.54 12.14 12.69 9.115

IGFL3 3.86 39.2 5.64 5.8

IGFL4 2.9 4.62 4.24 3.53

IGFN1 10.665 7.085 11.1 13.12

IGH@ 32.5 37 24.3 33.1

IGHMBP2 511 387 174 269

IGJ 47.6 96.5 32.3 41

IGKV1D-13 4.95 4.74 5.65 3.4

IGLL1 213 235.5 192.7 320.5

IGLON5 46 53.6 45.7 55.7

IGLV1-44 15.7 17.6 25.2 14.2

IGSF1 40.6266666666667 392.333333333333 49.0433333333333 156.166666666667

IGSF10 3.76 5.78 5.68 7.34

IGSF11 2.71 4.39 3.98 17.3

IGSF21 12.2 9.87 7.54 29.9

IGSF22 7.75 5.17 4.78 7.9

IGSF3 23.95 32.2 17.9 34.5

IGSF5 3.58 23.6 9.58 8.18

IGSF6 6.21 9.53 12.2 6.42

IGSF8 340.5 302 469.5 357

IGSF9 244 361 187 687

IGSF9B 666 956 1210 400

IHH 1440 942 17.8 67.3

IK 5580 6800 8100 10400

IKBIP 560.5 1005 2185 954

IKBKAP 348 375 83.2 323

IKBKB 737 611 784 984

IKBKE 44.1 22.3 27.7 25.4

IKBKG 1300 771 366 1010

IKZF1 10.7 13 13.2 8.26

IKZF2 99.3 21.31 48.2033333333333 23.9766666666667

IKZF3 2.56 4.15 3.76 29.5

IKZF4 343 180 216 272

IKZF5 192.5 265.5 193 353.5

IL10 18.941 23.204 18.39 12.214

IL10RA 2.93 4.66 4.3 88.1

IL10RB 704 552 600 606

IL11 2.8 89.7 25.1 5.19

IL11RA 128 48.2 53.3 163

IL12A 17.6 49.2 5.31 45.7

IL12B 11.6 6.32 5.59 4.55

IL12RB1 44.8 44.2 40.4 41.8

IL12RB2 5.53 6.43 8.58 65.1

IL13 12.2 11.6 15.6 6.55

IL13RA1 3668 5502.5 1774 3099.5

IL13RA2 3.66 42.4 8.49 669

IL15 75.4 906 198 27.7

IL15RA 256.333333333333 47.9166666666667 728.666666666667 113.133333333333

IL16 43.5525 74.945 45.04 52.185

IL17A 20.5 23.3 31.6 29.7

IL17B 4.98 5.89 7.44 4.97

IL17C 3.34 5.43 4.95 3.96

IL17D 256 109 12 272

IL17F 18.8 14.8 17 14.4

IL17RA 1278.26666666667 2480.9 1120.33333333333 1406

IL17RB 1740 873 193 137

IL17RC 291 214 268 106

IL17RD 207 38.4 189 305

IL17RE 230 117 111 103

IL17REL 103 103 108 106

IL18 64.31 1985 126.9 8.223

IL18BP 25.4 33.4 41.3 56.2

IL18R1 3.37 5.39 10.7833333333333 59.2666666666667

IL18RAP 198.585 190.585 148.595 187.3

IL19 13.3 8.27 9.71 8.65

IL1A 4.465 5.154 9.188 4.884

IL1B 7.012 7.08 5.928 6.432

IL1F10 19.9 26.3 29.7 32.2

IL1F5 3.59 5.75 5.41 4.4

IL1F6 24.3 21 25 24.5

IL1F7 13.5 13.2 26.3 14.2

IL1F8 10.5 4.72 21 9.61

IL1F9 3.94 6.19 10.6 5.35

IL1R1 151.8 228 315 28.75

IL1R2 10.1 47.4 635 5.98

IL1RAP 1255 917.5 431.5 110.65

IL1RAPL1 47.6 12.8 19.5 18.5

IL1RAPL2 10.2 8.84 11.9 4.19

IL1RL1 2.72 4.41 5.63 3.31

IL1RL2 9.86 72.2 9.03 8.8

IL1RN 42.45 25.35 34.55 30.705

IL2 5.151 6.043 6.303 5.481

IL20 8.94 11.7 5.63 4.63

IL20RA 25.5 8.69 5.77 4.71

IL20RB 138 576 343 410

IL21 24.7 39.9 26.7 23.3

IL21R 11.7 83.6 10.4 23.9

IL22 3.06 4.82 4.38 4.01

IL22RA1 305 34.4 71.1 9.76

IL22RA2 5.72 15.635 5.045 4.095

IL23A 49.1 71.3 390 208

IL23R 3.08 4.79 4.55 4.36

IL24 4.11 6.315 4.475 4.05

IL25 3.641 5.361 4.963 4.029

IL26 6.7 7.07 4.18 8.94

IL27 79.8 83.4 20.4 32.7

IL27RA 286 340 444 389

IL28A 56.1 65.9 98.4 67

IL28B 4.11 6.25 5.88 4.87

IL28RA 154.2 167.6 114.166666666667 190.266666666667

IL29 14.7 8.78 18 13.8

IL2RA 3.34 11.27 4.945 4.02

IL2RB 3.18 5.17 4.71 4.21

IL2RG 82.035 145.45 81.26 63.465

IL3 3.01 4.73 4.43 3.62

IL31 11.7 6.32 9.7 10.2

IL31RA 33.7 33.8 8.39 8.17

IL32 2025 2950 76.3 39.2

IL33 22.9 23.7 33.7 40.7

IL34 16.97 13.519 13.91 12.118

IL3RA 3.14 5.06 5.4 6.13

IL4 3.686 21.352 4.689 8.099

IL4I1 6.63 103 235 122

IL4R 2905.2 3747.5 2772.7 1164.7

IL5 10 99.9 13 8.38

IL5RA 30.5 5.75 5.3 4.34

IL6 11.546 13.631 11.815 17.863

IL6R 536 250 67 50.8

IL6ST 405.283333333333 480.066666666667 535.916666666667 266.116666666667

IL7 71.9 10.5 30.1 6.58

IL7R 3.33 5.43 4.94 3.94

IL8 508 344 4.88 3.96

IL8RA 14.1 9.8 18.9 19.3

IL8RB 4.42 5.19 4.56 12.5

IL8RBP 3.48 5.67 5.21 4.21

IL9 19.3 17.9 4.22 4.98

IL9R 67.2 73.1 64.6 95.8

ILDR1 40680 43260 40760 45790

ILDR2 4.75 7.1 7.89 42.4

ILF2 31200 35300 17500 39400

ILF3 6030 2903.33333333333 6611.33333333333 7063.33333333333

ILK 1871 2471 2053 1501

ILKAP 403 517 699 673

ILVBL 3000 1200 3340 3300

IMMP1L 992 2280 1410 1280

IMMP2L 658 735 1220 689

IMMT 549.9 608.666666666667 563.4 668

IMP3 10800 10800 7270 10700

IMP4 6190 4120 8980 4580

IMP5 4.89 5.53 11.7 3.87

IMPA1 2647 2510.5 2628.5 2417

IMPA2 2135.5 12695 13715 1220.5

IMPACT 477 330 544 948

IMPAD1 783 1564 1580 1897

IMPDH1 11900 28200 66200 40000

IMPDH2 4530 15100 8540 11400

IMPG1 3.2 5.23 4.76 3.85

IMPG2 21.6 14.2 16.7 14.3

INA 68.1 77 88.4 1280

INADL 687 358.3 1275.5 677.5

INCA1 21.9 17.9 28.2 15.1

INCENP 2039 1116 1635 1927

INE1 13.5 12.6 20.1 38.6

INE2 2.64 4.28 6.19 3.26

INF2 1474.66666666667 1344 2918.33333333333 1293

ING1 1020 495 1520 629

ING2 1580 1260 1040 2110

ING3 1078.63636363636 1897 2157.09090909091 3300.90909090909

ING4 327 229 491 519

ING5 978.5 1013.5 682.5 1503.5

INGX 3.13 5.1 4.65 3.79

INHA 25.3 17.3 29.2 33.6

INHBA 90.48 47.66 5.076 13.31

INHBB 4090 2050 20.6 114

INHBC 65.6 75 59.6 94.1

INHBE 683 66.9 17.1 16.2

INMT 10.69 8.055 7.53 11.875

INO80 109.1 112 104.9 104.5

INO80B 148 175 170 266

INO80C 782 827 442 1460

INO80D 604.2 597 438 468.5

INO80E 5750 5500 9330 10300

INPP1 978 1300 610 471

INPP4A 78.4 271 154 183

INPP4B 4.155 5.42 23.575 7.625

INPP5A 892.5 1403 715.5 911

INPP5B 165 115 242 269

INPP5D 2.81 4.55 4.65 91.5

INPP5E 2490 2270 2070 3980

INPP5F 232.333333333333 226.3 257.666666666667 422.333333333333

INPP5J 51.1 60.3 42.7 147

INPP5K 1260 1190 2600 1940

INPPL1 333 1121 1096.5 1194

INS 34.5 24.3 33 30.7

INS-IGF2 2.62 107 3.86 3.39

INSC 4.3 7.6 5.12 25.2

INSIG1 923 2730 1060 3540

INSIG2 1945 717.5 610 424

INSL3 74.7 97.3 106 102

INSL4 4.046 6.775 5.372 6.67

INSL5 3.94 6.25 9.42 4.77

INSL6 3.83 5.94 5.78 4.97

INSM1 10.367 5.136 4.757 57.69

INSM2 2.65 4.29 3.89 3.26

INSR 3000 855 206 787

INSRR 4.66 5.35 4.89 4.01

INTS1 14583.6 8181.2 4992.15 11684

INTS10 5340 8150 10300 7660

INTS12 913 1300 792 1280

INTS2 172 164 147 285

INTS3 1190 1230 789 1900

INTS4 1646.5 1251 1444 2183.5

INTS5 49.7 37.7 48.1 77

INTS6 266.366666666667 440.6 247.166666666667 254.066666666667

INTS7 1320 1040 755 1330

INTS8 701 733 899 1390

INTS9 2370 3240 4580 3160

INTU 115.1 204.1 148.85 242.7

INVS 513 1005 915.5 745.5

IP6K1 31500 37600 33900 42400

IP6K2 1372 4284 2223.5 2026

IP6K3 37.1 9.09 10.3 13.5

IPCEF1 6.155 5.57 8.155 4.285

IPMK 41.7 104 86.4 75.2

IPO11 132 191 363 354

IPO13 307 468 319 587

IPO4 3850 3580 3140 4450

IPO5 3960 1660 2440 3445

IPO7 1252 1259 1227 1560

IPO8 2920 2280 2010 4510

IPO9 63.4 353 161 510

IPP 162.95 344.25 278.8 272.35

IPPK 182.5 201 264.5 243

IPW 108 15.3 20.4 11.7

IQCA1 3.31 5.34 4.91 3.955

IQCA1L 5.33 5.81 5.52 4.38

IQCB1 1880 1320 1440 3310

IQCC 46.5 102 131 207

IQCD 27.3 260 405 274

IQCE 1121.5 735.5 814.5 1295

IQCF1 6.73 7.32 9.515 8.08

IQCF2 43.3 26.3 33.9 36.7

IQCF3 16.2 11.1 15.2 19

IQCF5 4.5 9.84 8.315 6.24

IQCF6 13.9 6.16 6.31 8.94

IQCG 33.4 52.2 117 43.5

IQCH 4.51 3878.01 21.36 23.25

IQCJ 2.88 4.675 4.265 3.53

IQCK 562 902 2350 1060

IQGAP1 5570 8610 8540 4850

IQGAP2 5110 4930 3120 3160

IQGAP3 3200 4680 4170 3300

IQSEC1 7540 7370 2380 3970

IQSEC2 3066.71666666667 2950.74 3291 2952.33333333333

IQSEC3 3.72 6 6.35 6.16

IQUB 15.5 8.41 53.8 8.32

IRAK1 952 1176.5 2060 2655

IRAK1BP1 120 97.7 393 282

IRAK2 6070 1620 75.6 403

IRAK3 3.72 5.94 5.53 4.51

IRAK4 83.6 108 216 86.8

IREB2 5430 7870 3410 7420

IRF1 120 50.9 219 86.3

IRF2 26.6 41.7 12.3 11.6

IRF2BP1 70.9 49.5 70.2 87.3

IRF2BP2 589 329 329 734

IRF3 916 1190 1845 864

IRF4 3.32 5.3 4.95 3.93

IRF5 226 115 5080 64.7

IRF6 2.9 4.66 9.79 3.53

IRF7 146 14.4 725 60.4

IRF8 17.5 22.1 4.28 418

IRF9 126 208 162 180

IRG1 14.7 29.2 26.1 31.1

IRGC 37.2 6.19 12 45.7

IRGM 3.21 5.16 8.135 3.9

IRGQ 127 121 79.2 90.4

IRS1 1085 856 112.45 30.05

IRS2 2160 4.18 2590 313

IRS4 3.64 5.6 5.48 658

IRX1 3.16 5.07 4.62 3.79

IRX2 3.375 5.475 5.03 4.07

IRX3 49 130 2690 704

IRX4 3.496 7.073 7.565 10.908

IRX5 49.1 57.5 1570 914

IRX6 12.2 11.6 63.6 34.7

ISCA1 718.666666666667 1030 940 837.333333333333

ISCA2 3590 4350 5410 8630

ISCU 8370 15300 15900 14200

ISG15 7780 5150 7190 420

ISG20 2550 2620 1810 758

ISG20L2 1920 2470 1640 3030

ISL1 585 70.8 278 898

ISL2 176 88.4 4.84 197

ISLR 121 113 115 142

ISLR2 18.6 22.975 27.6 38.43

ISM1 112 213 81.7 24.7

ISM2 14.5 15.6 20.2 35.9

ISOC1 3830 7590 9340 6640

ISOC2 1890 2670 4320 2560

ISPD 50.43 29.1066666666667 21.6066666666667 20.3833333333333

ISX 694 300 4.65 3.83

ISY1 839 1360 1030 1440

ISYNA1 697 426.5 2545 1219.5

ITCH 272 622 207 159

ITFG1 1250 1440 2390 1400

ITFG2 1070 559 1090 953

ITFG3 4200 3060 7240 3660

ITGA1 1510 329 45.2 105

ITGA10 10.2 62.5 187 56.8

ITGA11 47.8 39.8 39.8 43.3

ITGA2 173 321 235 27.9

ITGA2B 14.3 20.2 27.7 49.8

ITGA3 2.7 4.35 3440 43.2

ITGA4 3.64666666666667 5.81333333333333 5.46666666666667 100.3

ITGA5 464 407 695 230

ITGA6 1232 1325.5 2440 402.5

ITGA7 178 577 370 766

ITGA8 3.9 6.16 5.66 25.7

ITGA9 53.7 72.9 55.1 101

ITGAD 6.88 8.27 8.45 9.995

ITGAE 1600 4260 4130 3540

ITGAL 58.9 83 4.49 6.71

ITGAM 53.73 35.84 37.5 40.45

ITGAV 14130 11700 5248 1637

ITGAX 3.645 5.55 5.245 4.76

ITGB1 1685 2533 1716 523.5

ITGB1BP1 1201.99666666667 2459.37333333333 4560.58333333333 4224.56666666667

ITGB1BP2 28.2 143 145 188

ITGB1BP3 2.98 4.58 25.2 10.3

ITGB2 4.021 5.616 12.158 5.828

ITGB3 35.9 4.73 1980 8.5

ITGB3BP 570.4 2138 2190 5758

ITGB4 434 82.5 31500 132

ITGB5 3540 3050 899 649

ITGB6 7.23 18.2 4.92 7.84

ITGB7 148 158 146 90.4

ITGB8 2.75 8.18 808 6.81

ITGBL1 5.98454545454545 8.05818181818182 6.56636363636364 6.13

ITIH1 99.8 25.5 25.5 28.5

ITIH2 5890 2370 4.91 3.97

ITIH3 21.6 9.08 4.2 10.7

ITIH4 55.7 48.8 67.2 313

ITIH5 13.63 12.1566666666667 12.5066666666667 12.7666666666667

ITIH5L 38.5 45.4 44.9 63.6

ITK 3.58 5.54 5.42 4.74

ITLN1 8.61 13.7 9.53 9.01

ITLN2 7.22 13.5 3.9 7.49

ITM2A 3.14 5.1 4.65 118

ITM2B 4470 8150 6790 4130

ITM2C 3473 5121 1918 2852.5

ITPA 10900 14300 9550 10900

ITPK1 33.5 106.8 193.5 93.95

ITPKA 5870 4300 283 1050

ITPKB 42 35.3 795 863

ITPKC 1460 1930 1510 1150

ITPR1 677 543 534 457

ITPR2 270 105.9 107.05 245

ITPR3 2380 194 4670 590

ITPRIP 334 1090 715 978

ITPRIPL1 423 440 104 781

ITPRIPL2 1570 1550 4070 1480

ITSN1 196.666666666667 570 157.2 549.666666666667

ITSN2 160.5 160.5 172 156.5

IVD 3180 2950 5500 5810

IVL 7.12 4.37 5.24 8.06

IVNS1ABP 590.85 601.3 915.9 662.35

IWS1 1100 1100 1310 1020

IYD 10.16 25.25 13.345 14.045

IZUMO1 3.32 5.4 4.96 33.1

JAG1 2190 3950 4190 43.2

JAG2 478 26.88 133.7 213.4

JAGN1 15100 12000 11500 10700

JAK1 337 584 23.7 512

JAK2 92.9 85.4 246 145

JAK3 38.095 41.71 224.55 160.25

JAKMIP1 83.9 46.1 9.12 98.8

JAKMIP2 8.625 50.4 4.315 732

JAKMIP3 37.445 24.9 13.25 22.26

JAM2 12.2733333333333 4.75 8.54333333333333 139.5

JAM3 2.94 4.76 4.33 638

JARID2 1120 638 335 1720

JAZF1 3.08 41.7 98.1 376

JDP2 1631 1138 1906 1202

JHDM1D 106.25 195.55 49.475 605.75

JKAMP 850 2201.5 2259 1554

JMJD1C 251 302 273 195

JMJD4 8400 4290 4520 17000

JMJD5 729 909 1380 738

JMJD6 1720 1330 2140 1780

JMJD7 282 183 322 172

JMJD7-PLA2G4B 1620 1520 869 1050

JMJD8 417 419 914 786

JMY 207 200 467.5 836

JOSD1 7770 7960 7750 6010

JOSD2 27.2 46.3 124 56.6

JPH1 118 5.73 1150 2110

JPH2 20.05 19 45.1 21.55

JPH3 24.2 28.4 14.2 138

JPH4 11.715 14.37 10.46 11.15

JRK 605 474 666 839

JRKL 60.2 67.55 214.2 147.4

JSRP1 11.1 4.39 4.01 5.77

JTB 20000 27200 25300 37500

JUB 6250 16500 2550 1110

JUN 2690 2360 930 10300

JUNB 164 80.9 85.6 43.5

JUND 409 185 113 142

JUP 4660 1360 5950 1600

KAAG1 18.8 5.32 9.04 4

KAL1 3.42 5.47 5.1 4.3

KALRN 13.1166666666667 39.8066666666667 17.76 19.1333333333333

KANK1 5200 3060 3080 1830

KANK2 8240 3110 6760 3880

KANK3 24 24.6 35.6 98.4

KANK4 147.5 109.2 7.07 9.635

KARS 8620 14300 14800 13100

KAT2A 7160 4090 10400 12600

KAT2B 622 484 1490 372

KAT5 631 371 411 950

KATNA1 1969 2208 1337 4262

KATNAL1 667 952 1110 1520

KATNAL2 10.06 154.85 511 174.2

KATNB1 241 264 482 883

KAZALD1 67.7 304 777 293

KBTBD10 3.3 9.22 28.4 274

KBTBD11 600 5.22 64.6 2290

KBTBD12 3.195 5.13 4.73 3.82

KBTBD2 1450 638 543 445

KBTBD3 41.8 93.7 105 191

KBTBD4 760 715.5 411.5 893

KBTBD5 2.68 4.34 3.94 3.28

KBTBD6 1580 1480 2270 5230

KBTBD7 403 498 486 731

KBTBD8 80.7 107 62.7 332

KC6 3.27 17.7 4.85 3.9

KCMF1 458 1130 235 650

KCNA1 3.215 8.525 4.79 8.375

KCNA10 15.3 11.5 16.4 18.5

KCNA2 2.88 4.69 4.26 8.08

KCNA3 29.8 15.4 5.24 45.8

KCNA4 3.47 5.62 5.2 4.24

KCNA5 3.08 5 4.57 3.71

KCNA6 8.68 10.6 24.9 24.3

KCNA7 5.51 6.13 5.66 9.48

KCNAB1 6.61 10.7 5.21 13.2

KCNAB2 162.5 77.05 31.5 21.65

KCNAB3 20.2 24.1 36 41.9

KCNB1 2.97 4.78 4.35 9.66

KCNB2 6.79 5.41 13.9 150

KCNC1 3.33333333333333 6.17 4.86 159.136666666667

KCNC2 3.705 4.755 6.715 5.03

KCNC3 126.5 159 164 248

KCNC4 59.3 74.8 179 179

KCND1 3.49 5.66 5.2 4.25

KCND2 16.1 4.53 4.11 3.4

KCND3 134.495 124.78 138.585 110.24

KCNE1 5.93 8.68 20.2 13.5

KCNE1L 279 768 27.8 75.2

KCNE2 8.772 6.551 16.962 33.47

KCNE3 2.91 476 8430 3.53

KCNE4 11.3 9.7 9.68 4.12

KCNF1 3.65 5.91 5.46 31.5

KCNG1 18.1 23.3 42.4 2680

KCNG2 3.18 4.98 5 3.78

KCNG3 36.895 36.095 13.81 185.2

KCNG4 47.3 37.9 38 32.9

KCNH1 2.76 4.23 3.83 3.21

KCNH2 38.015 45.505 43.585 106.45

KCNH3 6610 6140 7530 5730

KCNH4 20.7 17.7 27 24.9

KCNH5 3.47 19.455 7.43 4.295

KCNH6 10.8 15.8 20.5 6.05

KCNH7 3.79 6.06 5.65 4.55

KCNH8 3.75 6 5.54 4.42

KCNIP1 3.6 6.44 62 4.23

KCNIP2 10.3 15.2 10.8 10.7

KCNIP3 3.18 5.16 4.71 329

KCNIP4 241 121 49.9 98.4

KCNJ1 3.15 5.13 4.67 3.85

KCNJ10 3.71 5.91 6.07 6.3

KCNJ11 8.43 7.88 13.7 33.6

KCNJ12 7.35 10.3 10.8 985

KCNJ13 23.4533333333333 21.93 26.0333333333333 24.6733333333333

KCNJ14 543 304 788 960

KCNJ15 9.77 14.25 10.48 10.24

KCNJ16 5.55 65 1450 5.55

KCNJ2 2.86 4.61 3560 25.9

KCNJ3 27.4 14.5 121 11

KCNJ4 6.29 4.25 11.7 5.58

KCNJ5 40.8 5.52 7.5 9.02

KCNJ6 3.67 5.83 5.43 4.42

KCNJ8 714 1910 4.44 1750

KCNJ9 3.53 5.67 5.21 4.18

KCNK1 915 4.73 9460 971

KCNK10 4.495 19.985 4.805 3.745

KCNK12 41.3 43.4 39.4 64.1

KCNK13 14.8 12.7 13.7 29.4

KCNK15 2131 2477 2661 1715

KCNK16 14.1 4.69 11.5 14.5

KCNK17 3.89 6.53 7.96 4.61

KCNK18 3.265 5.237 7.221 4.499

KCNK2 2.75 134 4.03 3.37

KCNK3 14.5 10.1 32.7 17.7

KCNK4 7.83 5.23 15.2 18

KCNK5 83.3 2140 5170 1900

KCNK6 2.62 5.18 3.85 3.22

KCNK7 11.5 20.5 19.2 39.1

KCNK9 15.8 18.3 19 22

KCNMA1 3.7525 5.53 185.3175 3.715

KCNMB1 441 511 274 582

KCNMB2 4 6.22 5.74 4.69

KCNMB3 226 154 97.5 67.8

KCNMB4 296 335 1380 4090

KCNN1 10.3 18.3 21.8 33.9

KCNN2 33.9 8.98 562 40.1

KCNN3 3.3 5.32 4.88 9.39

KCNN4 17.4 28.7 252 18.7

KCNQ1 4.89 4.67 19.8 134

KCNQ1DN 10.1 27 10.2 10.2

KCNQ1OT1 279 163 202 365

KCNQ2 3564.2 4556.3 4212.45 4339.2

KCNQ3 93.26 67.2 107.49 65.01

KCNQ4 11.2 4.19 13.6 7.63

KCNQ5 34.4 43.3 78.2 64.3

KCNRG 18.6 41.6 65.4 71.5

KCNS1 12.7 4.46 4.07 26.1

KCNS2 2.85 4.61 4.21 3.46

KCNS3 6.791 28.77 17.21 96.2

KCNT1 406.375 395.37 419.32 482.935

KCNT2 119 34.2 5.62 25.2

KCNU1 3.4 6.51 5.03 4.09

KCNV1 14.1 6.03 128 20.8

KCNV2 22.1 19.4 33.7 9.4

KCP 871.926666666667 927.663333333333 1521.03333333333 763.186666666667

KCTD1 405.5 558.5 7415 1551.5

KCTD10 367 588 409 589

KCTD11 93.8 282 372 260

KCTD12 6.255 6.5 130 11530

KCTD13 51.7 33.6 53.8 59.7

KCTD14 61.3 46.6 12.3 17.1

KCTD15 594.5 300 5.845 433

KCTD16 17.15 10.325 7.16 11.695

KCTD17 80.2 177 103 287

KCTD18 79.6 95.6 132 127

KCTD19 1730 2400 1760 2140

KCTD2 189 244 494 298

KCTD20 2200 1970 980 1930

KCTD21 495 1120 536 580

KCTD3 4044.66666666667 1795 1576.33333333333 2882.33333333333

KCTD4 5.16 4.79 4.41 3.59

KCTD5 1431 1388 1393.5 1456

KCTD6 1190 1110 649 1760

KCTD7 42.5 92.5 25.9 43.2

KCTD8 683 221 5.22 4.28

KCTD9 800.333333333333 1330.66666666667 2652 1259.33333333333

KDELC1 2046 798.4 1178 1148

KDELC2 625 911 5.665 1448.5

KDELR1 4740 6000 5710 4850

KDELR2 11000 12800 10100 2630

KDELR3 1300 1510 1510 976

KDM1A 457 339 396 516

KDM1B 128.05 61.35 95.55 121.7

KDM2A 2670 3350 989 832

KDM2B 196.5 195 193.5 550.5

KDM3A 210 137 215 283

KDM3B 1990 1920 2500 2840

KDM4A 1290 768 712 1350

KDM4B 2163.5 1171.8 1344.75 2709.1

KDM4C 909.245454545455 353.509090909091 522.209090909091 733.709090909091

KDM4D 55.5 34.3 199 451

KDM5A 7.87 5.52 5.14 13

KDM5B 2000 775 415.5 1317.5

KDM5C 458.4 1337.85 650.55 1688

KDM5D 74.7 84.8 49.3 53.8

KDM6A 455.5 514 154.1 569

KDM6B 169 127 24.9 54.4

KDR 4.22 6.62 7.49 9.27

KDSR 283.5 178.35 731.5 584.5

KEAP1 4054 1970 3597 2565

KEL 3.85 6.17 5.64 4.51

KERA 2.96 4.74 4.85 3.6

KGFLP1 777 182 712 763

KGFLP2 102 119 77.7 158

KHDC1 7.43 19.5 50.7 66.4

KHDRBS1 13800 16800 14800 23800

KHDRBS2 76.7 5.08 339 11.7

KHDRBS3 611 319.5 406 1375

KHK 604 267 47 561

KHNYN 1230 1260 686 1480

KHSRP 37314.5 16698.5 20990 39397

KIAA0020 5550 2900 6730 6660

KIAA0040 33.645 41.145 150.775 159.25

KIAA0087 15.3233333333333 19.03 21.1066666666667 19.7766666666667

KIAA0090 1073 998.5 1395 679

KIAA0100 9280 12200 11500 10200

KIAA0101 11240 10570 20140 4244

KIAA0114 13200 17000 3160 5640

KIAA0125 6.185 10.885 6.97 4.36

KIAA0141 803 884 1048 1345.5

KIAA0146 351 421 616 612

KIAA0174 336 404 417 370

KIAA0182 575 1040 267 611

KIAA0195 251 214 370 384

KIAA0196 2810 3116 2730 4106

KIAA0226 823.2 755.6 1207.6 721.35

KIAA0232 1350 2310 483 1060

KIAA0240 616 425 313 309

KIAA0247 2010 938 901 1010

KIAA0284 12000 7850 14300 6940

KIAA0317 630.45 554.45 689.55 909.5

KIAA0319 2.89 7.61 5.7 9.31

KIAA0319L 595 692 599 769

KIAA0355 1670 1940 3120 2100

KIAA0368 1206.8 1354.75 1208.9 1696.5

KIAA0391 1780 1520 1590 1340

KIAA0406 3489 3754 3623 3236

KIAA0408 2.53 4.1 3.72 194

KIAA0415 3369 1300.85 1325 1056.85

KIAA0427 274 579 319 467

KIAA0430 2690 2010 1820 2480

KIAA0467 16.9 21.3 37.6 34.9

KIAA0485 475 254 57.3 515

KIAA0494 1070 3300 2520 1650

KIAA0495 102 149 163 547

KIAA0509 15.9 9.41 14.5 33.4

KIAA0513 528 1210 1360 1100

KIAA0528 1160 1430 1920 2280

KIAA0556 39.25 46.15 62.5 58.75

KIAA0562 207 151 293 226

KIAA0564 336 320 316 589

KIAA0586 1441 2165 1286 2704

KIAA0649 189.3 35.81 760.25 574.9

KIAA0652 484.5 309 272 389

KIAA0664 1270 971 1450 1300

KIAA0748 25.8 24.1 34.7 47

KIAA0753 876 709 1240 1080

KIAA0754 128 89 208 78.1

KIAA0776 1720 1410 1160 1700

KIAA0802 499 202 271 165

KIAA0831 60.6 102 92.5 80.5

KIAA0892 2070 2380 639 2280

KIAA0895 86.4 74.4 135 176

KIAA0895L 347 478 614 1270

KIAA0907 1029 869.5 638.5 1247

KIAA0913 2880 4270 1710 2820

KIAA0922 2020 2480 1020 1880

KIAA0947 991 981 1280 1340

KIAA1009 172 140 132 200

KIAA1012 61.1 41.1 39.6 328

KIAA1024 46.2 102 141 487

KIAA1024L 3.1 5.05 4.58 13.4

KIAA1033 1809 3124.5 6505 1815.5

KIAA1045 2.96 221 4.39 3.59

KIAA1107 14.4 19.8 19.1 36.5

KIAA1109 377 480 370 354

KIAA1143 1580 4520 2410 2580

KIAA1147 1880 709.5 1116 2115

KIAA1161 1936.05 839.75 782.2 1015.7

KIAA1191 5120 14500 11400 13200

KIAA1199 14.27 32.17 4.43 3.52

KIAA1210 2.92 4.76 4.32 3.54

KIAA1211 30.9 15.9 5.73 20.4

KIAA1217 145.45 109.6 129.45 45.65

KIAA1239 4.265 5.165 4.72 3.875

KIAA1244 309.666666666667 224.666666666667 83.8666666666667 93.9

KIAA1257 14.7 33.7 4.62 35.6

KIAA1267 3200 2390 2180 3230

KIAA1274 79.8 98 265 618

KIAA1279 1430 2910 3180 3990

KIAA1310 377 320 306 431

KIAA1324 47.05 145.6 40.055 44.15

KIAA1324L 4.43 663 181 522

KIAA1328 53.7 44.3 124 147

KIAA1370 1430 444 301 472

KIAA1377 3.59 33.7 25.6 22.7

KIAA1383 4.58 5.49 5.03 4.05

KIAA1407 30.8 10.9 73.4 35.4

KIAA1409 3.67 5.91 5.48 4.47

KIAA1429 4683 4006 3724.5 6177.5

KIAA1430 2494.5 1240 982.5 1320

KIAA1432 323 383 305 366.333333333333

KIAA1462 23.9 610.5 41.4 475.5

KIAA1467 117 186 519 740

KIAA1468 414 424 691 553

KIAA1486 3.31 5.4 47.7 3.97

KIAA1522 9500 8670 11500 4550

KIAA1524 32.4 45.7 23.6 23.8

KIAA1529 458.5 1146 1549 1343.5

KIAA1530 1115.6 812.05 262.05 717.7

KIAA1539 68.05 185.7 164.9 50.05

KIAA1543 760 193 196 181

KIAA1549 798 1020 107 1370

KIAA1586 73.4 52.8 17 163

KIAA1598 498.6725 577.3575 446.725 395.525

KIAA1609 198.05 424.45 595.35 922.85

KIAA1614 30.9 28.6 32.9 40.8

KIAA1632 84.21 146.8 84.25 89.8

KIAA1644 9.68 5.795 8.915 92.995

KIAA1654 65500 73800 74800 74700

KIAA1659 33.2 5.52 5.1 9.03

KIAA1661 3.33 5.4 4.97 4.04

KIAA1671 584.5 557 334.25 381.2

KIAA1683 55.75 38.25 38.75 29.45

KIAA1688 219 250 367 461

KIAA1704 91.2 216.3 311.8 400

KIAA1712 148.5 187.1 75.35 568.5

KIAA1715 232.15 297.75 309.95 362

KIAA1731 1130 1400 2370 2000

KIAA1737 1250 1450 1510 2890

KIAA1751 17.54 13.07 5.355 12.22

KIAA1755 12.9 12.8 21.4 18.4

KIAA1797 2600 3770 4410 6650

KIAA1804 4260 3970 3530 4100

KIAA1826 718.636363636364 530.545454545455 215.136363636364 986

KIAA1841 144.606666666667 103.67 58.2 74.4533333333333

KIAA1875 2979.655 2684.9 2604.765 2659.1

KIAA1908 164.5 80.2 57.95 73.7

KIAA1919 377 306 421 274

KIAA1949 1078.95 808.7 1956.5 296.4

KIAA1958 292 328 322 243

KIAA1967 529 677 450 560

KIAA1984 5.805 15.385 11.685 12.01

KIAA2013 2680 2119 4270 2099.5

KIAA2018 658 644 428 525

KIAA2022 3.05 4.96 4.51 31.2

KIAA2026 127 74 116 206

KIDINS220 414.5 506 312 708.5

KIF11 1370 2540 2360 2790

KIF12 25.9 123 70.3 24.7

KIF13A 219 63.7 83.5 47.3

KIF13B 3011.5 2781 3258 578

KIF14 3300 2120 2260 2710

KIF15 1030 2150 2060 1490

KIF16B 591 1260 494 955

KIF17 46.1 48.8 61.5 264

KIF18A 678 1060 964 676

KIF18B 25.95 67.95 51.9 50.1

KIF19 11.13 20.35 9.51 28.3

KIF1A 1150 12.4 9.38 1140

KIF1B 1082.25 1047.175 962.225 1022.5

KIF1C 52500 34100 39900 32600

KIF20A 1422 1353 3922 1878

KIF20B 439.5 790.5 761 832.5

KIF21A 339.3 605.7 251.8 197.5

KIF21B 122.55 132.5 51.65 89.4

KIF22 2060 1648.66666666667 3331.33333333333 2532.66666666667

KIF23 3150 7855 7600 6410

KIF24 343 240 437 864

KIF25 3.49 27.8 5.18 63.1

KIF26A 3.04 4.95 4.49 676

KIF26B 26.4 20.1 91.7 39.1

KIF27 20.6 14.8 31.3 36.4

KIF2A 1400 1810 2840 1860

KIF2B 9.281 8.968 16.467 6.365

KIF2C 7840 9190 10700 8030

KIF3A 466 1110 1660 3610

KIF3B 2700 4180 2830 2470

KIF3C 94.345 100.6 100.425 234.95

KIF4A 1272.5 1614.5 1916.5 1712

KIF5A 7.67 18.6 19.8 48

KIF5B 3633.5 4290 4580 3037.5

KIF5C 25.6 124 5.85 685

KIF6 14.9133333333333 8.82 5.35 17.0833333333333

KIF7 77.2 235 4.86 605

KIF9 3.465 10.285 17.945 6.76

KIFAP3 1790 1290 1700 3970

KIFC1 1790 1070 1810 1140

KIFC2 83.75 48.55 168.35 89.75

KIFC3 129 387 368 101

KILLIN 62 77.4 201 128

KIN 1360 1870 2000 2330

KIR2DL2 5.1 8.28 4.29 3.51

KIR2DL4 23.1 21.7 24.15 18.55

KIR2DL5A 19.585 6.795 14.62 13.5

KIR2DS2 7.58 8.7 12.2 13.9

KIR2DS3 3.2 5.22 4.74 3.85

KIR2DS4 19.8 23.35 38.45 21.05

KIR3DL1 28.4 27 14.3 21.3

KIR3DL2 927 1040 1070 987

KIR3DL3 7.56 4.23 20.2 20.2

KIR3DP1 47 46.35 68.8 82.75

KIR3DX1 32 38.9 20.8 44.4

KIRREL 114 69.2 637 156

KIRREL2 501.91 497.075 738.55 421.3

KIRREL3 16.1845454545455 24.3763636363636 20.2481818181818 17.4436363636364

KISS1 50 125 63.8 21.1

KISS1R 1330 11.3 5.34 5.46

KIT 3.47333333333333 5.45166666666667 5.0175 561.866666666667

KITLG 183 1620 4150 606

KL 10.2 6.42 4.76 3.89

KLB 1780 1660 4.59 3.94

KLC1 582 1020 1680 1600

KLC2 3319.86666666667 4671.33333333333 1969.26666666667 2856.13333333333

KLC3 68.1 80.2 31.7 653

KLC4 31.4 28.7 24 43.4

KLF1 203 151 124 187

KLF10 954 1230 1430 705

KLF11 63.9 461 162 1190

KLF12 116.3 264.2 478.4 972.5

KLF13 7190 3790 6930 2460

KLF14 21.1 30.6 9.89 36.7

KLF15 24.5 104 12.3 13.5

KLF16 8280 8500 7570 5040

KLF17 12700 13000 12300 14100

KLF2 824.9 2094 191.6 189.6

KLF3 4760 3740 1570 2360

KLF4 46.5 106 59.6 87.5

KLF5 786 1610 2960 670

KLF6 3957.05 1414.11 3744.95 897.6

KLF7 216 194 4.46 50.8

KLF8 12.88 17.2 13.555 27.7

KLF9 657 257 334 2930

KLHDC1 2.58 7.43 26 3.17

KLHDC10 174 212 193 181

KLHDC2 2820 3210 3630 3440

KLHDC3 610 368 517 666

KLHDC4 1776 2143.5 3635 3594

KLHDC5 248 342 593 484

KLHDC7A 17.5 24.7 22.4 22.6

KLHDC7B 23.1 22.85 25.8 24.515

KLHDC8A 62.2 5.19 6.52 13.3

KLHDC8B 80 253 203 117

KLHDC9 240 194 1720 223

KLHL1 3.61 5.58 5.46 4.76

KLHL10 3.49 5.64 5.22 4.6

KLHL11 147 328 257 592

KLHL12 1012.5 741.35 1340 982.5

KLHL13 160 210 114 561

KLHL14 3.68 5.88 808 62.5

KLHL15 15.2 59.3 26.9 110

KLHL17 1660 1230 1410 1540

KLHL18 912 1150 1430 1420

KLHL2 526 883 267 458

KLHL20 385 323 212 277

KLHL21 5900 5690 5150 5570

KLHL22 163 282 65.5 188

KLHL23 921 751 182 1405

KLHL24 802 199 181 377

KLHL25 2970 3460 1430 2680

KLHL26 80.4 92.6 185 237

KLHL28 422 307 293 311

KLHL29 3719.5 1172 1204 1765.5

KLHL3 2.77 272 4.09 263

KLHL30 3.42 21.8 64.2 18.7

KLHL31 96 78.3 32.6 79.7

KLHL32 9.735 4.505 8.375 14

KLHL33 136 163 135 134

KLHL34 191 48.6 16.1 17

KLHL35 34.3 37.7 527 43.3

KLHL36 231.85 108.55 315 223.95

KLHL38 28.3 34.9 35.1 29.5

KLHL4 6.13 6.88 5.77 8.8

KLHL5 3823.63636363636 2732.72727272727 306 380.090909090909

KLHL6 6.32 8.18 10.125 5.78

KLHL7 863 500 732.5 693.5

KLHL8 1010 1260 2430 2020

KLHL9 2290 1610 3940 6610

KLK1 3.88 6.14 46.7 82.2

KLK10 33.7 24.6 29.1 25

KLK11 3.831 6.585 1702 4.153

KLK12 3.09 5.02 4.56 5.61

KLK13 12.575 7.305 15 14

KLK14 3.61 5.76 5.32 4.29

KLK15 15 6.17 12.6 10.7

KLK2 31.91 29.19 29.86 26.51

KLK3 10.835 7.95 10.825 14.22

KLK4 26.7 28.5 18.3 27.4

KLK5 16.3 25.5 27.4 23.6

KLK6 15.255 8.14 15.3 12.75

KLK7 16.6 5.64 11 45.1

KLK8 7.38 12.3 25.6 1490

KLK9 17.5 19.6 22.3 20.4

KLKB1 34.9 10.7 13.3 13.3

KLKBL4 3.68 5.84 5.55 4.56

KLKP1 8.78 7.28 9 6.54

KLRA1 33.6 28.2 38.2 64.6

KLRAQ1 390.666666666667 195.333333333333 292 278

KLRB1 8.16 10.4 14.5 11.3

KLRC1 13.32 598.3 8.15 13.75

KLRC3 5.72 518 5.03 6.26

KLRC4 8.54 942 9.26 15

KLRD1 3.14 5.02 4.685 3.93

KLRF1 3.48 5.61 8.97 4.285

KLRG1 26.15 118.195 215.62 264.8

KLRG2 239.65 286.565 188.735 534.5

KLRK1 2.74 4.44 4.03 3.36

KMO 52 57.75 49.3 40.75

KNCN 8.75 13.775 9.79 6.87

KNDC1 5.975 9.55 9.285 7.055

KNG1 476 453 13.9 19.9

KNTC1 1415 1268 1978 2011

KPNA1 199 259 153 302

KPNA2 76800 92800 77900 81800

KPNA3 2710 5260 2820 5060

KPNA4 16200 13100 14600 8770

KPNA5 215.5 219.5 95.2 596.5

KPNA6 2999 5560 4915 6875

KPNA7 12.26 105.9 4.56 4.24

KPNB1 33250 38850 21500 43250

KPRP 2.63 5.56 3.86 3.23

KPTN 768 541 349 639

KRAS 1521.5 1476.5 1071.5 1131.5

KRBA1 1460 3490 3070 8800

KRBA2 6370 6770 6360 6470

KRCC1 1180 1250 1930 300

KREMEN1 59.87 141.103333333333 312.1 533.256666666667

KREMEN2 18.5 116 1120 425

KRI1 4080 2660 4250 9270

KRIT1 15.8 38.8 12.4 51.7

KRR1 298 425 290 519

KRT1 2.66 4.33 3.92 3.26

KRT10 19600 28200 23500 19200

KRT12 7.03 15.8 7.93 5.96

KRT13 14.4 17.4 26.9 17.7

KRT14 21.3 16.1 5.46 4.49

KRT15 241 339 453 234

KRT16 6.02 7.42 11 8.51

KRT17 20.6 18 5.53 4.18

KRT18 61050 122250 54850 8565

KRT19 20800 101000 105000 17.9

KRT2 9.348 5.326 5.865 3.587

KRT20 8.36 17.1 5.01 4.44

KRT222 45.4 675 4.86 2070

KRT23 12980 3128.5 25.8 6.11

KRT24 3.01 4.85 4.46 3.69

KRT25 3.92 4.57 20 7.11

KRT26 28 24.3 20.6 24.4

KRT27 2.95 4.81 4.36 28.9

KRT28 5.97 7.04 8.35 7.61

KRT3 240 173 308 214

KRT31 60.3 64.4 51.5 52.5

KRT32 37.4 21 29.4 22.5

KRT33A 39.5 57.9 69 48.8

KRT33B 12 17.3 18.9 17.9

KRT34 6.764 7.764 5.402 6.2

KRT35 4.6 4.71 4.45 7.14

KRT36 3.65 4.39 3.98 4.56

KRT37 3.946 9.855 4.983 4.263

KRT38 3.55 5.582 5.204 4.285

KRT39 41.3 40.8 45.2 37.4

KRT4 32.5 26.6 26.2 26.4

KRT40 3.035 4.94 21.16 3.715

KRT5 3.06 4.97 4.52 3.66

KRT6A 11.9 12.1 16.7 8.34

KRT6B 3.67 5.77 8.33 4.59

KRT6C 33.2 4.45 8.59 4.67

KRT7 6520 6510 5190 5300

KRT71 16.1 25.2 27.6 33

KRT72 2.93 4.78 4.33 3.55

KRT73 1650 1580 1490 1420

KRT74 22.6 24.4 31.4 28.1

KRT75 3.75 6.05 5.55 4.41

KRT76 37.1 31.5 32.8 35.2

KRT77 3.28 5.29 4.88 4.01

KRT78 99.6 129 130 129

KRT79 10.2 15.5 6.21 5.78

KRT8 6655.5 14640 2238 467.75

KRT80 191 32.3 5.04 4.01

KRT81 18100 19100 15900 13700

KRT82 3.89 6.15 5.66 5.5

KRT83 277.26 656.9 108.8 16.545

KRT84 4.09 6.38 6 4.96

KRT85 164 150 162 188

KRT86 64 159 95.7 80.2

KRT8P12 3590 6720 1280 186

KRT9 95.1 85.3 81.8 70.3

KRTAP1-1 2.74 4.43 4.02 3.36

KRTAP1-3 2617.53333333333 4824.73666666667 3944.60666666667 3024.4

KRTAP1-5 14.21 15.6 16.63 15.52

KRTAP10-1 851 592 274 509

KRTAP10-10 802 531.5 554 514

KRTAP10-11 3.8 5.98 5.57 4.52

KRTAP10-12 712.15 431.9 291.8 275.2

KRTAP10-2 71.1 80.2 83.9 87.7

KRTAP10-3 44.8 37 44.2 45.9

KRTAP10-4 10.68 11.505 9.21 12.595

KRTAP10-5 66.7 63.8 62.3 54.1

KRTAP10-6 64 54.9 63.1 45.5

KRTAP10-7 8.41 17.8 18.2 13.9

KRTAP10-8 180 245 192 538

KRTAP10-9 121 171 266 164

KRTAP11-1 66.3 69.2 80.5 89.5

KRTAP12-1 25.5 32.8 19.8 28.7

KRTAP12-2 172 189 161 184

KRTAP12-3 3.27 4.74 7.83 11.9

KRTAP12-4 15.8 10.5 15.6 18.8

KRTAP13-1 12 4.73 8.82 7.98

KRTAP13-2 20.5 22.4 24.4 31.6

KRTAP13-3 3.8 6.1 5.72 5.59

KRTAP13-4 29.9 33.9 43.9 33.5

KRTAP15-1 2.66 4.24 3.85 3.21

KRTAP17-1 3.76 6.07 5.65 4.65

KRTAP19-1 25.5 61.5 62.8 64.1

KRTAP19-2 59.3 2910 2050 1210

KRTAP19-4 3.65 5.87 5.45 4.42

KRTAP19-5 1040 1710 2890 2700

KRTAP19-6 2.9 4.73 4.28 3.51

KRTAP19-7 42.8 57 41.8 43.4

KRTAP19-8 288 285 196 116

KRTAP2-1 134 144 121 151

KRTAP2-2 3.96 6.17 5.81 4.77

KRTAP2-4 5157.55 4809.66 3748.015 4080.25

KRTAP20-1 53 57.2 40.3 48.1

KRTAP20-2 240 377 246 304

KRTAP20-3 9.79 14.1 5.38 4.39

KRTAP20-4 10.1 78.4 14.7 63.1

KRTAP21-1 11.525 60.4 53.18 27.125

KRTAP21-2 19.5 42.9 16.3 20.7

KRTAP22-1 14.2 13.5 4.92 3.49

KRTAP22-2 2.72 4.42 4 3.32

KRTAP23-1 48.8 54.6 63.1 73.2

KRTAP24-1 12.7 26.6 43 8.63

KRTAP25-1 3.48 5.76 17.7 18.6

KRTAP26-1 6.32 10.5 4.02 5.42

KRTAP27-1 5.34 5.64 5.17 4.22

KRTAP3-1 82.5727272727273 51.6 38.5336363636364 32.6463636363636

KRTAP3-2 15.9 6.44 7.41 11.8

KRTAP3-3 11251.95 9002.6 28652.395 8401.95

KRTAP4-1 7.11636363636364 177.636363636364 13.3109090909091 8.20272727272727

KRTAP4-11 211 215 239 193

KRTAP4-12 68.3 61.3 71.2 126

KRTAP4-2 58.9 18.6 29.7 17.5

KRTAP4-3 18.93 18.595 14.8 16.365

KRTAP4-4 3.66 5.68 5.51 4.63

KRTAP4-5 6.17 11.3 13.6 16.9

KRTAP4-7 53.4933333333333 94.8233333333333 60.2833333333333 60.64

KRTAP4-8 44.73 48.54 47.39 46.305

KRTAP4-9 2.76 4.47 4.05 3.38

KRTAP5-1 20.3 21.5 23.2 17.3

KRTAP5-10 25.4 31.3 30.8 20.4

KRTAP5-11 5.36 4.77 8.77 6.69

KRTAP5-2 751 792 644 876

KRTAP5-3 3.93 7.57 29.3 3.99

KRTAP5-4 590.2 658.295 360.9 552.1

KRTAP5-5 38.4 32.45 30.85 31.15

KRTAP5-6 34.1 25.1 38.3 41.1

KRTAP5-7 51.5 38.7 75.7 64.8

KRTAP5-8 5.2 4.93 4.54 3.74

KRTAP5-9 13.4 24.4 17.2 12.1

KRTAP6-1 63 100 90.4 94.8

KRTAP6-2 54.1 77.4 66.9 97.8

KRTAP6-3 184 254 214 221

KRTAP7-1 9.43 14.6 14.2 4.8

KRTAP8-1 3.82 6.07 5.66 4.62

KRTAP9-3 3.493 7.269 5.184 4.256

KRTAP9-4 12.4 5.61 5.18 4.17

KRTAP9-8 3.01 4.89 4.43 3.63

KRTAP9-9 12.6 18.8 15.3 14.7

KRTAP9L1 37.85 45.7 37.75 33.85

KRTAP9L2 1010 865 1170 893

KRTCAP2 18200 27600 19000 30900

KRTCAP3 23.3 25.3 24.6 4.48

KRTDAP 31.7 5.03 27.4 153

KSR1 5.065 24.915 11.44 12.865

KSR2 4.85 12.635 8.85 33.9

KTELC1 1200 568 1230 1490

KTI12 366.6 499.2 449 658

KTN1 5970 13100 11000 11400

KU-MEL-3 375 386 322 279

KY 38.1 83.2 15.6 16.1

KYNU 128 1690 14 8.22

L1CAM 8.01 12.92 296.5 15.025

L1TD1 2.94 4.7 4.32 3.57

L2HGDH 3253.88333333333 4182.95833333333 3513.26666666667 3871.41666666667

L3MBTL 53.15 63.85 106.7 162.4

L3MBTL2 240 313 368 688

L3MBTL3 103 62.7 109 123

L3MBTL4 79.55 5.745 5.5 4.6

LACE1 449 203 389 672

LACRT 3.77 5.92 5.69 4.76

LACTB 1590 959 507 373

LACTB2 2270 4470 3960 2420

LAD1 19400 6320 21500 3.58

LAG3 56.1 32.7 179 21.6

LAGE3 5530 22300 3100 28800

LAIR1 40.395 43.715 51.755 54.22

LAIR2 12.3 5.7 5.3 4.29

LALBA 3.495 5.279 5.196 16.036

LAMA1 5.44 9.655 7.195 865.55

LAMA2 3.45 5.49 106 22.6

LAMA3 840 256 780.25 321.3

LAMA4 39.46 78.575 38.72 465.4

LAMA5 496 591 1190 156

LAMB1 13500 19300 10100 4480

LAMB2 1697.5 1806 4685 1148

LAMB2L 18 102 218 82.1

LAMB3 157 78.9 1240 19.7

LAMB4 3.695 5.925 5.5 4.46

LAMC1 3400 2090 12900 2000

LAMC2 82.025 168.4 6205 107.23

LAMC3 14.71 18.0966666666667 28.3666666666667 211.4

LAMP1 84100 26300 30500 22700

LAMP2 2250 1993.33333333333 803.333333333333 2573.33333333333

LAMP3 107 59 5.46 263

LANCL1 382 1030 1070 1220

LANCL2 51 14.3 4.48 47.6

LANCL3 1370 1500 1010 822

LAP3 6310 6200 3020 5010

LAPTM4A 28200 27900 61400 25500

LAPTM4B 16645 12190 14775 11900

LAPTM5 8.8 6.29 52.3 8.27

LARGE 1210 524 400 1290

LARP1 19400 24800 27400 28200

LARP1B 700 737.266666666667 490.666666666667 1187.33333333333

LARP4 427 582 494 551

LARP4B 1117.5 847 2101 1745

LARP6 556.454545454545 4195.45454545455 127.518181818182 2079.09090909091

LARP7 1935 2310 802 4790

LARS 2490 2460 4420 4050

LARS2 1360 2080 2330 4140

LAS1L 6240 16200 5340 18300

LASP1 11100 10200 13700 1670

LASS1 5580 2900 3460 9170

LASS2 15400 15600 6890 6180

LASS3 369.3 364.88 370.195 319.68

LASS4 837 285 30.3 248

LASS5 665 1650 2230 2680

LASS6 4070 3735 5850 3243

LAT 569 572 836 430

LAT2 3314.5 3450.2 3680.5 3867

LATS1 55.15 108.45 34.85 84.3

LATS2 327 840 442 294

LAX1 3.16 5.16 4.69 3.8

LAYN 4 17.5 5.37 1700

LBH 8.55083333333333 18.8391666666667 6.7475 59.8558333333333

LBP 3.36 5.35 8.78 9.9

LBR 29800 12500 13200 30000

LBX1 60800 68600 67500 70100

LBX2 35.615 38.15 80.85 73.95

LBXCOR1 2.72 4.39 5.29 14.5

LCA5 68 92.8 127 106

LCA5L 4.13 10.4 55.2 22.1

LCAT 76.7 184 80.2 36.7

LCE1A 14900 11800 8050 5910

LCE1B 11 42.2 30.3 14.2

LCE1C 4141.485 4227.415 2767.2 2192.855

LCE1D 86600 96000 65800 95500

LCE1E 162 1340 894 540

LCE1F 4.27 4.72 8.25 7.14

LCE2A 32 27 24.1 27.1

LCE2B 8.57 10.1 11.3 6.2

LCE2C 4.17 6.26 5.92 4.95

LCE2D 3.01 7.11 6.89 3.61

LCE3A 17.7 20 30.1 185

LCE3B 86.9 95.8 122 102

LCE3C 9.01 8.02 15.4 14.1

LCE3D 15.7 13.9 21.6 18.9

LCE3E 30.8 41.9 40.6 30.3

LCE4A 4.58 4.48 4.05 4.18

LCE5A 3.22 5.27 4.8 3.88

LCE6A 36.4 43.8 35.6 28.5

LCK 17.0218181818182 22.0136363636364 24.6372727272727 21.2454545454545

LCLAT1 766.6 1314 2913 2591

LCMT1 1663 1811 2814 2992

LCMT2 1581 797.1 6.867 1939

LCN1 6.86 6.48333333333333 5.35666666666667 29.34

LCN10 26.7 4.97 14 35.2

LCN12 12.1 18.5 12.5 17.6

LCN15 34.1 652 47.4 449

LCN2 3.83 6.02 7.16 4.56

LCN6 3.79 6.02 5.74 4.81

LCN8 7.25 6.04 5.63 3.41

LCN9 59.7 54.8 80.1 54.9

LCNL1 24.6 29.9 38.6 35.9

LCOR 437 711 169 397

LCORL 68.85 103.75 34.8 70.4

LCP1 137 3330 21.4 5710

LCP2 3.66 5.84 5.43 4.43

LCT 14.4 31.2 17.6 12.8

LCTL 343 1310 160 139

LDB1 133 177 160 173

LDB2 9.16 9.705 9.475 8.595

LDB3 13.585 15.835 10.355 18.8

LDHA 18450 34900 38200 41350

LDHAL6A 7250 7160 6550 6580

LDHAL6B 2.9 4.71 4.28 3.54

LDHB 274.5 1077.5 98650 113700

LDHC 101 162 154 1090

LDHD 26.9 25 660 74

LDLR 10500 10300 9700 2550

LDLRAD1 3.755 5.065 5.32 3.835

LDLRAD2 2.67 4.33 3.95 3.29

LDLRAD3 2.89 24.8 7.29 40.9

LDLRAP1 70.3 192 163 161

LDOC1 4780 8290 5360 7920

LDOC1L 1330 6.15 1000 2300

LEAP2 2270 633 855 342

LECT1 25.2 28.2 12.5 25.1

LECT2 72.85 7.528 5.125 5.205

LEF1 728 110 5.94 4510

LEFTY1 45.6 30.7 87 11.7

LEFTY2 3.63 5.78 5.37 4.37

LEKR1 78.45 15.98 5.18 26.8

LELP1 3.48 5.56 5.09 4.08

LEMD1 3.09 5.02 448 3.7

LEMD2 3080 2180 1910 2730

LEMD3 1530 1740 937 2540

LENEP 8.31 22.7 6.28 4.37

LENG1 205 180 275 228

LENG8 152.25 158.35 147.35 184.2

LENG9 270 105 109 304

LEO1 2535 1485 1467 1288.5

LEP 4.439 5.381 4.946 5.723

LEPR 9997.375 8649.04 9979.45 13295.975

LEPRE1 563.7 1527 1356 1163.5

LEPREL1 767 4450 7440 565

LEPREL2 690 2230 7150 9210

LEPROT 349 2520 1790 1300

LEPROTL1 2186.5 3384 2367.5 2631.5

LETM1 1446 1230.5 877 1823.5

LETM2 29.9 192 41.4 72.2

LETMD1 999.1 1051.6 1393 1608

LEUTX 5.79 6.23 5.95 5.11

LFNG 401.5 515 343.5 234

LGALS1 6650 11300 42900 515

LGALS12 21.2 23.5 25 39.5

LGALS13 3.35 5.4 4.96 4.06

LGALS14 3.57 5.68 5.33 4.21

LGALS16 3.08 4.97 4.57 3.78

LGALS2 14.93 969.2 748.6 7.108

LGALS3 4145 14280 1745 2820

LGALS3BP 2040 432 1450 415

LGALS4 25.9 48.9 73.2 30.9

LGALS7 75.5 286 138 82.5

LGALS8 1450 750 881 3.67

LGALS9 42.45 44.305 55.35 53.5

LGALS9C 83.6 130 150 264

LGI1 4.04 6.21 5.76 4.74

LGI2 7.095 238.5 4.93 101.45

LGI3 125 153 90 114

LGI4 47.1 11.5 25.8 23.4

LGMN 244 339 637 851

LGR4 7130 7190 6600 2140

LGR5 56.4 194 490 6.23

LGR6 376 502 300 472

LGSN 3.255 3660 4.83 3.955

LGTN 1320 1098 1055 2172

LHB 406 364 436 381

LHCGR 4.019 5.732 4.972 3.994

LHFP 8.88 32 54 2380

LHFPL1 6.47 5.065 7.06 10.245

LHFPL2 2080 2480 1710 756

LHFPL3 3.45 5.475 18.82 5.895

LHFPL4 457 37.4 57.3 58.1

LHFPL5 24.2 16.5 17.4 16.6

LHPP 780.5 2579 1017 1283

LHX1 11.1 377 20500 1150

LHX2 31.6 541 145 514

LHX3 3891.56 4642.455 3632.29 3831.875

LHX4 3.49 5.67 5.23 68.1

LHX5 2.69 7.66 6.81 13.6

LHX6 3.35 88.4 161 563

LHX8 2.92 4.72 4.28 414

LHX9 8.215 14.77 4.64 35.55

LIAS 1290 1020 362 460

LIF 162.2 131.15 552.55 56.35

LIFR 3.04 397 56.5 189

LIG1 4080 2570 9040 4200

LIG3 204 309 243 342

LIG4 45.6 30.6 46.9 12.7

LILRA1 104 72 69.6 61.6

LILRA2 3.76 5.88 5.67 4.74

LILRA3 7.29 5.35 8.99 8.34

LILRA4 19.9 14.5 16.3 15.2

LILRA5 3.57 4.25 3.85 3.23

LILRA6 2.79 4.54 4.11 3.4

LILRB1 3.64 5.68 5.5 4.6

LILRB2 2.57 4.17 3.78 3.17

LILRB3 32.3 9.72 65.1 47.95

LILRB4 18.105 12.36 18.17 11.79

LILRB5 3.03 4.69 4.46 9.27

LILRP2 11.6 5.87 8.36 23.1

LIM2 9.38 6.21 5.32 10

LIMA1 2300 3610 2690 4640

LIMCH1 748 694.5 133.85 231.75

LIMD1 1510 4600 2080 2920

LIMD2 71.4 49.4 19 35.4

LIME1 558 260 242 260

LIMK1 712 2220 2360 1080

LIMK2 2058 1126.5 706 879.5

LIMS1 2010 2560 2270 1830

LIMS2 36.445 319.95 195.425 176.45

LIMS3 11.155 20.33 14.85 8.7

LIMS3-LOC440895 7 8.01 9.11 12.8

LIN28 5.77 4.27 3.87 3.23

LIN28B 15700 30700 14.1 18500

LIN37 1540 1780 1540 1690

LIN52 181 430 226 379

LIN54 1100 1090 584 856

LIN7A 2480 2890 2530 423

LIN7B 549 1220 1040 1080

LIN9 1480 1077.5 1394 2954

LINGO1 333.2 106.35 9.265 461.5

LINGO2 4.23 6.41 26.2 5.1

LINGO3 2.74 6.28 26.9 30

LINGO4 331 248 401 126

LINS1 282 242 174 374

LIPA 2500 4280 6430 6960

LIPC 7050 3530 141 3.39

LIPE 296 168 4770 1460

LIPF 3.22 5.16 4.71 3.86

LIPG 2.67 318 1480 4.9

LIPH 52.9 131 381 11.7

LIPI 10.9 7.95 20.2 12.5

LIPJ 2.56 4.15 3.76 3.15

LIPK 3.18 5.06 4.64 3.81

LIPM 23.3 32.2 20.9 28.6

LIPN 92 84.2 54.2 118

LIPT1 321 681 462 656

LIPT2 109 394 926 1240

LITAF 525 530 3000 816

LIX1 5.8 4.74 4.33 3.55

LIX1L 299 439 786 735

LLGL1 4.24 6.37 6.06 5.11

LLGL2 6076 3188 10220 1258

LLPH 2680 3240 3800 4090

LMAN1 126 210 222 116

LMAN1L 68.3 70.6 58.1 74.8

LMAN2 5200 4830 6990 6290

LMAN2L 2380 2730 3830 1590

LMBR1 275.1 512.666666666667 808 753.333333333333

LMBR1L 182 430 128 500

LMBRD1 6810 6380 2440 2420

LMBRD2 73.4 36.5 74.6 30.5

LMCD1 1800 2250 1030 168

LMF1 67.6 48.2 74.8 76.2

LMF2 5261.5 8704.5 4901.5 6872

LMLN 3.07 12.6 18 14.8

LMNA 9440 13796.6666666667 7703.33333333333 2466.66666666667

LMNB1 1930 2560 3440 2560

LMNB2 3030 1360 2360 1800

LMO1 23.4 18.9 43.9 188

LMO2 3.271 5 74 265.7

LMO3 4.2 11.5 5.95 5.01

LMO4 1410 2230 6610 2680

LMO7 337.7 694.5 260.15 142.55

LMOD1 6.69 6.86 6.695 19.45

LMOD2 44.5 27.1 22.9 16

LMOD3 2.935 4.735 4.305 3.565

LMTK2 366 660 524 323

LMTK3 4.27 183 715 476

LMX1A 4.56 7.46 10.7 5.54

LMX1B 90.3 105.9 70.25 92.25

LNP1 181 466 108 266

LNPEP 363 517.5 501 395.5

LNX1 392 72.9 5.64 4.95

LNX2 1980 1180 1260 1360

LOC100008587 3890 11600 5770 5650

LOC100008588 14500 78500 17000 16300

LOC100008589 221050 225200 247575 230075

LOC100009676 120.6 174.15 333.5 303

LOC100034248 44.9 32.2 53.5 70.8

LOC100093698 2.73 4.43 4.02 3.33

LOC100124692 2.685 30.975 3.96 3.31

LOC100125556 83.4 114 178 90.8

LOC100126447 3.13 5.06 14.7 16.9

LOC100126584 3.5 5.61 5.2 4.23

LOC100126784 17.7 7.97 8.97 13.8

LOC100127904 129 132 106 103

LOC100127905 2.67 4.33 3.92 3.6

LOC100127909 3.65 9.54 66.9 72.3

LOC100127910 4.73 6.01 13.6 6.97

LOC100127913 46.1 45.6 33.4 28.6

LOC100127919 368 347 352 314

LOC100127920 2.66 4.31 3.9 3.57

LOC100127925 136 147 127 126

LOC100127930 4.23 6.41 6.09 5.13

LOC100127937 3.35 5.33 9.08 4.03

LOC100127940 3.09 5.02 4.57 3.77

LOC100127944 2.62 4.24 7.58 4.29

LOC100127946 18.4 17.8 26.4 19.7

LOC100127950 13.7 16.7 5.89 5.62

LOC100127953 75.8 70.1 131 59.7

LOC100127955 64 40 46.1 49.5

LOC100127961 3.47 4.19 7.09 48.1

LOC100127967 5.37 4.2 3.82 3.21

LOC100127972 3.02 4.75 4.43 3.63

LOC100127974 39.6 46.7 41.2 60.5

LOC100127980 944 244 612 471

LOC100127983 236 572 4.98 822

LOC100127984 3.26 5.27 4.86 3.99

LOC100127987 175 262 198 212

LOC100127988 13.2 4.81 4.4 3.58

LOC100127989 5.27 4.57 4.17 3.45

LOC100127991 3.42 5.56 5.1 4.18

LOC100127994 5.98 6.09 25 9.61

LOC100127997 19.6 14.8 24 28.4

LOC100127998 34.3 14 4.2 3.49

LOC100128001 14.6 13.7 4.16 22.1

LOC100128003 32.6 16.6 52.6 25.5

LOC100128006 12.7 55.6 5.68 18.1

LOC100128007 9.43 20.2 4.84 3.86

LOC100128009 23.7 33.3 19.6 36

LOC100128019 10.1 6.4 13.2 15.7

LOC100128025 25.1 24.6 71.7 32.2

LOC100128028 23 5.17 22.5 12.4

LOC100128031 270 292 317 352

LOC100128054 5.24 5 4.55 18.9

LOC100128055 18.2 8.41 6.53 31.6

LOC100128059 35.2 47.5 38.1 47

LOC100128063 3.19 4.61 4.22 3.49

LOC100128064 9.31 9.85 4.63 9.99

LOC100128067 47.1 61.05 61.45 58.85

LOC100128071 16.7 25.4 27 59.4

LOC100128074 115 73.6 32.4 44.5

LOC100128075 3.28 5.32 4.86 4

LOC100128077 10.225 10.34 7.965 5.375

LOC100128081 30.7 56.7 59.8 40.2

LOC100128084 263 231 227 290

LOC100128086 148 380 207 334

LOC100128088 12.6 6.46 11.7 13

LOC100128090 2.94 11.1 7.93 68.2

LOC100128093 12.5 9.86 5.51 15.3

LOC100128096 2.82 4.55 4.13 3.44

LOC100128098 6.66 260.5 4.94 4.075

LOC100128105 3.7 4.96 4.56 3.96

LOC100128107 9080 10400 6510 6760

LOC100128108 491 224 78.4 493

LOC100128126 35 52.2 27.9 55.9

LOC100128130 47.5 68.6 40.7 12.1

LOC100128131 17.6 10.8 514 18.1

LOC100128139 72.7 63 51.4 75.9

LOC100128142 291 237 283 252

LOC100128155 24.7 20.6 21.4 25.2

LOC100128160 3.42 5.51 5.05 4.14

LOC100128163 76.1 76 289 48.5

LOC100128164 15.6 8.41 11 13.1

LOC100128170 15.2 12.1 13.4 18.3

LOC100128172 4.18 4.14 5.12 5.74

LOC100128175 3.52 5.62 5.2 4.21

LOC100128176 14.7 4.43 10.7 3.36

LOC100128184 77.9 57.2 91.1 75.3

LOC100128186 3.54 5.73 5.32 4.33

LOC100128191 41.2 30 145 46.2

LOC100128198 11.4 19 20.7 15.8

LOC100128203 247 111 87.3 322

LOC100128219 41.9 43.6 51.8 29.9

LOC100128230 19.1 13.7 60.7 22

LOC100128233 19.4 25.1 16.6 21.7

LOC100128239 81.225 261.5 123.2 125.5

LOC100128242 3.35 5.39 70.8 175

LOC100128252 854.5 563 4.095 3.41

LOC100128253 178 213 157 210

LOC100128256 49.5 67.1 51.8 65.6

LOC100128262 8.49 7.24 7.71 11

LOC100128267 73.1 69.1 69 55.7

LOC100128276 30.9 28.1 32.2 27.2

LOC100128278 113 27.9 38.9 22.4

LOC100128281 21.1 12.7 20 22.3

LOC100128288 18.9 62 21.8 27.3

LOC100128292 376 566 58.1 378

LOC100128296 33.6 28.5 39.3 37.1

LOC100128300 22.2 24.5 4.59 13.4

LOC100128304 28.1 47.8 19.8 16.9

LOC100128317 3.44 5.55 5.14 4.19

LOC100128319 92.6 80.1 99.2 110

LOC100128320 42.7 37.3 63.4 70.2

LOC100128328 21.6 28.6 15 46.5

LOC100128332 10.7 11.2 21.5 21.6

LOC100128333 5.32 11.4 8.33 11.8

LOC100128334 4.11 6.25 7.22 6.33

LOC100128336 13.1 37.4 147 15.1

LOC100128342 62.3 65.2 62.5 62.1

LOC100128343 73 72 63.1 99.1

LOC100128348 2220.598 1904.52 1804.6 1766.98

LOC100128354 23.6 21.8 11.9 4

LOC100128356 3.6 5.68 5.43 4.51

LOC100128366 478 574 606 500

LOC100128370 84.6 122 230 170

LOC100128371 12.2 9.55 9.84 8.72

LOC100128372 1110 4340 8010 4150

LOC100128382 3.48 5.59 5.75 4.34

LOC100128386 30.4 22.6 23.6 13.8

LOC100128392 3.58 6.33 5.33 4.35

LOC100128398 214 56 54.6 40.8

LOC100128400 15.5 14.5 17.9 21

LOC100128401 19.7 11.2 4.85 12.8

LOC100128402 5.1575 10.79 13.55 13.925

LOC100128416 284 556 173 299

LOC100128430 3.61 4.775 4.38 4.755

LOC100128435 325 178 430 220

LOC100128437 3.64 5.89 5.44 4.46

LOC100128439 32.4 33.2 33.7 34

LOC100128440 105 157 162 103

LOC100128460 341.5 529 555 979

LOC100128469 362 231 180 246

LOC100128477 11.4 12 10.5 18.5

LOC100128482 4.13 5.49 5.08 5.05

LOC100128494 120 87.5 77.6 53.6

LOC100128496 3.19 5.06 5.835 5.075

LOC100128498 1020 695 550 740

LOC100128501 5.57 4.52 13 3.42

LOC100128507 4.26 6.27 6.01 5.2

LOC100128508 14.8 12.3 21.1 11.8

LOC100128510 761 712 438 739

LOC100128511 3.75 5.52 41.2 27.3

LOC100128517 7.18 8.1 16.8 17.7

LOC100128526 20.8 28.2 14.1 15.1

LOC100128528 128 108 106 105

LOC100128529 3.43 5.55 5.08 4.07

LOC100128531 57.1 19 5.08 4.47

LOC100128551 5.91 10 5.91 4.09

LOC100128556 3.6 5.84 5.33 4.22

LOC100128559 2.76 7.09 23.2 4.26

LOC100128562 470 510 343 280

LOC100128563 79.7 73 206 56.8

LOC100128567 570 287 42.9 196

LOC100128568 3 4.88 4.44 3.61

LOC100128571 9.98 7.51 10.9 3.92

LOC100128572 12.4 17.9 17 25.4

LOC100128573 2.95 12.2 4.35 3.56

LOC100128588 3.35 5.22 5.01 3.96

LOC100128591 14.5 10.9 9.2 10.4

LOC100128593 235 251 110 263

LOC100128597 11.7 11.5 5.83 6.39

LOC100128598 20 15 24.5 21.7

LOC100128607 3.5 5.69 6.76 4.28

LOC100128608 3.61 9.74 22.1 4.43

LOC100128613 4.54 4.875 4.44 4.785

LOC100128615 3.93 6.13 5.71 4.64

LOC100128619 3.54 5.72 5.33 4.31

LOC100128640 12 6.71 25.1 15.4

LOC100128651 3.42 5.51 5.11 4.13

LOC100128655 10.5 17.8 13.8 9.82

LOC100128657 4 6.19 5.77 4.71

LOC100128666 2.76 4.47 4.06 3.38

LOC100128668 57.4 58.5 3.9 9.13

LOC100128670 9.5 5.18 24.2 15

LOC100128672 4.8 4.81 4.37 3.63

LOC100128675 25.7533333333333 30.4 25.4666666666667 26.2

LOC100128682 48.93 25.965 4.62 5.775

LOC100128683 3.07 5.73 4.46 3.63

LOC100128688 4.06 11.5 5.79 4.78

LOC100128691 3.75 6.11 4.22 4.06

LOC100128695 7.47 68.9 60.1 3.39

LOC100128697 17 4.68 14 8.97

LOC100128699 3.01 4.89 4.45 3.66

LOC100128703 110.7 89.15 75.25 103.7

LOC100128714 1125.4 886.45 527.85 453.3

LOC100128717 3.3 5.3 4.89 4

LOC100128719 20800 39100 18500 16200

LOC100128726 5.86 4.32 9.47 42.3

LOC100128727 3.02 4.82 4.41 3.64

LOC100128728 5.22 5.8 9.64 7.07

LOC100128731 14000 18500 14300 21200

LOC100128737 246 377 790 901

LOC100128747 78.7 33.6 68.4 21.4

LOC100128749 3.86 11.4 19.2 38.6

LOC100128750 63 89.9 153 46

LOC100128751 3.03 4.91 4.46 3.7

LOC100128760 8920 9120 11500 9040

LOC100128787 3.84 6.07 5.81 4.9

LOC100128788 87.4 114 279 337

LOC100128792 6.82 4.69 9.92 8.63

LOC100128811 42.1 34.5 39.2 47.5

LOC100128813 3.21 5.22 8.37 3.89

LOC100128822 226 243 1680 1070

LOC100128823 8.12 6.04 13.3 8.2

LOC100128825 3.5 5.69 5.23 4.27

LOC100128830 7.77 12 11.6 10.1

LOC100128838 42.9 12.2 10 103

LOC100128841 4.49 5.4 5.73 4.26

LOC100128842 96.35 90.9 118.65 92.95

LOC100128843 71.2 60.3 74.5 75.2

LOC100128844 10.6 5.59 20.6 20

LOC100128851 2150 1460 1190 1230

LOC100128859 73.2 81.3 109 105

LOC100128869 32.6 58.3 40 31.7

LOC100128870 9.9 4.55 5.06 42.1

LOC100128881 240.45 13.315 1391 2486.5

LOC100128885 3.68 5.91 5.51 19

LOC100128889 2.61 4.23 3.83 3.2

LOC100128893 43.3 14.1 4.72 23.3

LOC100128895 48.1 50.4 58.4 269

LOC100128904 2.98 4.84 4.38 6.44

LOC100128905 3.72 5.9 5.63 4.68

LOC100128908 4.54 5.34 4.88 4.11

LOC100128909 17.1 15.9 17.2 19.9

LOC100128913 2.87 4.68 4.24 3.49

LOC100128916 4.62 4.6 5.43 39.5

LOC100128934 17.4 17.2 15.6 25

LOC100128935 5.91 4.57 10.4 6.27

LOC100128942 236.56 265.005 207.8 249.895

LOC100128943 39.9 14 7.31 13.2

LOC100128946 77.7 63.9 89.75 54.9

LOC100128949 24.5 22.5 20.2 39

LOC100128950 6.95 4.78 4.57 10.2

LOC100128960 2.99 11.9 6.23 8.76

LOC100128968 15.1 76 8.47 3.45

LOC100128975 2.96 4.71 4.32 3.57

LOC100128977 29.5 15.5 19 19.4

LOC100128979 3.28 5.35 4.86 3.94

LOC100128988 2.67 4.34 3.93 3.28

LOC100128993 6.19 27.7 11.8 10.3

LOC100128994 287 207.5 46.2 89.15

LOC100129000 2.94 4.69 4.29 3.55

LOC100129027 7.54 8.795 5.535 9.565

LOC100129029 5.06 8.145 55.85 5.39

LOC100129034 89.7 135 96.6 92.2

LOC100129035 40.4 37.4 43.6 34.8

LOC100129036 3.3 5.3 31.1 72.3

LOC100129042 3.9 6.11 5.72 4.67

LOC100129048 18.5 11.6 12.2 17.1

LOC100129053 3.54 5.67 5.26 4.3

LOC100129055 3.83 6.11 5.7 4.61

LOC100129058 15.1 11.4 17.7 8.17

LOC100129060 11.8 7.58 12.8 10.2

LOC100129069 48.3 42.5 42.8 49.3

LOC100129072 4.06 6.21 5.79 5.06

LOC100129081 25.1 33.7 17 23.8

LOC100129083 2.69 4.35 37.7 5.42

LOC100129085 1140 964 1140 2100

LOC100129089 2.67 4.33 3.93 3.28

LOC100129094 15.5 4.63 5.67 5.43

LOC100129098 3.74 5.82 5.64 4.75

LOC100129101 53.9 43.2 33.5 49.1

LOC100129103 30.7 25.2 51.6 14.8

LOC100129104 258 454 489 1090

LOC100129110 55.4 22.4 57 181

LOC100129111 26.7 31.9 34.4 38.4

LOC100129112 38.8 36.8 41 59.7

LOC100129113 52.5 63.5 254 73.2

LOC100129119 9.1 13.9 20.3 24.8

LOC100129122 12.6 7.77 102 98.2

LOC100129125 2.77 4.5 4.08 3.37

LOC100129129 195 255 197 388

LOC100129138 269 294 238 368

LOC100129144 3.13 5.11 4.65 3.77

LOC100129148 2.82 9.89 8.64 70.8

LOC100129149 289 241 310 257

LOC100129162 7.76 10.9 7.76 13.4

LOC100129166 34.2 32.7 26.4 34.5

LOC100129169 8.82 5.99 15.3 10.6

LOC100129171 5.72 11.6 11.9 8.42

LOC100129186 212.9 223.6 149.7 206.4

LOC100129193 3.38 5.48 5.06 33.9

LOC100129194 18.3 16 17.5 16.9

LOC100129195 53.8 18.7 22.5 123

LOC100129196 349.13 428.3 468 357.1

LOC100129198 7.89 6.68 5.19 3.51

LOC100129200 15.2 24.5 13.8 9.83

LOC100129203 8.64 6.3 5.93 13.5

LOC100129213 9.95 9.65 4.15 6.68

LOC100129214 3.66 5.85 5.38 4.32

LOC100129216 12.5 12.7 37.8 25.3

LOC100129218 13.1 5.96 16.4 5.21

LOC100129223 3.82 6.07 5.66 4.62

LOC100129231 3.57 5.7 5.27 4.26

LOC100129233 18.6 36.6 92.2 62.4

LOC100129235 37.7 19.1 26.2 30.6

LOC100129236 3.23 5 4.8 20.9

LOC100129238 27.2 21.9 21.3 21.6

LOC100129250 904 259 1300 1110

LOC100129258 3.23 5.26 6.39 3.88

LOC100129269 391 132 120 102

LOC100129275 19 26.4 19 93.8

LOC100129278 3.73 5.86 5.63 4.69

LOC100129280 45.2 98.9 30.9 25.2

LOC100129282 32.9 36 34.2 34.2

LOC100129291 13.2 13.2 20.1 20.8

LOC100129292 59.5 42 47 44.4

LOC100129295 24.8 19.1 27.7 27.9

LOC100129297 7.59 6.12 5.68 8.67

LOC100129303 3.12 5.09 4.63 5.88

LOC100129305 3.59 5.74 5.42 4.43

LOC100129311 3.23 5.25 4.8 35.2

LOC100129312 3.72 5.88 5.47 4.44

LOC100129316 4.02 6.21 5.81 4.76

LOC100129322 2.73 4.44 4.02 3.34

LOC100129323 3.49 5.62 5.16 81.8

LOC100129324 199 183 112 84.8

LOC100129354 3.52 5.72 23.4 5.7

LOC100129361 71.7 107 164 131

LOC100129362 959 1460 1030 476

LOC100129363 13.6 12.8 11.2 15.6

LOC100129365 35.8 47.1 46.5 42

LOC100129366 20.8 11.2 28.5 153

LOC100129373 7.54 8.53 12.7 29.9

LOC100129376 3.03 4.88 4.44 3.66

LOC100129380 27.5 33.4 35.4 49.7

LOC100129387 104 111 109 79.6

LOC100129390 3.25 5.26 4.82 3.89

LOC100129393 11.9 12.4 5.61 4.52

LOC100129395 13.8 9.66 9.87 10.6

LOC100129396 75.1666666666667 19.37 24.12 37.3233333333333

LOC100129397 3.87 6.1 6.69 6.83

LOC100129399 3.08 4.21 4.8 57.1

LOC100129406 386 452 321 376

LOC100129408 10.5 15.6 27.5 19.4

LOC100129411 4.06 5.91 10.7 14.7

LOC100129413 3.86 6.13 5.63 9.7

LOC100129417 5.29 16.9 10.1 4.59

LOC100129427 3.66 5.79 5.37 4.46

LOC100129436 23.7 26.4 37.3 27.9

LOC100129447 729 836 290 904

LOC100129453 4.1 5.02 4.58 3.73

LOC100129461 90.9 116 137 76.7

LOC100129463 4360 4290 2930 3090

LOC100129465 3.92 6.16 5.77 4.72

LOC100129466 3.2 5.2 4.74 3.9

LOC100129478 72.1 173 85.9 110

LOC100129480 2.88 4.68 4.25 3.51

LOC100129488 5.14 8.89 5.2 7.28

LOC100129498 2.66 4.31 3.9 3.27

LOC100129500 1030 610 270 203

LOC100129502 43.5 55.1 74.3 64.4

LOC100129509 24.4 27.4 24.5 27.8

LOC100129514 42.7 69.8 80.5 57.4

LOC100129515 7.16 4.18 6.65 9.07

LOC100129518 57.1 58.3 40.4 85.6

LOC100129520 3.47 5.6 5.16 4.26

LOC100129527 9190 13600 4810 9200

LOC100129531 37.7 35 52.2 50.4

LOC100129532 17.3 17.4 66.7 38.6

LOC100129534 58.3 24.6 67.5 99.6

LOC100129536 45 56.8 93.6 80.3

LOC100129538 3.37 5.37 4.98 4.06

LOC100129540 2.96 4.82 4.37 3.61

LOC100129550 158 548 398 489

LOC100129551 4.18 6.39 6.06 5.08

LOC100129555 18.6 49.5 86.8 78.7

LOC100129559 20.3 5.24 4.79 6.03

LOC100129571 33.5 54.2 41.6 37

LOC100129572 8 4.16 26.3 15.2

LOC100129578 7.77 9.79 12.4 20.3

LOC100129596 234 269 197 161

LOC100129617 17.1 12.3 4.26 16.5

LOC100129619 7.1 6.03 17.8 4.94

LOC100129620 10.6 5.89 14.5 5.71

LOC100129626 558 1350 657 644

LOC100129630 4.37 5.88 7.03 4.63

LOC100129633 8.41 8.51 17.1 18.9

LOC100129637 596.595 567.4 489.595 777.07

LOC100129648 34.5 35.2 35.8 35

LOC100129652 24.9 55.3 41.4 30.1

LOC100129654 6.48 4.44 4.03 87.1

LOC100129662 3.13 4.95 11 10.5

LOC100129675 38 53.3 42.2 18.7

LOC100129677 3.79 6.03 5.62 4.59

LOC100129697 3.94 6.21 5.81 4.76

LOC100129699 3.25 4.86 39 3.59

LOC100129700 13 36.1 16.7 11.7

LOC100129702 15200 16800 6420 18700

LOC100129707 16.5 12.5 5.6 4.6

LOC100129711 2.72 4.41 6.41 11.6

LOC100129716 93.05 84.52 88.29 100.395

LOC100129717 3.07 5.83 10.8 16.4

LOC100129721 6.88 13.2 4.86 9.75

LOC100129722 2890 2650 2860 3090

LOC100129724 3.09 73.8 4.54 3.69

LOC100129726 134 117 119 117

LOC100129733 26.7 31 68.3 46.1

LOC100129744 3.86 6.08 5.84 4.95

LOC100129763 27.3 43.1 37.6 34.6

LOC100129767 37.2 23.3 163 26.8

LOC100129773 6.97 26 15.3 10.4

LOC100129775 3.48 7.53 10.3 12.6

LOC100129778 3.39 5.5 31.4 4.13

LOC100129781 104 40.8 55.9 37.6

LOC100129785 6.96 5.29 11.4 5.19

LOC100129786 4.62 6.14 5.79 4.8

LOC100129791 10400 27400 9920 17000

LOC100129794 20.2 17.1 41.3 109

LOC100129797 3.28 5.34 4.87 3.9

LOC100129802 3.73 5.9 5.48 4.44

LOC100129826 65.1 65.4 79.9 52.1

LOC100129840 48.9 34.5 31 33

LOC100129841 6.12 4.11 3.73 3.13

LOC100129846 182 276 425 38

LOC100129850 37 18.8 12.9 4.56

LOC100129869 6.25 5.26 5.79 8.145

LOC100129878 27.7 59.3 26.5 10.7

LOC100129884 4.11 6.24 5.86 4.86

LOC100129887 4.12 6.22 5.92 5.02

LOC100129888 58.4 95.8 56.9 206

LOC100129890 2.83 4.54 55.7 9.17

LOC100129894 3.13 5.1 4.65 3.78

LOC100129897 409 187 195 281

LOC100129900 3.4 5.52 5.09 13

LOC100129906 14.9 4.44 6.06 8.33

LOC100129917 83.9 71.3 35.7 72.5

LOC100129931 27 92.6 16.6 19.3

LOC100129933 20.6 12.7 15.5 24.7

LOC100129935 34.7 5.25 4.8 3.88

LOC100129936 8.48 5.3 8.6 9.54

LOC100129938 4.21 6.28 6 5.14

LOC100129950 21.2 25.1 22.2 20

LOC100129954 2.92 4.75 4.31 3.57

LOC100129958 1120 2360 384 90.1

LOC100129961 19.1 10.4 5.77 10.9

LOC100129966 9290 4260 5840 6310

LOC100129969 2.81 4.56 4.13 3.42

LOC100129973 3.05 4.75 4.31 3.57

LOC100129974 315 318 342 309

LOC100129986 4.83 5.01 15.7 23.7

LOC100129988 10.8 6.06 43 15.1

LOC100130009 1320 1030 1850 698

LOC100130015 122 78.15 152.5 116.9

LOC100130017 20.3 11.5 28 10.3

LOC100130051 27.9 26.7 34.1 39.9

LOC100130054 5.18 8.22 5.25 4.24

LOC100130057 3.49 15.2 13.4 4.21

LOC100130065 10700 3790 2600 7940

LOC100130078 52.5 52.1 46.5 41.8

LOC100130079 2.79 4.54 4.11 3.4

LOC100130084 5.28 4.98 4.53 12.9

LOC100130093 66.5 37.7 306 93.6

LOC100130097 3.16 5.14 4.7 3.83

LOC100130098 14.7 25.9 23.9 9.96

LOC100130107 177.5 184 235.5 188.5

LOC100130111 2500 449 591 520

LOC100130116 3.88 6.22 5.83 10.4

LOC100130128 66.1 55.8 4.65 3.82

LOC100130131 47.65 103 57.8 102.3

LOC100130135 11.4 17 13.4 18.1

LOC100130138 2.59 4.18 13.8 8.91

LOC100130141 74.8 194.8 27.75 13.255

LOC100130148 5.19 4.6 4.75 3.44

LOC100130152 875 1220 904 998

LOC100130155 55.9 56.8 80.7 80.2

LOC100130157 2.75 4.47 4.05 3.37

LOC100130168 9.37 4.16 3.78 3.17

LOC100130169 2.64 4.28 3.88 3.25

LOC100130171 479 556 950 288

LOC100130172 2.8 4.55 7.55 3.42

LOC100130174 4.68 5.79 5.55 4.62

LOC100130175 459 500 413 627

LOC100130176 14.6 29.4 27.2 15.9

LOC100130178 571 489 371 331

LOC100130179 81.8 148 5.33 4.44

LOC100130193 11.5 10.9 40.3 17

LOC100130197 14.3 5.54 5.11 6

LOC100130207 3.32 5.36 4.92 4.03

LOC100130208 99.5 85.7 79 72.1

LOC100130218 10.4 11.7 22.1 40.4

LOC100130219 32.2 86 82.9 175

LOC100130231 4.38 4.77 4.35 3.61

LOC100130232 7.86 4.65 4.22 90.3

LOC100130238 856.45 962.345 447.77 911.5

LOC100130247 3.49 5.68 5.17 4.12

LOC100130248 3.41 5.48 5.04 4.06

LOC100130255 5.86 4.11 3.93 3.13

LOC100130256 5.6 5.1 4.85 4.96

LOC100130262 68.2 50.7 72.8 59.9

LOC100130263 209 518 394 268

LOC100130264 153 90.8 120 60.7

LOC100130274 127 116 131 152

LOC100130276 48 39.1 72 52.9

LOC100130278 3.82 10.3 7.16 10.1

LOC100130285 141 126 149 111

LOC100130288 2.95 4.8 12.9 3.57

LOC100130297 3.28 6.64 5.32 17.5

LOC100130298 3.23 5.24 4.77 3.93

LOC100130301 3.38 5.43 5 4.04

LOC100130314 2.78 4.49 4.08 3.4

LOC100130320 302 263 590 434

LOC100130325 6.04 19.8 9.88 6.88

LOC100130331 2.89 4.63 4.22 3.51

LOC100130342 14.1 20.1 24.8 24.8

LOC100130344 2.81 4.29 7.63 6.39

LOC100130345 85.4 69.1 84 79.7

LOC100130353 142 174 227 193

LOC100130354 3.72 5.76 5.61 4.75

LOC100130357 76.4 85.6 5.79 98.7

LOC100130363 110 19.3 41.2 35.1

LOC100130370 13 4.29 4.64 4.4

LOC100130372 9.05 6.83 10.6 6.66

LOC100130373 2.79 4.51 10.6 3.4

LOC100130383 78.9 153 43 45.6

LOC100130386 4.77 5.75 5.39 6.81

LOC100130387 63 55.7 63.7 96.2

LOC100130401 20.8 15 26.1 37.3

LOC100130413 66.3 70.7 26.9 438

LOC100130419 38 27.3 56.9 30.8

LOC100130429 11.9 5.55 12.7 12.8

LOC100130431 46.1 45.2 46.7 41.6

LOC100130433 32.2 33.8 17.6 62.4

LOC100130442 35.9 27.2 31.5 30

LOC100130445 6.1 7.27 8.125 6.81

LOC100130451 3.6 18.1 18.3 23

LOC100130452 3.55 5.71 6.8 13.1

LOC100130453 184 176 199 205

LOC100130454 4.36 4.4 4.02 4.67

LOC100130456 943 1060 944 940

LOC100130458 3.49 5.59 5.24 4.38

LOC100130460 47.1 38.9 38.3 43.9

LOC100130463 109 62.2 56.5 53.6

LOC100130465 4.13 5.41 5.05 16.8

LOC100130467 25.4 25.2 18.4 30.4

LOC100130468 2.55 4.13 3.74 3.14

LOC100130469 8.02 5.21 6.92 7.23

LOC100130472 10.8 8.9 13.8 10.5

LOC100130476 135 122 135 132

LOC100130480 73.9 4.18 3.8 9.68

LOC100130494 315 346 269 316

LOC100130502 427 20.7 44 17.5

LOC100130503 2.9 4.65 4.26 3.53

LOC100130506 319 1160 2200 602

LOC100130520 12.8 7.2 4.19 3.45

LOC100130522 134.75 128.2 97.05 128.1

LOC100130533 1770 1830 1650 1750

LOC100130539 73.6 84.7 71.1 76.2

LOC100130540 30.3 29.8 25 32.1

LOC100130542 3.9 6.09 8.48 6.7

LOC100130547 45.385 27.34 43.935 38.19

LOC100130557 836.2 1142.65 828 1095

LOC100130560 245 232 170 206

LOC100130563 3.44 5.48 5.16 7.81

LOC100130573 3.5 5.7 5.25 4.25

LOC100130580 10.3 4.8 5.84 26.2

LOC100130587 30.4766666666667 26.99 30.1333333333333 23.6766666666667

LOC100130589 3.32 5.31 4.87 3.99

LOC100130597 6.65 4.93 7.1 3.65

LOC100130600 238 186 273 237

LOC100130616 3.22 5.26 4.8 3.88

LOC100130627 3.39 5.41 5.02 4.09

LOC100130633 888 1170 2070 1210

LOC100130642 3.58 5.08 6.51 9.1

LOC100130644 3.57 5.79 7 4.95

LOC100130652 26 21 47.9 41.4

LOC100130654 2370 2420 3130 1950

LOC100130657 2.83 4.6 6.42 3.43

LOC100130663 32.3 34.4 40.8 37.5

LOC100130667 3.44 6.58 5.12 4.2

LOC100130673 90.2 272 59.6 213

LOC100130692 31 12.8 5.25 15.4

LOC100130700 12.5 27.3 127 54.9

LOC100130701 12 7.54 9.43 9.25

LOC100130705 22.3 19.2 27.9 18.5

LOC100130710 138.3 150.95 187.6 162.9

LOC100130711 70.2 1610 769 603

LOC100130713 12 16.4 22.6 13.3

LOC100130721 593 505 444 426

LOC100130729 22.9 27.4 32.5 24.1

LOC100130736 7.49 5.72 5.9 4.34

LOC100130741 29 24.3 44.1 38.9

LOC100130745 32.9 41.3 38.9 42

LOC100130761 5.65 7.96 9.28 6.56

LOC100130764 10.2 8.06 18.7 6.72

LOC100130768 28.3 29.5 30.8 35.2

LOC100130778 3.2 5.16 4.76 3.91

LOC100130794 11500 9490 6770 11300

LOC100130797 9.15 6.98 5.67 4.57

LOC100130798 2.78 4.13 3.74 3.14

LOC100130800 9.1 7.61 8.37 8.39

LOC100130803 3.43 6.02 5.12 4.19

LOC100130811 13.4 7.66 21 13.7

LOC100130814 11.3 11.1 16 16.5

LOC100130817 4.76 5.68 5.26 4.27

LOC100130819 175 182 158 246

LOC100130825 598.65 537.2 365.6 563.45

LOC100130827 63.1 30.8 44.9 24.8

LOC100130828 19.1 19 11.2 25.9

LOC100130837 8.81 6.18 40.4 5.63

LOC100130840 3.4 5.39 5.05 37.4

LOC100130846 3.7 5.87 5.58 4.6

LOC100130849 2.65 4.3 7.2 4.36

LOC100130850 2.88 5.09 4.27 3.55

LOC100130852 17 8.41 6.95 13.1

LOC100130855 2.75 4.47 4.06 3.36

LOC100130857 3.68 5.71 5.57 4.84

LOC100130859 6.7 29.8 5.71 4.68

LOC100130865 21.5 20.3 13.8 24.6

LOC100130872 63.8 63.1 81.4 63

LOC100130880 3.57 5.78 5.33 4.36

LOC100130882 18.7 21.2 4.47 6.73

LOC100130883 3.41 5.55 5.08 4.17

LOC100130890 639 630 2080 1150

LOC100130897 33 30.6 41.2 34.1

LOC100130899 3.07 4.98 4.53 3.75

LOC100130902 48.9 67.5 65.1 55.9

LOC100130905 76.6 136 73.9 146

LOC100130906 76.3 90.3 81.5 39.5

LOC100130913 30.6 26.3 17.5 33.8

LOC100130920 29.2 30.2 43.4 53.6

LOC100130921 72.6 6.39 53.6 167

LOC100130924 4.23 8.63 10.1 7.8

LOC100130927 58.2 29.4 22.8 23.6

LOC100130930 233 78.3 74.1 152

LOC100130931 3.76 5.95 5.67 7.22

LOC100130932 67900 88350 59400 64100

LOC100130935 1140 762 37.2 5130

LOC100130938 9.73 6.2 19.1 14.1

LOC100130943 16 11.7 559 127

LOC100130950 23.1 25.8 30.4 39.4

LOC100130951 2.79 4.5 118 3.4

LOC100130954 2.91 4.86 4.29 4.08

LOC100130966 2300 2660 4040 3650

LOC100130967 74.1 155 37.9 60.1

LOC100130982 58.7 61.7 57.3 67

LOC100130987 32.7 35 31.3 34.9

LOC100130996 109 59.3 33.6 84.3

LOC100130998 3.53 5.64 13.5 7.48

LOC100131000 32.2 9.08 92.5 156

LOC100131001 7.4 11.8 5.64 6.51

LOC100131009 46.7 44.8 55.3 44.8

LOC100131015 70.1 56.1 37.6 38

LOC100131023 2.57 4.15 3.77 7

LOC100131031 2.87 4.63 4.21 3.5

LOC100131032 5.13 6.38 6.14 4.76

LOC100131034 5.8 4.65 4.27 3.55

LOC100131043 15.6 4.72 5.43 3.61

LOC100131046 173 198 9.09 90.3

LOC100131048 14.235 33.285 19.05 26.1

LOC100131053 11 16.4 7.75 11.2

LOC100131060 9.56 16.6 14.7 13.1

LOC100131067 67 23.9 31.5 33.9

LOC100131068 3.73 5.97 5.52 6.5

LOC100131089 22.9 89.55 17.765 58.8

LOC100131098 3.77 6.04 7.4 25.7

LOC100131099 23.2 26 31.9 36.4

LOC100131101 7.85 7.93 6.58 8.13

LOC100131102 4.89 6.55 5.295 9.22

LOC100131106 11.1 11.3 11.9 10.9

LOC100131107 3.785 5.94 5.57 4.58

LOC100131109 13.3 10.1 7.1 13.2

LOC100131112 3.69 5.95 5.43 4.32

LOC100131129 3.38 5.43 4.99 4.03

LOC100131138 32.2 345 317 194

LOC100131149 85.7 67.9 28.6 97.5

LOC100131150 131 146 126 112

LOC100131154 43.85 55.15 55.35 46.9

LOC100131165 41.2 24.85 45.85 103.4

LOC100131170 13.2 4.79 4.7 9.09

LOC100131172 3.61 5.71 5.44 4.49

LOC100131176 14.7 17.3 4.93 3.99

LOC100131180 5.71 5.01 4.54 7.6

LOC100131195 9.08 11.6 27 23.2

LOC100131199 10.7 22.5 46.8 125

LOC100131203 143 4.68 4.43 3.57

LOC100131209 32.9 88.7 21.8 29

LOC100131225 4.45 4.33 14.2 12.2

LOC100131231 6.66 27.2 15.8 24.7

LOC100131232 8.7 6.22 10.1 10.6

LOC100131234 158 78.8 21.6 39.1

LOC100131242 20.6 20.8 25.7 37.3

LOC100131244 46.6 29.2 41.1 33

LOC100131251 26.9 39.1 25.3 46.7

LOC100131254 6.08 4.73 11.8 50.4

LOC100131257 53.4 105 49 87.1

LOC100131262 3640 1060 11100 6510

LOC100131271 31.7 5.22 4.06 44.2

LOC100131276 50.8 41 48.1 27.1

LOC100131283 2.83 4.59 5.12 4.16

LOC100131289 6.79 6.32 5.92 4.85

LOC100131297 2.62 4.24 3.85 3.22

LOC100131298 6.25 5.99 5.47 15.5

LOC100131303 3.14 4.46 4.06 3.38

LOC100131311 4.84 7.1 11.1 9.77

LOC100131315 17.8 17.2 17.1 22.6

LOC100131316 14.7 17.9 48.2 33

LOC100131320 16.48 4.98 24.775 36.54

LOC100131326 124 135 277 104

LOC100131342 39.4 37.3 42.5 32.9

LOC100131346 15.7 8.57 10.3 8.24

LOC100131354 2.54 16.9 194 28.6

LOC100131355 16.9 20.8 36.7 75.1

LOC100131366 16.2 11.9 13.9 14.1

LOC100131368 13 10.2 13.4 13.9

LOC100131372 23.3 8.43 8.53 8.42

LOC100131381 39.4 38.5 36.6 37.8

LOC100131391 6.63 5.16 86.1 3.38

LOC100131397 13.5 34.1 18.3 23.7

LOC100131403 11.4 13.8 30.1 46.4

LOC100131409 9.35 8.8 12 9.38

LOC100131414 18.275 21.63 30.05 20.92

LOC100131416 99.5 49.6 36.6 86

LOC100131426 12.5 14.4 9.84 4.28

LOC100131431 2.89 4.71 4.27 3.51

LOC100131432 180 192 123 153

LOC100131434 7.66 12.385 13.955 24.95

LOC100131435 15.2 9.98 24.4 9.91

LOC100131436 9.9 10.7 26.8 9.48

LOC100131473 1410 1490 1450 1210

LOC100131479 42.4 44.9 22.4 15.1

LOC100131482 10300 9530 17400 11600

LOC100131490 54 101 215 70.8

LOC100131496 4.11 5.35 7.04 3.98

LOC100131500 11.5 14.7 5.25 4.25

LOC100131510 10.3 5.35 30.4 12.6

LOC100131512 252 81 302 17.9

LOC100131514 14.405 8.6 17.595 7.495

LOC100131519 28.3 21.1 20.8 16

LOC100131525 26.9 25.7 33.2 26.4

LOC100131531 3970 7450 5510 6680

LOC100131532 2.66 4.32 3.91 3.27

LOC100131539 3.74 6.02 5.5 4.38

LOC100131540 2.93 4.78 4.33 3.55

LOC100131542 11.5 4.68 13.4 4.97

LOC100131544 98.7 99.6 118 97.5

LOC100131546 28.2 37.1 34.4 39.2

LOC100131551 596.7 542.485 541.05 394.835

LOC100131552 11.8 4.3 3.91 3.28

LOC100131554 47.1 53.3 43.7 50.1

LOC100131573 18.8 35.7 34.8 54.2

LOC100131580 2.8 4.52 4.11 3.42

LOC100131581 253.672857142857 430.77 156.33 293.651428571429

LOC100131582 9.18 10.8 159 27.5

LOC100131583 11.4 25.1 18.4 18

LOC100131589 20.5 16.5 22.2 14.9

LOC100131591 6.42 6.36 7.83 5.13

LOC100131594 43.2 48.9 7.09 17.4

LOC100131598 3.69 5.87 5.42 4.37

LOC100131599 7.65 17.3 5.67 4.48

LOC100131601 410 363 513 204

LOC100131608 3.25 5.3 4.83 5.34

LOC100131630 11.6 5.94 18.9 15.7

LOC100131635 7.56 9.59 9.92 3.62

LOC100131639 3.69 5.93 5.55 4.55

LOC100131642 18.1 32.7 8.73 24.2

LOC100131646 92.5 98.4 71.3 102

LOC100131650 12.1 9.21 12.8 12.9

LOC100131653 6.41 9.17 10.7 3.8

LOC100131654 7.91 6.37 7.69 11.3

LOC100131657 11.5 19.6 12.3 18.9

LOC100131662 2.76 4.49 4.06 3.38

LOC100131669 3.66 5.83 5.51 4.51

LOC100131673 3.34 5.46 4.97 3.96

LOC100131683 8.19 14.3 5.8 110

LOC100131686 87.0733333333333 99.8666666666667 82.8566666666667 90.28

LOC100131688 7.5 28.8 47.1 22.5

LOC100131691 5.49 4.81 11.4 5.07

LOC100131700 84.2 79.5 76.2 28.1

LOC100131702 3.45 34 16.2 23.8

LOC100131705 16 6.62 17.7 18.3

LOC100131706 76.3 49.2 71.8 88.9

LOC100131719 2.62 4.24 3.85 3.23

LOC100131726 3.15 5.14 8.71 5.89

LOC100131727 22.1 18.4 13.4 52

LOC100131733 24.4 10.1 25.4 14.7

LOC100131738 6.65 6 73.6 16.2

LOC100131742 15 10.7 13.1 11.7

LOC100131744 11 10.8 41 31.3

LOC100131746 16.2 24.1 14.5 15.2

LOC100131756 9 4.44 4.04 3.38

LOC100131763 3.5 5.6 5.18 4.23

LOC100131774 18.9 5.99 5.55 4.54

LOC100131778 80.9 95.1 72.4 93.2

LOC100131785 1690 1840 1110 1150

LOC100131802 3.57 5.74 5.32 4.33

LOC100131811 21 12.4 14 18.7

LOC100131818 16.2 4.76 9.8 7.42

LOC100131820 2.92 4.75 10.7 3.58

LOC100131821 10.4 14 13.2 13.2

LOC100131822 3.18 5.33 5.53 3.83

LOC100131825 3.38 4.35 3.94 10.9

LOC100131829 106 226 74.2 143

LOC100131830 297 348 277 292

LOC100131831 135.6 104.65 81.75 72.7

LOC100131836 3.24 5.27 4.81 3.95

LOC100131840 2.81 4.57 4.14 3.42

LOC100131850 23.9 8.27 30 22.725

LOC100131857 31.3 22.2 25 25.5

LOC100131860 27.9 20.1 67.3 34.6

LOC100131864 35.3 23.7 21 27.3

LOC100131869 12.7 18.7 8.25 16.9

LOC100131880 2.82 4.55 5.18 3.44

LOC100131891 3.3 5.36 4.89 4.02

LOC100131892 16 5.28 8.9 12.3

LOC100131894 7.19 6.83 18.5 20.5

LOC100131907 16.6 10.4 37.3 17.2

LOC100131909 27.5 27.8 32.1 41.7

LOC100131910 10.6 18.9 26.4 21.7

LOC100131929 43.2 48.7 46.7 49

LOC100131938 13.75 8.045 10.655 9.435

LOC100131941 11.3 12.9 20.2 17

LOC100131960 17.76 19.0733333333333 35.4 39.31

LOC100131968 65.8 71.5 59.1 73.7

LOC100131988 17.9 17.5 49.4 31.3

LOC100131993 17.2 19.7 21.3 15.9

LOC100131998 10.6 6.41 14 4

LOC100132005 5.57 5.07 4.74 3.86

LOC100132006 185 166 100 138

LOC100132014 4.18 4.52 7.13 5.59

LOC100132015 4.97 6.99 13.1 4.5

LOC100132051 47.3 34.6 6.56 3.39

LOC100132069 3.72 5.89 8.7 4.43

LOC100132071 4.2 4.96 13 3.68

LOC100132077 79.5 55.9 36.9 108

LOC100132078 3.78 5.83 6.71 4.97

LOC100132101 99.25 85.65 101.35 92.15

LOC100132107 3.62 5.76 5.34 4.33

LOC100132108 2460 3810 2960 3730

LOC100132111 5.97 12.3 7.76 4.28

LOC100132116 54.88 68.525 61.325 60.825

LOC100132146 3.4 16.7 4.87 7.53

LOC100132147 3.72 5.97 5.52 5.63

LOC100132159 6.595 9.67 8.23 7.925

LOC100132161 34.2 27.3 27.1 23.4

LOC100132168 552 464 435 512

LOC100132188 24.7 22.9 38.4 29.3

LOC100132197 81.1 58.2 68.7 65.6

LOC100132207 128 123 120 134

LOC100132215 20.1 16.1 6.34 4.36

LOC100132217 281 327.5 262.5 349.5

LOC100132234 10.6 37.9 7.25 12.5

LOC100132240 8.63 5.56 14.9 23.1

LOC100132244 38.1 34.9 43.7 42.4

LOC100132247 69100 63200 80050 75050

LOC100132261 242 301 273 304

LOC100132273 61.1 53.6 150.6 273.5

LOC100132288 116.2 37.2 168.55 130.5

LOC100132296 350 317 223 220

LOC100132301 29 31.1 27.5 46.7

LOC100132314 811 444 609 1200

LOC100132330 3.39 5.99 7.07 3.77

LOC100132336 2.6 4.21 3.81 3.19

LOC100132338 793 629 1060 735

LOC100132344 15.1 13.6 16.6 15.6

LOC100132345 6 210 4.1 94.2

LOC100132354 2.64 4.27 3.88 3.25

LOC100132363 110 134 105 118

LOC100132364 66.7 113 157 133

LOC100132368 52.7 73.2 49.8 68.4

LOC100132369 13 11 14.7 12.5

LOC100132388 18.8 14.4 20.5 22.7

LOC100132413 130 122 136 129

LOC100132423 3.08 4.98 4.52 52.4

LOC100132428 8.54 18.5 17.8 21.8

LOC100132433 238 325 289 310

LOC100132469 3.79 9.83 3.86 7.02

LOC100132474 57.4 10.2 6.02 16.7

LOC100132481 65.2 60.1 74.2 97.9

LOC100132483 40.4 41 43.1 47.2

LOC100132495 10.2 14.1 39.5 49.2

LOC100132501 15.2 10.4 15.1 17

LOC100132509 313 160 165 118

LOC100132514 12.9 11.3 13 9.62

LOC100132515 26.3 25.8 29.7 24.1

LOC100132526 3.77 6.04 5.53 8.62

LOC100132529 114 144 91.7 90.9

LOC100132541 342 651 437 406

LOC100132562 49.8 43.8 40.5 64.5

LOC100132588 65.1 72 9.75666666666667 19.8

LOC100132593 19.6 19.4 38.4 15.2

LOC100132598 2.99 6.07 30.5 3.62

LOC100132612 4.21 5.82 5.59 4.64

LOC100132614 512 352 810 698

LOC100132622 27.2 20.8 15.8 10.7

LOC100132625 12.1 9.02 14.3 22.9

LOC100132630 10.6 8.72 21.9 16.8

LOC100132649 10.4 18.1 23.2 28.7

LOC100132652 79.1 42.8 18.6 108

LOC100132653 16.5 5.82 11 10.2

LOC100132658 63.6 73.5 54.5 83.2

LOC100132663 6.79 5.75 5.39 4.35

LOC100132672 184 364.5 235 219

LOC100132701 12.9 4.7 15 6.51

LOC100132707 148 106 194 204

LOC100132713 3.38 5.41 5.04 4

LOC100132716 70.25 66.75 63.3 61.35

LOC100132724 2680 2630 3500 2840

LOC100132733 117 127 80.3 362

LOC100132735 16.2 17.7 12.5 14.4

LOC100132738 16.6 11.4 11 8.64

LOC100132764 6.18 16.5 14.3 6.45

LOC100132767 185 172 216 134

LOC100132774 52.1 184 394 229

LOC100132779 2.55 4.13 3.74 3.14

LOC100132781 6.48 5.93 5.73 4.92

LOC100132782 5.16 4.72 6.04 3.6

LOC100132787 44.4 56.9 64.9 42

LOC100132790 200 254 320 205

LOC100132799 4.26 6.96 10.2666666666667 29.8266666666667

LOC100132800 7.54 5.84 5.42 4.42

LOC100132805 115 55.4 28.1 31.4

LOC100132813 2.99 4.83 4.4 3.65

LOC100132815 58.9 73.8 49.1 37.4

LOC100132816 486.98 474.98 177.315 361.7

LOC100132826 4.34 4.23 6.57 3.21

LOC100132829 17 6.21 33.5 8.15

LOC100132831 790 997 1010 735

LOC100132832 122 114 90.3 155

LOC100132839 3.16 4.35 8.67 7.23

LOC100132848 706 795 576 649

LOC100132850 2.63 4.25 3.87 3.25

LOC100132855 19.7 25.6 23.2 35.8

LOC100132857 59400 60400 51700 62800

LOC100132859 15.6 11.5 38.4 74.9

LOC100132870 32.2 25.9 24.3 36.6

LOC100132878 3.34 9.02 67.9 3.43

LOC100132883 27.1 50.6 38.8 67.4

LOC100132887 497 483 475 492

LOC100132891 3.33 5.37 4.96 11.8

LOC100132893 11.9 12.2 8.66 8.83

LOC100132913 4.32 4.37 5.62 4.15

LOC100132919 33.9 4.66 5.3 3.55

LOC100132944 778 807 565 841

LOC100132952 13.2 5.51 27.9 28.8

LOC100132955 20.455 16.58 57.96 57.975

LOC100132966 1471.675 1762.65 1389.35 1404.075

LOC100132972 35.6 80.9 91.6 36.9

LOC100132973 9780 9470 10900 8900

LOC100132977 5.98 16 11.6 10.7

LOC100132983 2.71 4.39 3.98 3.32

LOC100132984 2560 2710 2440 5750

LOC100132987 3.24 5.29 4.81 6.52

LOC100133001 98.3 69.3 130 88.7

LOC100133008 32.2 1130 831 324

LOC100133019 2.94 4.67 12.8 3.57

LOC100133029 78.5 62.7 69.8 52.1

LOC100133039 13.9 4.28 23.3 4.1

LOC100133047 37.7 34.7 40.7 31.3

LOC100133050 586.5 802.5 404.5 605.65

LOC100133058 2.7 4.37 66 3.3

LOC100133065 3.09 4.33 4.69 5

LOC100133070 888 997 699 885

LOC100133075 99.06 94.1 99.98 103.87

LOC100133083 28.2 6.38 35.9 5.04

LOC100133086 82.8233333333333 98.3233333333333 66.0766666666667 62.6433333333333

LOC100133089 85.8 89.6 81.4 145

LOC100133091 299 409 422 510

LOC100133109 90.1 87.8 137 98.6

LOC100133116 19.5 20.5 29.9 25.8

LOC100133130 28.1 131 43.1 30.4

LOC100133142 38.1 63 108 22.7

LOC100133144 97.9 107 123 235

LOC100133145 3.65 5.8 5.5 4.51

LOC100133153 11.9 4.63 4.27 3.54

LOC100133161 36388.75 38132.5 24575 41650

LOC100133165 3.85 6.02 5.82 4.95

LOC100133173 3.89 6.09 5.67 4.6

LOC100133176 57.6 56.6 47.8 18.6

LOC100133180 95.5 180 67.5 150

LOC100133185 6.66 8.55 5 4.05

LOC100133190 11 4.86 33.8 5.41

LOC100133214 2080 1940 1150 788

LOC100133219 2.55 4.14 3.75 3.15

LOC100133224 3010 1570 1090 1260

LOC100133226 2.68 4.33 3.95 3.31

LOC100133227 23.5 62.2 54.1 26.6

LOC100133263 519 306 333 32.9

LOC100133264 18.7 43.5 69.8 95.7

LOC100133270 37.6 4.54 49 3.4

LOC100133280 49.17 42.99 56.8233333333333 48.29

LOC100133286 120 135 118 131

LOC100133287 24 25 32.5 25

LOC100133301 351 344 316 411

LOC100133306 3.38 5.39 4.97 4.1

LOC100133308 3.25 5.24 4.83 3.96

LOC100133311 4.89 5.535 19.4 57.15

LOC100133315 9.095 90.9 81.65 100.9

LOC100133319 27.3 26.9 23.7 93.3

LOC100133322 9.98 90.6 8.67 3.45

LOC100133331 311.25 384.75 424.75 530.25

LOC100133337 479 512 86.4 487

LOC100133402 51.1 45.7 38.5 61.8

LOC100133405 14.1 14.6 8.2 7.32

LOC100133408 15.9 18.7 32.7 20

LOC100133450 7.51 7.37 5.12 11.4

LOC100133461 3.54 5.73 7.21 4.32

LOC100133469 4.08 6.23 5.85 4.82

LOC100133478 2190 1350 786 1130

LOC100133479 53.6 51.1 23.4 51.4

LOC100133487 182 95.4 551 427

LOC100133500 5.9 11.9 7.99 15.2

LOC100133553 2.76 6.89 12 3.36

LOC100133554 9.83 10.8 26.3 17.6

LOC100133580 5.76 4.87 6.62 3.7

LOC100133588 48.8 54.2 68.2 59.2

LOC100133598 16.9 9.99 11.2 10.4

LOC100133599 2.82 4.57 5.36 3.43

LOC100133612 18.3 9.88 6.5 7.2

LOC100133636 3.6 5.81 5.31 4.23

LOC100133638 347 443 283 392

LOC100133641 3.43 5.49 5.07 4.23

LOC100133654 14.2 30.8 21.9 24.1

LOC100133655 68.8 70.3 65 81.9

LOC100133659 249 268 223 256

LOC100133660 280 335 227 243

LOC100133661 3.65 5.88 5.43 4.3

LOC100133683 3.01 4.85 4.43 3.67

LOC100133690 22 5.37 16.4 4.61

LOC100133716 4.05 6.36 5.97 10.5

LOC100133728 6.23 41.8 5.78 4.77

LOC100133732 3.59 5.83 5.31 6.08

LOC100133737 4075 4525 8635 4285

LOC100133741 2.74 4.44 4.03 6.36

LOC100133756 5.68 4.52 6.675 4.725

LOC100133758 36.4 24.2 14.7 38.3

LOC100133790 20.4 25 24.2 18

LOC100133791 18.6 20.9 80.8 11.7

LOC100133817 12.2 18.3 8.97 15.2

LOC100133839 14.2 15.2 9.18 5.06

LOC100133844 6.06 6.14 13.6 11.6

LOC100133857 9.1 9.49 11.9 16.6

LOC100133862 4.28 15.8 9.33 3.31

LOC100133863 2.63 8.21 4.04 3.23

LOC100133881 2.74 4.46 4.04 3.35

LOC100133882 10.7 4.7 6.58 3.51

LOC100133885 14.1 12.9 33 21.8

LOC100133889 2.81 58.8 8.9 362

LOC100133890 2.94 4.69 4.33 3.55

LOC100133893 7.45 8.91 5.395 8.795

LOC100133899 10.9 15.9 19 25.1

LOC100133910 3.4 18.3 5.03 7.5

LOC100133915 14 10.2 13.7 10.4

LOC100133920 12.4 10 62.9 10.4

LOC100133923 577 913 1550 2170

LOC100133928 16.7 24 9.09 21.8

LOC100133949 3.06 9.58 4.55 3.76

LOC100133957 2.99 4.74 13.4 7.98

LOC100133959 2.73 4.43 4.02 3.35

LOC100133975 3.54 5.74 5.29 4.34

LOC100133979 10.6 11.6 8.82 16.6

LOC100133981 173 139 106 145

LOC100133985 336 313 263 202

LOC100133990 274 296 221 337

LOC100133991 33.9 35.9 55.6 40.1

LOC100133994 14800 16100 7480 15400

LOC100133998 39.1 39.6 40.7 42.4

LOC100134002 483 491 583 675

LOC100134040 5.71 16.76 13.62 23.77

LOC100134041 4.72 4.76 10.1 3.54

LOC100134060 2.66 4.32 3.91 4.61

LOC100134067 9.32 5.85 5.44 4.43

LOC100134071 2.94 4.79 4.33 3.55

LOC100134101 3.57 5.79 5.33 4.37

LOC100134102 116 172 142 200

LOC100134123 2.59 13.4 4.64 3.2

LOC100134139 3.89 6.16 5.64 4.9

LOC100134147 23.5 20.2 21.5 43.3

LOC100134152 9.75 7.26 6.98 8.97

LOC100134160 3.07 4.96 4.56 3.77

LOC100134162 26.8 37.3 27.8 37.3

LOC100134170 8.13 15.2 13.1 9.89

LOC100134174 6.56 6.57 8.51 20.7

LOC100134189 4.07 6.22 5.8 4.79

LOC100134228 1010 862 531 1310

LOC100134229 3.1 22.1 23.8 64.8

LOC100134230 5.57 6.12 6.83 15.8

LOC100134235 6240 8130 11600 6490

LOC100134237 140 76.7 175 134

LOC100134240 5.58 5.58 11.285 26.445

LOC100134244 3.2 6.02 10 3.87

LOC100134253 766 711 765 704

LOC100134259 147 34.5 36.6 31

LOC100134261 15.8 5.78 5.45 4.45

LOC100134269 60.9 15.3 40.3 16.9

LOC100134274 3.06 4.9 4.53 3.73

LOC100134279 2.65 4.28 3.9 54.2

LOC100134292 81.6 118 155 168

LOC100134346 12 9.32 20.1 13.3

LOC100134353 7.9 7.94 8.09 5.49

LOC100134359 7.66 8.14 8.24 7.19

LOC100134360 310 394 314 374

LOC100134361 2490 219 768 2520

LOC100134365 42.3 41.7 166 53.4

LOC100134368 6.18 22.9 5.9 15.1

LOC100134372 8.49 10.7 6.76 4.64

LOC100134381 17.9 14.2 29 13.6

LOC100134387 143 162 308 567

LOC100134395 3.1 5.05 4.59 3.71

LOC100134403 22.2 78.1 18.3 20.8

LOC100134409 21.2 11.6 7.18 7.97

LOC100134413 2.98 4.83 7.43 3.64

LOC100134420 9.06 4.9 23.5 6.53

LOC100134423 6.69 12.3 4.52 7.22

LOC100134576 65.3 57.9 88.2 104

LOC100134663 1540 1810 1210 1490

LOC100134702 104 61.8 83.8 78.1

LOC100134713 271 316 241 334

LOC100134937 53.1 93.1 88.8 267

LOC100144595 3.04 4.95 4.48 3.66

LOC100144597 3.77 5.94 5.71 4.83

LOC100144602 327 1960 4.43 196

LOC100144603 235.5 233.5 279 624.5

LOC100144604 902 1060 792 798

LOC100147773 63.2 60.1 54 82.3

LOC100169752 2.79 4.53 4.11 3.43

LOC100170939 1330 2690 1240 3800

LOC100188947 56.4 72.4 249 94.1

LOC100189589 3.13 5.05 4.65 3.81

LOC100190938 4.25 6.63 6.1 5.15

LOC100190939 271 528 509 655

LOC100190940 3.445 5.385 5.365 4.185

LOC100190986 818 493 879 672

LOC100192378 5.04 15 10.7 31.4

LOC100192379 22.7 24.7 7.18 33.7

LOC100216001 42.3 54.8 4.61 4.68

LOC100216545 434 469 499 585

LOC100216546 259.5 195.5 452 377.5

LOC100233209 800 813 901 851

LOC100240726 132 190 235 364

LOC100240734 3.51 5.62 92.9 90.5

LOC100240735 2.6 4.21 506 377

LOC100268168 45.765 42.435 45.64 49.77

LOC100270679 22.81 29.24 17.115 15.605

LOC100270680 8.67 10.2 5.5 13.3

LOC100270710 3.27 9.51 15.5 11.4

LOC100270746 3.6 226 240 824

LOC100270804 31.7 45.8 45.3 217

LOC100271715 3.45 5.465 5.15 5.335

LOC100271836 9.12 12.9 16.8 18.8

LOC100272216 112 80 126 112

LOC100272228 32.4 32.6 33.7 92.5

LOC100286937 50.8 68.3 49.9 55.9

LOC100286949 35.9 36.4 50 42.1

LOC100286979 94.6 117 110 153

LOC100287006 66.4 75.6 53.4 78.5

LOC100287050 24.3 30.7 28.3 22.1

LOC100287092 172 240 161 185

LOC100287241 12.2 29.3 552 2540

LOC100287245 46 78.3 95.8 114

LOC100287322 37.9 35.6 35.1 48.9

LOC100287359 7.37 9.4 4.99 6.64

LOC100287507 15.6 20.5 16.7 13.5

LOC100287520 26.1 8.38 4.28 3.54

LOC100287593 17400 16700 18200 20200

LOC100287704 3.12 5.07 4.63 3.75

LOC100287737 2.83 4.59 4.16 3.47

LOC100287738 2.77 4.48 4.06 3.38

LOC100287820 655.5 696 839 2125

LOC100287898 69.57 10.975 11.7 5.095

LOC100287919 7.55 7.85 9.07 7.66

LOC100287948 3.17 5.09 4.71 3.84

LOC100288105 3.12 5.12 4.64 3.81

LOC100288106 3930 4300 4690 4780

LOC100288123 6.32 6.38 6.06 5.11

LOC100288271 26.7 24.6 27.9 29.4

LOC100288303 8.04 37.1 35.6 17.2

LOC100288406 12.4 14.8 21.7 17.5

LOC100288412 2860 3030 2860 2800

LOC100288418 110233.333333333 109100 104900 76166.6666666667

LOC100288426 363 115 53.5 268

LOC100288439 4.9 4.8 5.43 22.5

LOC100288455 103 57.5 40 129

LOC100288578 145000 140000 141700 83350

LOC100288600 22.6 183 9.71 16.6

LOC100288667 80.025 79.8 102.275 107.85

LOC100288678 11.5 5.18 9.12 59.6

LOC100288690 25.2 25.9 31 30.8

LOC100288701 11.4 11.3 107 7.22

LOC100288755 36.6 21.6 26.1 32.1

LOC100288765 6.56 14.6 26.7 3.68

LOC100288842 32.6 77.3 96.2 104

LOC100288884 22.65 28.05 12.7 15.43

LOC100288891 237.9 280.1 197.266666666667 270.233333333333

LOC100288900 4.41 5.96 5.71 14.4

LOC100288921 7.64 10.7 14.8 12.7

LOC100289004 284 291 289 270

LOC100289026 25.6 42.9 7.47 77.3

LOC100289079 55.5 28.7 39.6 85.3

LOC100289097 612 1990 759 791

LOC100289169 244 170 285 220

LOC100289178 4.08 8.09 5.34 4.6

LOC100289208 3.75 15.2 6.76 15.4

LOC100289258 187000 134000 170000 121000

LOC100289290 6.34 16.8 13.2 3.22

LOC100289383 160.95 139.2 138.45 207.2

LOC100289410 2340 70.9 17.7 765

LOC100289509 53.8 37.2 31.9 46.5

LOC100289600 676 746 623 508

LOC100289649 4.93 4.74 4.34 5.43

LOC100289760 8.71 7.23 15.5 10.3

LOC100289922 3.29 5.36 19.8 3.9

LOC100289949 2.55 4.12 3.74 3.14

LOC100290115 3.6 5.76 6.14 4.27

LOC100290344 7230 7110 5250 3280

LOC100290415 3.74 11.7 6.57 17.2

LOC100290819 11.7 9.03 17 10.6

LOC100290859 509 510 858 502

LOC100291056 3.77 6.03 5.62 4.52

LOC100291104 3.55 5.68 5.24 4.22

LOC100291206 17500 21700 20000 21900

LOC100291610 9.42 4.74 7.71 17.7

LOC100291656 10.4 17.4 16.2 8.9

LOC100291714 18.9 20.8 51.4 33.6

LOC100291791 912 1480 787 963

LOC100291851 5.89 5.99 5.12 3.26

LOC100292021 20.8 75.4 92 107

LOC100292196 11.3 5.68 19.4 18.8

LOC100292270 55.6 57.6 56.5 44.9

LOC100292387 4.16 6.34 6 8.51

LOC100292409 192 200 288 153

LOC100292420 3.04 4.92 4.47 3.71

LOC100292427 14.6 11.5 26.9 24.7

LOC100292626 3.38 5.47 5.04 4

LOC100292680 3.9 6.2 10.6 5.57

LOC100292717 1700 1720 2280 1380

LOC100292758 20 24.8 30.9 42.1

LOC100292768 3.45 4.13 3.74 3.14

LOC100292961 3.39 5.38 4.89 3.96

LOC100292999 3.9 4.81 5.4 4.725

LOC100293090 74600 78800 64700 31200

LOC100293142 23 22.7 19.1 22.8

LOC100293193 19.3 5.4 776 3200

LOC100293277 3.04 4.92 4.46 3.66

LOC100293318 3.64 5.77 5.49 4.51

LOC100293352 2.99 4.85 4.41 3.66

LOC100293406 2.91 11.1 4.24 6.08

LOC100293435 3.52 5.7 5.25 4.15

LOC100293440 10.8 42.9 11.1 35.1

LOC100293467 2.86 4.65 4.21 3.5

LOC100293499 29.8 22.1 13.6 18.8

LOC100293560 3.96 6.21 5.72 4.63

LOC100293561 12.5 4.64 4.55 8.43

LOC100293590 6.59 4.62 4.22 9.15

LOC100293611 1290 496 793 644

LOC100293798 67.5 51.1 111 58.5

LOC100294005 140 136 118 145

LOC100294070 2573.3 2402.06666666667 2070.63333333333 1745.8

LOC100294100 14.9 10.6 8.5 20.4

LOC100294232 85.2 80.7 103 80.7

LOC100294391 39 38.5 58.1 27.7

LOC100294464 57.1 26.4 34.5 22.8

LOC100294501 11.865 5.085 4.685 6.62

LOC113230 706 773 506 525

LOC115110 170.9 24.44 82.35 22.95

LOC116437 3.135 9.69 6.1 5.715

LOC119358 13600 16000 18500 20200

LOC120364 978 1740 1050 2590

LOC121838 6.2 6.81 4.36 13.3

LOC121906 22.5 23.3 12 12

LOC121952 3.24 5.27 4.83 3.93

LOC123855 77 273 78.2 166

LOC126536 2.62 4.23 3.85 3.23

LOC126987 31 4.68 248 6.04

LOC127841 68.9 8.31 31.9 6.75

LOC128322 293.055 498.47 632.73 692.16

LOC131055 69900 39900 58200 38200

LOC134466 3.81 205 5.6 4.48

LOC134505 2810 3100 3670 3240

LOC139431 3.14 9.64 4.66 3.89

LOC142937 138 4.69 188 251

LOC143188 55.75 123.75 64.45 163.45

LOC143286 267 286 334 342

LOC143666 45.9 27.2 45.9 60.2

LOC144438 698 869 1100 1560

LOC144486 12.5 18.2 59.5 40.2

LOC144571 156 200 139 175

LOC144742 6.13 5.47 5.28 6.55

LOC144766 270 369 317 207

LOC144817 9.91 12.5 8.02 7.27

LOC144874 64.1 159 128 705

LOC144920 84.8 14.3 4.36 7.83

LOC145216 110 106 114 108

LOC145474 646 536 535 507

LOC145678 7.18 5.67 5.26 4.25

LOC145694 25 61.2 153 103

LOC145783 17.3 89.9 18.7 4.37

LOC145814 47 25.1 19.3 12.5

LOC145820 158 174 143 317

LOC145837 19.6 22.5 115 21.8

LOC145845 93.9 129 83.2 88.4

LOC145945 32.8 27.3 27.9 31.6

LOC146336 3.28 4.5 5.68 3.41

LOC146429 3430 4420 3630 2600

LOC146481 2.85 9.52 4.2 3.46

LOC146513 3.79 6.08 5.56 5.35

LOC146795 151 120 139 116

LOC146880 27.3 28.3 32.7 24

LOC147004 7.58 7.84 10.6 9.94

LOC147093 3.22 15.6 4.79 10.3

LOC147646 36.1 42.2 54.8 66.7

LOC147670 102 25.3 18.8 56.1

LOC147727 937 658 2630 2030

LOC147804 450 737 680 479

LOC148145 7.53 12.8 11 25.9

LOC148189 320 622 766 313

LOC148413 321 185 417 380

LOC148638 6.59 12.5 9.68 17.8

LOC148696 43.5 37.8 35.4 33.5

LOC148709 2180 1740 2990 2190

LOC148987 7.36 4.52 87.9 31.3

LOC149086 184 197 203 192

LOC149134 55.6 70.3 48 68.5

LOC149157 9.71 17.1 9.02 20.6

LOC149351 264 402 211 628

LOC149773 13.3 9.23 11.6 9.03

LOC150005 5.65 4.76 4.55 14.9

LOC150051 17.4 25.7 82 126

LOC150185 3.69 5.82 5.57 4.61

LOC150197 13.8 7.2 11.2 12.8

LOC150381 3.85 4.23 113 15.8

LOC150527 118.4 125.25 105.95 105.7

LOC150568 2.56 4.15 3.77 3.16

LOC150577 3.11 4.99 4.58 3.78

LOC150622 16.38 13.195 18.77 13.005

LOC150759 38.9 70.3 234 552

LOC150786 4.05 6.22 5.79 34.6

LOC151009 114.1 115.9 206.5 118.45

LOC151121 3.24 5.29 4.81 90.5

LOC151146 503 498 1310 1200

LOC151162 2240 9740 11400 6180

LOC151174 179 146 125 124

LOC151234 6.89 9.11 15.2 11.5

LOC151300 4.665 7.31 9.27 8.035

LOC151438 6.22 13.9 95.7 8.32

LOC151475 11 5.89 13.3 5.31

LOC151484 12.9 13.4 21.7 8.71

LOC151657 4.75 5.99 17.7 28.2

LOC151658 3.77 5.77 5.68 4.95

LOC151760 3.27 7.98 4.84 35.6

LOC151877 28.4 24.4 27.9 32.7

LOC151878 212 113 167 183

LOC152024 12.1 19.4 10.1 6.91

LOC152217 20800 10100 21600 9440

LOC152225 12 12.6 4.64 3.73

LOC152274 4.44 5.59 5.15 4.15

LOC152286 16.6 4.63 13.4 8.81

LOC152578 3.19 8.83 4.75 3.84

LOC153328 3.74 5.93 9.07 101

LOC153469 3.62 5.87 5.42 4.44

LOC153546 487 132 852 286

LOC153577 43.7 65.6 70.5 30.1

LOC153684 91.6 307 6.81 19.9

LOC153811 147 82.5 89.3 312

LOC153910 3.6 16.9 4.99 6.89

LOC154092 3.54 5.66 6.92 4.22

LOC154449 3.82 6.06 5.58 4.48

LOC154761 3.01 4.9 4.45 3.68

LOC154860 98.2 77.6 87.3 97.6

LOC154872 3.36 5.34 4.92 4.06

LOC157273 7.26 5.96 5.51 4.45

LOC157278 13000 14800 15300 14900

LOC157381 49.69 59.67 60.465 62.14

LOC157503 3.14 5.07 4.66 3.82

LOC157562 108 140 44 62

LOC157627 9.32 7.54 117 8.56

LOC157740 297 281 313 322

LOC157860 77.1 662 38.6 96.7

LOC157931 2.87 4.64 4.21 3.49

LOC158257 20.9 39.7 20.1 17.1

LOC158376 3.87 8.99 31.1 3.93

LOC158381 298.16 361.14 231.93 229

LOC158402 36.7 73.8 4.59 29.8

LOC158434 3.96 6.17 5.79 4.74

LOC158435 2900 2960 3210 3780

LOC158572 2.85 28.2 6.37 44.2

LOC158696 6.46 8.12 6.57 25.3

LOC158863 64.9 67 61.8 108

LOC158960 55.5 49.8 38.1 56.1

LOC159110 8600 9270 8030 8980

LOC168474 2.97 4.8 4.36 3.62

LOC169834 4.66 5.87 5.47 28.8

LOC170425 44.7 128 96.7 188

LOC171220 150 638 283 160

LOC1720 51.7 47.3 110 54.2

LOC197350 19.5 23.2 15.3 4.94

LOC199897 3.74 5.76 5.64 4.93

LOC199899 2.7 4.38 3.97 9.91

LOC200261 29 20 30.4 24.7

LOC200609 9.05 31.8 6.7 44.1

LOC200772 6.51 4.41 13.8 11.5

LOC200830 471 457 353 369

LOC201477 23.9 37.5 20.9 45.7

LOC201617 37.4 12.8 32.1 6.05

LOC201651 74 28.4 4.67 5.31

LOC202181 50 27.8 40.8 289

LOC202781 65.4 68.2 92.9 100

LOC203274 981 1430 731 1880

LOC203510 208 322 104 694

LOC219347 4.23 5.66 10 6.44

LOC219690 3.22 5.23 4.79 3.83

LOC219731 5.93 13.1 17.2 20.5

LOC220077 7.45 57 63.1 49.3

LOC220115 8.40333333333333 54.8333333333333 6.40333333333333 8.49

LOC220429 214.5 99.9 98.15 73.5

LOC220729 2220 2360 9160 1840

LOC220930 56 64.6 294 97.3

LOC220980 5.17 6.42 6.07 5.05

LOC221122 3.26 5.16 4.85 3.86

LOC221272 464 317 486 521

LOC221442 54.9 46 15 133

LOC221710 240 126 169 227

LOC221814 26.3 25.5 26.5 62.2

LOC221946 32.3 32.9 63.6 33.5

LOC222070 2510 1680 2930 1940

LOC222159 70.4 33.9 61 47.8

LOC222699 37.3 45.1 3.93 295

LOC253039 65.5 48.9 91.9 4.41

LOC253044 3.13 5.09 4.65 11.1

LOC253264 4.57 5.62 6.98 14

LOC253573 3.22 5.18 5.41 11.8

LOC253724 13.4166666666667 25.1933333333333 8.83333333333333 20.1466666666667

LOC253805 8.22 18.5 8.93 16.3

LOC253962 9.5 4.23 3.85 4.17

LOC254057 2860 2040 3400 3090

LOC254099 20.7 7.3 95.6 4.64

LOC254100 22.9 23.1 29.1 42.1

LOC254128 50.7 79.9 62.5 66.9

LOC254312 2.66 4.32 3.91 3.27

LOC254559 114 145 135 130

LOC255025 3.29 5.36 5.92 3.9

LOC255167 3.36 5.4 4.98 4.07

LOC255177 364 570 451 420

LOC255411 4.28 15.3 14.3 5.66

LOC255480 2.7 4.37 3.96 3.55

LOC255512 119 55 287 150

LOC255654 12.6 10.6 27.6 16.3

LOC256483 3.35 8.21 4.45 3.61

LOC256880 9.76 16.3 17 30

LOC257152 888 1230 854 1000

LOC257358 3.86 6.16 9.38 5.69

LOC257396 38 52.65 101.65 173.15

LOC25845 742 665 1530 2260

LOC26080 5.63666666666667 5.69666666666667 5.65333333333333 3.60333333333333

LOC26102 21.6 18.8 16.2 15.7

LOC282980 7.45 5.44 13.1 4.76

LOC282997 190 62.8 140 294

LOC283028 112 136 130 488

LOC283033 3.75 6.02 5.54 4.41

LOC283038 102 95.2 178 90

LOC283050 16.1 109 6.54 14.4

LOC283070 3.25 5.14 4.98 329

LOC283075 53.8 58.2 32.2 37

LOC283079 3.87 6.19 5.65 4.54

LOC283089 3.24 5.22 4.82 36

LOC283104 13.6 19.3 14 29.1

LOC283112 6.02 15 7.49 6.86

LOC283140 11.9 13.4 10.8 22.7

LOC283143 3.01 4.72 4.43 3.62

LOC283174 2.84 4.59 4.2 220

LOC283177 25.3 22.2 14.5 17.6

LOC283214 3.29 5.26 4.9 3.9

LOC283267 754 914 279 2330

LOC283270 1120 1050 843 666

LOC283332 3.12 5.08 4.63 3.8

LOC283335 479 453 433 375

LOC283352 58.5 354 176 133

LOC283378 1500 1810 2450 2340

LOC283387 22.2 112 34.6 23.5

LOC283392 8.2 9.1 432 92.15

LOC283398 402 258 289 334

LOC283403 3.31 5.4 4.92 3.93

LOC283404 3.05 4.98 4.52 3.68

LOC283435 3.37 5.35 4.96 4.16

LOC283440 22.3 25.6 32.5 15.3

LOC283454 91.5 5.34 4.83 1430

LOC283475 3.59 4.45 4.05 3.39

LOC283480 3.17 5.08 4.66 3.84

LOC283481 60.7 11.3 5.49 15.7

LOC283482 6.15 4.14 3.76 5.18

LOC283483 8.36 5.21 4.84 17.4

LOC283484 3.64 5.67 144 4.75

LOC283485 3.91 6.15 5.75 9.07

LOC283486 2.93 4.78 4.32 3.55

LOC283501 3.18 119 9.6 5.92

LOC283516 3.88 21.2 3.81 21.4

LOC283547 1070 975 927 1390

LOC283575 17.5 18.4 16.7 16.7

LOC283585 58.7 71.7 64.9 73

LOC283587 81.2 229 12.6 73.3

LOC283588 6.07 4.29 30.3 149

LOC283624 53.7 84.7 55.2 182

LOC283663 113 155 204 133

LOC283665 3.5 5.69 5.22 7.59

LOC283674 11.4 44.6 27.4 8.04

LOC283682 5.8 5.54 10.5 6.59

LOC283692 25.8 5.43 4.99 4.07

LOC283693 35.1 27.1 53.4 53.6

LOC283710 20.6 20.1 104 12.3

LOC283711 6.13 5.49 18.3 4.57

LOC283713 4.98 5.28 9.62 24.1

LOC283728 4.02 6.21 6.87 28.4

LOC283731 3.81 12.7 8.31 21.9

LOC283737 3.46 5.64 5.19 4.38

LOC283738 3.57 5.77 5.32 4.35

LOC283761 12.1 14.5 20.6 11.4

LOC283788 634.555 1277.9225 1266.565 797.2625

LOC283854 58 64.3 87 67.7

LOC283856 20.7 21.4 17.9 19.8

LOC283861 56 105 492 400

LOC283867 8.08 5.58 5.16 4.2

LOC283887 17.6 24.1 29 24.5

LOC283888 94.5 62.5 105 88

LOC283904 2.88 4.67 4.23 39.1

LOC283911 1270 1500 1430 1890

LOC283914 3.42 5.55 5.1 4.17

LOC283922 67.6 21.3 51 49.3

LOC283999 19.81 37.52 32.8 28.645

LOC284009 48 28.1 50.35 63.25

LOC284014 2100 2400 2520 2540

LOC284023 6.75 13.5 18.9 25.8

LOC284033 3.56 5.74 5.36 4.37

LOC284072 5.97 4.66 10.4 3.53

LOC284080 2.98 4.74 34 3.6

LOC284100 3.94 6.15 5.77 4.72

LOC284108 4.86 6.66 4.79 51.6

LOC284191 2.81 4.53 68.2 3.43

LOC284215 3.3 5.33 4.91 3.91

LOC284219 643 596 356 174

LOC284232 76.5666666666667 185.766666666667 68.7 209.9

LOC284240 6.84 6.13 11.4 10.5

LOC284242 27.6 27.7 106 199

LOC284244 3.12 5.11 8.73 297

LOC284260 36.6 16 23.2 52.8

LOC284263 9.52 15.1 12.7 9.33

LOC284276 6.73 14.4 7.075 10.075

LOC284288 976 758 641 701

LOC284294 2.89 4.7 4.26 3.54

LOC284373 3510 3540 2500 2990

LOC284379 1381.9 1332.565 1216.5 1170.7

LOC284395 3.14 4.93 7.41 3.76

LOC284408 77.4 6.22 12.8 30.8

LOC284412 16.8 9.26 12.6 12.8

LOC284440 5.55 61.7 12 3.61

LOC284441 1690 1510 1480 1510

LOC284454 1040 991 1010 883

LOC284475 42.8 86.6 52.6 68.8

LOC284513 184 184 604 605

LOC284542 2.94 4.79 4.36 3.58

LOC284551 10.5 7.31 7.77 4.41

LOC284561 8.71 87.9 57.8 19.3

LOC284570 3.15 5.11 4.65 3.84

LOC284576 6.2 9.37 15.2 3.36

LOC284577 3.09 6.82 13.6 3.89

LOC284578 3.555 5.44 5.115 4.345

LOC284600 263000 252000 285000 261000

LOC284620 16.6 523 25.7 23

LOC284628 46 38.8 29.2 35.5

LOC284630 9.99 6.03 42.5 12.8

LOC284632 3.21 5.15 4.77 3.89

LOC284648 3.29 5.34 4.9 3.91

LOC284661 3.95 5.91 5.49 4.41

LOC284669 68.9 70.7 15 11.4

LOC284749 176 202 186 166

LOC284751 90.4 211 167 7.91

LOC284757 90.6 127 74.6 79.7

LOC284788 3.59 5.76 5.9 4.36

LOC284798 4.09 6.31 5.94 4.91

LOC284801 3.02 20.6 4.44 3.64

LOC284805 3.75 5.97 5.65 9.29

LOC284825 3.79 5.97 5.55 4.51

LOC284837 85.4 66.9 84.8 41.7

LOC284898 3.95 6.18 5.81 4.75

LOC284900 124.366666666667 228.2 164.266666666667 236.866666666667

LOC284926 13.5 16.6 30.9 28.7

LOC284930 3.76 6.08 9.45 4.64

LOC284933 2010 2180 2070 2280

LOC284939 9.13 40.5 18 357

LOC284950 15.1 5.19 4.73 3.8

LOC284998 3.52 5.7 7.95 4.34

LOC285000 3.37 5.48 5.01 3.98

LOC285033 16.3 8.41 12.1 18.1

LOC285043 2.91 4.67 4.29 3.53

LOC285045 3.31 5.22 4.89 3.96

LOC285084 2.69 4.36 3.95 3.29

LOC285095 140.93 203.1 133.95 299.8

LOC285147 167 33.3 52.3 176

LOC285173 7.56 12.8 17.3 14

LOC285178 600 1170 1770 1080

LOC285181 34.9 4.71 8.33 5.01

LOC285194 2.62 4.25 3.85 3.23

LOC285205 3.7 5.94 5.52 4.85

LOC285216 3.03 4.93 4.49 3.68

LOC285224 3.16 5.07 4.62 3.79

LOC285281 10.6 6.56 3.92 115

LOC285286 3.9 5.38 5 9

LOC285300 5.94 4.55 6.11 21.6

LOC285370 3.99 5.89 5.39 4.29

LOC285375 2.8 4.55 4.12 3.42

LOC285389 19.1 7.98 4.46 11.4

LOC285401 30.1 37.4 28.8 28

LOC285419 4.96 46.7 62.2 4.24

LOC285422 26.9 4.7 4.35 3.59

LOC285441 3.03 6.27 4.49 4.2

LOC285456 2.55 154 3.75 3.15

LOC285484 10.6 6.22 5.7 17.1

LOC285500 118 179 110 151

LOC285501 3.25 5.29 4.85 3.93

LOC285505 3.64 5.8 5.42 4.3

LOC285547 3.62 5.82 5.33 4.26

LOC285548 30.7 75 4.82 7.91

LOC285550 178 231 53.3 113

LOC285556 3.57 10.1 5.37 4.36

LOC285577 3.61 11 16.2 3.8

LOC285593 2.79 4.54 15 3.4

LOC285626 2.98 4.85 4.39 6.85

LOC285627 3.25 5.24 4.83 3.96

LOC285628 235 4.83 18.2 3.38

LOC285629 9.76 10.9 11.4 13.4

LOC285638 3.3 5.37 4.91 4.02

LOC285692 3.88 6.17 5.76 4.67

LOC285696 3.705 12.815 6.86 4.31

LOC285697 2.7 4.37 3.96 3.31

LOC285733 6290 2960 63.1 6.28

LOC285735 3.34 11.9 4.98 4.05

LOC285740 37.865 9.54 11.195 10.765

LOC285758 224 183 50.6 49.3

LOC285762 8.75 15.7 11.1 17.8

LOC285766 2.84 4.62 4.18 3.48

LOC285768 3.87 6.06 7.35 4.6

LOC285771 34 46.3 38.5 43.2

LOC285778 3.3 5.37 4.9 3.92

LOC285796 3.78 5.8 5.7 4.96

LOC285804 16.7 15.6 20.6 13.5

LOC285819 2.79 4.51 4.09 3.42

LOC285827 3.08 5 4.54 3.75

LOC285857 8.27 6.26 8.01 10.6

LOC285878 3.46 5.61 5.17 4.22

LOC285889 22.5 65.2 8.78 17.1

LOC285943 11.7 8.92 95.4 20.7

LOC285944 3.5 13.2 16.9 26.1

LOC285954 5.77 5.25 5.27 3.98

LOC285957 28.6 26.7 29.5 30.7

LOC285965 2.62 4.24 3.84 3.22

LOC285972 5.34 11.1 16.2 6.98

LOC286002 3.14 5.07 4.61 3.78

LOC286052 124 124 229 792

LOC286058 41.4 7.4 6.34 10.9

LOC286059 4.04 9 6.01 11.6

LOC286063 2.88 9.26 10.9 107

LOC286071 50.9 20.3 39.1 25.5

LOC286083 8.14 7.86 5.77 4.78

LOC286087 27.6 21.9 23.6 22.9

LOC286094 3.41 5.53 5.08 4.16

LOC286109 281 150 220 228

LOC286114 3.78 5.99 5.69 4.71

LOC286121 3.77 14.4 20.2 20.9

LOC286123 2.91 4.67 4.27 3.54

LOC286126 3.72 6.01 5.58 4.58

LOC286135 2.95 4.69 4.33 3.58

LOC286149 63.8 4.5 4.12 3.44

LOC286154 12.3 12.1 23.2 19.9

LOC286161 726 238 315 432

LOC286177 7.22 4.97 4.69 3.76

LOC286178 4.01 6.22 5.87 4.85

LOC286184 2.82 4.58 9.64 3.43

LOC286186 23.2 27 35.5 28.4

LOC286189 3.38 5.44 5.05 4.07

LOC286190 3.01 4.86 4.46 7.8

LOC286238 3.33 5.33 4.89 4.01

LOC286254 79.8 73.6 117 138

LOC286272 27.3 12 22.8 236

LOC286299 3.33 4.12 5.93 3.32

LOC286367 79.1 72 12.9 171

LOC286370 5.09 53.8 17.5 4.03

LOC286382 216 308 169 199

LOC286434 143602 138124.5 163764.5 155367

LOC286437 338 700 189 1200

LOC286442 15.7 13.9 5.58 9.58

LOC286467 45.7 9.2 4.29 60

LOC286528 67.3 37.1 33.2 12.3

LOC29034 6.7 7.61 7.22 3.75

LOC338579 9.93 11.1 7.72 12.4

LOC338588 5.72 6.35 5.96 4.89

LOC338620 209 560 108 174

LOC338651 3.47 5.43 5.2 4.1

LOC338653 34.6 6.42 8.32 5.12

LOC338667 2.78 4.52 4.1 3.39

LOC338694 12.1 10.7 10 13

LOC338739 30.8 42.3 33.8 34.6

LOC338756 15800 29200 14600 19200

LOC338758 7.86 17.6 12.2 4.05

LOC338799 537 356 773 1190

LOC338817 7.32 5.86 51.2 29.9

LOC338864 2.65 4.29 3.89 3.25

LOC338963 12.1 10 14.2 43.2

LOC339192 19.2 15.1 71.8 27.1

LOC339240 17.0666666666667 21.8366666666667 49.28 26.5666666666667

LOC339260 3.92 6.18 5.8 4.71

LOC339290 2.91 75.5 4.27 5.64

LOC339298 3.17 5.11 4.71 3.88

LOC339316 3.81 12.2 5.6 4.47

LOC339352 2220 986 2190 2250

LOC339400 3.98 6.17 5.77 4.72

LOC339442 13.3 12.5 16.1 15

LOC339468 3.35 5.43 5.38 4.08

LOC339505 2.84 4.62 4.19 3.46

LOC339524 4.67666666666667 16.37 16.6233333333333 26.76

LOC339529 18.8 5.72 5.33 4.32

LOC339535 5.545 6.76 5.07 4.225

LOC339539 3.06 4.96 4.53 3.68

LOC339568 15.8 5.58 5.14 4.15

LOC339593 3.44 7.83 10.5 6.96

LOC339622 3.04 4.87 4.5 11

LOC339666 5.92 7.05 11.8 12.8

LOC339674 18.8 9.095 66.55 192.3

LOC339685 7.35 9.83 6.69 4.36

LOC339742 62.605 48.81 50.94 82.94

LOC339751 3.67 5.93 5.49 4.49

LOC339760 4.69 6.16 5.64 4.54

LOC339788 3.04 4.77 4.47 3.65

LOC339803 849 1260 1120 846

LOC339807 98 103 105 92.3

LOC339822 2.83 4.55 4.15 3.45

LOC339862 43.9 86.8 4.14 11.9

LOC339874 6.38 8.78 7.75 7.58

LOC339894 3.5 5.155 4.855 3.9

LOC339926 3.34 5.37 4.95 4.02

LOC339929 43.5 44.2 191 12.5

LOC339975 8.74 20.5 5.6 4.61

LOC339988 11.3 22.7 18.8 76.1

LOC340017 3.64 14 5.25 4.24

LOC340037 7.16 4.5 36.2 3.4

LOC340073 21.1 29.1 23.4 25.4

LOC340074 5.88 4.32 3.93 3.29

LOC340090 3.71 5.95 5.58 4.58

LOC340094 3.07 4.95 6.555 3.77

LOC340096 3.71 4.66 4.25 3.48

LOC340107 2.86 4.64 4.2 3.48

LOC340113 3.78 6.01 5.54 4.45

LOC340178 31.8 29.6 84.2 6.94

LOC340335 162 168 232 378

LOC340357 23.8 24.6 25.9 35.4

LOC340508 714 1620 1580 181

LOC340512 2.58 4.18 3.79 3.18

LOC340515 107 70.1 83.9 66

LOC340581 3.05 4.92 4.52 3.73

LOC340900 2.93 4.77 4.34 4.86

LOC340970 25.73 39.295 21.71 38.765

LOC341378 36.8 42.8 30.3 22.3

LOC342293 6.06 6.16 8.94 3.51

LOC342346 7.06 6.07 9 6.05

LOC342918 52.7 61.7 69.8 48.7

LOC343052 3.01 4.91 4.44 3.63

LOC344065 92.1 65.2 5.27 160

LOC344382 779 1780 1770 1790

LOC344887 159.05 172.65 332.3 3.335

LOC344967 72.9 98.6 276 102

LOC345645 2650 4870 3780 4330

LOC346329 157 118 159 197

LOC346702 7.61 4.77 4.36 12.2

LOC346887 372 181 5100 5470

LOC347281 3.25 5.28 4.84 3.93

LOC347411 2.84 4.61 4.18 3.48

LOC348021 16.2 11 17.4 16.4

LOC348751 87.3 14.6 8 46.8

LOC348761 86.3 173 3.93 265

LOC348817 2.59 5.29 3.8 3.2

LOC348840 30 25.2 46.1 52.2

LOC349114 1328.35 1769.25 1373.75 2074

LOC349160 6.04 18.3 14.4 24.5

LOC349196 8.9 7.555 8.85 19.7

LOC349408 4.08 6.23 5.83 4.81

LOC360030 2.99 4.88 4.44 3.64

LOC374443 45.8 114 70.8 73.1

LOC374491 36 134 43.1 125

LOC374890 155 138 96.8 94.3

LOC375190 39.3 80.6 27.8 269

LOC375196 45.4 5.03 4.62 3.72

LOC375295 3.94 34.7 105 240

LOC386597 46.4 38.5 45.4 42

LOC387646 51.5 130 7.09 13.2

LOC387647 2150 1570 1000 691

LOC387720 2.77 24.9 4.07 4.05

LOC387723 4.21 4.63 14.9 3.53

LOC387763 8.91 36 5.63 777

LOC387771 26.4 11.5 5.12 4.14

LOC387810 2.98 4.85 4.39 3.6

LOC387876 3.53 5.69 5.21 4.18

LOC388079 971 852 456 938

LOC388152 851 1099.75 937.5 1842.75

LOC388210 4.55 5.9 5.72 13.1

LOC388242 19.7 24.6 41.8 21.9

LOC388271 8.92 11.6 15.5 11

LOC388279 23.8 30.8 120 139

LOC388387 32.5 90.1 25.2 39.5

LOC388414 3.36 5.42 5.01 4.1

LOC388428 8.54 4.11 3.73 3.13

LOC388456 3.57 5.74 5.37 4.36

LOC388458 2.68 4.35 3.93 3.29

LOC388514 6.41 19.4 5.31 4.22

LOC388553 3.16 5.06 4.7 5.99

LOC388564 17100 13500 17000 9200

LOC388588 387 219 488 429

LOC388630 19.1 150.45 396.95 110.55

LOC388692 593.5 412.5 269 394.5

LOC388780 260 413 55.4 551

LOC388789 23300 24800 18400 21200

LOC388796 6195.65 1793 2345.5 3605

LOC388813 44.7 39.2 46.7 63.8

LOC388849 7.22 9.41 7.51 8.54

LOC388882 8.41 5.99 5.56 8.32

LOC388889 350 292 205 212

LOC388906 40 5.94 95.2 89

LOC388946 4.22 6.09 5.58 4.58

LOC388948 156 88.4 54.8 69.9

LOC388965 1060 1310 502 2650

LOC389023 2.86 4.61 4.22 3.52

LOC389024 114 21.4 25.6 47.8

LOC389033 565 552 521 532

LOC389043 4.19 6.38 6.04 5.04

LOC389217 1520 2310 1650 2050

LOC389247 46.3 21.8 8.58 30.6

LOC389300 4.12 6.36 6 4.97

LOC389332 5000 127 11.1 8.31

LOC389333 23.8 22.3 31.4 33.2

LOC389458 1740 2330 1820 1820

LOC389493 368 757 963 448

LOC389634 21.778 26.006 40.48 377.38

LOC389641 45.1 57.1 13.9 4.32

LOC389676 5.89 6.18 12 4.88

LOC389765 205 281 359 601

LOC389791 20.3 96.7 22.6 96.8

LOC389831 2160 862 1840 597

LOC389834 315.914285714286 357.528571428571 205.454285714286 257.485714285714

LOC389842 1980 3560 1360 3490

LOC389857 26.065 25.54 22.21 41.65

LOC389895 4.1 6.39 6.01 201

LOC389936 3.11 5.07 4.61 3.75

LOC390213 17.1 11 14.9 8.16

LOC390251 49.2 46.3 37.4 43.2

LOC390282 8740 7500 9850 11000

LOC390298 22 16.5 39.7 21.7

LOC390424 4750 8450 11000 12600

LOC390557 213 211 95.5 145

LOC390595 149 235 161 230

LOC390638 13.95 9.24 15.1 13.65

LOC390660 47.8 44.4 31.5 65.3

LOC390705 2.93 4.68 4.3 3.55

LOC390748 20 12.7 23.2 17.4

LOC390760 12.2 4.9 7.94 8.86

LOC390806 1030 1770 967 1440

LOC390834 349 231.466666666667 251.533333333333 289.333333333333

LOC390937 77.3 71.6 91.5 76.7

LOC390940 36.7 58.3 114 70

LOC390998 179 6190 1500 6380

LOC391073 32.8 35.7 37.8 33.3

LOC391081 13.9 14.6 20.3 15.9

LOC391322 67.4 4.44 4.02 3.33

LOC391334 14600 10700 12200 8710

LOC391358 17100 13600 14200 22000

LOC391359 130 219 1730 2280

LOC391578 77.4 57.3 92.1 101

LOC391636 13 21 26.3 27.2

LOC391767 29.6 27.2 31.1 41.1

LOC391769 77900 66200 92300 125000

LOC392196 208 116 93.2 73.9

LOC392232 3.29 5.33 4.88 8.7

LOC392275 12.3 12.5 24.9 10.4

LOC392288 563 423 364 555

LOC392335 849 166 1620 149

LOC392435 2.97 4.83 4.38 3.59

LOC392452 3.91 6.16 5.64 4.6

LOC399708 3.64 5.89 5.44 4.45

LOC399715 21.6 5.34 140 11.7

LOC399744 967.025 1313.8 1025.9 1175.65

LOC399804 15700 25800 18000 22600

LOC399815 65.6 156 342 118

LOC399829 3.53 4.57 7.52 7.12

LOC399875 14.2 6.39 8.03 5.16

LOC399881 2.95 4.81 4.37 3.57

LOC399904 13.7 7.63 14.3 10.8

LOC399959 17 4.7 4.3 3.56

LOC400019 2310 1590 1480 1400

LOC400027 111 146 565 286

LOC400043 1200 2930 57.3 3530

LOC400084 2.91 4.71 4.3 3.51

LOC400099 696 359 2530 515

LOC400128 17600 24500 17700 18800

LOC400236 303 215 280 546

LOC400238 19.3 16.4 9.45 13.6

LOC400406 139.25 177.565 139.355 281.9

LOC400419 392.945 499.1 399.835 449.45

LOC400456 19.4 5.44 5.02 23.9

LOC400499 21.8 9.49 15.6 39

LOC400541 3.09 4.86 12.6 3.72

LOC400548 3.45 5.66 5.14 4.29

LOC400558 16000 14900 10800 13100

LOC400568 3.01 4.88 4.42 3.63

LOC400573 10.9 14 6.24 12.2

LOC400578 9.22 7.41 9.92 8.48

LOC400604 49700 55500 59800 60500

LOC400620 3.42 5.52 5.06 4.15

LOC400622 3.71 6 5.55 4.55

LOC400654 3.14 22.1 4.76 20.8

LOC400655 2.75 4.43 4.03 3.37

LOC400657 38.16 41.055 98.9 50.05

LOC400662 79.3 70.7 141 82.3

LOC400682 8.96 4.17 4.22 4.61

LOC400684 130 247 287 159

LOC400685 12 18 13.5 14.4

LOC400696 8.39 12.9 9.93 10.5

LOC400743 30.09 31.7 103.6 44.45

LOC400748 3.78 6.06 5.64 4.57

LOC400756 99.8 50 53.1 18

LOC400759 3.77 5.31 6.53 6.45

LOC400768 2.92 4.66 4.26 3.54

LOC400794 2.89 4.7 4.27 3.49

LOC400804 115 175 110 176

LOC400891 3.41 5.445 5.06 4.06

LOC400927 54.2 55.7 39.6 99.45

LOC400931 174.5 24.04 820.1 49

LOC400940 3.86 34.5 7.38 8.75

LOC400958 2.63 4.26 3.86 3.23

LOC400965 14.8 7.84 5.48 8.95

LOC400968 516 460 408 346

LOC400986 50.8 151 487 183

LOC400987 343 482 877 1010

LOC401021 2.81 60.3 19.1 44

LOC401022 31 622 4.5 329

LOC401037 21.6 40 23 37.2

LOC401052 4.24 4.97 8.41 3.69

LOC401068 20.6 5.73 15.5 36.2

LOC401097 12.32 84 8.33 102.45

LOC401098 3.07 4.99 4.54 3.73

LOC401127 776 742 1160 1090

LOC401134 3.15 5.12 4.66 3.84

LOC401164 16.2 24.6 22.2 22.5

LOC401177 2.84 4.61 4.2 3.45

LOC401180 9.64 9.45 15.1 11.9

LOC401188 35.5 29 40.8 36

LOC401218 298 536 367 1210

LOC401220 3.21333333333333 5.21666666666667 4.75 3.85333333333333

LOC401233 88.745 76.8 53.59 53.8

LOC401312 3.215 5.18 7.945 3.89

LOC401387 34.08 3872.155 2056.955 1363.785

LOC401397 4150 5245 7215 11740

LOC401400 3.48 5.55 5.18 4.19

LOC401431 29.3 5.24 477 1440

LOC401433 3.25 5.18 4.8 5.96

LOC401442 2.56 4.14 3.75 3.15

LOC401445 3.76 6.06 5.55 4.4

LOC401463 3.47 5.64 5.17 4.09

LOC401480 1720 1950 2110 2010

LOC401490 3.8 6.12 5.71 4.69

LOC401497 3.69 17.9 5.44 4.41

LOC401504 2450 8560 2040 4240

LOC401561 66.3 63.2 100 68.4

LOC401577 57.4 27.7 109 78.2

LOC401588 262 399 765 950

LOC401630 55.4 21.5 6.13 13.7

LOC401847 111.95 94.95 100.9 96.95

LOC401913 3.61 5.68 5.45 4.53

LOC401980 3.91 5.95 7.35 4.35

LOC402036 511.45 542.5 531.5 580

LOC402160 4.39 4.92 10.1 9.77

LOC402198 2.93 4.72 4.3 3.57

LOC402360 6260 10500 4440 6440

LOC402382 3.1 4.88 5.71 3.72

LOC402509 250 88 29.8 33

LOC402679 36.4 36.1 41.9 27.8

LOC402779 5.02 4.93 6.68 3.75

LOC407835 8250 2300 3290 4620

LOC414300 3.27 5.26 4.82 3.96

LOC415056 3.12 26.1 4.59 3.73

LOC439911 45.6 75.1 497 85.2

LOC439949 119 9.66 1220 2240

LOC439950 2.57 4.16 3.77 3.17

LOC439990 625 517 527 208

LOC440028 45.7 125 19.1 78.9

LOC440031 3.76 5.94 5.52 4.48

LOC440040 3.4 5.44 5.01 4.14

LOC440043 40000 68200 60300 69300

LOC440047 4.12 4.28 3.87 3.24

LOC440082 61.3 66.3 62.4 84.3

LOC440104 105.266666666667 121.533333333333 251.966666666667 239.466666666667

LOC440117 3.45 5.5 5.15 4.24

LOC440149 111 48.2 49.7 13.5

LOC440173 3.16 5.045 4.71 44.65

LOC440180 3.4 7.99 5.02 4.21

LOC440181 310 241 10.9 486

LOC440292 1470 1380 1630 2560

LOC440300 17.6 12 17.7 46.9

LOC440313 94.2 95 65 76.7

LOC440330 25.9 30.9 52.6 32.7

LOC440335 12.9 8.92 10.6 11

LOC440346 2.77 4.49 4.1 3.39

LOC440356 15.8 75.9 96.5 106

LOC440419 138 92.9 145 162

LOC440461 129 131 122 142

LOC440525 966 1200 751 841

LOC440600 44.8 86.5 8.04 49

LOC440602 2.84 4.57 4.17 3.46

LOC440700 3.36 5.28 5.02 3.97

LOC440704 2.84 4.59 4.2 3.49

LOC440839 214.6 191.15 219.05 257.85

LOC440864 63.9 83.4 62.5 65.8

LOC440896 24.2 23.8 26.7 34.5

LOC440900 19.8 6.35 33.9 11.3

LOC440905 2.64 4.26 155 3.25

LOC440910 11.1 4.46 206 4.96

LOC440925 120 24.55 5.32 4.365

LOC440934 2.58 4.17 5.57 5.72

LOC440944 376 287 525 326

LOC440957 7530 2270 9880 11800

LOC440970 2.63 4.26 3.87 3.25

LOC440983 16.9 24.7 8.48 19.7

LOC440993 53.4 68 97.1 19.4

LOC440995 4.59 5.15 5.68 10.1

LOC441005 3.33 6.32 4.96 6.22

LOC441016 147 166 140 150

LOC441025 24.5 18.5 19.5 174

LOC441046 3.5 4.97 4.51 3.67

LOC441052 2.93 4.72 4.29 149

LOC441177 2.78 4.53 4.13 3.39

LOC441178 10.84 56.12 20.975 7.13

LOC441208 43.2 29.5 80.6 71

LOC441233 5.59 7.26 13.8 8.88

LOC441239 11.8 9.21 7.34 3.51

LOC441242 4.24 6.27 5.97 5.07

LOC441245 159 313 162 158

LOC441268 3.5 14.3 36.1 94.6

LOC441294 258 174 250 81

LOC441455 360 493 561 990

LOC441461 270 79.7 87.6 230

LOC441493 72.5 12.6 5.06 4.12

LOC441528 78.9 69.7 114.2 156

LOC441601 2.67 4.34 3.93 3.27

LOC441617 74.9 85.8 88 114

LOC441644 156 160 113 163

LOC441666 969.18 1025.6 668.4 430.31

LOC441687 6.92 5.29 11.9 36.9

LOC441698 38.2 36.2 40 44.7

LOC441722 2640 2570 5770 6320

LOC441795 2760 4260 1540 12100

LOC441806 10900 17500 17100 12000

LOC442132 62.7 71.195 55.05 74.615

LOC442245 3.55 53.7 4.48 65.8

LOC442249 16300 35900 14500 1950

LOC442283 75.9 40.9 58.1 62.1

LOC442308 3920 3020 1800 2280

LOC442366 30.8 27.6 41.6 35.5

LOC442421 21.48 22.885 27.065 35.8875

LOC442434 2.82 4.57 4.14 3.43

LOC442459 111 110 70.9 108

LOC442497 3.22 5.26 4.79 3.84

LOC442572 159 147 158 232

LOC442676 12.1 4.64 13.6 9.33

LOC474358 3.23 5.13 4.76 3.89

LOC492303 68.1 181 112 184

LOC493754 24500 11100 20500 23900

LOC494150 129000 131000 131000 143000

LOC494558 3.94 6.16 7.85 4.63

LOC497256 169 204 167 166

LOC503519 8.01 7.88 3.97 8.77

LOC51145 3.355 4.79 4.39 16.97

LOC54074 7.63 5.84 5.49 4.49

LOC54082 9.055 6.745 24.115 18.205

LOC541467 22.5 6.21 27.2 14.5

LOC541471 2750 4950 3730 1660

LOC541472 2.91 4.67 4.28 3.52

LOC541473 4.29 16 23.3 19.3

LOC550112 2270 920 748 1360

LOC550643 181 26.9 3780 11700

LOC552889 2180 1540 1410 2120

LOC553103 8.18 6.05 28.9 13.6

LOC553137 41.1 13 4.32 3.55

LOC554174 2.83 4.57 4.18 3.49

LOC554201 3.81 6.09 5.67 4.6

LOC554202 2.84 4.63 1000 67.1

LOC554203 3.44 5.6 13.4 57.8

LOC554223 8.84 9 8.69 9.455

LOC55908 4620 139 11.1 4.5

LOC572558 9.43 6.18 11.4 17.9

LOC57399 6.59 5.62 5.31 4.42

LOC574538 2.93 4.73 4.3 3.58

LOC606724 37.2 27.1 42.3 40.3

LOC613126 2.81 4.54 4.13 3.99

LOC613266 3.09 4.95 7 14.3

LOC619207 18.435 33.7 27.5 24.145

LOC63930 3.33 5.41 4.95 3.94

LOC641364 3.24 5.26 4.8 3.95

LOC641365 5.11 7.44 5.45 4.46

LOC641467 3.72 5.94 5.46 4.37

LOC641510 26.1 15.2 163 87.6

LOC641515 17.1 17.1 25.8 16.8

LOC641518 10.7 6.21 5.79 21.4

LOC642031 1570 761 831 1880

LOC642127 6.36 5.79 5.67 4.87

LOC642278 23.3 30 46.7 29.6

LOC642288 9.03 5.71 15.4 33.8

LOC642335 55.8 50.2 36.8 58.5

LOC642340 19.4 5.48 49.2 17.4

LOC642350 62.7 26.6 37 17.7

LOC642361 1770 1390 3100 3740

LOC642366 123 12.8 190 230

LOC642384 28.6 37.1 53.4 49

LOC642406 42.4 59 77.6 89.1

LOC642413 206 1510 989 1070

LOC642423 29.1 28.9 35.6 34.5

LOC642426 18.6 10.6 22.5 16.3

LOC642513 926 1130 2730 1350

LOC642515 178 173 141 160

LOC642521 876 1440 848 803

LOC642574 45.4 38.8 53.7 52.7

LOC642587 2.79 4.51 23.6 3.44

LOC642597 3.09 4.8 4.58 3.68

LOC642622 5.63 5.78 14 8.76

LOC642635 8.7 19.2 14.2 28.5

LOC642648 33.8 37.4 42.9 40.8

LOC642666 16.9 19.1 25.1 23.5

LOC642776 21.4 14.9 14.3 19.6

LOC642781 17.8 10.7 6.52 10.4

LOC642808 3.25 5.28 23.6 47.8

LOC642826 617 469 367 527

LOC642852 168.8 143.66 333.83 422.85

LOC642864 3.59 5.78 6.44 4.23

LOC642891 4.66 4.87 4.85 3.71

LOC642924 3.04 15.8 12.8 3.44

LOC642947 35.2 42.6 52.9 46.4

LOC642980 36.8 53.5 39 63.9

LOC643008 12.85 10.095 386.35 6.88

LOC643037 72.1 5.49 8.65 4.95

LOC643072 25.5 30.8 30.5 32.1

LOC643153 4.09 6.26 5.93 5.14

LOC643201 10.9 10.9 25.8 19.7

LOC643327 14.8 4.76 10.2 40.3

LOC643355 21.4 39 31.7 28

LOC643368 4.65 4.18 44 5.32

LOC643371 668 1340 1060 626

LOC643401 54.1 5.37 4.94 116

LOC643406 37.4 158 6.02 5.54

LOC643441 9.31 5.71 5.27 9.45

LOC643454 1960 1950 3980 2770

LOC643475 20.7 5.13 25.4 17.2

LOC643542 2.6 4.2 3.82 3.2

LOC643551 61 21 48.8 8.65

LOC643563 22900 24200 11200 18200

LOC643623 2.69 4.37 3.96 3.3

LOC643669 17.9 23.4 33.4 37.9

LOC643719 36.05 40.15 37.8 47.1

LOC643723 4.49 4.11 3.73 11.9

LOC643733 2.65 4.28 3.9 5.78

LOC643763 4.48 6.33 7.32 4.88

LOC643770 9 5.65 17.2 13.6

LOC643783 192 307 67.3 63.1

LOC643802 342 341 112 295

LOC643837 373 331 267 294

LOC643873 1270 1280 1130 1830

LOC643923 2.88 4.66 4.23 190

LOC643962 3.48 5.53 5.22 4.39

LOC643972 2.84 4.57 4.17 3.46

LOC643988 140 143 1610 914

LOC643997 83400 54900 77900 51400

LOC644021 581 1410 853 1000

LOC644070 7.91 6.68 7.61 7.3

LOC644075 55.8 52.3 59.4 42.8

LOC644100 3.1 5.05 4.6 3.77

LOC644135 2.9 4.7 4.26 3.55

LOC644173 52.8 83.8 101 102

LOC644189 1090 410 8.97 11.3

LOC644192 25.2 23.5 122 33.5

LOC644193 115 144 123 127

LOC644196 131 71.1 101 128

LOC644213 65.3 84.5 74.1 41

LOC644231 1540 1550 1680 3060

LOC644242 20.8 20.7 24.2 8.87

LOC644246 490 47.6 153 65

LOC644248 3.82 5.91 5.76 4.94

LOC644265 14.4 4.71 4.37 3.59

LOC644277 552 570 624 507

LOC644280 106 112 112 138

LOC644285 8400 9280 14200 13400

LOC644310 1710 4340 4480 8340

LOC644334 27.9 44.2 27.7 20.2

LOC644366 3.95 6.22 5.7 4.63

LOC644422 257 277 183 272

LOC644450 441 811 220 913

LOC644525 61.3 58.4 79.6 160

LOC644538 239 273 4.75 775

LOC644548 26.6 48.4 30.5 29.6

LOC644554 5.29 4.78 4.48 3.66

LOC644563 26100 15700 23500 25600

LOC644578 3.23 5.23 4.81 3.84

LOC644587 8.9 8.95 65.1 52

LOC644613 181 69.1 248 371

LOC644620 6.82 8.28 4.74 3.78

LOC644649 25.5 18.9 24.9 19.7

LOC644662 3.54 5.73 47.8 4.32

LOC644686 175 209 221 128

LOC644717 9.87 53.7 7.47 8.5

LOC644727 733 237 443 884

LOC644794 41.1 43.8 27.1 46.1

LOC644841 3.795 5.64 5.205 6.455

LOC644852 3.56 5.73 5.3 6.16

LOC644893 26 33.9 19.2 24.7

LOC644919 2.54 51.7 3.73 48

LOC644925 27 36.3 57.3 52.1

LOC644949 24.2 14 30.9 17.9

LOC644961 115000 109000 127000 123000

LOC644962 1510 1510 977 1300

LOC644990 80.5 14.2 66.4 16.9

LOC644992 74.1 286 518 290

LOC645101 4.75 5.34 4.88 4

LOC645146 583.5 73 9.185 40

LOC645158 20.9 13.2 7.58 17.3

LOC645166 30600 19000 2690 2710

LOC645177 3.3 5.38 4.89 3.96

LOC645188 2.9 4.68 4.26 3.54

LOC645212 276 323 458 266

LOC645225 41.6 60.9 46.7 55.1

LOC645249 1110 191 1270 1700

LOC645261 1790 2110 2510 1680

LOC645277 51.8 7.09 12 21.5

LOC645307 2.93 4.735 13.88 7.68

LOC645314 181 194 170 189

LOC645321 7.3 17.2 11.6 76.2

LOC645323 5.445 5.005 38.745 61.435

LOC645332 68.8 137 234 170

LOC645339 1710 1910 1940 2300

LOC645390 41.4 54.1 41.6 44.7

LOC645431 13.7 138 58.4 47.9

LOC645435 134.266666666667 137.666666666667 133.966666666667 122.766666666667

LOC645446 3.72 8.15 148 19.1

LOC645485 51.5 4.68 4.3 3.58

LOC645534 342 194 420 346

LOC645553 10300 10300 4950 11100

LOC645586 75.3 96.1 199 81.5

LOC645591 3.59 5.61 11.3 4.66

LOC645602 62.1 54.6 25.3 38.8

LOC645605 9.06 25.4 20.7 10.6

LOC645634 35.6 45.4 50.3 33.2

LOC645645 173 162 137 209

LOC645676 783.5 716.5 1055.5 1575

LOC645685 33.8 37.2 40.5 46.2

LOC645722 1800 565 317 1480

LOC645744 40.3 21.8 24.5 71.8

LOC645752 60.965 71.19 72.32 119.615

LOC645769 18 14 22.4 22.1

LOC645781 517 672 431 488

LOC645851 31 71 69.6 65.8

LOC645877 8.01 5.1 7.03 5.21

LOC645937 10261 12170 14780 22450

LOC645944 66.8 5.85 5.68 4.82

LOC645949 3.21 5.24 4.75 3.86

LOC645955 1300 2340 1570 1560

LOC645961 2.88 4.15 6.41 3.16

LOC645967 33.6 27.2 88.3 64.3

LOC645971 48.1 45.2 80.7 113

LOC645978 70.6 20.8 48.7 53.6

LOC645984 3.1 5.06 4.6 3.71

LOC646014 113 128 130 178

LOC646034 13 21.1 13.1 10.7

LOC646048 2640 2230 1930 1160

LOC646049 1190 4310 7700 3800

LOC646079 18.4 21.1 14.3 12

LOC646085 9.06 14.4 35.2 4.96

LOC646139 6.32 17.5 7.94 9.5

LOC646160 2.98 4.71 23.4 34.8

LOC646168 3.62 5.86 5.34 4.25

LOC646201 146 89.2 92.5 85.9

LOC646214 1780 2860 1730 884

LOC646241 3.7 5.88 5.45 18.8

LOC646262 79 81.2 62.4 68

LOC646324 3.88 6.11 5.65 4.56

LOC646344 2.71 8.84 12.7 4.85

LOC646377 2.83 4.61 4.18 3.44

LOC646396 8.73 7.72 17.8 16.2

LOC646470 8.92 46.3 35.8 5.12

LOC646471 18.6 14.4 17.9 22.6

LOC646482 9.59 4.72 4.29 8.72

LOC646498 3.16 9.08 5 15

LOC646508 4.49 4.93 7.62 3.75

LOC646509 17.7 25 81 50

LOC646522 3.44 5.54 5.08 4.08

LOC646543 9.46 13.3 6.3 19.6

LOC646548 7 6.23 5.79 9.49

LOC646576 9.15 18.6 4.65 18.8

LOC646588 2.64 4.27 3.87 3.24

LOC646626 48 221 96.9 243

LOC646627 3.77 6.04 5.53 4.42

LOC646629 4.26 6.4 6.09 5.16

LOC646698 8.17 7.14 9.06 9.49

LOC646709 198 160 144 105

LOC646719 27 19.4 27.4 49.4

LOC646730 2.85 4.64 7.2 16.9

LOC646736 2.94 4.7 4.29 3.56

LOC646762 630 726 1030 1930

LOC646791 8220 12000 10400 15700

LOC646804 3.82 6.09 5.75 8.12

LOC646808 2010 7450 1400 10900

LOC646813 21.9 16.4 8.79 16.58

LOC646821 54800 46700 68000 50600

LOC646853 24.4 69.1 114 82.4

LOC646862 62.8 46.8 40.8 53.2

LOC646870 22 15.5 15 20.9

LOC646879 3.58 5.71 5.52 4.22

LOC646890 5640 4980 8130 3970

LOC646891 30.5 25.5 16.9 30.2

LOC646934 3.14 5.34 4.66 3.74

LOC646936 5.17 56.8 154 195

LOC646960 6.21 45.2 7.65 95

LOC646976 14.3 5.78 82.5 15.6

LOC646982 3.735 4.41 4.005 3.33

LOC646993 283 413 130 1020

LOC646999 22.6 208 4.81 53.4

LOC647012 3.48 5.66 5.16 4.1

LOC647020 3.8 6.08 5.59 4.48

LOC647055 51.5 56.9 62.3 52.8

LOC647070 5840 6410 6420 6630

LOC647086 7520 14900 8050 13500

LOC647107 3.49 42.9 4.29 3.55

LOC647166 4.355 5.35 4.955 4.1

LOC647169 2.9 18.8 4.25 3.53

LOC647188 179 120 152 253

LOC647281 11.545 17.02 17.115 10.975

LOC647286 3.95 6.15 5.75 4.7

LOC647302 15200 13300 13600 12100

LOC647309 8.9 10.4 9.07 11.2

LOC647310 11.1 5.08 16.2 14.5

LOC647323 4 8.33 5.76 4.7

LOC647343 6.34 9.495 9.995 7.32

LOC647546 3.86 6.13 5.83 4.88

LOC647946 42.7 86.9 8.37 178

LOC647979 981 1300 1228.5 1430

LOC647983 20.5 17.75 25.95 21.65

LOC648044 76.1 71.9 74.6 74.1

LOC648149 81.9 46 4.54 3.76

LOC648262 600.77 601.01 644.466666666667 548.976666666667

LOC648556 88.4 39.8 185 148

LOC648570 32 24 46.7 44.3

LOC648691 28.5 12.6 18 17.7

LOC648740 117000 20000 20400 16800

LOC648771 120000 114000 119000 155000

LOC648809 2.92 21.4 10.5 28.9

LOC648822 27.3 72.5 29.4 36.9

LOC648987 396 636 704 719

LOC649034 144 97.3 46.3 39.6

LOC649133 3.22 5.22 4.79 3.83

LOC649201 32.8 34.5 22.9 38.1

LOC649238 3.83 5.98 7.45 4.96

LOC649264 2.65 4.3 18.6 3.26

LOC649294 29.925 33.125 42.57 40.12

LOC649305 220 184 189 226

LOC649346 3.25 5.28 8.16 12.5

LOC649395 685 764 1110 1440

LOC649458 3.24 5.19 4.78 3.92

LOC649489 2.55 4.12 3.74 3.14

LOC649660 9.2 4.53 4.76 9.59

LOC649873 76800 53300 66200 43800

LOC649941 36.3 56.7 34.5 33.2

LOC649956 4.12 6.39 6.02 4.97

LOC650095 28 4.34 3.95 3.31

LOC650157 905 477 350 232

LOC650177 3.71 5.93 27.4 7.09

LOC650262 58.6 47.8 63.7 84.8

LOC650293 7.875 11.48 8.135 10.93

LOC650392 24.1 12.9 36.3 444

LOC650457 51.5 63.7 34.7 49.6

LOC650621 31 24 26 34

LOC650638 1130 985 711 860

LOC650794 9.7 4.52 83.3 12.7

LOC651337 27.9 24.1 27.7 22.3

LOC651536 51.3 44.55 45.35 46.5

LOC651581 3.42 15.3 6.88 8.96

LOC651721 3.57 5.79 5.3 4.19

LOC651845 6.48 5.54 5.07 4.15

LOC651868 7.73 13.5 11.9 13.5

LOC651986 78.15 53.7 54.85 44.4

LOC652022 17.2 17.4 35.4 16.2

LOC652119 551 607 403 473

LOC652147 454 463 324 426

LOC652191 3980 3040 2170 3910

LOC652215 2.55 4.13 3.74 3.14

LOC652494 14.5 20.5 13.9 21

LOC652554 38.9 54.9 49.8 29.8

LOC652586 161 140 115 110

LOC652614 11.8 10.3 18.4 24

LOC652636 176 174 113 108

LOC652797 5.4 11.6 13.8 9.3

LOC652859 4.03 6.22 5.77 4.73

LOC652875 35.1 16.5 22.2 14.2

LOC652990 78.2 117 134 146

LOC653056 1750 2040 1540 2430

LOC653113 123 359 657 435

LOC653390 8.18 16.1 19.4 17.9

LOC653391 497 844 252 669

LOC653510 206 44.2 75.7 143

LOC653602 2.81 4.51 4.12 3.44

LOC653653 19 34.7 34.6 88.6

LOC653707 4.03 6.33 5.94 4.86

LOC653712 19.2 16.8 32.4 28.2

LOC654056 410 450 424 456

LOC654433 3.83 1060 5.62 4.49

LOC654779 21 16.8 7 17.6

LOC654780 4.57 5.75 6.2 5

LOC654841 2.62 4.23 3.85 7.28

LOC678655 35.5 37.2 52.5 42.6

LOC723805 5.71 6.26 23 5.25

LOC723809 25.8 9.2 721 18.9

LOC727677 3.21 6.34 4.77 4.74

LOC727710 2.57 4.16 3.77 3.16

LOC727721 94.7 71.5 65.9 65.4

LOC727775 3.11 5.08 4.63 3.75

LOC727788 318 377 364 293

LOC727796 2260 1340 2120 3190

LOC727804 519 572 611 988

LOC727808 28.5 25.8 38.6 19.8

LOC727818 10.8 9.68 12.3 7.23

LOC727838 5.74 5.81 5.35 4.39

LOC727844 14.2 9.5 18 11.6

LOC727845 128 53.9 290 232

LOC727847 102 71.3 71.8 21

LOC727869 26.3 33.2 28.4 11.9

LOC727872 36.4 35.4 17.6 23.3

LOC727878 141 89.4 108 87.6

LOC727894 122 132 109 89.5

LOC727900 86 101 149 107

LOC727915 2.9 4.71 4.27 3.52

LOC727916 85.3 18.5 45.2 29.4

LOC727918 11.1 11.5 5.46 11

LOC727924 3.38 5.41 5.03 4.26

LOC727927 3.71 5.9 5.6 4.57

LOC727934 10.1 10.5 6.24 8.25

LOC727938 6.8 6.23 4.61 14.1

LOC727941 3.97 6.2 5.73 4.65

LOC727962 11.6 7.76 9.84 15.5

LOC727969 42 50.8 39.7 40.6

LOC727973 3.44 5.58 17.3 4.2

LOC727982 6.68 9.93 13.5 9.3

LOC727993 88.8 105 84.6 91.7

LOC728027 3.05 15 53.4 3.65

LOC728046 2.92 4.76 8.06 3.99

LOC728052 6.59 4.12 5.31 7.39

LOC728056 4.23 6.36 6.06 5.15

LOC728064 3.32 41.5 7.6 16.4

LOC728065 2.97 4.78 4.39 3.65

LOC728073 13.2 11.5 7.16 18.4

LOC728084 3.29 5.35 5.59 3.92

LOC728086 3.91 4.71 4.35 6.42

LOC728095 3.01 4.81 4.4 3.63

LOC728099 4.74 7.64 4.58 8.85

LOC728114 3.49 5.63 5.21 4.24

LOC728129 80.8 96.3 34.6 106

LOC728142 36.8 42.1 108 56.6

LOC728145 40.7 16.9 23.6 33.4

LOC728147 46.1 5.56 169 97.6

LOC728148 2.59 4.2 3.8 3.19

LOC728152 4.78 8.71 9.18 4.06

LOC728163 64.5 61.6 61.9 69.5

LOC728175 9.43 4.845 4.4 3.62

LOC728178 39 36 105 53.4

LOC728190 510 712 301 679

LOC728192 3.15 5.02 4.64 3.81

LOC728196 3.05 4.91 4.47 3.67

LOC728208 9.175 9.77 10.395 10.875

LOC728211 8.18 5.46 5.075 11.49

LOC728217 3150 1450 1650 1740

LOC728218 7.37 90.2 5.75 4.77

LOC728254 29.2 33.7 25.8 32.8

LOC728264 67.3 69.1 54.1 45.7

LOC728276 17.905 8.25 8.185 7.105

LOC728288 3.06 4.89 4.49 6.28

LOC728290 2.86 17.1 4.21 3.48

LOC728297 27 27.8 42.8 55

LOC728316 3.99 10.8 7.52 4.43

LOC728317 14.54 22.225 13.32 12.28

LOC728323 8790 3410 3330 2920

LOC728324 32000 14500 16700 17400

LOC728339 12.3 6.25 5.88 4.83

LOC728342 3.4 5.47 5.08 7.3

LOC728347 2.84 12.8 4.19 8.13

LOC728353 15.2 7.68 43.9 75.2

LOC728361 7.07 7.9 17.8 7.77

LOC728392 247 855 3.76 1630

LOC728411 529.25 509.75 484.2 787.5

LOC728416 13800 30000 27900 25900

LOC728417 133.133333333333 142.533333333333 108.6 199.466666666667

LOC728431 59.4 71.4 59.3 26.9

LOC728448 35.83 107.235 106 211.95

LOC728449 1854.85 2449 1386.5 1806.15

LOC728460 3.47 5.63 5.16 4.22

LOC728463 14.7 62.2 5.85 6.42

LOC728470 2.72 4.41 3.99 3.34

LOC728500 3.47 5.63 6.43 8.08

LOC728510 2.92 4.64 4.26 3.54

LOC728528 16.5 5.38 16.5 13.9

LOC728530 11.7 12.4 18.1 12.9

LOC728534 148 202 313 243

LOC728537 109 158 179 245

LOC728543 3.9 6.17 5.77 12.7

LOC728558 32.5 25.9 4.56 63.2

LOC728573 3.05 4.92 4.52 3.73

LOC728588 50.7 43.3 55.4 51.3

LOC728606 3.42 5.53 5.08 4.16

LOC728613 282 32.7 46.7 158

LOC728627 44.6 51.5 51.4 51.1

LOC728650 36.525 63.6 216.45 551

LOC728671 8.25 7.58 11.5 5.97

LOC728675 992 1060 764 407

LOC728684 28.5 33 36 42.8

LOC728694 15.4 9.8 20.8 13.3

LOC728698 18900 24900 19200 23300

LOC728701 919.386666666667 867.396666666667 688.743333333333 738.6

LOC728705 39.7 71.8 155 80.9

LOC728723 25.4 21.2 92.7 154

LOC728735 13.4 11 15.4 15.8

LOC728739 1630 3220 1360 3380

LOC728741 12800 10500 17000 15200

LOC728752 13.8 5.57 11.7 15

LOC728755 3.74 18.7 5.61 4.61

LOC728767 3.44 5.54 5.09 4.09

LOC728769 368 499 1150 906

LOC728789 3.34 5.31 4.95 4.02

LOC728792 11.1 16.4 10.4 16

LOC728800 2.56 4.15 5.55 3.16

LOC728805 16.1 5.5 5.02 4.05

LOC728806 90 33.6 98.3 127

LOC728809 55 135 56.5 111

LOC728811 10.4 6.19 5.66 4.56

LOC728816 3.21 5.22 4.78 3.83

LOC728836 57.2 31.6 21.5 25.8

LOC728846 38763.8 45358.85 44260.4 46522.85

LOC728853 3.85 6.17 5.8 4.81

LOC728857 31.9 199 134 55.5

LOC728868 2.73 4.42 4.02 3.34

LOC728875 5445 8530 1895 5775

LOC728903 56.45 14.95 517 1137.5

LOC728927 502 390 232 210

LOC728940 3.07 5.01 4.55 3.7

LOC728965 18 21.9 30 30.7

LOC728975 348 24.4 346 81.5

LOC728991 3.5 5.66 5.17 4.14

LOC729009 213000 174000 189000 57500

LOC729013 133 804 963 642

LOC729032 28.5 24.7 50.8 42.1

LOC729040 9.36 24 11.4 13.7

LOC729041 41 48.7 66.5 261

LOC729046 33300 49700 41800 37000

LOC729047 3.55 4.76 4.38 45

LOC729051 3.24 5.3 34.8 3.9

LOC729059 7.33 5.89 17.5 28.6

LOC729061 13.8 13.95 14.6 16.6

LOC729065 3.85 4.69 4.26 3.49

LOC729069 4.68 4.58 9.08 5.25

LOC729076 2.98 44 4.4 3.59

LOC729078 3.41 5.46 11.2 9.1

LOC729081 7.13 10.4 10.8 5.07

LOC729082 1990 1670 1450 1130

LOC729083 4.06 6.22 22.7 4.79

LOC729088 191 160 52.7 338

LOC729113 3.03 4.95 19.4 14.2

LOC729121 23.2 5.64 5.22 4.25

LOC729130 62 39.3 59.3 57.2

LOC729141 9.54 8.66 9.77 9.14

LOC729142 9.65 6.82 9.17 14.1

LOC729153 14.3 12.9 6.08 6.93

LOC729154 6.84 4.17 11.8 7.24

LOC729159 3.27 5.21 4.755 3.905

LOC729175 85.3 94.4 112 119

LOC729176 3.2 7.36 26.9 3.84

LOC729177 2.97 4.82 4.38 3.63

LOC729178 23 18.8 16.7 22.8

LOC729187 3.16 4.96 4.68 3.75

LOC729189 57.4 57.5 60.7 46.2

LOC729199 42.3 40.4 71.5 57.4

LOC729204 15.3 8.78 10.5 13.5

LOC729213 2.58 4.18 3.79 3.18

LOC729218 376 427 268 563

LOC729225 18.3 16.9 31.6 54.6

LOC729242 15 15.6033333333333 13.4333333333333 19.12

LOC729249 5.61 12.2 6.04 16.8

LOC729251 2.69 4.35 3.97 7.28

LOC729255 47300 105000 90400 79200

LOC729289 3.26 5.19 4.76 3.91

LOC729291 42.5 28.7 175 55.2

LOC729296 3.17 5.17 4.72 3.84

LOC729305 131 101 89.4 99.7

LOC729307 3.49 5.66 5.23 4.22

LOC729312 21.4 22.1 21.6 24.6

LOC729313 110000 66700 93700 61600

LOC729314 78.4 90.3 79 71.3

LOC729327 2.7 4.38 3.97 3.38

LOC729338 3.95 16 7.17 42.9

LOC729350 63 133 28.1 55.3

LOC729370 41.8 31.4 34.1 26.4

LOC729378 76.5 38.1 43.5 40.3

LOC729384 3.08 5.02 4.57 4.24

LOC729385 128 13.1 4.25 3.53

LOC729390 3.14 5.075 4.635 3.765

LOC729409 26.9 7.27 5.43 24.2

LOC729412 21.31 17.165 13.98 16.87

LOC729417 24.8 146 23.6 39

LOC729421 92.3 43.6 201 76

LOC729424 54.4 60.8 67.5 63.6

LOC729426 4.94 4.14 4.73 3.15

LOC729451 6.12 6.13 5.7 5.52

LOC729454 77.9 410 138 108

LOC729461 3.77 117 5.68 848

LOC729467 4.02 5.08 95.2 13.1

LOC729479 7.04 4.9 4.48 3.66

LOC729492 3.17 5.16 4.72 3.84

LOC729494 3.74 4.72 5.51 3.61

LOC729506 12.9 4.41 6.06 78.7

LOC729519 113 123 141 121

LOC729526 3.33 5.35 4.97 4.02

LOC729537 109 84.1 67.8 67.6

LOC729549 9.83 15.1 16.2 18

LOC729558 73.05 86.55 49.2 75.45

LOC729570 208 210 386 311

LOC729580 2250 2260 2950 3240

LOC729590 81500 93200 90100 97400

LOC729595 473 776 328 2350

LOC729601 3.64 5.63 5.15 4.99

LOC729602 227 336 384 385

LOC729603 147 103 23.5 76.8

LOC729609 2710 3000 3150 3110

LOC729626 32.1 15.4 12.7 10.6

LOC729645 331 350 285 356

LOC729652 2.8 4.51 7.11 7.8

LOC729658 3.15 13.6 4.68 3.76

LOC729668 1730 1730 1270 3290

LOC729669 3.38 5.6 5.01 4.08

LOC729678 222 508 5.4 133

LOC729680 291 112 150 1130

LOC729681 4.34 4.82 4.45 8.98

LOC729683 60.8 114 48.7 38

LOC729686 224 365 224 252

LOC729687 1240 4400 8100 4210

LOC729696 20 12.2 21.5 19.3

LOC729706 6.71 16.3 6.36 12.1

LOC729723 25.9 21.1 26.6 19.6

LOC729739 14.3 62.9 13.5 12.2

LOC729756 2.86 4.66 4.22 3.48

LOC729770 385 262 333 177

LOC729799 338 318 584 419

LOC729800 3.57 5.68 13.9 5.06

LOC729806 840 411 536 200

LOC729815 3.83 6.15 5.77 4.75

LOC729822 16.6 28.6 653 62.1

LOC729832 3.74 5.18 5.21 3.61

LOC729839 126 435 481 313

LOC729841 17600 25400 28200 20800

LOC729856 131 162 176 176

LOC729860 2.9 4.71 4.27 1130

LOC729867 4.95 6.1 20 58.7

LOC729870 17.6 54.5 85.2 43.9

LOC729879 29 21.2 27.1 35.9

LOC729885 8.31 6.02 89.5 4.55

LOC729887 766 400 5000 1090

LOC729897 29.2 24.5 44.6 45.1

LOC729907 91.9666666666667 73.4666666666667 60.3666666666667 54.2666666666667

LOC729911 3.51 7.5 19.7 24.1

LOC729915 88.3 111 85.9 64.8

LOC729930 5.93 5.53 5.09 4.2

LOC729933 2.88 4.63 4.23 3.51

LOC729950 3.48 4.17 3.78 4.4

LOC729968 3.26 5.3 4.84 3.97

LOC729970 188 297 77.9 171

LOC729977 5.89 4.99 4.59 3.79

LOC729978 976 626 978 873

LOC729983 8410 4780 8190 3430

LOC729986 3.88 6.07 5.66 4.6

LOC729987 29.5 15 10.1 4.8

LOC729989 3.65 40.8 26.7 4.51

LOC729991 458.333333333333 781.666666666667 498.333333333333 689.666666666667

LOC730007 3.89 6.15 5.63 4.58

LOC730011 3.18 5.18 4.73 3.85

LOC730020 9.92 36.9 38.5 46.6

LOC730032 18.9 16.5 21.8 10.6

LOC730045 3.28 5.29 4.89 14

LOC730058 6.49 12.3 21.2 27.3

LOC730060 76.8 56.2 86 43.4

LOC730078 3.61 5.76 5.32 4.3

LOC730081 6.54 5.42 12.1 12.6

LOC730085 115 29.5 5.78 166

LOC730091 28.2 23.3 4.24 3.5

LOC730092 177 152 417 181

LOC730098 45.1 193 254 448

LOC730100 3.18 5.19 4.72 3.79

LOC730101 143 210 45.2 68.6

LOC730107 2270 1950 2040 2430

LOC730109 8.61 5.25 4.87 7.16

LOC730121 3.15 11.6 4.69 3.86

LOC730129 354 428 263 286

LOC730132 85.3 22.9 34 25

LOC730134 3.8 5.97 5.72 4.78

LOC730139 6.3 9.46 14.1 5.46

LOC730144 21600 28600 20300 23300

LOC730152 9.36 59 4.59 3.75

LOC730159 2.95 20.5 4.29 3.55

LOC730167 541 429 108 106

LOC730179 4.04 14.7 4.54 3.73

LOC730183 295 132 923 1630

LOC730184 7.6 11 9.32 7.95

LOC730198 5.02 6.05 5.62 4.44

LOC730202 104 97.3 276 137

LOC730227 20.3 26.7 38.6 27.3

LOC730232 16 22.1 9.03 11.1

LOC730236 41.9 71.1 145 274

LOC730242 2.73 23.7 4.07 3.33

LOC730254 3.66 5.88 5.48 4.45

LOC730256 1580 955 498 565

LOC730351 6.64 4.81 8.52 12.6

LOC730375 3635 3430 1955 3635

LOC730413 1610 5110 1410 4690

LOC730441 2.98 4.77 4.35 3.6

LOC730456 3.62 5.81 5.4 4.38

LOC730474 6.67 11.5 6.21 9.15

LOC730495 12 16 23.1 23.7

LOC730631 94.1 127 160 101

LOC730651 10.6 10.1 11.1 23.8

LOC730668 3.63 5.64 5.47 4.59

LOC730811 4.13 6.32 5.96 4.94

LOC730883 61 148 86.2 308

LOC730961 14.9 21.8 89.8 38.2

LOC730974 13.9 4.9 4.51 3.71

LOC730978 62.2 72.1 77.2 68.4

LOC731039 3.04 4.81 4.47 3.67

LOC731109 10.9 6.33 5.95 5.55

LOC731139 201 220 157 1500

LOC731223 314 332 629 482

LOC731275 324.98 410.4 464.6 504.6

LOC731282 28.3 77.2 26.8 54.2

LOC731312 8.02 6.66 13.9 9.33

LOC731414 3.28 5.36 4.88 3.9

LOC731419 54.6 68.6 155 93.7

LOC731424 3.16 5.15 6.27 3.83

LOC731656 3.53 13.6 5.29 4.3

LOC731779 9.75 5.37 6.66 4.06

LOC731789 10.435 7.05 6.025 6.515

LOC731852 7.84 17.8 19 6.68

LOC731932 188 192 157 187

LOC731957 3.69 5.79 7.31 6.28

LOC732043 15.9 14 27.9 23.9

LOC732096 8.23 5.6 18.4 7.64

LOC732146 15.8 11 23.9 17.3

LOC732160 264 226 230 269

LOC732272 875 106 3.95 3.3

LOC732275 18.2 10.1 20.2 14.8

LOC732419 286 399 355 302

LOC732435 66.3 5.76 11.3 3.94

LOC732443 8.57 6.07 12.3 16.5

LOC732455 2.66 4.31 38.6 3.27

LOC780529 6.04 6.11 5.7 16.8

LOC80054 75.1 120 18.5 74.7

LOC80154 1417.4 2993 1782.66666666667 5387.66666666667

LOC81691 261 173 345 244

LOC84740 3.657 5.336 4.914 4.024

LOC84856 10.9 12.1 344 222

LOC84931 192 220 210 207

LOC84989 102 206 441 199

LOC90110 506 369 636 2300

LOC90246 159 150 42.8 64.4

LOC90586 15.1 11.7 6.45 13.7

LOC90784 723 1090 439 1120

LOC90834 21.6 29.6 9.74 35.6

LOC91149 3.29 8.18 3.78 3.16

LOC91316 2315 3075.5 1324 2520.5

LOC91450 52.4 325 118 258

LOC91548 260 346 216 208

LOC91948 1400 1190 822 445

LOC92249 69.5 96.8 97.2 203

LOC92659 126 143 309 151.5

LOC92973 2.62 4.23 10.9 10

LOC93432 5.76 9.92 5.92 3.68

LOC93444 6.49 6.1 8.87 7.26

LOC93463 2.89 4.7 4.26 3.54

LOC93622 2000 5070 2910 6700

LOC96610 72.95 261.5 35.55 195.5

LOH12CR1 393 504 1070 660

LOH12CR2 51.45 49.55 136.5 83.05

LOH3CR2A 69.6 130 58.5 10.6

LONP1 3790 927 2380 2340

LONP2 276 288 414 355

LONRF1 38.4 333 72.6 148

LONRF2 12.55 5.465 25.95 703.5

LONRF3 466.545454545455 842 135.518181818182 600

LOR 3.79 5.83 25.3 4.95

LOX 285 1021.9 9.545 155.5

LOXHD1 4.02 6.225 7.79 4.745

LOXL1 7.99 7.17 5.57 182

LOXL2 43.85 696.8 1160 251.2

LOXL3 44.5 99.05 399.5 829.5

LOXL4 1570 826 6.05 32.1

LPA 24.7 25.35 26.65 31.2

LPAL2 2.56 4.14 3.75 3.15

LPAR1 4.93 6.18 207 157

LPAR2 115 68.2 78 67.9

LPAR3 19.1 148 3.73 150

LPAR4 3.06 4.99 4.54 3.67

LPAR5 3.72 5.69 5.62 4.95

LPAR6 119 501 5.59 49.4

LPCAT1 238 189 596 578

LPCAT2 3.02 4.91 320 249

LPCAT3 6520 6150 11800 5710

LPCAT4 84.9 143 330 113

LPGAT1 8090 4890 1770 3470

LPHN1 263 207 584 1110

LPHN2 977.175 2237 2333.5 1067.675

LPHN3 349.709090909091 67.0909090909091 64.9472727272727 356.163636363636

LPIN1 380.2 1117.1 81.95 684.1

LPIN2 1750 2140 650 374

LPIN3 1400 1450 1880 2360

LPL 3.98 6.27 5.87 75.7

LPO 18.9 18.1 28.8 13.9

LPP 666 639 649 110

LPPR2 122 215 349 346

LPPR3 20.7 33.7 12.6 102

LPPR4 68.5 32.25 43.65 58.1

LPPR5 24.7 6.09 5.61 4.52

LPXN 65.2 34.1 145 53.8

LQK1 236 203 636 212

LRAT 17.7 4.59 5.57 14.2

LRBA 879 718 945 1370

LRCH1 43.8 180.75 118.1 95.45

LRCH2 11.8 15.7 8.33 912

LRCH3 54.6 86.45 73.45 74.1

LRCH4 3240 3520 2550 2390

LRDD 2800 1330 2120 3490

LRFN1 96.6 217 207 898

LRFN2 6.27 15.5 14.1 17

LRFN3 2070 1630 2750 1840

LRFN4 69.55 74.75 5535 3595

LRFN5 10.6 20.2 4.45 9.99

LRG1 3660 664 344 126

LRGUK 9.39 6.61 13 18.8

LRIG1 1596 1154 645.3 1808

LRIG2 182 230 261 502

LRIG3 1050 1430 406 1260

LRIT1 11.3 14.5 9.27 11.8

LRIT2 2.93 4.76 4.31 3.55

LRIT3 8.8 4.41 4 10.8

LRMP 85.8 181 25.8 18.1

LRP1 189.181818181818 494.545454545455 75.5454545454545 43.2090909090909

LRP10 346 448 600 162

LRP11 234 110 218 142

LRP12 253 519 436 391

LRP1B 62.1 149 8.3 116

LRP2 663 721 686 717

LRP2BP 2.8 4.53 4.11 3.42

LRP3 4955.6 6320 3268.6 5867.5

LRP4 580 1420 87.9 1040

LRP5 3360 1690 498 488

LRP5L 432.5 224.25 94.3 148

LRP6 1910 1940 2850 1620

LRP8 660 1600 2170 1020

LRPAP1 2570 2830 1270 2960

LRPPRC 3726.33333333333 2841.33333333333 3602.33333333333 2774.66666666667

LRRC1 1090.12666666667 566.193333333333 372.4 411.403333333333

LRRC10 69.8 74.3 88.9 60.6

LRRC10B 3.195 5.19 4.73 3.9

LRRC14 1970 1090 1530 3830

LRRC14B 8.12 5.67 5.23 8.49

LRRC15 4.73 5.99 5.7 4.73

LRRC16A 143.6 44.45 98.5 92.35

LRRC16B 57.7 53.1 58.4 70.2

LRRC17 7.345 35.25 5.885 66

LRRC18 18.3 19.4 23 23.5

LRRC19 8.72 10.4 358 3.85

LRRC2 38.9 51.565 13.75 20.385

LRRC20 1560 4380 4910 2220

LRRC23 88.1 311 220 115

LRRC24 756 294 296 333

LRRC25 35.2 4.29 5.81 7.01

LRRC26 1580 1280 1170 1030

LRRC27 125.2425 179.735 105.17 135.425

LRRC28 277.5 321.1 71.25 161.25

LRRC29 8.46 7.54 13.4 15.4

LRRC3 68.1 84.75 8.205 484.55

LRRC30 21.2 24.2 25.4 20.4

LRRC31 184.25 25.85 5.405 3.755

LRRC32 9.23 14.1 11.8 40.5

LRRC33 25.7 48 27.2 43.5

LRRC34 9.17 302 3.89 1070

LRRC36 12.955 8.919 423.6 80.92

LRRC37A2 400 237 605 896

LRRC37A3 188.433333333333 81.5 235.566666666667 315.833333333333

LRRC37B 188.5 282.8 506.5 504

LRRC37B2 251.5 547 748 1070

LRRC38 8.103 5.32 12.292 11.543

LRRC39 6.21 16.7 23.1 92.7

LRRC3B 2.8 4.5 4.1 3.43

LRRC4 7.08 6.11 5.71 130

LRRC40 2550 4280 2400 5850

LRRC41 722.5 924.5 493.5 1193.5

LRRC42 342 674 605 335

LRRC43 3.63 24.7 5.43 8.17

LRRC45 332.8 286.45 614.65 406.75

LRRC46 71.8 111 67.6 124

LRRC47 4460 4390 4800 4630

LRRC48 2.97 4.71 4.37 3.59

LRRC49 204.5 200 360 954.5

LRRC4B 7.6 26.1 15.5 32.2

LRRC4C 2.63 4.26 16.4 3.23

LRRC50 4.05 5.72 5.44 4.585

LRRC52 10.4 8.32 11.4 7.73

LRRC55 13.4 15.5 28.6 19.9

LRRC56 674 81.7 337 232

LRRC57 650 437 591 416

LRRC58 676 634 794 856

LRRC59 12500 11500 13600 14400

LRRC6 14.532 9.939 168.3 16.97

LRRC61 1200 2090 6.3 859

LRRC63 2.81 73.6 9.66 24.5

LRRC66 2.98 11.8 4.38 53.8

LRRC67 2.81 49.7 82.9 3.41

LRRC69 30.35 519.5 23.25 366

LRRC7 3.54 5.73 5.31 4.31

LRRC70 22.3 45.4 46.1 52.2

LRRC8A 22100 15200 7370 9440

LRRC8B 444.316666666667 497.666666666667 533.089166666667 582.375

LRRC8C 20.9236363636364 50.1909090909091 157.481818181818 183.054545454545

LRRC8D 2900 1580 1530 1160

LRRC8E 311 166 395 282

LRRCC1 450 114 559 441

LRRFIP1 896.25 1141.5 1455.75 930.25

LRRFIP2 991.666666666667 1594.33333333333 2343.33333333333 1122.33333333333

LRRIQ1 3.63 5.775 65.45 30.2

LRRIQ3 110.25 96.3 288.085 380.095

LRRIQ4 6.69 5.38 4.61 5

LRRK1 13.7 14 22.5 47.9

LRRK2 8.21 5.49 4.39 23

LRRN1 104 7.12 3.9 3.25

LRRN2 224.5 213.5 207.7 543.9

LRRN3 60.1 51.6 53.2 80.5

LRRN4 9.95 4.61 13.8 3.47

LRRN4CL 10.9 5.83 25 20.8

LRRTM1 4.54 4.34 73.4 3.27

LRRTM2 6.5 6.985 14.31 12.455

LRRTM3 3.62 5.67 5.36 4.32

LRRTM4 3.26666666666667 45.1 4.81333333333333 69.1

LRSAM1 212 218 184 184

LRTM1 2.83 4.58 4.17 3.44

LRTM2 3.98 6.21 5.7 6.6

LRTOMT 32.3233333333333 79.9 228.433333333333 184.666666666667

LRWD1 2880 8930 6850 9210

LSAMP 1610 37 17.6 42.4

LSG1 3380 2410 2260 2010

LSM1 8530 12900 10400 10900

LSM10 2650 4310 4420 6160

LSM11 31.7 36.4 148 160

LSM12 2300 3120 3560 5310

LSM14A 8830 8730 12100 6670

LSM14B 394.933333333333 610.333333333333 324.233333333333 419.7

LSM2 27335 25645 19290 20460

LSM3 19931.65 16383.1 17923.55 15715.5

LSM4 6400 5230 6890 7210

LSM5 32800 18600 19300 19300

LSM6 4950 10300 5060 8990

LSM7 40800 22600 25800 38900

LSM8 8000 12300 17300 10200

LSMD1 24826.05 15560.5 31905.9 26677.7

LSP1 2673.505 2187.59 213.865 414.1

LSR 1330 288 1810 371

LSS 2383.5 1601.5 2213.5 1257

LST-3TM12 3.35 5.45 4.99 4.09

LST1 9.65 9 24.5 12.7

LTA 24 27.2 36 22.4

LTA4H 3306.5 4593 8871 13750

LTB 390 24.5 317 6.36

LTB4R 124 156 139 237

LTB4R2 202 206 187 233

LTBP1 198.6 267.35 10.815 213.5

LTBP2 10.2 5.27 4.94 4

LTBP3 105.31 107.01 82.37 57.39

LTBP4 2489 1595.5 2251 4397

LTBR 1140 1620 3010 4.14

LTC4S 13.4 16 17.1 29.8

LTF 3.11 5.07 5.56 3.78

LTK 1760 1340 817 1340

LTV1 4565 8530 2670 10241

LUC7L 1863.63636363636 1998.18181818182 2043.63636363636 3609.09090909091

LUC7L2 1910 2770 2440 5240

LUC7L3 11300 11500 9330 22300

LUM 647 7530 4.49 263

LUZP1 141 179 373 228

LUZP2 3.05 4.92 4.52 3.66

LUZP4 2.54 4.11 3.72 3.13

LUZP6 1590 1930 2780 3510

LVRN 3.48 152.32 5.165 4.175

LXN 3.25 5.28 586 1030

LY6D 2.67 4.32 3.93 3.3

LY6E 362 4.94 543 2690

LY6G5B 142 106 38.3 77

LY6G5C 62.9 47.5 125 53.4

LY6G6C 64.4 84.9 91.5 74

LY6G6D 435 371 246 414

LY6G6E 11.49 13.585 11.75 9.185

LY6G6F 3.49 19.5 12.7 4.27

LY6H 3.64 5.84 5.44 4.42

LY6K 9.1 7.72 14500 33.5

LY75 2.86 4.59 6.06 3.51

LY86 114 104 122 103

LY9 686.25 746.45 491.75 445.9

LY96 2.96 8.09 4.38 3.6

LYAR 7260 7620 4260 10700

LYG1 154 467 251 497

LYG2 3.19 5.16 4.71 56.9

LYL1 6.56 18.9 12.8 9.23

LYN 1476 1946 3118 2279

LYNX1 2231.925 2676.7875 1740.285 2267.7525

LYPD1 839.4 119.243333333333 7.44 1405.66666666667

LYPD2 8.06 118 3.93 6.45

LYPD3 14.21 10.7 21.065 18.18

LYPD4 4.195 5.195 51.135 4.3

LYPD5 5.34 6.24 5.86 4.9

LYPD6 274.5 99.45 1352 90.45

LYPD6B 157 37.1 364 77.2

LYPLA1 3595 2280 4525 4835

LYPLA2 10680 7107 7115.5 4637.75

LYPLAL1 802 516 173 715

LYRM1 1210 884 879 499

LYRM2 837 801 758 1360.5

LYRM4 4449 2983 2610 3179.5

LYRM5 163 198 134 234

LYRM7 107 255 286 335

LYSMD1 1590 881 235 1740

LYSMD2 334 567 1430 643

LYSMD3 29.1 59.2 10.9 40.7

LYSMD4 1933 1639 594.7 1067

LYST 180 43.5 9.68 213

LYVE1 3.61 5.72 4.44 43.9

LYZL1 41.1 56.2 26.5 38.7

LYZL2 4.24 7.18 6.44 4.72

LYZL4 3.981 5.344 6.849 4.08

LYZL6 3.3 5.36 4.89 3.92

LZIC 2786 2796 3678.5 1659.5

LZTFL1 43.7 223 176 607

LZTR1 483 492 296 661

LZTS1 9.94333333333333 7.24 11.1333333333333 28.82

LZTS2 889 969 1059 661

M6PR 924 1240 1990 794

MAB21L1 2.86 4.65 4.22 35

MAB21L2 38.5 1280 946 370

MACC1 3.95 6.25 5.85 4.76

MACF1 3372 2193 3173 1638

MACROD1 395 256 2150 925

MACROD2 41.5 27 58.85 67.1

MAD1L1 503 276 417 322

MAD2L1 4150 4620 3850 8100

MAD2L1BP 3740 2420 2730 3400

MAD2L2 1110 1320 3450 1910

MADCAM1 24.3 16 34 33.2

MADD 585.5 645.5 681 691

MAEA 1210 761 471 811

MAEL 3.381 5.924 4.924 3.991

MAF 1390 593 33.8 1660

MAF1 696 642 812 913

MAFA 401 453 325 369

MAFB 712 770.5 34.785 4590

MAFF 442 301.2 215.3 28.47

MAFG 1431.5 2119 1816 1325.5

MAFK 2660 1550 685 671

MAG 118 119 118 103

MAGEA1 835 519 4.54 8520

MAGEA10 2.6 41.1 3.83 4.76

MAGEA11 4.382 117.76 5.399 11.79

MAGEA12 1650 1400 5.99 9000

MAGEA13P 3.2 5.2 4.75 3.84

MAGEA2B 3054 2848.5 85.91 14505

MAGEA4 168 171 189 990

MAGEA5 3.24 5.28 4.83 3.91

MAGEA6 1606.90909090909 1211.63636363636 63.4990909090909 6880

MAGEA8 6.61 837 424 1700

MAGEA9 3.11 194 3140 3.76

MAGEB1 28.2 35.2 19.9 24.1

MAGEB10 2.85 4.59 4.2 3.5

MAGEB16 3.06 4.75 4.5 3.66

MAGEB17 46.85 14.7 13.02 19.05

MAGEB18 3.6 5.84 5.34 4.23

MAGEB2 66 757 40.4 206

MAGEB3 3.51 5.69 5.19 4.13

MAGEB4 3.09 4.98 4.53 3.72

MAGEB5 9.215 10.66 12.785 11.515

MAGEB6 6.77 4.71 36.9 25.4

MAGEC1 10.32 18.96 17.645 12.475

MAGEC2 3.785 430 4.175 17.905

MAGEC3 3.69 5.64 5.56 4.8

MAGED1 2450 4.65 2180 6990

MAGED2 529.6 2072.5 559.15 1999.5

MAGED4B 874.65 23.48 19.32 761.35

MAGEE1 74.94 48.05 76.82 165.4

MAGEE2 5.601 24.012 5.036 5.964

MAGEF1 978 1450 1540 1120

MAGEH1 5.76 7.52 13.2 581

MAGEL2 22.3 96 31.2 136

MAGI1 78.8333333333333 52.1666666666667 123.233333333333 97.3666666666667

MAGI2 121.2 11.435 7.415 39.35

MAGI3 706 1330 766 1440

MAGIX 244 728 123 532

MAGOH 4350 6980 5080 9320

MAGOHB 2958.95 5432.6 8128.2 6521

MAGT1 2431 1667 1810.33333333333 4781

MAK 32.8 35.8 23.9 181

MAK10 1820 2290 2270 2410

MAK16 4325 4817 6455 3396

MAL 15.3 13.5 127 17.8

MAL2 12500 4160 6250 3.66

MALAT1 1070 484 402 292

MALL 4.06 6.27 3860 4.92

MALT1 60.9 126 80.6 43.4

MAMDC2 2.87 4.66 4.22 3.52

MAMDC4 85 118 155 119

MAML1 8162.36363636364 9266.90909090909 8920.09090909091 10156.8181818182

MAML2 4.18 9.01 8.32 5.06

MAML3 58.5 95.4666666666667 36.6666666666667 37.1666666666667

MAMLD1 147.85 15.435 64.445 80.1

MAMSTR 301 139.5 207.95 330.5

MAN1A1 10500 6390 332 1420

MAN1A2 657 488.5 794 826

MAN1B1 785 816 1140 706

MAN1C1 43.91 92.04 38.62 166.385

MAN2A1 569 598 550 198

MAN2A2 216 526 54.9 348

MAN2B1 677 262 263 416

MAN2B2 408 265 169 186

MAN2C1 236 280 220 549

MANBA 1280 810 398 458

MANBAL 1593.33333333333 1590 2196.66666666667 2433.33333333333

MANEA 389.5 243.5 279 555

MANEAL 1103.5 479 2549 1782.5

MANF 13800 34200 24600 27400

MANSC1 287 348 1140 267

MAOA 1670 1470 21.1 163

MAOB 1330 736 5.22 18.2

MAP1A 11.3 1300 105 280

MAP1B 17.16 314.5 8.635 436.5

MAP1D 642 349 174 308

MAP1LC3A 37 36.9 32.1 35.9

MAP1LC3B 2649 1731.5 1447 2245.5

MAP1LC3C 3.02 4.91 4.46 3.69

MAP1S 13700 14000 14800 17400

MAP2 1640 1210 218 48.4

MAP2K1 3440 5690 3970 4230

MAP2K2 4850 1180 1930 2590

MAP2K3 5966.33333333333 5970 8177 3602.66666666667

MAP2K4 4250 4670 2845 4195

MAP2K5 297 273 202 300

MAP2K6 521 640 803 1030

MAP2K7 10600 4120 4870 9110

MAP3K1 2370 3040 1280 2900

MAP3K10 298 476 1130 755

MAP3K11 20000 14700 8620 5230

MAP3K12 5.61 21 70.3 15.4

MAP3K13 109.95 114.8 37.495 42.36

MAP3K14 773 348 4850 422

MAP3K15 105 135 14.6 174

MAP3K2 568.5 821.5 1063.5 562.5

MAP3K3 1140 1390 3500 4420

MAP3K4 850.5 1228.5 597 1415

MAP3K5 51.2 7.73 507.5 631.5

MAP3K6 92.4 117.9 664.5 174.5

MAP3K7 2650 1720 1700 2060

MAP3K7IP1 123.35 196.375 92.665 277.765

MAP3K7IP2 1808.5 3622.5 1194 2912.5

MAP3K7IP3 1294 724 103.5 322.5

MAP3K8 70 307 760 101

MAP3K9 279 527.5 514.5 358

MAP4 28690.9 31596.82 27563 30214.72

MAP4K1 14.3 11.8 32.4 35.7

MAP4K2 385 269 478 185

MAP4K3 134 162 74.8 107

MAP4K4 1570 1270 298 467

MAP4K5 759 864 1430 1710

MAP6 16.85 20.69 15.315 35.4

MAP6D1 882 411 471 3080

MAP7 581.5 45.9 140.75 516.5

MAP7D1 2125 3715 3970 3695

MAP7D2 71.8 76.6 29.195 412.5

MAP7D3 110.2 316.636363636364 38.8909090909091 1345.45454545455

MAP9 166.65 5.55 197.6 1279.5

MAPK1 985.7 2256 1460 1155

MAPK10 3.925 5.565 33.535 23.4

MAPK11 75.75 136 1419 128

MAPK12 120 319 388 283

MAPK13 1942 106.55 29.8 100.9

MAPK14 783 497 553 500

MAPK15 30.9 23.55 26.3 27.25

MAPK1IP1L 6300 10455 5675 12600

MAPK3 312.4 279.3 416.1 678.6

MAPK4 3.55 5.71 5.33 88.9

MAPK6 6619.5 3639.5 1764 2535.5

MAPK7 1200 2660 1630 1670

MAPK8 55.4666666666667 126.7 66.5333333333333 160.6

MAPK8IP1 162 97.7 88.7 139

MAPK8IP2 57.7 52.6 54.4 497

MAPK8IP3 2680 3030 2690 3310

MAPK9 310 499 624 839

MAPKAP1 407.333333333333 503.333333333333 348.333333333333 500.333333333333

MAPKAPK2 1060 1420 1320 1040

MAPKAPK3 1810 1790 4950 3690

MAPKAPK5 3050 4110 3600 3200

MAPKBP1 228 197 322 219

MAPKSP1 591.654545454545 644.6 353.688181818182 678.245454545455

MAPRE1 4600 7405 5790 4745

MAPRE2 1940 3530 1990 5330

MAPRE3 3.81 31.6 6.73 34.4

MAPT 2.91 18.4 110 357

MARCH1 2.62 4.26 3.86 3.22

MARCH10 3.63 18.1 15.9 14.1

MARCH11 3.03 4.87 4.48 3.7

MARCH2 179 45.3 250 83.4

MARCH3 69.895 246.8 87.95 113.5

MARCH4 3.44 5.52 5.13 4.06

MARCH5 170 253 131 372

MARCH6 1961 1916 1226 2311

MARCH7 656.7 728.4 536.3 488.2

MARCH8 18 69.8 37.2 56.7

MARCH9 707 734 1470 1510

MARCKS 30400 32400 4460 15700

MARCKSL1 3100 3890 2920 3810

MARCO 15.7 43.5 29.4 11.4

MARK1 17.7 4.47 4.06 147

MARK2 593.5 507 658 598.5

MARK3 5190 7370 6660 8390

MARK4 86.1 70.9666666666667 75.3666666666667 61.4

MARS 5064.5 5184 5022 7195.5

MARS2 117.65 79.51 72.7 129.2

MARVELD1 8.02 3420 10700 11000

MARVELD2 3260 1881 1544.5 186.1

MARVELD3 300 12.145 6.5 38.8

MAS1 3.06 25.2 4.52 3.65

MAS1L 17.7 27.2 25.6 21.1

MASP1 196.525 156.67 4.92 3.985

MASP2 38.54 6.3 14.215 18.95

MAST1 199 189 148 283

MAST2 326.45 518.6 288.7 252.705

MAST3 273 186 245 222

MAST4 220.6 197.565 125.455 164.9

MASTL 199 463 589 424

MAT1A 612 149 28.1 73.9

MAT2A 4850 4010.5 4785 6700

MAT2B 516 1282.5 2437 1897.5

MATK 4.64 11 7.75 453

MATN1 44.25 53.35 85 50.6

MATN2 32.7 43.2 54.2 23.9

MATN3 146.5 796 118.45 31.6

MATN4 6.42 13.9 64 5.35

MATR3 4150 4800 5240 7350

MAVS 2004 2991.66666666667 2227 2120

MAX 1283.13333333333 1471.46666666667 1900.66666666667 4205.66666666667

MAZ 435.5 274.7 358 1150.5

MB 2.85 4.64 6.26 5.68

MBD1 597.45 757.35 645.1 662.275

MBD2 1871.5 3264.85 6967.95 1706.5

MBD3 5760 2510 3230 6000

MBD3L1 2.86 4.6 4.21 3.48

MBD3L2 5.04 9.29 4.49 7.35

MBD4 1810 3870 2020 1590

MBD5 80.1 47.8 103 62.5

MBD6 316.25 861.5 804.5 673.5

MBIP 1450 1350 1530 1935

MBL1P1 7.61 6.84 10.7 91.2

MBL2 55.7 219 4.86 3.92

MBLAC1 223 251 458 328

MBLAC2 243 333 488 569

MBNL1 4280 4480 5350 2430

MBNL2 4068.5 1653 1325 503.4

MBNL3 2910 3350 124 288

MBOAT1 734 322 414 523

MBOAT2 3.58 5.75 74.5 743

MBOAT4 2.73 95.3 4.02 3.34

MBOAT7 1643.5 2480 5050 1536.5

MBP 1251.83333333333 141.25 65.0133333333333 27.9966666666667

MBTD1 513 311 334.5 459.5

MBTPS1 2285 3830 5125 4855

MBTPS2 252 571.7 267.8 416.4

MC1R 162 384 1170 860

MC2R 9.94 11.4 40.1 14.4

MC3R 8.06 5.73 21 18.9

MC4R 3.76 5.83 5.69 4.94

MC5R 18.6 23 22 21.9

MCAM 506 1160 5.66 1280

MCART1 841.5 761 938 999.5

MCART6 26.5 23.3 15.7 83.9

MCAT 1060 919 1230 3460

MCC 22.3 60.3 8.31 49.5

MCCC1 7470 4250 7070 2130

MCCC2 772 735 2690 631

MCCD1 28.5 19.8 23.8 22.9

MCEE 1780 1970 1710 1390

MCF2 3.49 5.58 5.69 9.41

MCF2L 274.7 29.73 83.6075 133.1325

MCF2L2 3.75 5.74 5.66 4.92

MCFD2 3690.09090909091 5018.72727272727 1930.27272727273 3723.90909090909

MCHR1 9.38 6.365 11.96 9.05

MCHR2 7.505 11.85 15.06 11.665

MCL1 2990 2960 1500 2590

MCM10 637 1030 884 1520

MCM2 6850 8190 9880 9460

MCM3 35400 31400 18800 35500

MCM3AP 2224 706.2 1749 2434

MCM3APAS 136 144 199 341

MCM4 4810 4500 6640 8480

MCM5 3465 2650 4010 4175

MCM6 8720 9390 10100 14800

MCM7 24035 45470 30560 41890

MCM8 2910 6140 3730 5130

MCM9 166 148 241 323

MCOLN1 402 155 194 241

MCOLN2 60.6 44.5 60.7 540

MCOLN3 23.42 6.186 44.54 82.67

MCPH1 49.1 65 64.7 68.1

MCRS1 1500 1920 2550 3240

MCTP1 61.55 195.5 205 28.1

MCTP2 4.915 4.855 4.52 3.705

MCTS1 8510 11367.9090909091 8116.31818181818 27255.5454545455

MDC1 4620 3860 3340 7590

MDFI 15.6 539 1010 20.2

MDFIC 109 936 780 387

MDGA1 105.295 103.6 97.905 107.65

MDGA2 41.9 1760 344 106

MDH1 43300 35963.6363636364 34390.9090909091 46345.4545454545

MDH1B 4.18 6.43 42.3 20.3

MDH2 28600 19900 22700 28700

MDK 23300 39500 89.8 5040

MDM1 57.7333333333333 77.8083333333333 128.75 127.916666666667

MDM2 115.583333333333 136.441666666667 141.391666666667 240

MDM4 615.272727272727 330.545454545455 497.181818181818 616

MDN1 327 268 227 466

MDP1 1450 2600 2040 4290

MDS2 3.2 5.2 4.74 3.9

ME1 2470 1780 383 768

ME2 1210 1390 3410 1590

ME3 6.22909090909091 85.7636363636364 13.21 4.97272727272727

MEA1 8890 8490 9460 12800

MEAF6 3545.45454545455 3204.54545454545 3639.09090909091 4795.45454545455

MECOM 347.9 572.233333333333 4062.33333333333 410.5

MECP2 339.766666666667 579.666666666667 436.766666666667 1676

MECR 465 446 1210 1590

MED1 273 231 247.5 363.5

MED10 746.7 982.7 797 936.6

MED11 2650 1870 1870 3050

MED12 263 256 137 445

MED12L 3.01 4.8 41.6 108

MED13 4170 3050 2200 5230

MED13L 895 1010 971 2200

MED14 445.58 652.55 428.35 1426.935

MED15 689 856 648 709

MED16 853 339 419 562

MED17 244 534 220 464

MED18 332.5 285 274 471

MED19 2590 8260 2770 3740

MED20 847.25 811.15 565.55 815.3

MED21 611 978 1870 1930

MED22 379.5 438 302.5 481

MED23 206.866666666667 235.366666666667 138.966666666667 345.666666666667

MED24 1230 1010 1130 2150

MED25 1910 1595 3590 3010

MED26 2750 3000 2720 3890

MED27 391 523 546 922

MED28 230 172 52.6 175

MED29 208 356 273 276

MED30 898 656 1400 936

MED31 46.8 60.3 26 129

MED4 2012 2520 3360 5715

MED6 761 805 773 1610

MED7 520 614 907 862

MED8 1337 1855 1210 2050

MED9 2400 2650 1480 2360

MEF2A 2960 2360 1680 2140

MEF2B 622.5 529.5 494 663.5

MEF2C 357 461 9.47 958

MEF2D 1423.5 1480 1128 1262

MEFV 142 146 93.7 156

MEG3 514.533333333333 569.033333333333 435.833333333333 424.8

MEG8 12.8 28.7 10.1 19.3

MEGF10 3.36 5.41 4.98 4.86

MEGF11 90.05 114.8 121.75 149.8

MEGF6 3.18 5.18 1200 26.9

MEGF8 1650 1970 2540 4290

MEGF9 597 1520 1030 352

MEI1 43.7 5.77 19.5 65.1

MEIG1 49.2 10.6 181 25.6

MEIS1 133 200 348 749

MEIS2 1780 1250 750 725

MEIS3 2.69 4.37 3.95 20.1

MEIS3P1 595 829 819 312

MELK 3960 10600 7440 4250

MEMO1 773.5 1174.5 1429.5 1296

MEN1 700.5 582 648 1440

MEOX1 4.423 5.588 65.8 6.613

MEOX2 3.72 5.91 5.46 4.4

MEP1A 348 707 15.3 23.7

MEP1B 2.73 4.43 4.01 3.35

MEPCE 562 854 1310 964

MEPE 3.4 5.52 5.06 4.15

MERTK 82.6 657 824 315

MESDC1 3380 2790 2130 3470

MESDC2 715 1230 229 636

MESP1 289 162 46.7 1330

MESP2 229 405 184 485

MEST 3650 1440 2170 3.56

MESTIT1 5.565 7.93 8.255 6.715

MET 17680 13380 34030 4790

METAP1 4620 8520 6000 8060

METAP2 11072 18875 18338 22570

METRN 2720 2240 12200 3210

METRNL 1300 409 113 1630

METT10D 2453 1117.5 2783 3389

METT11D1 2400 1980 2200 3340

METT5D1 594.5 903 363.5 540.5

METTL1 1260 2245 1022 2680

METTL10 537.8 1231.83333333333 734.533333333333 275.166666666667

METTL11A 3840 4710 6300 7920

METTL11B 5.97 5.35 5.13 17

METTL12 421 519 815 335

METTL13 16000 9790 9930 16700

METTL14 640.4 656.2 254.7 714.8

METTL2A 601 642 935 1080

METTL2B 1000 2290 1970 3130

METTL3 4940 5310 3100 7100

METTL4 390 666 261 335

METTL5 8325 9780 9675 8565

METTL6 750.85 706.2 1432.7 834.9

METTL7A 2950 2760 8270 2040

METTL7B 606 388 854 444

METTL8 2255 1641.5 2005 1512

METTL9 178 179 110 271

MEX3A 6310 3820 309 1840

MEX3B 145 144 10.3 242

MEX3C 2900 3710 4130 2080

MEX3D 3025 3285 2620 2226

MFAP1 4700 4590 5250 5550
[truncated: 277,311 more chars]
